# Interventions for negative symptoms in schizophrenia: efficacy and clinical interpretability in a meta-analysis of 451 randomized controlled trials

**DOI:** 10.1038/s41380-026-03543-1

**Published:** 2026-03-23

**Authors:** Stefano Damiani, Riccardo Stefanelli, Lydia Fortea, Aldo D’Imperio, Matteo Calò, Francesco Casarini, Andrea Crippa, Cecilia Maria Esposito, Roberto Leggi, Marika Orlandi, Sara Patron, Alessandro Peviani, Alessandro Piccolo, Umberto Provenzani, Fabrizio Santilli, Cecilia Spallarossa, Evangelos Papanastasiou, Matteo Cella, Rashmi Patel, Marco Solmi, Silvana Galderisi, Stefan Leucht, Daniel Stahl, Joaquim Radua, Paolo Fusar-Poli

**Affiliations:** 1 University of Pavia, Department of Brain and Behavioral Sciences, Pavia, Italy; 2 Institut d’Investigacions Biomèdiques August Pi I Sunyer (IDIBAPS), Barcelona, Spain; 3 Department of Medicine, University of Barcelona, Institute of Neuroscience, Barcelona, Spain; 4 Helsingborg General Hospital, Adult Psychiatric Clinic, Helsingborg, Sweden; 5 Lund University, Division of Psychiatry, Clinical Sciences Helsingborg, Lund, Sweden; 6 IRCCS Fondazione Ca’ Granda Ospedale Maggiore Policlinico, Department of Neurosciences and Mental Health, Milan, Italy; 7 IRCCS Mondino Foundation, Pavia, Italy; 8 HMNC Brain Health, Munich, Bavaria Germany; 9 South London and Maudsley NHS Foundation Trust, London, UK; 10 Institute of Psychiatry, Psychology & Neuroscience, King’s College London, London, UK; 11 Department of Psychiatry, University of Cambridge, Cambridge, UK; 12 SCIENCES lab, Department of Psychiatry, University of Ottawa, Ontario, Canada; 13 Regional Centre for the Treatment of Eating Disorders and On Track: The Champlain First Episode Psychosis Program, Department of Mental Health, The Ottawa Hospital, Ontario, Canada; 14 Ottawa Hospital Research Institute (OHRI) Clinical Epidemiology Program University of Ottawa, Ottawa, Ontario Canada; 15 Department of Child and Adolescent Psychiatry, Charité Universitätsmedizin, Berlin, Germany; 16 University of Campania “Luigi Vanvitelli”, Naples, Italy; 17 Department of Psychiatry and Psychotherapy, University of Munich, Munich, Germany; 18 Department of Psychosis Studies, King’s College London, London, UK; 19 Centro de Investigación Biomédica en Red de Salud Mental (CIBERSAM), Instituto de Salud Carlos III, Madrid, Spain; 20 Early Psychosis: Interventions and Clinical-detection (EPIC) Laboratory, Department of Psychosis Studies, Institute of Psychiatry, Psychology and Neuroscience, King’s College London, London, UK; 21 Outreach and Support in South-London (OASIS) service, South London and Maudlsey (SLaM) NHS Foundation Trust, London, UK

**Keywords:** Schizophrenia, Prognostic markers

## Abstract

Negative symptoms (avolition, anhedonia, asociality, blunted affect, and alogia) are among the most disabling features of schizophrenia spectrum disorders. In the absence of treatment consensus guidelines, this PRISMA-compliant meta-analysis (PROSPERO: CRD42024613967) evaluated efficacy and clinical significance of interventions targeting this dimension. Web of Science/PsycInfo databases were searched from inception to December 2024. Five categories (antipsychotics, other pharmacological agents, brain stimulation, psychosocial, and lifestyle interventions) were analyzed across short/middle/long follow-up times. Categories were divided into 27 subcategories (e.g., ‘other pharmacological agents’ divided in 14 subcategories including antidepressants, antibiotics, immunomodulators) regardless of follow-up, assessing evidence with GRADE criteria. The primary outcome was the change in negative symptom severity, measured with validated scales (PANSS/SANS/BPRS/CAINS/BNSS) as standardized mean differences (SMD). A clinically meaningful SMD threshold was estimated from the regression between SMD and one-point reductions on the Clinical Global Impression-Severity (CGI-S) scale. This study meta-analyzed 451 trials (n = 42566). The clinically meaningful threshold, obtained from 122 trials reporting CGI-S, was SMD ≥ 0.457. In 214 high-quality studies (n = 19746), 2 category-by-follow-up combinations and 16 subcategories showed significant improvements. Clinically meaningful SMDs for subcategories were antibiotics (0.95; CI: 0.18–1.71; moderate-GRADE), integrated psychosocial interventions (0.93; CI: 0.53–1.33; very-low-GRADE), antidepressants (0.76; CI: 0.33–1.19; moderate-GRADE), physical activity (0.68; CI: 0.39–0.96; very-low-GRADE), transcranial current stimulation (0.52; CI: 0.17–0.86; low-GRADE), and immunomodulators (0.47; CI: 0.26–0.67; high-GRADE), typically as adjuncts to antipsychotics. Heterogeneity was the main limitation. While selected interventions may yield meaningful improvements, more rigorous designs are needed to identify reliable, personalized and scalable treatment options.

## Introduction

Negative symptoms (NS) are hallmark features of schizophrenia spectrum disorders and include avolition (reduced initiation and persistence in goal-directed activities), anhedonia (reduced ability to experience pleasure), asociality (lack of interest in social interactions), blunted affect (reduced expression of emotion) and alogia (poverty of speech) [1, 2]. These symptoms can be clustered into two factors: diminished expression (blunted affect, alogia) and avolition/apathy (anhedonia, asociality, avolition). NS often emerge early in life, persist across all illness stages, and worsen with psychotic episodes and chronicity [3–5].

NS place a significant burden on people living with schizophrenia, impairing quality of life [6], goal-directed behavior [7], cognition [8] and socio-occupational functioning [9]. While many treatment options are available, NS remain very challenging to treat. Possible explanations include the lack of effective, tailored interventions, as many patients continue to experience significant NS despite (standard) antipsychotic treatment. Also, NS are associated with poor illness insight, leading to reduced medication adherence and delayed access to care [8]. For these reasons, NS still represent an unmet clinical need and therefore a critical priority for improving the overall prognosis of people with schizophrenia.

Over the past decades, numerous meta-analyses have examined the efficacy of specific interventions in improving NS in schizophrenia. Antipsychotics have generally demonstrated small effect sizes across studies, including those focusing on monotherapy, augmentation strategies, and comparisons among individual agents [10–14]. Reviews on adjunctive pharmacological treatments, such as antidepressants [15, 16] and memantine [17, 18], reported small-to-moderate benefits, though results were often limited by heterogeneity and short trial durations. Brain stimulation techniques, particularly transcranial magnetic and direct current stimulation, showed moderate improvements in NS [19–21], albeit with variability in protocols and modest sample sizes. Psychosocial interventions, including cognitive remediation [22–24], cognitive-behavioural therapy [25, 26], and social skills training [27], yielded small-to-moderate effects even when investigating NS-adapted strategies [26–28]. Lastly, lifestyle interventions such as aerobic or mind-body exercise also demonstrated moderate efficacy across trials [29–31].

To date, only Fusar-Poli and colleagues’ meta-analysis [32] in 2013 has compared treatment efficacy on NS across multiple treatment strategies, finding that while a range of interventions showed statistically significant effects, none of them reached the threshold for clinical relevance. The novelty in their methodology was indeed to link standardized mean differences (SMDs) in symptom improvement to changes on the Clinical Global Impression–Severity (CGI-S) scale, allowing estimation of real-world clinical benefits. No subsequent study has applied this methodology across intervention categories, leaving the last decade of research in this area without systematic evaluation.

Therefore, in the present study we meta-analyzed the statistical and clinical significance of all current interventions for NS in schizophrenia, following Fusar-Poli and colleagues’ methodology [32]. In addition to that, we grouped interventions by category (e.g., antipsychotics) and follow-up times, while also considering subcategories (e.g., first, second, and third generation antipsychotics). To further explore the clinical relevance of the findings, we assessed the percentage of improvement in NS from baseline to follow-up [33], and evaluated coherence between instruments used to measure NS treatment efficacy.

## Methods

### Study design

This systematic review and pairwise meta-analysis (PROSPERO protocol number CRD42024613967) was conducted according to the Preferred Reporting Items for Systematic Reviews and Meta‐Analyses (PRISMA 2020) [34].

### Search strategy and review criteria

We systematically searched PsycINFO and all Web of Science databases (see Supplementary Methods 1.1) from inception to November 30, 2024, limiting results to peer-reviewed journal articles and excluding grey literature [35]. References from prior systematic reviews/meta-analyses were manually screened for additional eligible studies. The complete search string is provided in Supplementary Table 1.

Inclusion/exclusion criteria followed the Patient/Population, Intervention, Comparison, and Outcome (PICO) guidelines [36]. Population: individuals with schizophrenia spectrum disorders, with ≥80% of the sample diagnosed via DSM/ICD as schizophrenia, schizophreniform, schizoaffective, or delusional disorder, alone or with secondary psychiatric comorbidities. Trials including first-episode psychosis were eligible only if ≥80% of participants received at least one of these four diagnoses during the trial. Studies were excluded if >20% of participants were diagnosed with substance-induced psychotic disorder, brief psychotic disorder, catatonia, and/or unspecified schizophrenia spectrum or other psychotic disorders. Intervention-Comparison: any treatment for schizophrenia symptoms. Only randomized controlled trials (RCTs) with active vs non-active control (no treatment, placebo/sham, or waiting list) were included. Studies that compared only active interventions (without a non-active control) or that compared a non-active control against mixed interventions (e.g., combined pharmacological and psychological treatments) were excluded. Non-randomized designs (e.g., open-label trials/extensions) were excluded. “Treatment as usual” was permitted in both arms; many trials randomized add-on interventions. Each arm required ≥10 participants at randomization (retained at follow-up even if <10). Studies without arm-level sample size or insufficient data to calculate/estimate mean pre-post change and SD were excluded. Outcome measurement: unadjusted data from the most used scales to assess NS: Positive and Negative Symptoms Scale (PANSS) [37], Scale for the Assessment of Negative Symptoms (SANS) [38], Brief Psychiatric Rating Scale (BPRS) [39], Brief Negative Symptoms Scale (BNSS) [40], Clinical Assessment for Negative Symptoms (CAINS) [41] (see Supplementary Methods 1.1).

Due to the high resource cost of including studies in languages other than English, such articles were excluded a priori. Potential duplicate inclusion of samples was systematically assessed by comparing authorship, publication dates, and sample characteristics (age, sample size, and baseline severity) across studies evaluating the same intervention. When multiple studies referred to the same sample, article selection was based on the following hierarchical set of criteria (from highest to lowest priority): (i) the presence of scales for assessing NS in schizophrenia, (ii) largest sample size, (iii) data quality regarding treatments (i.e., studies with a higher number of active intervention arms or dosages/sessions arms of the same intervention), (iv) the quality of demographic data (age, sex, description of treatment as usual). Coherently, we considered post-hoc studies provided that their samples did not overlap with those of other included studies, while pooled analyses were excluded.

### Study assessment, data extraction and quality assessment

In the first screening phase, titles and abstracts were assessed. Study protocols, posters, short papers, communications, and articles that were unrelated to clinical trials for treating schizophrenia spectrum disorders were excluded.

The eligibility of the remaining studies was assessed after reading the full-text. Each title/abstract and full-text was assessed by two independent screeners. The workload was divided among multiple reviewer pairs (reviewers list: AD, MC, FC, RL, MO, CME, ELLL, SP, APe, APi, FS, CS) and discrepancies were resolved via consensus with a third reviewer (SD, UP).

Two raters independently extracted relevant data after a training phase to harmonize data extraction provided by SD and UP. At the end of the extraction phase, the final datasets were cross-checked for consistency by a third reviewer (AD or RS), and inconsistencies were again resolved by consensus with a senior reviewer (SD, UP).

We extracted the following study-level variables: sample main diagnosis, diagnostic criteria tools (DSM/ICD), percentage of sample with main diagnosis, maximum follow-up (in weeks), treatment category, and the scales used to assess negative symptoms. Participant-level variables were sample size, mean age and sex, intervention (active versus non-active treatment), mean negative, positive and total symptoms at baseline and follow-up, mean difference and percentage of change in negative symptoms from baseline to follow-up. For each study, we extracted the longest available follow-up timepoint to capture sustained effects on negative symptoms, which we deemed more indicative of clinically meaningful change. Studies were then categorized into follow-up subgroups (<7 weeks, 7–24 weeks, >24 weeks) based on this longest timepoint. No study contributed more than one timepoint to the analyses.

For each study, we extracted the between-group difference in negative symptom severity from baseline to follow-up. When multiple negative symptom scales were reported, we applied the following hierarchy to select one: PANSS-Negative Factor Score > SANS > BPRS > BNSS > CAINS. This prioritization reflected both frequency of use across studies and comparability with previous meta-analytic literature. If multiple timepoints were reported, only the longest available follow-up was extracted (see Statistical Analysis).

To maximise comparability between trials categories, only data reporting unadjusted scores from the negative symptoms scales listed above were extracted (authors were contacted when the article included adjusted scores only, see Supplementary Methods 1.3). Also, variables that could be reported in different metrics (e.g., duration of illness in years, months) were converted to a common metric (e.g., duration of illness in years). When outcome measures were only reported graphically, numerical data were extracted from the images using the GIMP software [42].

A quality assessment of each individual study was conducted by two independent raters using the Cochrane Collaboration’s tool for assessing the risk of bias in randomised trials (RoB) [43].

### Groups definition

Interventions were grouped into 5 macro-categories (antipsychotics, other pharmacological agents, brain stimulation, psychosocial, and lifestyle interventions) and 3 follow-up times (<7 weeks=short, 7–24 weeks=medium/middle, >24 weeks=long). Since absolute standards defining short/middle/long follow-up times are unavailable, these windows were selected to maintain a balanced distribution of studies across categories/follow-up times.

Each category was further divided into subcategories; for example, the “antipsychotics” category was divided into first-, second-, and third-generation antipsychotics. We did not further stratify subcategories by follow-up duration given the limited number of studies per group (often <10 studies). Such an approach would have reduced statistical power, widened confidence intervals, and increased the risk of spurious findings. Moreover, analyses at the category-by-follow-up level revealed broadly consistent SMDs across follow-up durations, supporting the decision to retain a single pooled estimate for each subcategory.

The search string (Supplementary Table 1) displays the specific category/subcategory of each intervention.

### Strategies for data synthesis

#### Primary outcome

The primary outcome was the difference in NS severity from baseline to follow-up between active and non-active treatment groups. Negative standardized mean differences (SMD) favored active versus control treatment groups. If multiple scales were used to measure NS within a single study, the hierarchy was: PANSS, SANS, BPRS, BNSS, CAINS (based on frequency across studies).

For inclusion, studies had to report baseline and follow-up scores or change scores, with means and SDs. When SDs of change scores were missing, they were estimated from baseline and follow-up SDs assuming correlations of r = 0.3/0.5/ 0.7 between pre-post scores. For the primary analysis, we assumed a correlation of r = 0.5, in accordance with Cochrane recommendations [44], and performed sensitivity analyses using r = 0.3 and r = 0.7 to assess the robustness of the results.

Meta-analyses were conducted for categories/subcategories assessed in ≥3 studies [45], using a random-effects model to account for heterogeneity [46]. Unlike main categories, subcategories were not further stratified by follow-up time to preserve meta-analytic power, as most included a limited number of studies. Benjamini-Hochberg False-Discovery Rate correction [47] was applied to correct SMD for multiple comparisons both for categories-by-follow-up combinations and subcategories results.

#### Clinical significance thresholds

Statistical significance does not imply clinical significance [32]. As a secondary outcome, we assessed the magnitude of NS change from baseline to follow-up using two clinical significance thresholds.

First, we computed the SMD threshold corresponding to the minimally detectable clinical improvement, defined as the predicted SMD of NS when the mean CGI-S improvement difference between active and control groups is 1 [32]. SMD threshold was derived from the unstandardized beta coefficient of the linear regression between SMD (dependent variable) and CGI-S reduction.

Second, following Leucht and colleagues [33], a 27% PANSS NS reduction from baseline in intervention arms corresponds to minimal improvement on the CGI-I scale. For this, SANS scores were converted to PANSS scores using [48]:

1. SANS_total available: PANSS_negative = 7.1196 + 0.3362 × SANS_total
2. SANS_total not available: PANSS_negative = 6.7515 + 1.0287 × SANS_summary

We then calculated percentage change in PANSS scores from baseline to follow-up to determine their clinical significance.

### High-quality studies

Study quality was assessed with the Risk of Bias (RoB) tool, version 1 [43]. The same pairs of reviewers who extracted study data also independently assessed RoB. To ensure consistency given the central role of this variable in meta-analytic models, a senior author (SD or UP) reviewed the RoB ratings across all included studies.

The quality of evidence at the meta-analytic level was defined following the Grading of Recommendations Assessment, Development, and Evaluation (GRADE) guidelines [49]. GRADE downgrading criteria are reported in Supplementary Methods 1.4.

Due to low GRADE certainty across studies, we focused on high-quality studies (i.e., those with an overall “Low” RoB across items, see also Supplementary Methods 1.4), reporting the whole-sample results in the Supplementary Results 2.2 for completeness. When selecting high-quality studies, the RoB item “participant and personnel blinding” was not considered in studies evaluating psychosocial, lifestyle, and brain-stimulation categories, as these designs almost invariably preclude a low risk of bias for blinding. However, all RoB domains were retained when determining the overall GRADE certainty in order to ensure consistency across intervention categories.

### Sensitivity analyses

Although different scales measure the same NS, their differences can impact outcomes [50]. We analyzed studies using both PANSS and SANS to compare effect sizes via a multivariate meta-analysis.

In addition, we conducted random-effects meta-regressions to examine whether treatment effects on NS were moderated by mean age, follow-up duration, or baseline NS severity. These analyses were performed for each subcategory and restricted to high-quality studies (Supplementary Methods 1.5).

## Results

### Study selection

**Fig. 1:**
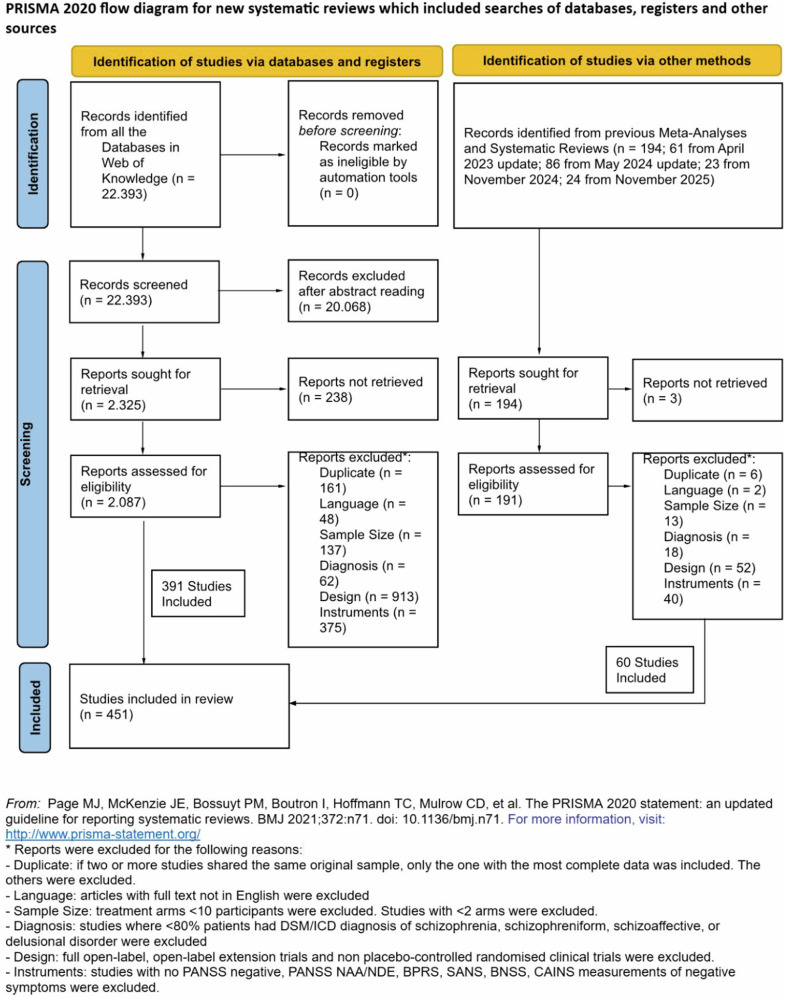
PRISMA flow chart. Preferred Reporting Items for Systematic reviews and Meta-Analyses (PRISMA 2020) flow diagram.

Our search uncovered 451 studies for a total of k = 572 arms comparing active interventions with control groups (i.e., nothing, placebo/sham or waiting list) (see PRISMA flow diagram, Fig. 1). A total of 25151 patients under active treatment and 17415 patients under control treatment were analyzed. Male percentage was 67.49% (active treatment) and 67.00% (control). Mean age was 38.63 ± 7.83 (SD) (active-treatment) and 39.03 ± 7.82 (control). PANSS_neg and SANS scales accounted for 96.25% of data. Drop-out rates were 16.66% (active treatment) and 14.89% (control). Individual study characteristics are provided in Supplementary Table 12.

Of the 572 arms, k = 266 (48.54%) had an overall “Low” RoB, subdivided as follows: antipsychotics: k = 24 (23.53%); other pharmaceutical agents: k = 128 (51.41%); brain stimulation: k = 45 (81.81%); psychosocial interventions: k = 63 (48.54%); lifestyle interventions: k = 6 (35.29%).

Reported effect sizes assume a correlation of 0.5 between baseline and follow-up values (results for 0.3/0.7 correlations reported in Supplementary Tables 3, 4, 8, 9) [44].

### Clinical significance threshold (SMD) estimation

**Fig. 2:**
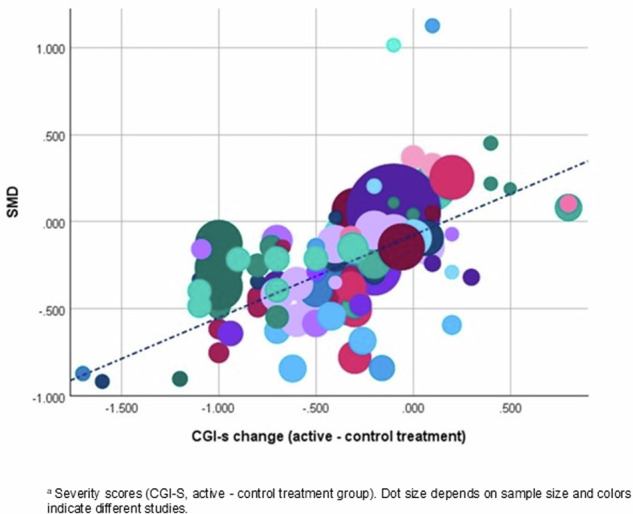
Clinically meaningful SMD threshold. Scatter plot displaying the correlation between standardized mean differences (SMD) related to negative symptom changes from baseline to follow-up (active vs control treatment group) and improvements in the Clinical Global Impression Scale^a^.

The correlation between SMD and CGI-S change was computed over 122 studies and was moderate-to-high (Pearson r = 0.566; p < 0.001; 95% CI: 0.431–0.676). The SMD threshold corresponding to a change of 1 point in the CGI-S improvement difference between active and control groups (unstandardized beta) was −0.457. A similar value (unstandardized beta: −0.453; n = 44) was found when restricting the analysis to high-quality studies. The −0.457 threshold was therefore considered as the main indicator of clinical significance when assessing treatment effectiveness (Fig. 2).

### Category-by-follow-up combinations

Due to important study quality issues (see GRADE section below), only results related to the high-quality studies are reported in the main text (see Supplementary Result 2.2 and Supplementary Table 8 for whole-sample results).

**Fig. 3:**
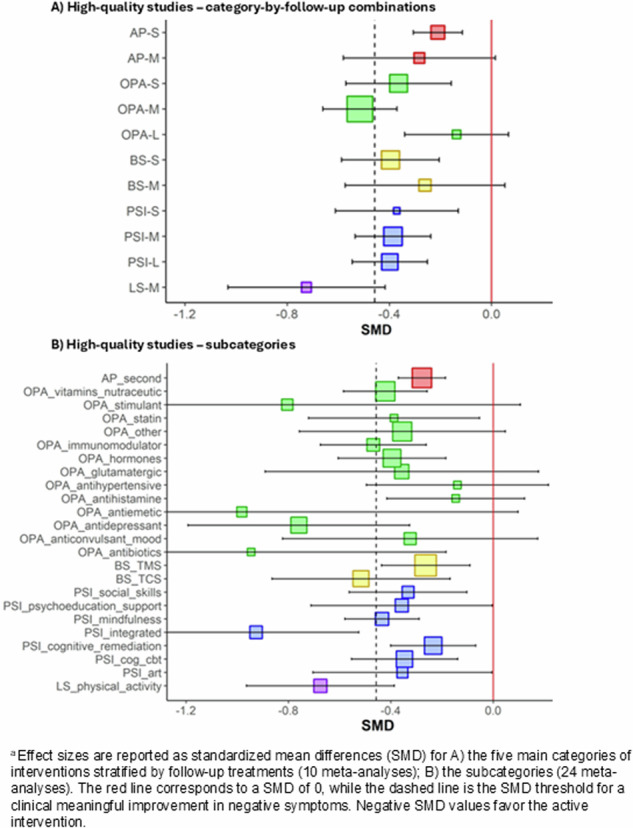
Mean efficacy of active interventions. Forest plots displaying the mean efficacy of active interventions versus control comparators for negative symptoms in schizophrenia in high-quality studies^a^.

For category-by-follow-up combinations (short, middle, long follow-up time), it was possible to conduct 11 pairwise meta-analyses. These were: antipsychotics-short (k = 16), antipsychotics-middle (k = 8), lifestyle-middle (k = 7), other pharmacological agents-short (k = 33), other pharmacological agents-middle (k = 89), other pharmacological agents-long (k = 5), psychosocial interventions-short (k = 4), psychosocial interventions-middle (k = 38), psychosocial interventions-long (k = 26), brain stimulation-short (k = 35), brain stimulation-middle (k = 11). The two clinically meaningful effect sizes were observed for the lifestyle-middle (k = 7; n = 403; SMD = −0.72; CI: −1.03–−0.41; p < 0.001; I² = 52.36%) and other pharmacological agents-middle (k = 89; n = 6243; SMD = −0.52; CI: −0.66–−0.37; p < 0.001; I² = 86.76%). Full results are reported in Fig. 3A and Supplementary Table 3.

Effect sizes remained stable across assumed correlations between baseline and follow-up scores (r = 0.3, 0.5, 0.7), with only minor increases observed at higher r values. These variations minimally affected statistical and/or clinical significance. For example, antipsychotic-middle combination gained statistical significance at r = 0.3, whereas psychological (short, middle, and long follow-up) and brain stimulation (short follow-up) interventions reached clinical significance at r = 0.7 (Supplementary Table 3).

### Subcategories

As for categories-by-follow-up time combinations, only high-quality studies results on subcategories are reported here in the main text (see Supplementary Result 2.2 and Supplementary Table 9 for whole-sample results).

The final set of 24 subcategories included: second generation antipsychotics (k = 25), antibiotics (k = 3), anticonvulsant_mood_stabilizers (k = 6), antidepressants (k = 14), antiemetics (k = 4), antihistamines (k = 3), antihypertensives (k = 3), glutamatergics (k = 10), hormones (k = 19), immunomodulators (k = 7), statins (k = 3), stimulants (k = 5), vitamins-nutraceutics (k = 24), other pharmacological agents not included in one of the previous subcategories (k = 23), transcranial current stimulation (TCS, k = 14), transcranial magnetic stimulation (TMS; k = 33), art therapy (k = 5), cognitive and cognitive-behavioral therapy (k = 15), cognitive-remediation therapy (k = 18), integrated psychosocial interventions (k = 7), mindfulness (k = 8), psychoeducation-support (k = 8), social skills (k = 6) and physical activity (k = 8).

Clinically significant effects were observed for the following subcategories: antibiotics (k = 3; n = 158; SMD = –0.95, 95% CI –1.71––0.19; p = 0.026; I² = 76.95%), integrated psychosocial interventions (k = 7; n = 468; SMD = –0.93, 95% CI –1.33 to –0.53; p < 0.001; I² = 72.78%), antidepressants (k = 14; n = 834; SMD = –0.76, 95% CI –1.19 to –0.33; p = 0.002; I² = 58.90%), physical activity (k = 8; n = 434; SMD = –0.68, 95% CI –0.96 to –0.39; p < 0.001; I² = 50.18%), transcranial current stimulation (k = 14; n = 725; SMD = –0.52, 95% CI –0.86––0.17; p = 0.007; I² = 32.98%), and immunomodulators (k = 7; n = 379; SMD = –0.47, 95% CI –0.67 to –0.26; p < 0.001; I² = 0.00%). Notably, overall sample sizes in these six subcategories were small. Full results are reported in Fig. 3B and Supplementary Table 4.

Effect sizes remained stable across assumed correlations between baseline and follow-up scores (r = 0.3/0.5/0.7). Again, with minor increases observed at higher r values. No changes in statistical significance were observed, except for p values in statin and psychoeducation-support which became > 0.05 at r = 0.3 and at r = 0.7, respectively. Immunomodulators and mindfulness did not reach the clinically meaningful threshold at r = 0.3, whereas vitamins-nutraceutics achieved it at r = 0.7 (Supplementary Table 4).

### Percentage of improvement from baseline scores

**Fig. 4:**
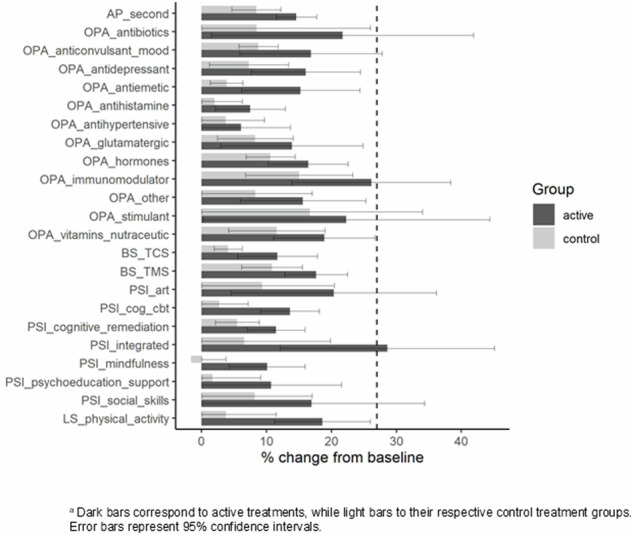
Mean percentage of improvement from baseline in active and control treatment arms. Bar plots illustrating the percentage of improvement from baseline for each subcategory of intervention in high-quality studies^a^.

Integrated psychosocial interventions (28.61%; CI: 12.11–45.11%) were the only subcategory to reach the 27% threshold for a clinically meaningful improvement. Improvements in NS > 20% were reached by immunomodulator (26.13%; CI: 13.88–38.39%), stimulant (22.28%; CI: 0.19–44.36), antibiotic (21.71%; CI: 1.55–41.86%) and art therapy (20.35%; CI: 4.54–36.15%) subcategories. Improvements in control groups were highly heterogeneous across subcategories (Fig. 4, Supplementary Table 5).

Whole-sample results are reported in Supplementary Materials (Results 2.2, Fig. 2 and Table 10).

### Data quality

Table 1 displays the subcategories’ GRADE scores (n° high=3, moderate=6, low=4, very-low=11) for high-quality studies. The most effective interventions varied in evidence quality: immunomodulators (high), antidepressants and antibiotics (moderate), transcranial current stimulation (low), integrated psychosocial interventions and physical activity (very low). Information on RoB, heterogeneity and publication bias are provided in Supplementary Materials (Results 2.3 and 4, Table S2 for whole-sample results).

**Table 1:** GRADE scores indicating the certainty of evidence for each intervention subcategory (high-quality studies only)^a^.

Cat.	Subcategory	RoB	Inconsistency	Indirectness	Imprecision	Publication bias	DR gradient	GRADE
AP	AP_second	-	-	-	-	-	NA	high
BS	BS_TCS	−1	−1	-	-	-	NA	low
	BS_TMS	−1	−1	-	-	-	NA	low
LS	LS_physical_activity	−2	−1	-	-	-	NA	very low
OPA	OPA_antibiotics	-	−1	-	-	-	NA	moderate
	OPA_anticonvulsant_mood	-	−1	-	−1	−1	NA	very low
	OPA_antidepressant	-	−1	-	-	-	NA	moderate
	OPA_antiemetic	-	−2	-	−1	-	NA	very low
	OPA_antihistamine	-	-	-	-	-	NA	high
	OPA_antihypertensive	-	-	-	−1	-	NA	moderate
	OPA_glutamatergic	-	−2	-	−1	-	NA	very low
	OPA_hormones	-	−1	-	-	-	NA	moderate
	OPA_immunomodulator	-	-	-	-	-	NA	high
	OPA_other	-	−2	-	−1	-	NA	very low
	OPA_statin	-	−1	-	-	-	NA	moderate
	OPA_stimulant	-	−2	-	−1	-	NA	very low
	OPA_vitamins_nutraceutic	-	−1	-	-	-	NA	moderate
PSI	PSI_art	−2	−1	-	−1	-	NA	very low
	PSI_cog_cbt	−2	−1	-	-	-	NA	very low
	PSI_cognitive_remediation	−2	-	-	-	-	NA	low
	PSI_integrated	−2	−1	-	-	-	NA	very low
	PSI_mindfulness	−2	-	-	-	-	NA	low
	PSI_psychoeducation_support	−2	−1	-	-	-	NA	very low
	PSI_social_skills	−2	−1	-	-	-	NA	very low

^a^*AP* antipsychotic, *LF* lifestyle, *OPA* other pharma agent, *PSI* psychosocial intervention, *BS* brain stimulation.

*Cat*. category of intervention, *RoB* risk of bias, *DR grad*. dose-response gradient, *GRADE* = final score indicating the certainty of meta-analytic evidence.

### Sensitivity analyses

Twenty-one studies (4.78% of the total) used both scales to measure changes in NS. PANSS_neg and SANS yielded similar SMDs (r = 0.5: SMD_PANSS_neg_ = −0.412, SMD_SANS_ = −0.373, p = 0.485). Results were confirmed for r = 0.3 (SMD_PANSS_neg_ = −0.354, SMD_SANS_ = −0.311, p = 0.574) and r = 0.7 (SMD_PANSS_neg_ = −0.506, SMD_SANS_ = −0.477, p = 0.458) (Supplementary Table 6 and 11 for whole-sample results).

Concerning meta-regressions, longer follow-up duration was associated with larger treatment effects in anticonvulsants (β = 0.23, p = 0.001), whereas a smaller but opposite trend was observed for second-generation antipsychotics (β = −0.02, p = 0.021) and social skills interventions (β = −0.02, p = 0.009). Age was negatively associated with treatment effects in integrated psychological interventions (β = −0.03, p = 0.019) and positively associated with glutamatergic agents (β = 0.10, p = 0.039). Baseline NS showed a positive association with treatment effects in antiemetics (β = 0.35, p < 0.001). The remaining associations were non-significant (Supplementary Table 7).

## Discussion

This study compared the efficacy of all known intervention approaches for NS in schizophrenia spectrum disorders. In high-quality studies, the clinically meaningful threshold was reached by non-antipsychotic pharmacological agents and lifestyle interventions at middle follow-up. At the subcategory level, antibiotics (minocycline, D-cycloserine), integrated psychosocial interventions (combining CBT, cognitive remediation, motivational interviewing, and family therapy), antidepressants (SSRIs, duloxetine, buspirone, reboxetine), physical activity interventions (physical exercise, dance/movement, and horticultural therapy), transcranial current stimulation (direct current), and immunomodulatory agents (aspirin, celecoxib, fingolimod, methotrexate) achieved clinically meaningful effect sizes. The remaining subcategories showed either statistically significant but subclinical effects or non-significant improvements.

### Clinically meaningful thresholds

The following chapter expands the clinically meaningful findings, listing subcategories from highest to lowest effect sizes.

Antibiotics: minocycline shows anti-inflammatory, antioxidant, and anti-apoptotic effects [51], while D-cycloserine acts on N-methyl D-aspartate (NMDA) receptors [52]. Of note, the largest work on minocycline - the BenMin study - reported no benefit in first-episode schizophrenia/schizoaffective psychosis. However, this study was excluded due to lack of follow-up raw data [53]. Given the low number of studies investigating antibiotics, their low concordance and possible publication bias, findings should be taken carefully.

Integrated psychosocial interventions yielded larger effect sizes than other psychosocial subcategories, and were the only subcategory meeting both thresholds for clinical meaningfulness. However, these interventions were typically delivered over extended periods, combined multiple strategies, and ad hoc designs (e.g., individual vs group formats). These elements, together with possibly enhancing effectiveness, favored heterogeneity at the expense of evidence quality and replicability.

Antidepressants: the effect size we observed exceeded that of a prior meta-analysis, which nevertheless reached clinical significance [54], possibly due to the inclusion of different agents and our focus on high-quality studies. Several core negative symptom domains, particularly anhedonia, anergia, and avolition, overlap substantially with depressive features, complicating both clinical assessment and interpretation of treatment effects, especially when using broad scales such as PANSS, SANS, or BPRS that lack fine discrimination between affective and negative constructs [55]. Moreover, systematic reviews indicate that while some features (e.g., blunted affect and alogia) may be more specific to schizophrenia, others (notably anhedonia and avolition) are shared with depressive syndromes [56], and detailed phenomenological assessment or depression-specific scales may be needed to effectively disentangle these domains [55].

Other interventions: Physical activity interventions produced greater effects than prior exercise-only meta-analyses, which displayed non-clinical effects [30]. In transcranial current stimulation studies, effects emerged despite heterogeneous protocols (e.g., target regions, stimulus intensity). Immunomodulators were among the few subcategories showing non-negligible improvements from baseline scores, further supporting their potential role in regulating neuroinflammation linked to negative/cognitive symptoms [57].

An important consideration when interpreting the present findings is that, across the included studies, non-antipsychotic interventions were almost always used as adjuncts to antipsychotic treatment [58]. Such a design feature may help understand why several subcategories - including immunomodulators, vitamins/nutraceutics, and stimulants - displayed marked improvements even in the control groups, whereas others did not. Although the use of adjunctive interventions reflects routine clinical practice, it limits the ability to disentangle the specific effects of non-antipsychotic treatments from their potential synergistic interactions with antipsychotics. The comparatively lower effectiveness observed for antipsychotics relative to most other intervention categories may, in part, be attributable to this same feature. In addition, the more modest effects observed for antipsychotics may reflect the larger sample sizes required in antipsychotic trials to establish both efficacy and safety, as effect size estimates are known to attenuate with increasing trial size [59].

### Implications for clinical practice

Given the substantial heterogeneity and the predominance of low or very-low GRADE certainty across subcategories, the present findings do not support specific clinical recommendations. Nevertheless, they highlight several considerations that may inform real-world clinical practice, where persistent negative symptoms markedly compromise functioning and even modest improvements may be meaningful.

First, although second-generation antipsychotics produced modest effects, these were relatively stable in a large sample of participants. Often studied as monotherapy, antipsychotics remain crucial for other pivotal domains, including positive symptoms.

Second, no single intervention demonstrated robust and unequivocal efficacy for negative symptoms. Integrated, sustained, and multimodal strategies remain thus promising albeit unproven. Agents such as antidepressants or aspirin represent interesting pharmacological candidates due to their manageable side-effect profiles, while integrated psychosocial approaches, notably involving group-based formats in several trials, may help when core NS such as low motivation and social withdrawal are prevalent.

Other insights apply to non-pharmacological interventions. They avoid medication-related side effects, aiding compliance and evaluation of primary NS. Physical activity is cost-effective and can easily be implemented by non-medical staff. In contrast, psychosocial and brain-stimulation interventions require greater resources in terms of personnel, time, and equipment, limiting scalability. Patient motivation represents a key barrier, particularly when further undermined by avolition, underscoring the need to tailor interventions to both clinical presentation and available resources.

At present, the high heterogeneity observed among subcategories with non-significant effects (antiemetics, stimulants, psychoeducation, glutamatergic agents, other pharmacological agents, art therapy, and anticonvulsants/mood stabilizers) does not support their use in clinical practice. However, the uncertain and contradictory evidence prevents ruling out efficacy too. Antihistamines and antihypertensives showed minimal efficacy and moderate/high certainty; their use is therefore not recommended.

### Implications for future research

The present findings identify several priorities for future research. First and foremost, there is a need for large-scale, long-term, high-quality RCTs specifically targeting NS as a main outcome.

Key challenges such as heterogeneity and limited replicability could be addressed by harmonizing study designs through the use of domain-specific instruments, such as the BNSS or CAINS, alongside systematic reporting of subdomain scores. This approach would also facilitate differentiation between primary NS and overlapping domains such as depressive or extrapyramidal symptoms.

The pattern of effects observed across subcategories supports the need for stratified or mechanism-focused trials. To develop evidence-based precision approaches, clinical trials on interventions such as antiemetics, antibiotics, immunomodulators, or estrogen modulators may benefit from targeted allocation based on hypothesized mechanisms, including sensory gating deficits [60], microglial inflammation [53], elevated C-reactive protein [61] or hormonal alterations [62], respectively.

Focusing on subcategories, antiemetics and stimulants showed impressive improvements but with discouraging magnitudes of heterogeneity. For antiemetics, this likely reflects conflicting results across research teams. Transcranial current stimulation may benefit from standardized protocols involving well-powered studies directly comparing stimulation parameters, such as target regions, frequency, and intensity [63]. Compared with the full set of included trials, analyses restricted to high-quality studies showed that effect sizes for several subcategories either decreased (mindfulness, art therapy and social skills training) or increased (antibiotics and stimulants).

Overall, these findings emphasize the importance of rigorous trial methodology, including preregistration, the use of digital tools [64], transparent data sharing of individual scores, and predefined thresholds for clinical meaningfulness. Adoption of these practices would substantially improve reproducibility and allow more reliable translation of future findings into clinical research and, ultimately, practice.

### Limitations

The main strength (and limitation) of this study is its broad coverage of interventions. Categories and subcategories were organized to balance sample sizes, number of meta-analyses, and heterogeneity, as a detailed characterization of individual interventions was beyond our scope. However, control group improvements varied substantially across subcategories, with the possible presence of publication bias. Furthermore, despite efforts to control for duplicate inclusion, partial sample overlap cannot be fully excluded.

Clozapine and electroconvulsive therapy, the most effective treatments for schizophrenia, could not be meta-analyzed due to the insufficient number of trials versus non-active comparators, as they are typically tested against active treatments (e.g., antipsychotics).

Concerning language, although restrictions to articles written in English may introduce bias according to the PRISMA/Cochrane guidelines, several empirical methodological studies found little or no evidence of a systematic bias from the use of language restrictions in systematic review-based meta-analyses in medicine [35, 65, 66].

Although a sub-analysis comparing PANSS_neg and SANS showed similar effect sizes, domain-specific scales were rarely used, and the present study was not specifically designed to disentangle primary from secondary NS. As shown in our recent metasynthesis [58], standard treatment may differentially influence treatment response across subcategories.

## Conclusions

This meta-analysis provides the most comprehensive comparative synthesis to date of interventions targeting negative symptoms, introducing a data-driven threshold for clinical meaningfulness alongside percentage improvement from baseline to enhance interpretability of effect sizes. Across high-quality studies, only six subcategories reached clinically meaningful significance. The largest effects were observed for antibiotics, integrated psychosocial interventions, and antidepressants, with more modest but promising findings for physical activity, transcranial current stimulation, and immunomodulators. However, substantial heterogeneity and predominantly low certainty of evidence limit the strength of these findings. While selected interventions may yield meaningful improvements, more rigorous designs are needed to identify reliable, personalized and scalable treatment options.

## Supplementary information

Supplementary Materials 1

Supplementary Materials 2 Funnel_Eggers_high_quality_studies

Supplementary Materials 3 - Funnel_Eggers_whole_sample_studies
