# Supplementary material for: Interventions for negative symptoms in schizophrenia: efficacy and clinical interpretability in a meta-analysis of 451 randomized controlled trials
Source: Mol Psychiatry. 2026 Mar 23;31(8):4259–69. doi: 10.1038/s41380-026-03543-1 (PMC13364721; doi:10.1038/s41380-026-03543-1)
Supplement: Supplementary file 1 — Supplementary Materials 1 [file 41380_2026_3543_MOESM1_ESM.docx]

**SUPPLEMENTARY MATERIALS**

**List of Contents**

1. Supplementary Methods

1.1 Search string

1.2 Negative symptom scales

1.3 Strategies for data synthesis

1.4 GRADE downgrading criteria

1.5 Sensitivity Analyses

2. Supplementary Results

2.1 High-quality results

2.2 Whole-sample results

2.2.1 Categories divided by follow-up times

2.2.2 Subcategories

Supplementary Figure 1 (forest plots)

2.2.3 Percentage of improvement from baseline

Supplementary Figure 2 (percentage of improvement)

2.2.4 GRADE: whole sample

2.2.5 Coherence in measurements in PANSS_neg and SANS scores

2.3 Heterogeneity

2.3.1 Main categories: high-quality studies

2.3.2 Subcategories: whole sample

2.3.3 Subcategories: high-quality studies

3. Supplementary Tables: high-quality studies

4. Supplementary Tables: whole-sample studies

5. Supplementary Figures displaying RoB plots

6. List of the included studies

Supplementary Materials - References

References of the included studies

# **1. Supplementary Methods**

## **1.1 Search string**

We systematically searched PsycINFO and all Web of Knowledge databases (Web of Science, BIOSIS, Current Contents Connect, Russian Science Citation Index, Derwent Innovations Index, KCI, MEDLINE, SciELO, Zoological Record, Data Citation Index)

**Supplementary Table 1. Clustering of the literature and search string^a^**

| ***Hypothesized subcategory*** | **Search string** |
| --- | --- |
| *general terms* | (treatment OR therapy OR intervention OR |
| *antipsychotics: first-gen.* | antipsychotic OR chlorpromazine OR flupenthixol OR fluphenazine OR perphenazine OR haloperidol OR pimozide OR zuclopenthixol OR methotrimeprazine OR trifluoperazine OR |
| *antipsychotics: second-gen.* | amisulpride OR asenapine OR blonanserin OR clozapine OR levosulpiride OR lumateperone OR lurasidone OR olanzapine OR paliperidone OR perospirone OR quetiapine OR risperidone OR sertindole OR sonepiprazole OR ziprasidone OR zotepine OR |
| *antipsychotics: third-gen* | aripiprazole OR cariprazine OR brexpiprazole OR lumateperone OR |
| *pharmacological: glutamatergic agents* | glutamat* OR mglu* OR NMDA OR AMPA OR kainate OR ampakine OR glycine OR D-alanine OR D-cycloserine OR D-serine OR LY404039 OR memantine OR (N-acetyl cysteine) OR cystine OR sarcosine OR bitopertine OR |
| *pharmacological: antidepressants* | reboxetine OR citalopram OR fluoxetine OR fluvoxamine OR sertraline OR paroxetine OR buspirone OR trazodone OR maprotiline OR mianserin OR mirtazapine OR ritanserin OR tandospirone OR venlafaxine OR duloxetine OR vortioxetine OR |
| *pharmacological: anticonvulsants* | antiepileptic OR anticonvulsant OR carbamazepine OR lamotrigine OR levetiracetam OR topiramate OR valproate OR (valproic acid) OR |
| *pharmacological: stimulants* | stimulant OR modafinil OR armodafinil OR atomoxetine OR |
| *pharmacological: antiemetics* | antiemetic OR betahistine OR granisetron OR tropisetron OR ondansetron OR palonosetron OR dolansetron OR |
| *pharmacological: antiparkinson* | anti-parkinson OR cerebrolysin OR pramipexole OR selegiline OR latrepirdine OR |
| *pharmacological: anti-cholinesterase* | (cholinesterase inhibitor) OR donepezil OR rivastigmine OR galantamine OR |
| *pharmacological: vitamins/nutraceuticals* | vitamin OR folate OR retinoid OR (fatty acid) OR (omega 3) OR nutraceutical OR (gingko biloba) OR |
| *pharmacological: immunomodulators (non steroidal anti-inflammatory)* | anti-inflammatory OR aspirin OR celecoxib OR fingolimod OR methotrexate OR |
| *pharmacological: hormones* | hormone OR insulin OR oxytocin OR estradiol OR pregnolone OR pregnenolone OR testosterone OR dehydroepiandrosterone OR raloxifene |
| *pharmacological: antibiotics* | antibiotic OR minocycline OR |
| *pharmacological: cannabidiol* | cannabidiol OR |
| *pharmacological: others* | roluperidone OR (sodium nitroprusside) OR antihistamine OR statin |
| *brain stimulation: rTMS* | (Transcranial Magnetic) OR rTMS OR (theta burst stimulation) OR TBS OR |
| *brain stimulation tDCS* | (Transcranial Direct Current) OR tDCS OR (transcranial alternate current) OR tACS OR (transcranial random noise stimulation) OR tRNS OR |
| *brain stimulation: ECT* | (Electro-convulsive) OR ECT OR |
| *psychosocial: general terms* | psychotherapy OR rehab* OR psychosocial OR |
| *psychosocial: cognitive-behavioral* | (cognitive behavioural) OR (cognitive behavioral) OR CBT OR |
| *psychosocial: cognitive remediation* | (computer-assisted cognitive rehabilitation) OR (cognitive remediation) OR metacognit* OR (emotional recognition) OR (affect recognition) OR |
| *psychosocial: mindfulness based* | mindfulness OR meditation OR yoga OR (music therapy) OR |
| *psychosocial: counselling / supportive* | (supportive counselling) OR (supportive analytical therapy) OR (enhanced supportive therapy) OR (key-person counselling) OR befriending OR |
| *psychosocial: psychoeducation / information* | OR psychoeducation OR debriefing OR (psycho-educational medication management training) OR |
| *psychosocial: group* | (group intervention) OR (group therapy) OR (integrated psychosocial therapy) OR (social skills) OR |
| *lifestyle: diet* | diet OR |
| *lifestyle: physical activity* | (dance and movement therapy) OR horticultural OR (physical activity) OR (physical exercise) OR (weight loss) OR (weight reduction) |
| *lifestyle: smoke* | OR (smoking cessation)) |
| *condition and topic* | AND (schizophrenia OR schizoaffective OR schizophreniform OR delusional) AND (negative symptoms) OR apathy OR avolition OR anhedonia OR asociality OR passive social withdrawal OR alogia OR blunted affect OR expressive deficit* OR experiential deficit*) |

^A^ Different colors indicate the five categories (antipsychotics, other pharmacological agents, brain stimulation, psychosocial interventions, life-style interventions), whereas rows identify subcategories. The search string was built on an a priori, hypothetical set of categories/subcategories derived from previous literature. We also included general terms (in violet) to further widen the initial pool of studies. After the screening phase was concluded, we confirmed the five categories and defined a final set of 27 subcategories: 1) antipsychotics: first, second, and third generation; 2) other pharmacological agents: antibiotic, anticholinesterase, anticonvulsant, antidepressant, antiemetic, antihistamine, antihypertensive, immunomodulators, glutamatergic, hormones, statin, stimulant, vitamins-nutraceutic, other pharmacological agents not included in one of the previous subcategories, 3) brain stimulation: transcranial current stimulation, transcranial magnetic stimulation, 4) psychological: art therapy, cognitive and cognitive-behavioral therapy, cognitive-remediation therapy, integrated psychosocial interventions, mindfulness, psychoeducation-support, social skills; 5) lifestyle: physical activity.

## **1.2 Negative symptom scales**

The PANSS encompasses three subscales including one assessing NS (7 items)^1^. The latter assesses the following domains: blunted affect, emotional withdrawal, poor social rapport, passive/apathetic social withdrawal, difficulty in abstract thinking, lack of spontaneity, and stereotyped thinking. We additionally included articles using different scoring methods obtained by recombining different PANSS items, such as the Apathy/Avolition and/or Deficit of Expression (PANSS_NAA/NDE) domains^2^.

The SANS consists of 25 items ranging from 0 to 5 divided into five distinct domains (Affective Flattening or Blunting, Alogia, Avolition-Apathy, Anhedonia-Asociality, Attention^3^. Both a total global score and a summary score can be obtained using this instrument. The total score is the sum of all 25 items, with possible scores ranging from 0 to 125. This total score reflects the overall severity of the individual's NS. In addition to that, the SANS is sometimes summarized by domain scores. Each domain's total score can give an idea of which type of negative symptom is more prominent. The summary scores per domain are calculated by summing the scores for the individual items within each domain.

The BPRS is a scale conceived to measure different symptom manifestations, including anxiety, depression, delusions, thought disorders, and behavioural problems^4^. We only considered the items related to NS, i.e., blunted affect, emotional withdrawal, and motor retardation

The BNSS is a more recent instrument developed to assess specifically NS^5^. It investigates several domains such as emotional expression, flattening of affect, reduction in motivation, decrease in verbal communication, and lack of pleasure in daily activities.

Lastly, the CAINS attempts to measure the impact of NS on social and occupational functioning^6^. It comprises a set of questions designed to evaluate one's capacity to experience pleasure, interest in social interactions, drive, verbal communication, and emotional expression. This instrument lacks an overall score but features two distinct subscales assessing Motivation and Pleasure (9 items) and Expression (4 items).

## **1.3 Strategies for data synthesis**

The primary outcome measures (difference in NS severity from baseline to follow-up between active and non-active treatment groups) was standardized by dividing it by the pooled standard deviations (SD) of the difference scores, or, if unavailable, by the pooled baseline SD for treatment and control groups separately^8^.

Following Cochrane Collaboration guidance^9^, in trials with multiple active arms and one control, the control group size was divided equally across active arms, treating each comparison as a distinct study to prevent sample size inflation, type I error increase, and small effect size overestimation.

Both per-protocol and intention-to-treat designs were included, but when standard follow-up and last-observation-carried-forward (LOCF) data coexisted, standard follow-up was preferred to minimize bias, maintain consistency, and avoid variability underestimation.

In cases where studies reported only adjusted or incomplete data, study authors were contacted to request raw data or clarifying information. A total of 45 authors were contacted. Of these, 19 responded, and 17 ultimately provided usable data: 15 supplied data related to PANSS, one provided information from an alternative NS scale, and one clarified diagnostic composition. Two authors replied but could not provide data, 24 did not respond, and 2 were unreachable due to inactive email addresses. Overall, 17 studies were successfully included in the meta-analysis thanks to author correspondence.

To ensure consistency across studies and enable valid comparisons, several variables reported using different units were harmonized to a common metric:

- Treatment duration was converted to weeks when the original study reported it in days or months.
- Follow-up time was standardized in weeks for all outcome assessments.
- Duration of untreated psychosis (DUP) was uniformly expressed in months.
- Duration of illness was converted to years.
- Age, education, and IQ scores were maintained in the units originally used by the studies, as they were consistently reported in comparable formats (e.g., years or standard test scores).

All harmonized variables were systematically annotated in the dataset to preserve transparency. Where necessary, assumptions used in unit conversions (e.g., average month = 4.33 weeks) were consistently applied.

## **1.4 GRADE downgrading criteria**

The evaluation was made for each treatment subcategory and started from “high” as we considered RCTs. Following the GRADE recommendation *“although it is theoretically possible to rate up results from randomized control trials (RCTs), we have yet to find a compelling example of such an instance”*, no upgrading was applied. Downgrading criteria were:

*Risk of bias (RoB)*. RoB assessment was based on the seven criteria suggested by the Cochrain’s guidelines^9^. Each study was assigned an overall RoB rating. Scores were attributed as follows: “Low” to studies with < 2 unclear and no high RoB items, “Unclear” to studies with 2-3 unclear and no high RoB items, “High” if > 3 unclear or at least 1 high RoB item. As general rule, meta-analytical findings where overall “high” RoB > 25% were downgraded by one point, while those where “high” RoB > 50% were downgraded by two points.

*Inconsistency*. Inconsistency was assessed based on GRADE’s guidelines measuring the heterogeneity between studies within each subcategory^10^. Heterogeneity was computed using Cochrane’s *Q* statistics and I^2^ index^11^. The I^2^ index described the proportion of the total variability in the effect size estimates due to the heterogeneity rather than sampling errors and range, and is considered statistically significant, e.g. moderate heterogeneity, when I^2^ > 50 and substantial heterogeneity when I^2^ > 90^12^. Consequently, we applied no downgrading for I^2^ < 50%, a downgrade of 1 for 50% < I^2^ < 90% and a downgrade of 2 for I^2^ > 90%.

*Indirectness*. Indirectness is addressed by assessing possible mismatches between the study conditions and the research question (eg., the population, intervention or comparator differs from the population/intervention/comparator of interest) which may have affected the generalizability of the results. Due to the design of our meta-analysis and exclusion of studies diverging from the PICOS criteria we defined, indirectness was considered negligible for all of the subcategories.

*Imprecision*. Imprecision was judged based on the effect size CI of each subcategory. Where the CI cut both the 0.472 ES threshold of clinical significance and the 0, a downgrade of 1 was given, while if CI cut both 0.472 and -0.472, a downgrade of 2 was given.

*Publication bias*. Publication bias was evaluated by visual inspection of funnel plots and considering the Egger’s test statistic. Accordingly, asymmetry within the funnel plots combined with a significant intercept (p < .05) from the Egger’s test resulted in downgrading the quality of the evidence.

**1.5 Sensitivity Analyses**

To further explore potential sources of heterogeneity, random-effects meta-regressions were conducted as sensitivity analyses. Meta-regressions examined whether treatment effects on negative symptoms were moderated by mean age, follow-up duration, or baseline severity of negative symptoms.

Given that the primary interpretation of the study focuses on intervention subcategories, all meta-regressions were performed at the subcategory level. Analyses were restricted to high-quality studies and the assumed correlation between baseline and follow-up negative symptoms was r=0.5, consistent with the main analyses. Separate meta-regression models were estimated for each moderator.

Baseline negative symptom severity was included as a moderator only for subcategories in which baseline scores were available on the PANSS negative scale or on the SANS (total or summary), with SANS scores converted to PANSS-equivalent values using established linear transformations. For age and baseline severity, study-level values were computed as the mean of case and control group values when both were available, or as the available group value otherwise. Follow-up duration was operationalized as the maximum follow-up time (in weeks).

Meta-regressions were conducted using random-effects models, which account for both within-study sampling error and between-study heterogeneity. Subcategories contributing fewer than three studies to a given moderator analysis were excluded. All analyses were conducted using the metafor package in R.

# **2. Supplementary Results**

## **2.1 High-quality results**

The number of included arms per follow-up window (k) were: antipsychotic (k short/middle follow-up = 16/8), other pharmacological agents (k short/middle/long follow-up = 33/90/5), brain stimulation (k short/middle follow-up = 35/10), psychosocial (k middle/long follow-up= 32/23), and lifestyle (k middle follow-up = 6). NS improvements were statistically significant in all categories except for antipsychotic_middle, brain_stimulation_middle, and other_pharmacological_long follow-up (Supplementary Tables 3, 4).

## **2.2 Whole-sample results**

### **2.2.1 Categories divided by follow-up times**

Twelve pairwise metanalyses were conducted. These were: antipsychotic (k short/middle/long follow-up = 62/40/10), other pharmacological agents (k short/middle/long follow-up = 63/176/10), brain stimulation (k short/middle follow-up = 42/13), psychosocial (k short/middle/long follow-up= 14/64/37), and lifestyle (k middle follow-up = 17). The improvement was statistically significant in all categories except for brain stimulation, middle follow-up (Supplementary Figure 1A, Supplementary Table 7). The highest effect sizes were observed for lifestyle_middle (SMD = -0.64; CI: -0.85 to -0.43), psychosocial_middle (SMD = 0.61; CI: -0.83 to -0.39) and antipsychotic_long (SMD = -0.57; CI: -0.98 to -0.16). SMD values were consistent even when the assumed correlations between baseline and follow-up values were 0.3 and 0.7, showing a small trend of increase when assuming stronger correlations (0.7 > 0.5 > 0.3, see Supplementary Figure 1A and Supplementary Table 8).

### **2.2.2 Subcategories**

The final set of 27 subcategories included: first (k=11), second (k=92), and third (k=9) generation antipsychotic, antibiotic (k=9), anticholinesterase (k=3), anticonvulsant (k=9), antidepressant (k=38), antiemetic (k=7), antihistamine (k=4), antihypertensive (k=5), immunomodulators (k=9), glutamatergic (k=23), hormones (k=30), statin (k=7), stimulant (k=13), vitamins-nutraceutic (k=45), other pharmacological agents not included in one of the previous subcategories (k=43), transcranial current stimulation (TCS, k=14), transcranial magnetic stimulation (TMS; k=41), art therapy (k=8), cognitive and cognitive-behavioral therapy (cog-CBT, k=24), cognitive-remediation therapy (k=34), integrated psychosocial interventions (k=16), mindfulness (k=17), psychoeducation-support (k=14), social skills (k=15) and physical activity (k=18).

Twenty-three out of 27 subcategories showed a statically significant improvement (Supplementary Figure 1B, Supplementary Table 9). Thirteen studies showed a clinically meaningful improvement. The highest effect size was observed for antiemetics (SMD = -1.01; CI: -1.66 to -0.37), followed by mindfulness (SMD = -0.81; CI: -1.28 to -0.32) and social skills (SMD = -0.81; CI: -1.60 to -0.01). As for the results based on categories/follow-up, subcategories SMD showed a small trend of increase when assuming stronger correlations (0.7 > 0.5 > 0.3).

### **Supplementary Figure 1. Forest plots displaying the mean efficacy of active interventions versus control comparators for negative symptoms in schizophrenia in the whole sample of studies^a^**


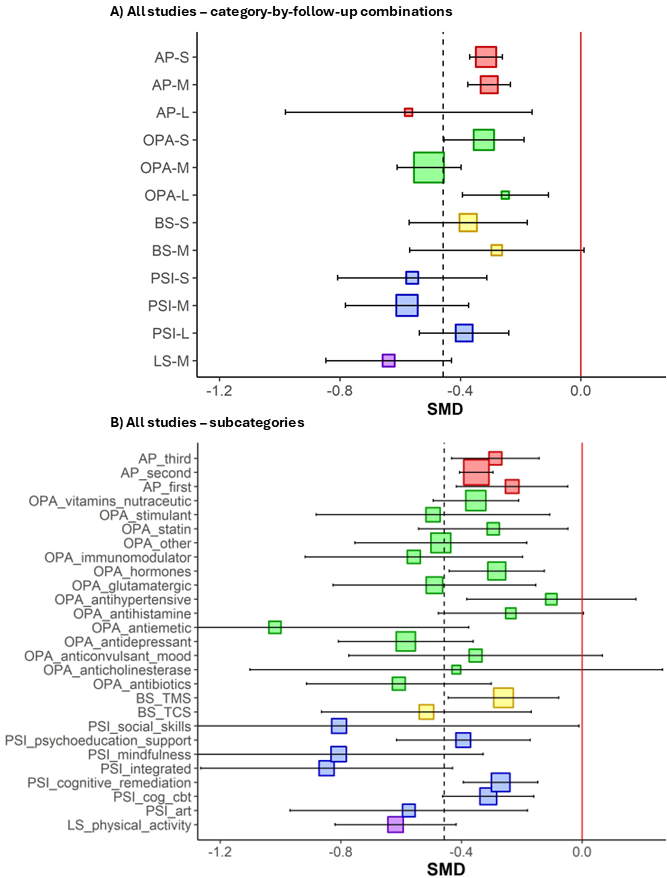


^a^ Effect sizes are reported as standardized mean differences (SMD) for A) the five main categories of interventions stratified by follow-up treatments (12 meta-analyses); B) the subcategories (27 meta-analyses). The red line corresponds to a SMD of 0, while the dashed line is the SMD threshold for a clinical meaningful improvement in negative symptoms. Negative SMD values favor the active intervention.

### **2.2.3 Percentage of improvement from baseline**

This analysis only included studies utilizing PANSS_neg or SANS (converted to PANSS_neg) for which the percentage of improvement from baseline scores could be calculated. The following results were thus obtained from 37602 of the 41903 total participants included. None of the mean percentages reached the 27% threshold for clinical significance. However, the subcategories with the highest percentage of change approached that value: integrated psychosocial interventions (24.59%; CI: 13.23-35.93%), antibiotics (23.77%; CI: 13.23-35.93%), and social skills (23.22%; CI: 5.78-40.66%). Of note, improvements in control groups were highly heterogeneous across the different subcategories (Supplementary Figure 2 and Supplementary Table 10).

**Supplementary Figure 2. Bar plots illustrating the percentage of improvement from baseline for each subcategory of intervention in whole-sample studies^a^**

**
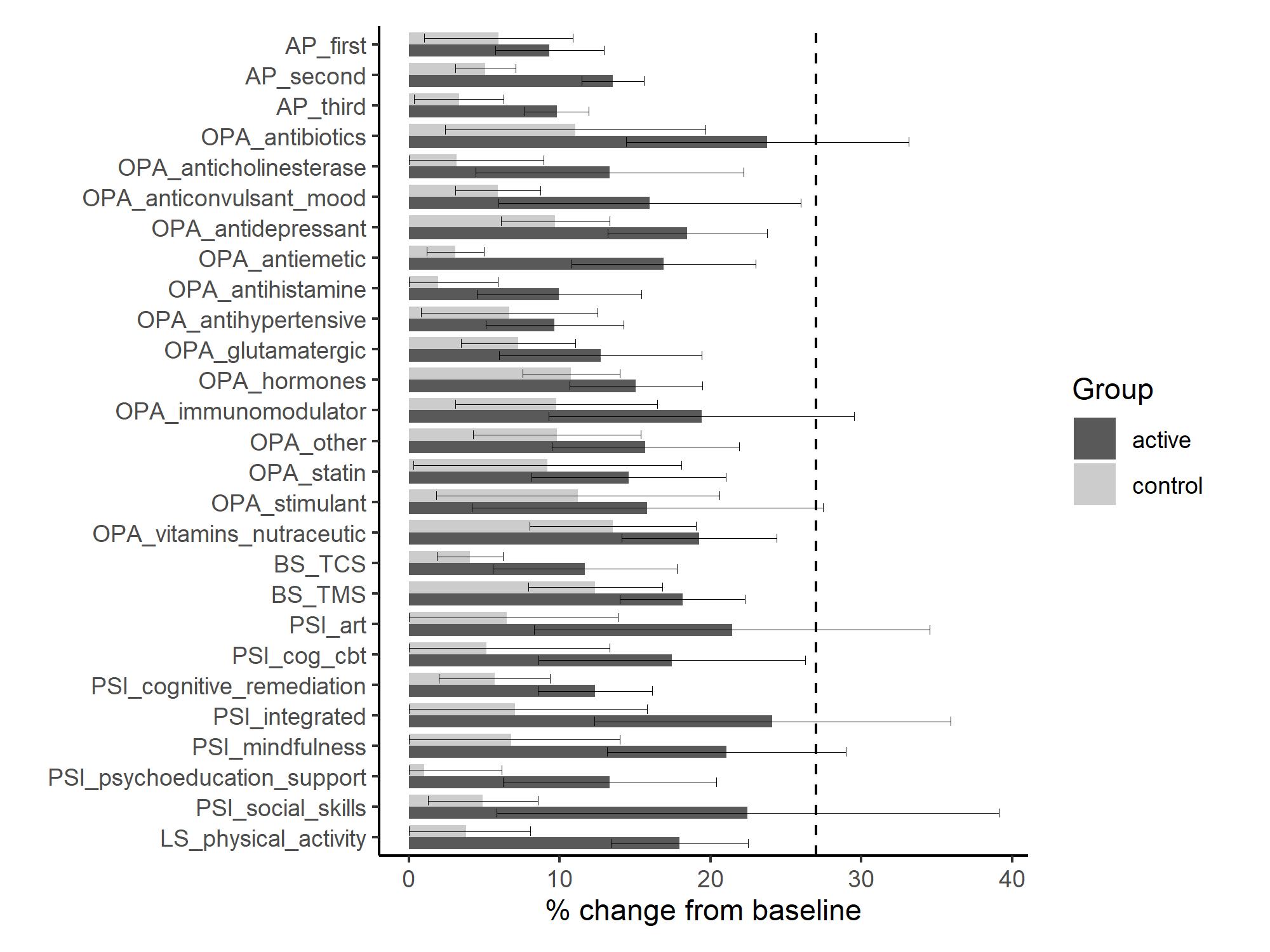
**

^a^ Dark bars correspond to active treatments, while light bars to their respective control treatment groups. Error bars represent 95% confidence intervals.

### **2.2.4 GRADE: whole sample**

Supplementary Table 2 displays the results of the GRADE for each of the 27 subcategories (high: n = 0, moderate = 6, low = 7, very-low = 14).

**Supplementary Table 2. GRADE scores indicating the certainty of evidence for each intervention subcategory (whole-sample studies)^a^**

| **Cat.** | **Subcategory** | **RoB** | **Inconsistency** | **Indirectness** | **Imprecision** | **Publication bias** | **DR gradient** | **GRADE** |
| --- | --- | --- | --- | --- | --- | --- | --- | --- |
| **AP** | AP_first | -2 | - | - | - | - | NA | low |
|  | AP_second | -2 | - | - | - | -1 | NA | very low |
|  | AP_third | -2 | - | - | - | - | NA | low |
| **BS** | BS_TCS | - | -1 | - | - | - | NA | moderate |
|  | BS_TMS | - | -1 | - | - | - | NA | moderate |
| **LS** | LS_physical_activity | -2 | - | - | - | - | NA | low |
| **OPA** | OPA_antibiotics | -2 | -1 | - | - | - | NA | very low |
|  | OPA_anticholinesterase | -1 | -1 | - | -1 | - | NA | very low |
|  | OPA_anticonvulsant_mood | - | -1 | - | -1 | - | NA | low |
|  | OPA_antidepressant | -2 | -1 | - | - | - | NA | very low |
|  | OPA_antiemetic | -1 | -1 | - | - | -1* | NA | very low |
|  | OPA_antihistamine | - | - | - | -1 | - | NA | moderate |
|  | OPA_antihypertensive | -1 | - | - | - | - | NA | moderate |
|  | OPA_immunomodulator | - | -1 | - | - | - | NA | moderate |
|  | OPA_glutamatergic | -2 | -1 | - | - | -1 | NA | very low |
|  | OPA_hormones | -1 | -1 | - | - | - | NA | low |
|  | OPA_other | -1 | -2 | - | - | -1 | NA | very low |
|  | OPA_statin | -1 | - | - | - | - | NA | moderate |
|  | OPA_stimulant | -2 | -1 | - | - | - | NA | very low |
|  | OPA_vitamins_nutraceutic | -1 | -1 | - | - | - | NA | low |
| **PSI** | PSI_art | -2 | -1 | - | - | - | NA | very low |
|  | PSI_cog_cbt | -2 | -1 | - | - | -1 | NA | very low |
|  | PSI_cognitive_remediation | -2 | - | - | - | - | NA | low |
|  | PSI_integrated | -2 | -2 | - | - | - | NA | very low |
|  | PSI_mindfulness | -2 | -2 | - | - | - | NA | very low |
|  | PSI_psychoeducation_support | -2 | -1 | - | - | - | NA | very low |
|  | PSI_social_skills | -2 | -2 | - | - | - | NA | very low |

^a^ Abbreviations: AP = antipsychotic; LF = lifestyle; OPA = other pharma agent; PSI = psychosocial intervention; BS = brain stimulation.

Cat. = category of intervention; RoB = Risk of Bias; DR gradient = dose-response gradient; GRADE = final score indicating the certainty of meta-analytic evidence.

*downgraded as four studies (the only ones detecting significant improvements compared to placebo) were produced by same research group

### **2.2.5 Coherence in measurements in PANSS_neg and SANS scores**

A total of 38 studies (including 46 active intervention arms) used both scales to measure changes in negative symptoms. PANSS_neg and SANS estimated similar SMDs even when assuming different correlations between baseline and follow-up scores (r0.3: SMD PANSS_neg = -0.333, SMD SANS = -0.313, p = 0.726; r0.5: SMD PANSS_neg = -0.385, SMD SANS = -0.372, p = 0.747; r0.7: SMD PANSS_neg = -0.467, SMD SANS = -0.467, p = 0.987) (see Supplementary Table 11).

## **2.3 Heterogeneity**

### **2.3.1 Main categories**

Antipsychotics (short and middle follow-up) and other pharmacological interventions (long) showed the lowest heterogeneity (I^2^ < 0.01). Lifestyle and brain stimulation (middle) showed a relatively low heterogeneity (I^2^ = 43.56 and 43.39 respectively). The remaining categories showed high heterogeneity (I^2^ > 50). The highest heterogeneity was reached by antipsychotic (long) and other pharmacological interventions (middle) (I^2^ = 89.12 and 81.45 respectively).

### **2.3.2 Subcategories: whole sample**

Heterogeneity was at least moderate (I^2^ > 50) in 21 out or 27 subcategories, with higher values in some of the categories with the highest effects in improving negative symptoms (I^2^ antiemetics = 82.45, antibiotics 82.84 and integrated psychotherapies 82.50). The highest value of heterogeneity was found for studies on glutamatergic agents (I^2^ = 92.69). Full results are reported in Supplementary Table 9.

### **2.3.3 Subcategories: high-quality studies**

Heterogeneity was at least moderate (I^2^ > 50) in 14 out of 24 subcategories. The highest values of heterogeneity were observed for studies on glutamatergic agents (I^2^ = 91.50), stimulants (I^2^ = 91.36), and antiemetics (I^2^ = 90.82). Full results are reported in Supplementary Table 4.

Funnel plots and Hegger’s tests results can be found in the Supplementary Material 2 and Supplementary Material 3 files.

# **3. Supplementary Tables: high-quality studies**

**Supplementary Table 3. Effects in category-by-follow-up combinations with different baseline/follow-up correlations assumed for negative symptoms (high-quality studies).**

| **Treatment** | **Follow-up time** | **k** | **n** | | **SMD** | **se** | **z value** | **p value** | **p value (FDR)** | **CI** | | **tau** | **I2** | **H2** | **Q** | **Qp value** |
| --- | --- | --- | --- | --- | --- | --- | --- | --- | --- | --- | --- | --- | --- | --- | --- | --- |
|  |  |  | **active** | **control** |  |  |  |  |  | **lower** | **upper** |  |  |  |  |  |
| estimate r = 0.3 | | | | | | | | | | | | | | | | |
| AP | short | 16 | 2099 | 883 | -0.207 | 0.049 | -4.227 | 0.000 | 0.000 | -0.304 | -0.111 | 0.010 | 28.225 | 1.393 | 18.691 | 0.228 |
| AP | middle | 8 | 530 | 331 | -0.293 | 0.125 | -2.337 | 0.019 | 0.024 | -0.539 | -0.047 | 0.071 | 60.200 | 2.513 | 17.070 | 0.017 |
| LS | middle | 7 | 211 | 192 | -0.614 | 0.127 | -4.842 | 0.000 | 0.000 | -0.862 | -0.365 | 0.033 | 29.623 | 1.421 | 9.059 | 0.170 |
| OPA | short | 33 | 761 | 718 | -0.328 | 0.096 | -3.435 | 0.001 | 0.001 | -0.515 | -0.141 | 0.187 | 65.932 | 2.935 | 90.746 | 0.000 |
| OPA | middle | 89 | 3332 | 2911 | -0.467 | 0.069 | -6.770 | 0.000 | 0.000 | -0.602 | -0.332 | 0.344 | 84.801 | 6.579 | 444.997 | 0.000 |
| OPA | long | 5 | 206 | 176 | -0.124 | 0.104 | -1.199 | 0.231 | 0.231 | -0.327 | 0.079 | 0.000 | 0.000 | 1.000 | 1.706 | 0.790 |
| PSI | short | 4 | 138 | 133 | -0.317 | 0.122 | -2.587 | 0.010 | 0.013 | -0.556 | -0.077 | 0.000 | 0.000 | 1.000 | 0.616 | 0.893 |
| PSI | middle | 38 | 1350 | 1152 | -0.331 | 0.066 | -5.045 | 0.000 | 0.000 | -0.460 | -0.203 | 0.079 | 54.634 | 2.204 | 75.114 | 0.000 |
| PSI | long | 26 | 1411 | 1284 | -0.340 | 0.065 | -5.248 | 0.000 | 0.000 | -0.468 | -0.213 | 0.049 | 55.113 | 2.228 | 55.396 | 0.000 |
| BS | short | 35 | 905 | 788 | -0.347 | 0.088 | -3.944 | 0.000 | 0.000 | -0.520 | -0.175 | 0.167 | 65.410 | 2.891 | 91.916 | 0.000 |
| BS | middle | 11 | 349 | 343 | -0.228 | 0.148 | -1.541 | 0.123 | 0.136 | -0.519 | 0.062 | 0.165 | 70.701 | 3.413 | 33.751 | 0.000 |
| estimate r = 0.5 | | | | | | | | | | | | | | | | |
| AP | short | 16 | 2099 | 883 | -0.210 | 0.049 | -4.277 | 0.000 | 0.000 | -0.306 | -0.114 | 0.011 | 28.399 | 1.397 | 18.746 | 0.225 |
| AP | middle | 8 | 530 | 331 | -0.283 | 0.152 | -1.860 | 0.063 | 0.077 | -0.580 | 0.015 | 0.126 | 72.905 | 3.691 | 20.206 | 0.005 |
| LS | middle | 7 | 211 | 192 | -0.725 | 0.157 | -4.615 | 0.000 | 0.000 | -1.032 | -0.417 | 0.087 | 52.360 | 2.099 | 12.410 | 0.053 |
| OPA | short | 33 | 761 | 718 | -0.364 | 0.105 | -3.452 | 0.001 | 0.001 | -0.571 | -0.157 | 0.250 | 71.991 | 3.570 | 103.266 | 0.000 |
| OPA | middle | 89 | 3332 | 2911 | -0.515 | 0.074 | -6.963 | 0.000 | 0.000 | -0.660 | -0.370 | 0.407 | 86.756 | 7.550 | 492.582 | 0.000 |
| OPA | long | 5 | 206 | 176 | -0.137 | 0.104 | -1.318 | 0.187 | 0.187 | -0.340 | 0.066 | 0.000 | 0.000 | 1.000 | 2.293 | 0.682 |
| PSI | short | 4 | 138 | 133 | -0.371 | 0.123 | -3.023 | 0.003 | 0.003 | -0.611 | -0.130 | 0.000 | 0.000 | 1.000 | 0.801 | 0.849 |
| PSI | middle | 38 | 1350 | 1152 | -0.386 | 0.076 | -5.102 | 0.000 | 0.000 | -0.534 | -0.238 | 0.126 | 65.622 | 2.909 | 98.673 | 0.000 |
| PSI | long | 26 | 1411 | 1284 | -0.398 | 0.075 | -5.290 | 0.000 | 0.000 | -0.546 | -0.251 | 0.082 | 66.826 | 3.014 | 71.413 | 0.000 |
| BS | short | 35 | 905 | 788 | -0.396 | 0.098 | -4.055 | 0.000 | 0.000 | -0.587 | -0.204 | 0.226 | 71.699 | 3.533 | 109.558 | 0.000 |
| BS | middle | 11 | 349 | 343 | -0.260 | 0.159 | -1.634 | 0.102 | 0.112 | -0.573 | 0.052 | 0.201 | 74.546 | 3.929 | 39.403 | 0.000 |
| estimate r = 0.7 | | | | | | | | | | | | | | | | |
| AP | short | 16 | 2099 | 883 | -0.215 | 0.050 | -4.332 | 0.000 | 0.000 | -0.313 | -0.118 | 0.011 | 29.807 | 1.425 | 19.199 | 0.205 |
| AP | middle | 8 | 530 | 331 | -0.266 | 0.206 | -1.291 | 0.197 | 0.197 | -0.669 | 0.138 | 0.276 | 85.367 | 6.834 | 26.780 | 0.000 |
| LS | middle | 7 | 211 | 192 | -0.922 | 0.211 | -4.373 | 0.000 | 0.000 | -1.336 | -0.509 | 0.218 | 72.501 | 3.637 | 19.597 | 0.003 |
| OPA | short | 33 | 761 | 718 | -0.430 | 0.124 | -3.457 | 0.001 | 0.001 | -0.674 | -0.186 | 0.389 | 79.811 | 4.953 | 125.161 | 0.000 |
| OPA | middle | 89 | 3332 | 2911 | -0.599 | 0.084 | -7.173 | 0.000 | 0.000 | -0.763 | -0.435 | 0.536 | 89.515 | 9.537 | 581.862 | 0.000 |
| OPA | long | 5 | 206 | 176 | -0.159 | 0.104 | -1.536 | 0.124 | 0.137 | -0.363 | 0.044 | 0.000 | 0.000 | 1.000 | 3.672 | 0.452 |
| PSI | short | 4 | 138 | 133 | -0.469 | 0.123 | -3.806 | 0.000 | 0.000 | -0.711 | -0.228 | 0.000 | 0.000 | 1.000 | 1.163 | 0.762 |
| PSI | middle | 38 | 1350 | 1152 | -0.485 | 0.095 | -5.132 | 0.000 | 0.000 | -0.670 | -0.300 | 0.237 | 77.922 | 4.529 | 148.579 | 0.000 |
| PSI | long | 26 | 1411 | 1284 | -0.496 | 0.094 | -5.296 | 0.000 | 0.000 | -0.680 | -0.313 | 0.153 | 78.797 | 4.716 | 103.259 | 0.000 |
| BS | short | 35 | 905 | 788 | -0.483 | 0.116 | -4.158 | 0.000 | 0.000 | -0.711 | -0.255 | 0.361 | 79.895 | 4.974 | 146.273 | 0.000 |
| BS | middle | 11 | 349 | 343 | -0.318 | 0.181 | -1.757 | 0.079 | 0.096 | -0.673 | 0.037 | 0.281 | 80.089 | 5.022 | 51.790 | 0.000 |

^a^ Abbreviations: AP = antipsychotic; PSI = psych; LFS = lifestyle; BS = brain stimulation; OPA = pharma. SMD = standard mean difference; CI = confidence interval.

**Supplementary Table 4. Effects in subcategories with different baseline/follow-up correlations assumed for negative symptoms (high-quality studies)^a^**

| **Treatment** | **k** | **n** | | **SMD** | **se** | **z value** | **p value** | **p value (FDR)** | **CI** | | **tau** | **I2** | **H2** | **Q** | **Qp** |
| --- | --- | --- | --- | --- | --- | --- | --- | --- | --- | --- | --- | --- | --- | --- | --- |
|  |  | **cases** | **controls** |  |  |  |  |  | **lower** | **upper** |  |  |  |  |  |
| r = 0,3 | | | | | | | | | | | | | | | |
| AP_second | 25 | 2850 | 1432 | -0.273 | 0.047 | -5.839 | 0.000 | 0.000 | -0.365 | -0.182 | 0.023 | 44.853 | 1.813 | 43.178 | 0.009 |
| LS_physical_activity | 8 | 227 | 207 | -0.577 | 0.119 | -4.845 | 0.000 | 0.000 | -0.810 | -0.343 | 0.029 | 26.476 | 1.360 | 10.195 | 0.178 |
| OPA_antibiotics | 3 | 79 | 79 | -0.816 | 0.317 | -2.574 | 0.010 | 0.017 | -1.437 | -0.195 | 0.200 | 66.703 | 3.003 | 5.496 | 0.064 |
| OPA_anticonvulsant_mood | 6 | 303 | 293 | -0.286 | 0.241 | -1.188 | 0.235 | 0.256 | -0.758 | 0.186 | 0.276 | 85.209 | 6.761 | 23.826 | 0.000 |
| OPA_antidepressant | 14 | 439 | 395 | -0.683 | 0.196 | -3.480 | 0.001 | 0.002 | -1.067 | -0.298 | 0.448 | 85.384 | 6.842 | 70.833 | 0.000 |
| OPA_antiemetic | 4 | 133 | 97 | -0.941 | 0.531 | -1.771 | 0.077 | 0.097 | -1.983 | 0.100 | 1.024 | 91.572 | 11.866 | 32.076 | 0.000 |
| OPA_antihistamine | 3 | 108 | 105 | -0.124 | 0.137 | -0.906 | 0.365 | 0.381 | -0.394 | 0.145 | 0.000 | 0.000 | 1.000 | 0.511 | 0.775 |
| OPA_antihypertensive | 3 | 55 | 72 | -0.141 | 0.181 | -0.776 | 0.438 | 0.438 | -0.496 | 0.215 | 0.000 | 0.000 | 1.000 | 0.205 | 0.903 |
| OPA_glutamatergic | 10 | 398 | 351 | -0.281 | 0.227 | -1.234 | 0.217 | 0.248 | -0.726 | 0.165 | 0.427 | 87.779 | 8.183 | 40.162 | 0.000 |
| OPA_hormones | 19 | 559 | 490 | -0.381 | 0.101 | -3.770 | 0.000 | 0.001 | -0.579 | -0.183 | 0.106 | 57.688 | 2.363 | 41.947 | 0.001 |
| OPA_immunomodulator | 7 | 197 | 182 | -0.413 | 0.105 | -3.937 | 0.000 | 0.000 | -0.619 | -0.208 | 0.000 | 0.000 | 1.000 | 3.182 | 0.786 |
| OPA_other | 23 | 897 | 675 | -0.312 | 0.199 | -1.569 | 0.117 | 0.140 | -0.703 | 0.078 | 0.825 | 92.520 | 13.369 | 172.704 | 0.000 |
| OPA_statin | 3 | 173 | 131 | -0.373 | 0.174 | -2.141 | 0.032 | 0.052 | -0.714 | -0.032 | 0.047 | 52.293 | 2.096 | 4.168 | 0.124 |
| OPA_stimulant | 5 | 132 | 131 | -0.712 | 0.395 | -1.800 | 0.072 | 0.096 | -1.486 | 0.063 | 0.677 | 88.343 | 8.579 | 30.541 | 0.000 |
| OPA_vitamins_nutraceutic | 24 | 768 | 756 | -0.385 | 0.080 | -4.794 | 0.000 | 0.000 | -0.542 | -0.228 | 0.079 | 54.294 | 2.188 | 51.240 | 0.001 |
| PSI_art | 5 | 270 | 218 | -0.291 | 0.148 | -1.972 | 0.049 | 0.069 | -0.580 | -0.002 | 0.059 | 55.352 | 2.240 | 8.894 | 0.064 |
| PSI_cog_cbt | 15 | 656 | 630 | -0.294 | 0.094 | -3.114 | 0.002 | 0.005 | -0.479 | -0.109 | 0.061 | 52.761 | 2.117 | 32.010 | 0.004 |
| PSI_cognitive_remediation | 18 | 515 | 506 | -0.198 | 0.071 | -2.801 | 0.005 | 0.009 | -0.336 | -0.059 | 0.002 | 2.360 | 1.024 | 14.868 | 0.387 |
| PSI_integrated | 7 | 267 | 201 | -0.795 | 0.181 | -4.384 | 0.000 | 0.000 | -1.150 | -0.439 | 0.143 | 66.036 | 2.944 | 19.035 | 0.004 |
| PSI_mindfulness | 8 | 431 | 342 | -0.370 | 0.074 | -5.005 | 0.000 | 0.000 | -0.515 | -0.225 | 0.000 | 0.000 | 1.000 | 4.539 | 0.716 |
| PSI_psychoeducation_support | 8 | 320 | 293 | -0.321 | 0.159 | -2.011 | 0.044 | 0.066 | -0.633 | -0.008 | 0.145 | 72.361 | 3.618 | 24.732 | 0.001 |
| PSI_social_skills | 6 | 410 | 350 | -0.288 | 0.099 | -2.924 | 0.003 | 0.008 | -0.481 | -0.095 | 0.015 | 31.174 | 1.453 | 6.176 | 0.186 |
| BS_TCS | 14 | 318 | 307 | -0.483 | 0.168 | -2.878 | 0.004 | 0.008 | -0.813 | -0.154 | 0.284 | 74.410 | 3.908 | 50.461 | 0.000 |
| BS_TMS | 33 | 1000 | 848 | -0.223 | 0.077 | -2.916 | 0.004 | 0.008 | -0.373 | -0.073 | 0.105 | 58.015 | 2.382 | 74.861 | 0.000 |
| r = 0,5 | | | | | | | | | | | | | | | |
| AP_second | 25 | 2850 | 1432 | -0.278 | 0.047 | -5.925 | 0.000 | 0.000 | -0.371 | -0.186 | 0.023 | 45.266 | 1.827 | 43.899 | 0.008 |
| LS_physical_activity | 8 | 227 | 207 | -0.675 | 0.147 | -4.582 | 0.000 | 0.000 | -0.964 | -0.386 | 0.084 | 50.183 | 2.007 | 13.917 | 0.053 |
| OPA_antibiotics | 3 | 79 | 79 | -0.946 | 0.389 | -2.434 | 0.015 | 0.026 | -1.708 | -0.184 | 0.345 | 76.955 | 4.339 | 7.389 | 0.025 |
| OPA_anticonvulsant_mood | 6 | 303 | 293 | -0.324 | 0.254 | -1.275 | 0.202 | 0.221 | -0.823 | 0.174 | 0.315 | 86.757 | 7.551 | 26.383 | 0.000 |
| OPA_antidepressant | 14 | 439 | 395 | -0.760 | 0.221 | -3.439 | 0.001 | 0.002 | -1.192 | -0.327 | 0.589 | 88.357 | 8.589 | 79.730 | 0.000 |
| OPA_antiemetic | 4 | 133 | 97 | -0.982 | 0.551 | -1.783 | 0.075 | 0.099 | -2.062 | 0.097 | 1.106 | 92.066 | 12.604 | 33.648 | 0.000 |
| OPA_antihistamine | 3 | 108 | 105 | -0.146 | 0.137 | -1.065 | 0.287 | 0.300 | -0.416 | 0.123 | 0.000 | 0.000 | 1.000 | 0.698 | 0.705 |
| OPA_antihypertensive | 3 | 55 | 72 | -0.140 | 0.181 | -0.770 | 0.442 | 0.442 | -0.495 | 0.216 | 0.000 | 0.000 | 1.000 | 0.218 | 0.897 |
| OPA_glutamatergic | 10 | 398 | 351 | -0.357 | 0.272 | -1.311 | 0.190 | 0.217 | -0.891 | 0.177 | 0.645 | 91.495 | 11.757 | 49.360 | 0.000 |
| OPA_hormones | 19 | 559 | 490 | -0.395 | 0.107 | -3.687 | 0.000 | 0.001 | -0.605 | -0.185 | 0.129 | 62.252 | 2.649 | 45.885 | 0.000 |
| OPA_immunomodulator | 7 | 197 | 182 | -0.468 | 0.105 | -4.446 | 0.000 | 0.000 | -0.675 | -0.262 | 0.000 | 0.000 | 1.000 | 4.226 | 0.646 |
| OPA_other | 23 | 897 | 675 | -0.355 | 0.205 | -1.731 | 0.084 | 0.100 | -0.758 | 0.047 | 0.881 | 92.922 | 14.128 | 183.147 | 0.000 |
| OPA_statin | 3 | 173 | 131 | -0.387 | 0.170 | -2.272 | 0.023 | 0.037 | -0.722 | -0.053 | 0.044 | 50.257 | 2.010 | 4.022 | 0.134 |
| OPA_stimulant | 5 | 132 | 131 | -0.804 | 0.465 | -1.731 | 0.083 | 0.100 | -1.715 | 0.106 | 0.971 | 91.364 | 11.580 | 37.945 | 0.000 |
| OPA_vitamins_nutraceutic | 24 | 768 | 756 | -0.421 | 0.083 | -5.059 | 0.000 | 0.000 | -0.585 | -0.258 | 0.090 | 57.350 | 2.345 | 55.696 | 0.000 |
| PSI_art | 5 | 270 | 218 | -0.355 | 0.179 | -1.988 | 0.047 | 0.068 | -0.705 | -0.005 | 0.108 | 69.202 | 3.247 | 12.101 | 0.017 |
| PSI_cog_cbt | 15 | 656 | 630 | -0.346 | 0.106 | -3.271 | 0.001 | 0.003 | -0.554 | -0.139 | 0.091 | 62.413 | 2.661 | 42.145 | 0.000 |
| PSI_cognitive_remediation | 18 | 515 | 506 | -0.235 | 0.085 | -2.762 | 0.006 | 0.011 | -0.401 | -0.068 | 0.029 | 28.332 | 1.395 | 19.469 | 0.148 |
| PSI_integrated | 7 | 267 | 201 | -0.926 | 0.205 | -4.524 | 0.000 | 0.000 | -1.328 | -0.525 | 0.203 | 72.778 | 3.673 | 24.223 | 0.000 |
| PSI_mindfulness | 8 | 431 | 342 | -0.435 | 0.074 | -5.858 | 0.000 | 0.000 | -0.580 | -0.289 | 0.000 | 0.000 | 1.000 | 6.152 | 0.522 |
| PSI_psychoeducation_support | 8 | 320 | 293 | -0.357 | 0.181 | -1.978 | 0.048 | 0.068 | -0.712 | -0.003 | 0.202 | 78.317 | 4.612 | 31.389 | 0.000 |
| PSI_social_skills | 6 | 410 | 350 | -0.332 | 0.117 | -2.832 | 0.005 | 0.009 | -0.562 | -0.102 | 0.033 | 50.012 | 2.000 | 8.058 | 0.089 |
| BS_TCS | 14 | 318 | 307 | -0.516 | 0.178 | -2.909 | 0.004 | 0.008 | -0.864 | -0.168 | 0.328 | 76.954 | 4.339 | 55.504 | 0.000 |
| BS_TMS | 33 | 1000 | 848 | -0.264 | 0.088 | -2.994 | 0.003 | 0.007 | -0.436 | -0.091 | 0.165 | 68.208 | 3.145 | 95.483 | 0.000 |
| r = 0,7 | | | | | | | | | | | | | | | |
| AP_second | 25 | 2850 | 1432 | -0.287 | 0.048 | -6.029 | 0.000 | 0.000 | -0.381 | -0.194 | 0.025 | 46.621 | 1.873 | 46.036 | 0.004 |
| LS_physical_activity | 8 | 227 | 207 | -0.853 | 0.197 | -4.331 | 0.000 | 0.000 | -1.239 | -0.467 | 0.213 | 71.085 | 3.458 | 21.826 | 0.003 |
| OPA_antibiotics | 3 | 79 | 79 | -1.177 | 0.517 | -2.276 | 0.023 | 0.037 | -2.191 | -0.163 | 0.684 | 86.057 | 7.172 | 11.259 | 0.004 |
| OPA_anticonvulsant_mood | 6 | 303 | 293 | -0.395 | 0.282 | -1.399 | 0.162 | 0.177 | -0.948 | 0.158 | 0.401 | 89.241 | 9.295 | 31.747 | 0.000 |
| OPA_antidepressant | 14 | 439 | 395 | -0.891 | 0.265 | -3.370 | 0.001 | 0.002 | -1.410 | -0.373 | 0.880 | 91.742 | 12.110 | 92.936 | 0.000 |
| OPA_antiemetic | 4 | 133 | 97 | -1.037 | 0.577 | -1.798 | 0.072 | 0.091 | -2.167 | 0.093 | 1.220 | 92.654 | 13.612 | 35.458 | 0.000 |
| OPA_antihistamine | 3 | 108 | 105 | -0.186 | 0.138 | -1.352 | 0.176 | 0.184 | -0.456 | 0.084 | 0.000 | 0.000 | 1.000 | 1.103 | 0.576 |
| OPA_antihypertensive | 3 | 55 | 72 | -0.137 | 0.181 | -0.757 | 0.449 | 0.449 | -0.493 | 0.218 | 0.000 | 0.000 | 1.000 | 0.245 | 0.885 |
| OPA_glutamatergic | 10 | 398 | 351 | -0.493 | 0.353 | -1.397 | 0.162 | 0.177 | -1.184 | 0.199 | 1.132 | 94.897 | 19.597 | 65.808 | 0.000 |
| OPA_hormones | 19 | 559 | 490 | -0.417 | 0.119 | -3.492 | 0.000 | 0.002 | -0.651 | -0.183 | 0.179 | 69.488 | 3.277 | 53.828 | 0.000 |
| OPA_immunomodulator | 7 | 197 | 182 | -0.560 | 0.113 | -4.940 | 0.000 | 0.000 | -0.782 | -0.338 | 0.009 | 10.180 | 1.113 | 6.326 | 0.388 |
| OPA_other | 23 | 897 | 675 | -0.430 | 0.218 | -1.977 | 0.048 | 0.068 | -0.857 | -0.004 | 0.998 | 93.635 | 15.711 | 203.983 | 0.000 |
| OPA_statin | 3 | 173 | 131 | -0.415 | 0.169 | -2.464 | 0.014 | 0.024 | -0.746 | -0.085 | 0.042 | 49.033 | 1.962 | 3.933 | 0.140 |
| OPA_stimulant | 5 | 132 | 131 | -0.980 | 0.599 | -1.638 | 0.102 | 0.122 | -2.153 | 0.193 | 1.672 | 94.563 | 18.394 | 51.255 | 0.000 |
| OPA_vitamins_nutraceutic | 24 | 768 | 756 | -0.487 | 0.090 | -5.424 | 0.000 | 0.000 | -0.663 | -0.311 | 0.115 | 63.032 | 2.705 | 65.498 | 0.000 |
| PSI_art | 5 | 270 | 218 | -0.468 | 0.236 | -1.987 | 0.047 | 0.068 | -0.930 | -0.006 | 0.223 | 82.078 | 5.580 | 18.940 | 0.001 |
| PSI_cog_cbt | 15 | 656 | 630 | -0.440 | 0.128 | -3.423 | 0.001 | 0.002 | -0.691 | -0.188 | 0.164 | 74.665 | 3.947 | 63.236 | 0.000 |
| PSI_cognitive_remediation | 18 | 515 | 506 | -0.296 | 0.108 | -2.737 | 0.006 | 0.011 | -0.508 | -0.084 | 0.091 | 54.771 | 2.211 | 29.604 | 0.009 |
| PSI_integrated | 7 | 267 | 201 | -1.153 | 0.247 | -4.668 | 0.000 | 0.000 | -1.637 | -0.669 | 0.328 | 80.434 | 5.111 | 33.979 | 0.000 |
| PSI_mindfulness | 8 | 431 | 342 | -0.549 | 0.079 | -6.965 | 0.000 | 0.000 | -0.704 | -0.395 | 0.003 | 6.659 | 1.071 | 9.556 | 0.215 |
| PSI_psychoeducation_support | 8 | 320 | 293 | -0.425 | 0.221 | -1.923 | 0.055 | 0.073 | -0.858 | 0.008 | 0.330 | 85.254 | 6.782 | 45.846 | 0.000 |
| PSI_social_skills | 6 | 410 | 350 | -0.408 | 0.149 | -2.745 | 0.006 | 0.011 | -0.700 | -0.117 | 0.073 | 68.416 | 3.166 | 11.773 | 0.019 |
| BS_TCS | 14 | 318 | 307 | -0.576 | 0.199 | -2.899 | 0.004 | 0.008 | -0.966 | -0.187 | 0.436 | 81.406 | 5.378 | 66.413 | 0.000 |
| BS_TMS | 33 | 1000 | 848 | -0.340 | 0.109 | -3.116 | 0.002 | 0.004 | -0.554 | -0.126 | 0.297 | 79.152 | 4.797 | 137.947 | 0.000 |

^a^ Abbreviations: AT = active treatment; CI = confidence interval. AP = antipsychotic; PSI = psych; LFS = lifestyle; BS = brain stimulation; OPA = pharma. cog = cognitive; cbt = cognitive behavioral therapy ;TMS = transcranial magnetic stimulation; TCS = transcranial current stimulation. SMD = standard mean difference

**Supplementary Table 5. Percentage of improvement from baseline scores within each subcategory (high-quality studies)^a^**

| **Treatment** | **k** | **Group** | **n** | **PANSS_neg baseline severity** | **Change from baseline** | | | | |
| --- | --- | --- | --- | --- | --- | --- | --- | --- | --- |
|  |  |  |  |  | **mean** | **p value** | **percentage** | **CI upper** | **CI lower** |
| antipsychotic_second | 20 | active treatment | 2289 | 21.811 | 3.176 | 0 | 14.564 | 11.445 | 17.683 |
| antipsychotic_second | 20 | placebo | 1177 | 21.946 | 1.854 | 0 | 8.447 | 4.683 | 12.21 |
| lifestyle_physical_activity | 8 | active treatment | 227 | 19.228 | 3.579 | 0 | 18.613 | 11.215 | 26.012 |
| lifestyle_physical_activity | 8 | placebo | 207 | 18.551 | 0.696 | 0.341 | 3.75 | -3.97 | 11.47 |
| pharma_antibiotics | 3 | active treatment | 79 | 23.618 | 5.126 | 0.035 | 21.706 | 1.551 | 41.861 |
| pharma_antibiotics | 3 | placebo | 79 | 21.844 | 1.851 | 0.343 | 8.476 | -9.037 | 25.989 |
| pharma_anticonvulsant_mood | 6 | active treatment | 303 | 21.868 | 3.68 | 0.003 | 16.83 | 5.858 | 27.802 |
| pharma_anticonvulsant_mood | 6 | placebo | 293 | 21.544 | 1.883 | 0 | 8.741 | 5.713 | 11.769 |
| pharma_antidepressant | 13 | active treatment | 408 | 24.735 | 3.963 | 0 | 16.023 | 7.622 | 24.423 |
| pharma_antidepressant | 13 | placebo | 364 | 24.52 | 1.789 | 0.019 | 7.298 | 1.201 | 13.395 |
| pharma_antiemetic | 4 | active treatment | 133 | 14.832 | 2.264 | 0.001 | 15.261 | 6.139 | 24.383 |
| pharma_antiemetic | 4 | placebo | 97 | 14.95 | 0.578 | 0.003 | 3.864 | 1.329 | 6.399 |
| pharma_antihistamine | 3 | active treatment | 108 | 23.872 | 1.789 | 0.006 | 7.496 | 2.101 | 12.89 |
| pharma_antihistamine | 3 | placebo | 105 | 24.413 | 0.494 | 0.346 | 2.024 | -2.184 | 6.232 |
| pharma_antihypertensive | 3 | active treatment | 55 | 20.579 | 1.255 | 0.115 | 6.098 | -1.476 | 13.673 |
| pharma_antihypertensive | 3 | placebo | 72 | 20.514 | 0.762 | 0.223 | 3.715 | -2.261 | 9.691 |
| pharma_glutamatergic | 9 | active treatment | 388 | 24.307 | 3.381 | 0.013 | 13.911 | 2.97 | 24.851 |
| pharma_glutamatergic | 9 | placebo | 340 | 24.76 | 2.046 | 0.005 | 8.265 | 2.447 | 14.083 |
| pharma_hormones | 19 | active treatment | 559 | 20.841 | 3.429 | 0 | 16.45 | 10.302 | 22.599 |
| pharma_hormones | 19 | placebo | 490 | 20.59 | 2.186 | 0 | 10.618 | 6.831 | 14.404 |
| pharma_immunomodulator | 6 | active treatment | 164 | 24.24 | 6.334 | 0 | 26.132 | 13.879 | 38.385 |
| pharma_immunomodulator | 6 | placebo | 145 | 21.992 | 3.307 | 0 | 15.036 | 6.791 | 23.282 |
| pharma_other | 23 | active treatment | 897 | 24.171 | 3.768 | 0.002 | 15.59 | 5.928 | 25.252 |
| pharma_other | 23 | placebo | 675 | 24.209 | 2.003 | 0.064 | 8.275 | -0.478 | 17.028 |
| pharma_stimulant | 5 | active treatment | 132 | 21.331 | 4.751 | 0.048 | 22.275 | 0.193 | 44.357 |
| pharma_stimulant | 5 | placebo | 131 | 21.308 | 3.554 | 0.059 | 16.679 | -0.616 | 33.974 |
| pharma_vitamins_nutraceutic | 24 | active treatment | 768 | 23.726 | 4.481 | 0 | 18.887 | 11.069 | 26.706 |
| pharma_vitamins_nutraceutic | 24 | placebo | 756 | 23.849 | 2.757 | 0.002 | 11.558 | 4.115 | 19.002 |
| psych_art | 5 | active treatment | 270 | 21.266 | 4.327 | 0.012 | 20.348 | 4.542 | 36.155 |
| psych_art | 5 | placebo | 218 | 20.355 | 1.906 | 0.097 | 9.362 | -1.696 | 20.419 |
| psych_cog_cbt | 15 | active treatment | 656 | 16.096 | 2.191 | 0 | 13.614 | 9.069 | 18.16 |
| psych_cog_cbt | 15 | placebo | 630 | 16.245 | 0.439 | 0.231 | 2.703 | -1.723 | 7.13 |
| psych_cognitive_remediation | 18 | active treatment | 515 | 19.137 | 2.197 | 0 | 11.48 | 7.056 | 15.904 |
| psych_cognitive_remediation | 18 | placebo | 506 | 19.361 | 1.065 | 0.002 | 5.502 | 2.092 | 8.911 |
| psych_integrated | 7 | active treatment | 267 | 27.378 | 7.832 | 0.001 | 28.606 | 12.105 | 45.106 |
| psych_integrated | 7 | placebo | 201 | 27.742 | 1.825 | 0.332 | 6.58 | -6.701 | 19.861 |
| psych_mindfulness | 8 | active treatment | 431 | 20.219 | 2.04 | 0.001 | 10.088 | 4.23 | 15.946 |
| psych_mindfulness | 8 | placebo | 342 | 19.546 | -0.318 | 0.549 | -1.628 | -6.956 | 3.7 |
| psych_psychoeducation_support | 7 | active treatment | 293 | 17.563 | 1.879 | 0.054 | 10.698 | -0.194 | 21.589 |
| psych_psychoeducation_support | 7 | placebo | 264 | 17.161 | 0.292 | 0.653 | 1.7 | -5.705 | 9.104 |
| psych_social_skills | 6 | active treatment | 410 | 18.622 | 3.156 | 0.056 | 16.949 | -0.439 | 34.336 |
| psych_social_skills | 6 | placebo | 350 | 18.069 | 1.478 | 0.07 | 8.182 | -0.659 | 17.022 |
| stimulation_TCS | 14 | active treatment | 318 | 22.751 | 2.66 | 0 | 11.691 | 5.591 | 17.792 |
| stimulation_TCS | 14 | placebo | 307 | 22.836 | 0.926 | 0 | 4.056 | 1.884 | 6.228 |
| stimulation_TMS | 33 | active treatment | 1000 | 23.329 | 4.113 | 0 | 17.63 | 12.764 | 22.495 |
| stimulation_TMS | 33 | placebo | 848 | 23.424 | 2.547 | 0 | 10.871 | 6.197 | 15.546 |

^a^ Abbreviations: AT = AT; CI = confidence interval. AP = AP; PSI = PSI; LFS = LS; BS = brain BS; OPA = OPA. cog = cognitive; cbt = cognitive behavioral therapy ;TMS = transcranial magnetic BS; TCS = transcranial current BS. AT = active treatment.

**Supplementary Table 6. Effect comparisons of PANSS_neg and SANS scores in studies using both scales (high-quality studies)^a^**

| **Variables** | **r** | **SMD** | **se** | **z value** | **p value** | **CI lower** | **CI upper** |
| --- | --- | --- | --- | --- | --- | --- | --- |
| PANSS_neg | 0.3 | -0.354 | 0.073 | -4.839 | 0.000 | -0.498 | -0.211 |
| Diff_SANS | 0.3 | 0.044 | 0.079 | 0.562 | 0.574 | -0.110 | 0.199 |
| PANSS_neg | 0.5 | -0.412 | 0.079 | -5.196 | 0.000 | -0.568 | -0.257 |
| Diff_SANS | 0.5 | 0.039 | 0.056 | 0.698 | 0.485 | -0.071 | 0.149 |
| PANSS_neg | 0.7 | -0.506 | 0.101 | -5.006 | 0.000 | -0.704 | -0.308 |
| Diff_SANS | 0.7 | 0.030 | 0.040 | 0.741 | 0.458 | -0.049 | 0.108 |

^a^Abbreviations: PANSS = Positive and Negative Syndrome Scale; SANS = Scale for the Assessment of Negative Symptoms; BPRS = Brief Psychiatric Rating Scale; BNSS = Brief Negative Symptom Scale. CI =Confidence Interval. SMD = standard mean difference; CI = confidence interval

### **Supplementary Table 7. Results of the meta-regression analyses^a^**

| **subcategory** | **model** | **k** | **estimate** | **se** | **z** | **p** | **ci.lb** | **ci.ub** |
| --- | --- | --- | --- | --- | --- | --- | --- | --- |
| AP_second | age | 22 | 0.003 | 0.007 | 0.377 | 0.706 | -0.011 | 0.017 |
|  | followup weeks | 25 | -0.015 | 0.006 | -2.314 | 0.021 | -0.028 | -0.002 |
|  | baseline NS | 20 | 0.003 | 0.011 | 0.228 | 0.819 | -0.020 | 0.025 |
| BS_TCS | age | 13 | 0.015 | 0.026 | 0.585 | 0.558 | -0.036 | 0.067 |
|  | followup weeks | 14 | 0.046 | 0.052 | 0.886 | 0.375 | -0.056 | 0.148 |
|  | baseline NS | 14 | -0.094 | 0.049 | -1.926 | 0.054 | -0.190 | 0.002 |
| BS_TMS | age | 32 | 0.012 | 0.011 | 1.130 | 0.259 | -0.009 | 0.033 |
|  | followup weeks | 33 | 0.006 | 0.011 | 0.509 | 0.610 | -0.016 | 0.027 |
|  | baseline NS | 33 | 0.013 | 0.016 | 0.777 | 0.437 | -0.019 | 0.045 |
| LS_physical_activity | age | 8 | -0.017 | 0.019 | -0.932 | 0.351 | -0.054 | 0.019 |
|  | followup weeks | 8 | 0.023 | 0.019 | 1.204 | 0.229 | -0.015 | 0.061 |
|  | baseline NS | 8 | 0.008 | 0.050 | 0.151 | 0.880 | -0.091 | 0.106 |
| OPA_antibiotics | age | 3 | 0.009 | 0.079 | 0.111 | 0.912 | -0.145 | 0.163 |
|  | followup weeks | 3 | 0.021 | 0.150 | 0.139 | 0.889 | -0.273 | 0.315 |
|  | baseline NS | 3 | 0.128 | 0.070 | 1.839 | 0.066 | -0.008 | 0.265 |
| OPA_anticonvulsant_mood | age | 6 | -0.024 | 0.101 | -0.233 | 0.815 | -0.222 | 0.175 |
|  | followup weeks | 6 | 0.232 | 0.072 | 3.242 | 0.001 | 0.092 | 0.372 |
|  | baseline NS | 6 | -0.049 | 0.051 | -0.972 | 0.331 | -0.149 | 0.050 |
| OPA_antidepressant | age | 14 | 0.013 | 0.037 | 0.352 | 0.725 | -0.059 | 0.085 |
|  | followup weeks | 14 | 0.038 | 0.036 | 1.059 | 0.289 | -0.033 | 0.109 |
|  | baseline NS | 13 | 0.029 | 0.035 | 0.834 | 0.404 | -0.039 | 0.096 |
| OPA_antiemetic | age | 4 | -0.227 | 0.347 | -0.654 | 0.513 | -0.907 | 0.453 |
|  | followup weeks | 4 | 0.067 | 0.084 | 0.801 | 0.423 | -0.097 | 0.232 |
|  | baseline NS | 4 | 0.350 | 0.061 | 5.741 | 0.000 | 0.230 | 0.469 |
| OPA_antihistamine | age | 3 | -0.068 | 0.082 | -0.831 | 0.406 | -0.230 | 0.093 |
|  | followup weeks | 3 | 0.004 | 0.036 | 0.111 | 0.912 | -0.066 | 0.074 |
|  | baseline NS | 3 | -0.095 | 0.114 | -0.833 | 0.405 | -0.317 | 0.128 |
| OPA_antihypertensive | age | 3 | -0.030 | 0.066 | -0.449 | 0.653 | -0.158 | 0.099 |
|  | followup weeks | 3 | -0.007 | 0.046 | -0.164 | 0.870 | -0.097 | 0.082 |
|  | baseline NS | 3 | 0.045 | 0.402 | 0.113 | 0.910 | -0.742 | 0.832 |
| OPA_glutamatergic | age | 10 | 0.095 | 0.046 | 2.067 | 0.039 | 0.005 | 0.184 |
|  | followup weeks | 10 | 0.017 | 0.053 | 0.322 | 0.748 | -0.087 | 0.121 |
|  | baseline NS | 9 | 0.030 | 0.038 | 0.780 | 0.436 | -0.045 | 0.105 |
| OPA_hormones | age | 19 | -0.004 | 0.012 | -0.378 | 0.705 | -0.027 | 0.018 |
|  | followup weeks | 19 | -0.001 | 0.013 | -0.105 | 0.916 | -0.026 | 0.024 |
|  | baseline NS | 19 | -0.034 | 0.029 | -1.184 | 0.236 | -0.091 | 0.022 |
| OPA_immunomodulator | age | 7 | -0.014 | 0.017 | -0.830 | 0.407 | -0.048 | 0.019 |
|  | followup weeks | 7 | 0.021 | 0.041 | 0.506 | 0.613 | -0.059 | 0.100 |
|  | baseline NS | 6 | -0.013 | 0.019 | -0.668 | 0.504 | -0.050 | 0.024 |
| OPA_other | age | 23 | 0.139 | 0.026 | 5.243 | 0.000 | 0.087 | 0.191 |
|  | followup weeks | 23 | 0.005 | 0.076 | 0.065 | 0.948 | -0.144 | 0.154 |
|  | baseline NS | 23 | 0.003 | 0.054 | 0.047 | 0.962 | -0.103 | 0.108 |
| OPA_statin | followup weeks | 3 | 0.005 | 0.004 | 1.295 | 0.195 | -0.003 | 0.013 |
|  | age | 5 | 0.081 | 0.068 | 1.188 | 0.235 | -0.053 | 0.215 |
|  | baseline NS | 5 | -0.112 | 0.146 | -0.769 | 0.442 | -0.397 | 0.173 |
| OPA_vitamins_nutraceutic | age | 23 | -0.010 | 0.012 | -0.822 | 0.411 | -0.034 | 0.014 |
|  | followup weeks | 24 | 0.029 | 0.010 | 2.826 | 0.005 | 0.009 | 0.049 |
|  | baseline NS | 24 | 0.006 | 0.018 | 0.335 | 0.738 | -0.029 | 0.041 |
| PSI_art | age | 5 | 0.026 | 0.036 | 0.715 | 0.475 | -0.045 | 0.096 |
|  | followup weeks | 5 | 0.045 | 0.027 | 1.700 | 0.089 | -0.007 | 0.098 |
|  | baseline NS | 5 | -0.024 | 0.083 | -0.294 | 0.769 | -0.188 | 0.139 |
| PSI_cog_cbt | age | 15 | -0.005 | 0.020 | -0.235 | 0.814 | -0.044 | 0.034 |
|  | followup weeks | 15 | 0.006 | 0.004 | 1.746 | 0.081 | -0.001 | 0.013 |
|  | baseline NS | 15 | 0.011 | 0.037 | 0.293 | 0.769 | -0.061 | 0.083 |
| PSI_cognitive_remediation | age | 18 | -0.030 | 0.016 | -1.897 | 0.058 | -0.061 | 0.001 |
|  | followup weeks | 18 | 0.002 | 0.004 | 0.487 | 0.626 | -0.006 | 0.010 |
|  | baseline NS | 18 | -0.011 | 0.019 | -0.580 | 0.562 | -0.049 | 0.027 |
| PSI_integrated | age | 6 | -0.026 | 0.011 | -2.344 | 0.019 | -0.049 | -0.004 |
|  | followup weeks | 7 | 0.011 | 0.006 | 1.830 | 0.067 | -0.001 | 0.023 |
|  | baseline NS | 7 | -0.038 | 0.024 | -1.565 | 0.118 | -0.086 | 0.010 |
| PSI_mindfulness | age | 7 | 0.011 | 0.006 | 1.760 | 0.078 | -0.001 | 0.024 |
|  | followup weeks | 8 | -0.001 | 0.002 | -0.333 | 0.739 | -0.005 | 0.004 |
|  | baseline NS | 8 | 0.022 | 0.018 | 1.232 | 0.218 | -0.013 | 0.058 |
| PSI_psychoeducation_support | age | 6 | -0.013 | 0.024 | -0.528 | 0.597 | -0.061 | 0.035 |
|  | followup weeks | 8 | 0.007 | 0.013 | 0.586 | 0.558 | -0.018 | 0.032 |
|  | baseline NS | 7 | 0.026 | 0.078 | 0.333 | 0.739 | -0.127 | 0.179 |
| PSI_social_skills | age | 6 | 0.007 | 0.023 | 0.312 | 0.755 | -0.038 | 0.052 |
|  | followup weeks | 6 | -0.015 | 0.006 | -2.609 | 0.009 | -0.025 | -0.004 |
|  | baseline NS | 6 | 0.021 | 0.029 | 0.733 | 0.463 | -0.035 | 0.077 |

^a^ Meta-regression analyses assessing the association of age, follow-up weeks and baseline negative symptoms severity with Standardized Mean Differences (SMDs) between baseline and follow-up negative symptoms severity in each subcategory.

# **4. Supplementary Tables: whole-sample studies**

**Supplementary Table 8. Effects in category-by-follow-up combinations with different baseline/follow-up correlations assumed for negative symptoms (whole-sample studies)^a^**

| **Treatment** | **Follow-up time** | **k** | **n** | | **SMD** | **se** | **z value** | **p value** | **CI** | | **tau** | **I2** | **H2** | **Q** | **Qp value** |
| --- | --- | --- | --- | --- | --- | --- | --- | --- | --- | --- | --- | --- | --- | --- | --- |
|  |  |  | **active** | **control** |  |  |  |  | **lower** | **upper** |  |  |  |  |  |
| estimate r = 0.3 | | | | | | | | | | | | | | | |
| AP | short | 62 | 5947 | 2217 | -0.307 | 0.028 | -11.112 | 0.000 | -0.362 | -0.253 | 0.006 | 12.336 | 1.141 | 59.279 | 0.539 |
| AP | middle | 10 | 762 | 450 | -0.531 | 0.175 | -3.038 | 0.002 | -0.873 | -0.188 | 0.246 | 84.440 | 6.427 | 43.349 | 0.000 |
| AP | long | 40 | 2799 | 1222 | -0.299 | 0.036 | -8.284 | 0.000 | -0.370 | -0.228 | 0.000 | 0.003 | 1.000 | 40.212 | 0.416 |
| LS | middle | 17 | 423 | 354 | -0.551 | 0.089 | -6.185 | 0.000 | -0.726 | -0.377 | 0.035 | 26.836 | 1.367 | 22.268 | 0.135 |
| OPA | short | 63 | 1447 | 1219 | -0.292 | 0.062 | -4.701 | 0.000 | -0.414 | -0.170 | 0.122 | 54.425 | 2.194 | 135.811 | 0.000 |
| OPA | middle | 174 | 6678 | 5548 | -0.463 | 0.051 | -9.053 | 0.000 | -0.564 | -0.363 | 0.369 | 85.423 | 6.860 | 1046.277 | 0.000 |
| OPA | long | 10 | 402 | 370 | -0.226 | 0.073 | -3.110 | 0.002 | -0.369 | -0.084 | 0.000 | 0.010 | 1.000 | 8.876 | 0.449 |
| PSI | short | 17 | 466 | 409 | -0.478 | 0.110 | -4.331 | 0.000 | -0.694 | -0.261 | 0.094 | 53.459 | 2.149 | 29.981 | 0.008 |
| PSI | middle | 72 | 2362 | 2060 | -0.509 | 0.095 | -5.357 | 0.000 | -0.696 | -0.323 | 0.530 | 88.099 | 8.402 | 420.000 | 0.000 |
| PSI | long | 39 | 2220 | 2095 | -0.330 | 0.064 | -5.122 | 0.000 | -0.456 | -0.204 | 0.098 | 72.208 | 3.598 | 119.689 | 0.000 |
| BS | short | 42 | 1134 | 977 | -0.330 | 0.090 | -3.674 | 0.000 | -0.506 | -0.154 | 0.232 | 73.024 | 3.707 | 131.830 | 0.000 |
| BS | middle | 13 | 394 | 378 | -0.245 | 0.136 | -1.805 | 0.071 | -0.511 | 0.021 | 0.157 | 68.496 | 3.174 | 37.544 | 0.000 |
| estimate r = 0.5 | | | | | | | | | | | | | | | |
| AP | short | 62 | 5947 | 2217 | -0.315 | 0.028 | -11.322 | 0.000 | -0.370 | -0.260 | 0.006 | 13.130 | 1.151 | 60.711 | 0.486 |
| AP | middle | 10 | 762 | 450 | -0.572 | 0.209 | -2.737 | 0.006 | -0.981 | -0.162 | 0.375 | 89.123 | 9.193 | 55.229 | 0.000 |
| AP | long | 40 | 2799 | 1222 | -0.305 | 0.036 | -8.431 | 0.000 | -0.376 | -0.234 | 0.000 | 0.000 | 1.000 | 44.270 | 0.259 |
| LS | middle | 17 | 423 | 354 | -0.638 | 0.106 | -6.003 | 0.000 | -0.847 | -0.430 | 0.088 | 47.280 | 1.897 | 29.944 | 0.018 |
| OPA | short | 63 | 1447 | 1219 | -0.322 | 0.068 | -4.738 | 0.000 | -0.455 | -0.189 | 0.167 | 61.833 | 2.620 | 156.798 | 0.000 |
| OPA | middle | 174 | 6678 | 5548 | -0.504 | 0.054 | -9.321 | 0.000 | -0.610 | -0.398 | 0.420 | 86.911 | 7.640 | 1127.808 | 0.000 |
| OPA | long | 10 | 402 | 370 | -0.251 | 0.073 | -3.440 | 0.001 | -0.394 | -0.108 | 0.000 | 0.001 | 1.000 | 11.634 | 0.235 |
| PSI | short | 17 | 466 | 409 | -0.560 | 0.126 | -4.435 | 0.000 | -0.808 | -0.313 | 0.148 | 64.063 | 2.783 | 39.204 | 0.000 |
| PSI | middle | 72 | 2362 | 2060 | -0.578 | 0.104 | -5.530 | 0.000 | -0.782 | -0.373 | 0.657 | 90.070 | 10.071 | 471.897 | 0.000 |
| PSI | long | 39 | 2220 | 2095 | -0.388 | 0.076 | -5.121 | 0.000 | -0.536 | -0.239 | 0.154 | 80.239 | 5.060 | 140.258 | 0.000 |
| BS | short | 42 | 1134 | 977 | -0.374 | 0.100 | -3.742 | 0.000 | -0.570 | -0.178 | 0.310 | 78.196 | 4.586 | 157.485 | 0.000 |
| BS | middle | 13 | 394 | 378 | -0.279 | 0.148 | -1.891 | 0.059 | -0.569 | 0.010 | 0.200 | 73.291 | 3.744 | 44.611 | 0.000 |
| estimate r = 0.7 | | | | | | | | | | | | | | | |
| AP | short | 62 | 5947 | 2217 | -0.330 | 0.028 | -11.580 | 0.000 | -0.386 | -0.274 | 0.008 | 16.378 | 1.196 | 66.142 | 0.304 |
| AP | middle | 10 | 762 | 450 | -0.647 | 0.274 | -2.366 | 0.018 | -1.183 | -0.111 | 0.684 | 93.622 | 15.678 | 76.294 | 0.000 |
| AP | long | 40 | 2799 | 1222 | -0.314 | 0.036 | -8.670 | 0.000 | -0.385 | -0.243 | 0.000 | 0.011 | 1.000 | 53.384 | 0.062 |
| LFS | middle | 17 | 423 | 354 | -0.797 | 0.138 | -5.771 | 0.000 | -1.068 | -0.526 | 0.212 | 67.756 | 3.101 | 46.557 | 0.000 |
| OPA | short | 63 | 1447 | 1219 | -0.376 | 0.079 | -4.731 | 0.000 | -0.531 | -0.220 | 0.268 | 71.990 | 3.570 | 195.863 | 0.000 |
| OPA | middle | 174 | 6678 | 5548 | -0.576 | 0.060 | -9.605 | 0.000 | -0.694 | -0.458 | 0.534 | 89.289 | 9.337 | 1288.686 | 0.000 |
| OPA | long | 10 | 402 | 370 | -0.315 | 0.091 | -3.474 | 0.001 | -0.492 | -0.137 | 0.025 | 31.632 | 1.463 | 17.388 | 0.043 |
| PSI | short | 17 | 466 | 409 | -0.707 | 0.155 | -4.554 | 0.000 | -1.011 | -0.403 | 0.265 | 75.680 | 4.112 | 56.753 | 0.000 |
| PSI | middle | 72 | 2362 | 2060 | -0.702 | 0.123 | -5.718 | 0.000 | -0.942 | -0.461 | 0.938 | 92.685 | 13.670 | 568.700 | 0.000 |
| PSI | long | 39 | 2220 | 2095 | -0.483 | 0.092 | -5.247 | 0.000 | -0.663 | -0.302 | 0.251 | 86.790 | 7.570 | 182.585 | 0.000 |
| BS | short | 42 | 1134 | 977 | -0.455 | 0.119 | -3.826 | 0.000 | -0.687 | -0.222 | 0.478 | 84.440 | 6.427 | 209.415 | 0.000 |
| BS | middle | 13 | 394 | 378 | -0.343 | 0.171 | -2.002 | 0.045 | -0.678 | -0.007 | 0.296 | 79.895 | 4.974 | 60.085 | 0.000 |

^a^ Abbreviations: AP = antipsychotic; PSI = psych; LFS = lifestyle; BS = brain stimulation; OPA = pharma. SMD = standard mean difference; CI = confidence interval.

**Supplementary Table 9. Effects in subcategories with different baseline/follow-up correlations assumed for negative symptoms (whole-sample studies)^a^**

| **Treatment** | **k** | **n** | | **SMD** | **se** | **z value** | **p value** | **CI** | | **tau** | **I2** | **H2** | **Q** | **Qp** |
| --- | --- | --- | --- | --- | --- | --- | --- | --- | --- | --- | --- | --- | --- | --- |
|  |  | **cases** | **controls** |  |  |  |  | **lower** | **upper** |  |  |  |  |  |
| r = 0,3 | | | | | | | | | | | | | | |
| AP_first | 11 | 713 | 186 | -0.239 | 0.084 | -2.836 | 0.005 | -0.404 | -0.074 | 0.000 | 0.005 | 1.000 | 12.636 | 0.245 |
| AP_second | 92 | 8119 | 3419 | -0.341 | 0.027 | -12.529 | 0.000 | -0.394 | -0.287 | 0.020 | 32.922 | 1.491 | 138.004 | 0.001 |
| AP_third | 9 | 676 | 284 | -0.275 | 0.074 | -3.718 | 0.000 | -0.419 | -0.130 | 0.000 | 0.000 | 1.000 | 4.195 | 0.839 |
| LS_physical_activity | 18 | 439 | 368 | -0.535 | 0.086 | -6.216 | 0.000 | -0.704 | -0.366 | 0.032 | 24.992 | 1.333 | 23.132 | 0.145 |
| OPA_antibiotics | 9 | 311 | 263 | -0.509 | 0.129 | -3.952 | 0.000 | -0.761 | -0.256 | 0.073 | 51.298 | 2.053 | 16.542 | 0.035 |
| OPA_anticholinesterase | 3 | 53 | 44 | -0.352 | 0.294 | -1.196 | 0.232 | -0.929 | 0.225 | 0.126 | 48.384 | 1.937 | 3.883 | 0.143 |
| OPA_anticonvulsant_mood | 9 | 374 | 364 | -0.305 | 0.196 | -1.558 | 0.119 | -0.690 | 0.079 | 0.267 | 82.689 | 5.777 | 39.433 | 0.000 |
| OPA_antidepressant | 38 | 1175 | 1086 | -0.521 | 0.101 | -5.167 | 0.000 | -0.719 | -0.323 | 0.285 | 79.566 | 4.894 | 152.243 | 0.000 |
| OPA_antiemetic | 7 | 178 | 138 | -0.927 | 0.310 | -2.992 | 0.003 | -1.535 | -0.320 | 0.540 | 82.649 | 5.763 | 36.228 | 0.000 |
| OPA_antihistamine | 4 | 137 | 132 | -0.202 | 0.123 | -1.645 | 0.100 | -0.442 | 0.039 | 0.000 | 0.000 | 1.000 | 2.061 | 0.560 |
| OPA_antihypertensive | 5 | 92 | 109 | -0.100 | 0.143 | -0.701 | 0.483 | -0.381 | 0.180 | 0.000 | 0.000 | 1.000 | 0.775 | 0.942 |
| OPA_glutamatergic | 23 | 875 | 714 | -0.443 | 0.158 | -2.806 | 0.005 | -0.752 | -0.133 | 0.479 | 88.076 | 8.387 | 116.751 | 0.000 |
| OPA_hormones | 30 | 846 | 756 | -0.273 | 0.076 | -3.565 | 0.000 | -0.423 | -0.123 | 0.080 | 49.857 | 1.994 | 59.180 | 0.001 |
| OPA_immunomodulator | 9 | 281 | 267 | -0.516 | 0.184 | -2.800 | 0.005 | -0.877 | -0.155 | 0.218 | 74.495 | 3.921 | 37.689 | 0.000 |
| OPA_other | 43 | 1984 | 1303 | -0.443 | 0.143 | -3.099 | 0.002 | -0.723 | -0.163 | 0.790 | 92.547 | 13.417 | 478.548 | 0.000 |
| OPA_statin | 7 | 250 | 190 | -0.274 | 0.122 | -2.249 | 0.025 | -0.513 | -0.035 | 0.028 | 28.261 | 1.394 | 8.697 | 0.191 |
| OPA_stimulant | 13 | 427 | 255 | -0.445 | 0.175 | -2.539 | 0.011 | -0.788 | -0.101 | 0.277 | 74.649 | 3.945 | 41.933 | 0.000 |
| OPA_vitamins_nutraceutic | 45 | 1494 | 1466 | -0.326 | 0.069 | -4.712 | 0.000 | -0.461 | -0.190 | 0.136 | 68.053 | 3.130 | 130.424 | 0.000 |
| PSI_art | 8 | 411 | 362 | -0.489 | 0.172 | -2.841 | 0.004 | -0.826 | -0.152 | 0.186 | 80.123 | 5.031 | 35.661 | 0.000 |
| PSI_cog_cbt | 24 | 1083 | 1050 | -0.258 | 0.067 | -3.834 | 0.000 | -0.390 | -0.126 | 0.045 | 47.829 | 1.917 | 47.075 | 0.002 |
| PSI_cognitive_remediation | 34 | 905 | 790 | -0.225 | 0.054 | -4.199 | 0.000 | -0.331 | -0.120 | 0.000 | 0.000 | 1.000 | 27.364 | 0.499 |
| PSI_integrated | 16 | 831 | 747 | -0.759 | 0.204 | -3.729 | 0.000 | -1.159 | -0.360 | 0.578 | 92.116 | 12.684 | 95.561 | 0.000 |
| PSI_mindfulness | 17 | 699 | 603 | -0.727 | 0.244 | -2.985 | 0.003 | -1.205 | -0.250 | 0.929 | 93.775 | 16.064 | 147.561 | 0.000 |
| PSI_psychoeducation_support | 14 | 464 | 411 | -0.349 | 0.102 | -3.441 | 0.001 | -0.548 | -0.150 | 0.071 | 51.198 | 2.049 | 26.243 | 0.016 |
| PSI_social_skills | 15 | 676 | 620 | -0.691 | 0.346 | -1.999 | 0.046 | -1.368 | -0.013 | 1.480 | 96.658 | 29.918 | 178.214 | 0.000 |
| BS_TCS | 14 | 318 | 307 | -0.483 | 0.168 | -2.878 | 0.004 | -0.813 | -0.154 | 0.284 | 74.410 | 3.908 | 50.461 | 0.000 |
| BS_TMS | 41 | 1194 | 992 | -0.221 | 0.082 | -2.713 | 0.007 | -0.382 | -0.061 | 0.177 | 68.674 | 3.192 | 118.273 | 0.000 |
| r = 0,5 | | | | | | | | | | | | | | |
| AP_first | 11 | 713 | 186 | -0.232 | 0.094 | -2.461 | 0.014 | -0.417 | -0.047 | 0.015 | 15.933 | 1.190 | 15.706 | 0.108 |
| AP_second | 92 | 8119 | 3419 | -0.351 | 0.028 | -12.384 | 0.000 | -0.406 | -0.295 | 0.025 | 37.724 | 1.606 | 153.189 | 0.000 |
| AP_third | 9 | 676 | 284 | -0.287 | 0.074 | -3.889 | 0.000 | -0.432 | -0.143 | 0.000 | 0.000 | 1.000 | 4.566 | 0.803 |
| LS_physical_activity | 18 | 439 | 368 | -0.619 | 0.103 | -6.032 | 0.000 | -0.820 | -0.418 | 0.083 | 45.736 | 1.843 | 31.032 | 0.020 |
| OPA_antibiotics | 9 | 311 | 263 | -0.608 | 0.156 | -3.889 | 0.000 | -0.914 | -0.301 | 0.139 | 66.413 | 2.977 | 21.871 | 0.005 |
| OPA_anticholinesterase | 3 | 53 | 44 | -0.417 | 0.349 | -1.195 | 0.232 | -1.101 | 0.267 | 0.229 | 62.759 | 2.685 | 5.265 | 0.072 |
| OPA_anticonvulsant_mood | 9 | 374 | 364 | -0.353 | 0.215 | -1.646 | 0.100 | -0.774 | 0.067 | 0.334 | 85.580 | 6.935 | 46.598 | 0.000 |
| OPA_antidepressant | 38 | 1175 | 1086 | -0.585 | 0.114 | -5.122 | 0.000 | -0.808 | -0.361 | 0.390 | 84.060 | 6.274 | 181.398 | 0.000 |
| OPA_antiemetic | 7 | 178 | 138 | -1.019 | 0.328 | -3.105 | 0.002 | -1.662 | -0.376 | 0.617 | 84.193 | 6.326 | 40.291 | 0.000 |
| OPA_antihistamine | 4 | 137 | 132 | -0.236 | 0.123 | -1.924 | 0.054 | -0.477 | 0.004 | 0.000 | 0.000 | 1.000 | 2.820 | 0.420 |
| OPA_antihypertensive | 5 | 92 | 109 | -0.102 | 0.143 | -0.714 | 0.475 | -0.383 | 0.178 | 0.000 | 0.000 | 1.000 | 0.930 | 0.920 |
| OPA_glutamatergic | 23 | 875 | 714 | -0.490 | 0.172 | -2.854 | 0.004 | -0.826 | -0.153 | 0.580 | 89.892 | 9.893 | 130.003 | 0.000 |
| OPA_hormones | 30 | 846 | 756 | -0.283 | 0.081 | -3.512 | 0.000 | -0.441 | -0.125 | 0.097 | 54.722 | 2.209 | 65.974 | 0.000 |
| OPA_immunomodulator | 9 | 281 | 267 | -0.558 | 0.184 | -3.034 | 0.002 | -0.919 | -0.198 | 0.217 | 74.284 | 3.889 | 36.705 | 0.000 |
| OPA_other | 43 | 1984 | 1303 | -0.468 | 0.145 | -3.223 | 0.001 | -0.753 | -0.184 | 0.819 | 92.770 | 13.832 | 486.292 | 0.000 |
| OPA_statin | 7 | 250 | 190 | -0.295 | 0.126 | -2.332 | 0.020 | -0.542 | -0.047 | 0.035 | 32.322 | 1.478 | 9.514 | 0.147 |
| OPA_stimulant | 13 | 427 | 255 | -0.494 | 0.198 | -2.501 | 0.012 | -0.882 | -0.107 | 0.382 | 80.060 | 5.015 | 49.533 | 0.000 |
| OPA_vitamins_nutraceutic | 45 | 1494 | 1466 | -0.352 | 0.072 | -4.868 | 0.000 | -0.494 | -0.210 | 0.155 | 70.784 | 3.423 | 141.961 | 0.000 |
| PSI_art | 8 | 411 | 362 | -0.575 | 0.201 | -2.860 | 0.004 | -0.968 | -0.181 | 0.271 | 85.228 | 6.770 | 47.233 | 0.000 |
| PSI_cog_cbt | 24 | 1083 | 1050 | -0.311 | 0.077 | -4.033 | 0.000 | -0.463 | -0.160 | 0.074 | 60.112 | 2.507 | 62.186 | 0.000 |
| PSI_cognitive_remediation | 34 | 905 | 790 | -0.270 | 0.063 | -4.282 | 0.000 | -0.394 | -0.147 | 0.026 | 23.400 | 1.305 | 36.509 | 0.130 |
| PSI_integrated | 16 | 831 | 747 | -0.847 | 0.213 | -3.975 | 0.000 | -1.265 | -0.429 | 0.639 | 92.728 | 13.751 | 108.073 | 0.000 |
| PSI_mindfulness | 17 | 699 | 603 | -0.807 | 0.245 | -3.301 | 0.001 | -1.287 | -0.328 | 0.936 | 93.749 | 15.998 | 149.928 | 0.000 |
| PSI_psychoeducation_support | 14 | 464 | 411 | -0.394 | 0.113 | -3.486 | 0.000 | -0.616 | -0.173 | 0.104 | 60.358 | 2.523 | 33.135 | 0.002 |
| PSI_social_skills | 15 | 676 | 620 | -0.806 | 0.406 | -1.984 | 0.047 | -1.602 | -0.010 | 2.067 | 97.548 | 40.786 | 199.776 | 0.000 |
| BS_TCS | 14 | 318 | 307 | -0.516 | 0.178 | -2.909 | 0.004 | -0.864 | -0.168 | 0.328 | 76.954 | 4.339 | 55.504 | 0.000 |
| BS_TMS | 41 | 1194 | 992 | -0.261 | 0.094 | -2.785 | 0.005 | -0.444 | -0.077 | 0.260 | 76.104 | 4.185 | 148.287 | 0.000 |
| r = 0,7 | | | | | | | | | | | | | | |
| AP_first | 11 | 713 | 186 | -0.214 | 0.132 | -1.623 | 0.105 | -0.472 | 0.044 | 0.098 | 54.607 | 2.203 | 22.155 | 0.014 |
| AP_second | 92 | 8119 | 3419 | -0.369 | 0.031 | -12.051 | 0.000 | -0.429 | -0.309 | 0.036 | 46.088 | 1.855 | 182.319 | 0.000 |
| AP_third | 9 | 676 | 284 | -0.310 | 0.074 | -4.184 | 0.000 | -0.455 | -0.165 | 0.000 | 0.000 | 1.000 | 5.741 | 0.676 |
| LS_physical_activity | 18 | 439 | 368 | -0.771 | 0.133 | -5.807 | 0.000 | -1.031 | -0.511 | 0.204 | 66.645 | 2.998 | 48.046 | 0.000 |
| OPA_antibiotics | 9 | 311 | 263 | -0.781 | 0.206 | -3.796 | 0.000 | -1.184 | -0.378 | 0.293 | 80.192 | 5.049 | 32.387 | 0.000 |
| OPA_anticholinesterase | 3 | 53 | 44 | -0.538 | 0.451 | -1.194 | 0.233 | -1.422 | 0.345 | 0.468 | 76.970 | 4.342 | 8.175 | 0.017 |
| OPA_anticonvulsant_mood | 9 | 374 | 364 | -0.438 | 0.250 | -1.755 | 0.079 | -0.927 | 0.051 | 0.477 | 89.318 | 9.361 | 60.699 | 0.000 |
| OPA_antidepressant | 38 | 1175 | 1086 | -0.697 | 0.139 | -5.024 | 0.000 | -0.969 | -0.425 | 0.620 | 89.142 | 9.209 | 233.761 | 0.000 |
| OPA_antiemetic | 7 | 178 | 138 | -1.175 | 0.363 | -3.232 | 0.001 | -1.887 | -0.462 | 0.780 | 86.617 | 7.472 | 47.999 | 0.000 |
| OPA_antihistamine | 4 | 137 | 132 | -0.328 | 0.157 | -2.088 | 0.037 | -0.636 | -0.020 | 0.034 | 34.599 | 1.529 | 4.461 | 0.216 |
| OPA_antihypertensive | 5 | 92 | 109 | -0.106 | 0.143 | -0.740 | 0.459 | -0.387 | 0.175 | 0.000 | 0.000 | 1.000 | 1.273 | 0.866 |
| OPA_glutamatergic | 23 | 875 | 714 | -0.573 | 0.199 | -2.889 | 0.004 | -0.963 | -0.184 | 0.802 | 92.397 | 13.153 | 154.372 | 0.000 |
| OPA_hormones | 30 | 846 | 756 | -0.299 | 0.089 | -3.375 | 0.001 | -0.472 | -0.125 | 0.134 | 62.378 | 2.658 | 79.563 | 0.000 |
| OPA_immunomodulator | 9 | 281 | 267 | -0.631 | 0.186 | -3.402 | 0.001 | -0.995 | -0.268 | 0.221 | 74.434 | 3.911 | 35.731 | 0.000 |
| OPA_other | 43 | 1984 | 1303 | -0.513 | 0.151 | -3.403 | 0.001 | -0.809 | -0.218 | 0.888 | 93.249 | 14.812 | 504.575 | 0.000 |
| OPA_statin | 7 | 250 | 190 | -0.332 | 0.139 | -2.382 | 0.017 | -0.606 | -0.059 | 0.055 | 42.955 | 1.753 | 11.477 | 0.075 |
| OPA_stimulant | 13 | 427 | 255 | -0.583 | 0.241 | -2.420 | 0.016 | -1.055 | -0.111 | 0.621 | 86.524 | 7.420 | 62.693 | 0.000 |
| OPA_vitamins_nutraceutic | 45 | 1494 | 1466 | -0.400 | 0.079 | -5.074 | 0.000 | -0.554 | -0.245 | 0.197 | 75.328 | 4.053 | 166.353 | 0.000 |
| PSI_art | 8 | 411 | 362 | -0.729 | 0.253 | -2.884 | 0.004 | -1.225 | -0.234 | 0.457 | 90.395 | 10.411 | 70.031 | 0.000 |
| PSI_cog_cbt | 24 | 1083 | 1050 | -0.408 | 0.097 | -4.185 | 0.000 | -0.599 | -0.217 | 0.150 | 75.266 | 4.043 | 93.504 | 0.000 |
| PSI_cognitive_remediation | 34 | 905 | 790 | -0.349 | 0.081 | -4.315 | 0.000 | -0.508 | -0.190 | 0.093 | 51.950 | 2.081 | 56.255 | 0.001 |
| PSI_integrated | 16 | 831 | 747 | -1.002 | 0.232 | -4.320 | 0.000 | -1.456 | -0.547 | 0.768 | 93.721 | 15.925 | 132.579 | 0.000 |
| PSI_mindfulness | 17 | 699 | 603 | -0.951 | 0.249 | -3.827 | 0.000 | -1.438 | -0.464 | 0.966 | 93.798 | 16.123 | 155.594 | 0.000 |
| PSI_psychoeducation_support | 14 | 464 | 411 | -0.476 | 0.135 | -3.517 | 0.000 | -0.741 | -0.211 | 0.179 | 71.964 | 3.567 | 48.279 | 0.000 |
| PSI_social_skills | 15 | 676 | 620 | -1.014 | 0.520 | -1.951 | 0.051 | -2.033 | 0.005 | 3.428 | 98.471 | 65.400 | 231.465 | 0.000 |
| BS_TCS | 14 | 318 | 307 | -0.576 | 0.199 | -2.899 | 0.004 | -0.966 | -0.187 | 0.436 | 81.406 | 5.378 | 66.413 | 0.000 |
| BS_TMS | 41 | 1194 | 992 | -0.333 | 0.114 | -2.915 | 0.004 | -0.557 | -0.109 | 0.432 | 83.848 | 6.191 | 208.983 | 0.000 |

^a^ Abbreviations: AT = active treatment; CI = confidence interval. AP = antipsychotic; PSI = psych; LFS = lifestyle; BS = brain stimulation; OPA = pharma. cog = cognitive; cbt = cognitive behavioral therapy ;TMS = transcranial magnetic stimulation; TCS = transcranial current stimulation. SMD = standard mean difference

**Supplementary Table 10. Percentage of improvement from baseline scores within each subcategory (whole-sample studies)^a^**

| **Treatment** | **k** | **Group** | **n** | **PANSS_neg baseline severity** | **Change from baseline** | | | | |
| --- | --- | --- | --- | --- | --- | --- | --- | --- | --- |
|  |  |  |  |  | **mean** | **p value** | **percentage** | **CI upper** | **CI lower** |
| antipsychotic_first | 9 | active treatment | 575 | 23.476 | 2.191 | 0 | 9.333 | 5.718 | 12.948 |
| antipsychotic_first | 9 | placebo | 152 | 21.847 | 1.3 | 0.018 | 5.951 | 1.009 | 10.894 |
| antipsychotic_second | 70 | active treatment | 6006 | 23.125 | 3.126 | 0 | 13.517 | 11.444 | 15.591 |
| antipsychotic_second | 70 | placebo | 2446 | 22.801 | 1.157 | 0 | 5.074 | 3.077 | 7.072 |
| antipsychotic_third | 9 | active treatment | 676 | 22.314 | 2.187 | 0 | 9.802 | 7.667 | 11.936 |
| antipsychotic_third | 9 | placebo | 284 | 20.095 | 0.669 | 0.028 | 3.328 | 0.362 | 6.295 |
| lifestyle_physical_activity | 18 | active treatment | 439 | 20.55 | 3.684 | 0 | 17.929 | 13.38 | 22.478 |
| lifestyle_physical_activity | 18 | placebo | 368 | 20.753 | 0.788 | 0.079 | 3.796 | -0.441 | 8.034 |
| pharma_antibiotics | 9 | active treatment | 311 | 24.095 | 5.728 | 0 | 23.774 | 14.414 | 33.133 |
| pharma_antibiotics | 9 | placebo | 263 | 23.243 | 2.567 | 0.012 | 11.043 | 2.394 | 19.692 |
| pharma_anticholinesterase | 3 | active treatment | 53 | 16.713 | 2.228 | 0.003 | 13.332 | 4.445 | 22.218 |
| pharma_anticholinesterase | 3 | placebo | 44 | 17.322 | 0.551 | 0.278 | 3.181 | -2.562 | 8.925 |
| pharma_anticonvulsant_mood | 9 | active treatment | 374 | 23.111 | 3.689 | 0.002 | 15.964 | 5.931 | 25.997 |
| pharma_anticonvulsant_mood | 9 | placebo | 364 | 22.934 | 1.352 | 0 | 5.895 | 3.069 | 8.721 |
| pharma_antidepressant | 36 | active treatment | 1124 | 24.773 | 4.574 | 0 | 18.464 | 13.186 | 23.742 |
| pharma_antidepressant | 36 | placebo | 1034 | 24.767 | 2.402 | 0 | 9.699 | 6.102 | 13.296 |
| pharma_antiemetic | 7 | active treatment | 178 | 17.296 | 2.921 | 0 | 16.886 | 10.777 | 22.996 |
| pharma_antiemetic | 7 | placebo | 138 | 17.557 | 0.543 | 0.001 | 3.093 | 1.198 | 4.988 |
| pharma_antihistamine | 4 | active treatment | 137 | 23.434 | 2.333 | 0 | 9.955 | 4.505 | 15.405 |
| pharma_antihistamine | 4 | placebo | 132 | 23.664 | 0.46 | 0.334 | 1.944 | -1.998 | 5.887 |
| pharma_antihypertensive | 5 | active treatment | 92 | 20.827 | 2.015 | 0 | 9.673 | 5.088 | 14.258 |
| pharma_antihypertensive | 5 | placebo | 109 | 21.273 | 1.419 | 0.025 | 6.669 | 0.817 | 12.52 |
| pharma_glutamatergic | 20 | active treatment | 708 | 21.88 | 2.78 | 0 | 12.708 | 6.003 | 19.412 |
| pharma_glutamatergic | 20 | placebo | 650 | 22.389 | 1.624 | 0 | 7.255 | 3.476 | 11.034 |
| pharma_hormones | 30 | active treatment | 846 | 22.047 | 3.32 | 0 | 15.059 | 10.677 | 19.442 |
| pharma_hormones | 30 | placebo | 756 | 21.561 | 2.321 | 0 | 10.766 | 7.541 | 13.991 |
| pharma_immunomodulator | 8 | active treatment | 248 | 26.307 | 5.106 | 0 | 19.411 | 9.291 | 29.531 |
| pharma_immunomodulator | 8 | placebo | 230 | 24.955 | 2.442 | 0.004 | 9.787 | 3.088 | 16.487 |
| pharma_other | 40 | active treatment | 1886 | 24.199 | 3.796 | 0 | 15.687 | 9.466 | 21.908 |
| pharma_other | 40 | placebo | 1226 | 24.174 | 2.376 | 0.001 | 9.828 | 4.267 | 15.388 |
| pharma_statin | 6 | active treatment | 189 | 22.365 | 3.26 | 0 | 14.577 | 8.119 | 21.034 |
| pharma_statin | 6 | placebo | 132 | 21.128 | 1.945 | 0.042 | 9.207 | 0.326 | 18.088 |
| pharma_stimulant | 10 | active treatment | 382 | 20.915 | 3.305 | 0.008 | 15.805 | 4.17 | 27.439 |
| pharma_stimulant | 10 | placebo | 240 | 21.067 | 2.359 | 0.019 | 11.198 | 1.805 | 20.591 |
| pharma_vitamins_nutraceutic | 44 | active treatment | 1477 | 24.232 | 4.668 | 0 | 19.264 | 14.125 | 24.403 |
| pharma_vitamins_nutraceutic | 44 | placebo | 1448 | 24.153 | 3.266 | 0 | 13.522 | 8.024 | 19.021 |
| psych_art | 8 | active treatment | 411 | 19.706 | 4.224 | 0.001 | 21.436 | 8.325 | 34.546 |
| psych_art | 8 | placebo | 362 | 18.873 | 1.222 | 0.086 | 6.476 | -0.92 | 13.872 |
| psych_cog_cbt | 24 | active treatment | 1083 | 16.864 | 2.938 | 0 | 17.424 | 8.581 | 26.267 |
| psych_cog_cbt | 24 | placebo | 1050 | 17.23 | 0.885 | 0.219 | 5.136 | -3.048 | 13.32 |
| psych_cognitive_remediation | 34 | active treatment | 905 | 18.589 | 2.295 | 0 | 12.345 | 8.558 | 16.132 |
| psych_cognitive_remediation | 34 | placebo | 790 | 18.558 | 1.054 | 0.002 | 5.679 | 2.008 | 9.35 |
| psych_integrated | 15 | active treatment | 810 | 22.728 | 5.479 | 0 | 24.105 | 12.308 | 35.902 |
| psych_integrated | 15 | placebo | 727 | 23.161 | 1.63 | 0.116 | 7.037 | -1.728 | 15.802 |
| psych_mindfulness | 17 | active treatment | 699 | 20.94 | 4.411 | 0 | 21.067 | 13.164 | 28.97 |
| psych_mindfulness | 17 | placebo | 603 | 20.316 | 1.378 | 0.066 | 6.782 | -0.438 | 14.002 |
| psych_psychoeducation_support | 12 | active treatment | 395 | 17.832 | 2.374 | 0 | 13.314 | 6.246 | 20.382 |
| psych_psychoeducation_support | 12 | placebo | 353 | 17.043 | 0.177 | 0.692 | 1.036 | -4.098 | 6.171 |
| psych_social_skills | 14 | active treatment | 666 | 21.962 | 4.934 | 0.008 | 22.467 | 5.819 | 39.114 |
| psych_social_skills | 14 | placebo | 610 | 21.277 | 1.046 | 0.008 | 4.914 | 1.257 | 8.571 |
| stimulation_TCS | 14 | active treatment | 318 | 22.751 | 2.66 | 0 | 11.691 | 5.591 | 17.792 |
| stimulation_TCS | 14 | placebo | 307 | 22.836 | 0.926 | 0 | 4.056 | 1.884 | 6.228 |
| stimulation_TMS | 40 | active treatment | 1184 | 22.598 | 4.1 | 0 | 18.144 | 13.997 | 22.291 |
| stimulation_TMS | 40 | placebo | 982 | 22.585 | 2.792 | 0 | 12.362 | 7.919 | 16.805 |

^a^ Abbreviations: AT = AT; CI = confidence interval. AP = AP; PSI = PSI; LFS = LS; BS = brain BS; OPA = OPA. cog = cognitive; cbt = cognitive behavioral therapy ;TMS = transcranial magnetic BS; TCS = transcranial current BS. AT = active treatment.

**Supplementary Table 11. Effect comparisons of PANSS_neg and SANS scores in studies using both scales (whole-sample studies)^a^**

| **Variables** | **r** | **SMD** | **se** | **z value** | **p value** | **CI lower** | **CI upper** |
| --- | --- | --- | --- | --- | --- | --- | --- |
| PANSS_neg | 0.3 | -0.333 | 0.060 | -5.587 | 0.000 | -0.450 | -0.216 |
| Diff_SANS | 0.3 | 0.020 | 0.057 | 0.350 | 0.726 | -0.092 | 0.132 |
| PANSS_neg | 0.5 | -0.385 | 0.063 | -6.063 | 0.000 | -0.509 | -0.260 |
| Diff_SANS | 0.5 | 0.013 | 0.041 | 0.323 | 0.747 | -0.067 | 0.093 |
| PANSS_neg | 0.7 | -0.467 | 0.077 | -6.074 | 0.000 | -0.618 | -0.317 |
| Diff_SANS | 0.7 | 0.000 | 0.029 | 0.016 | 0.987 | -0.056 | 0.057 |

^a^ AbbreviationsPANSS = Positive and Negative Syndrome Scale; SANS = Scale for the Assessment of Negative Symptoms; BPRS = Brief Psychiatric Rating Scale; BNSS = Brief Negative Symptom Scale. CI =Confidence Interval. SMD = standard mean difference; CI = confidence interval

# **4. Supplementary Figures for Risk of Bias (RoB) plots for each of the 27 subcategory^a^**

**
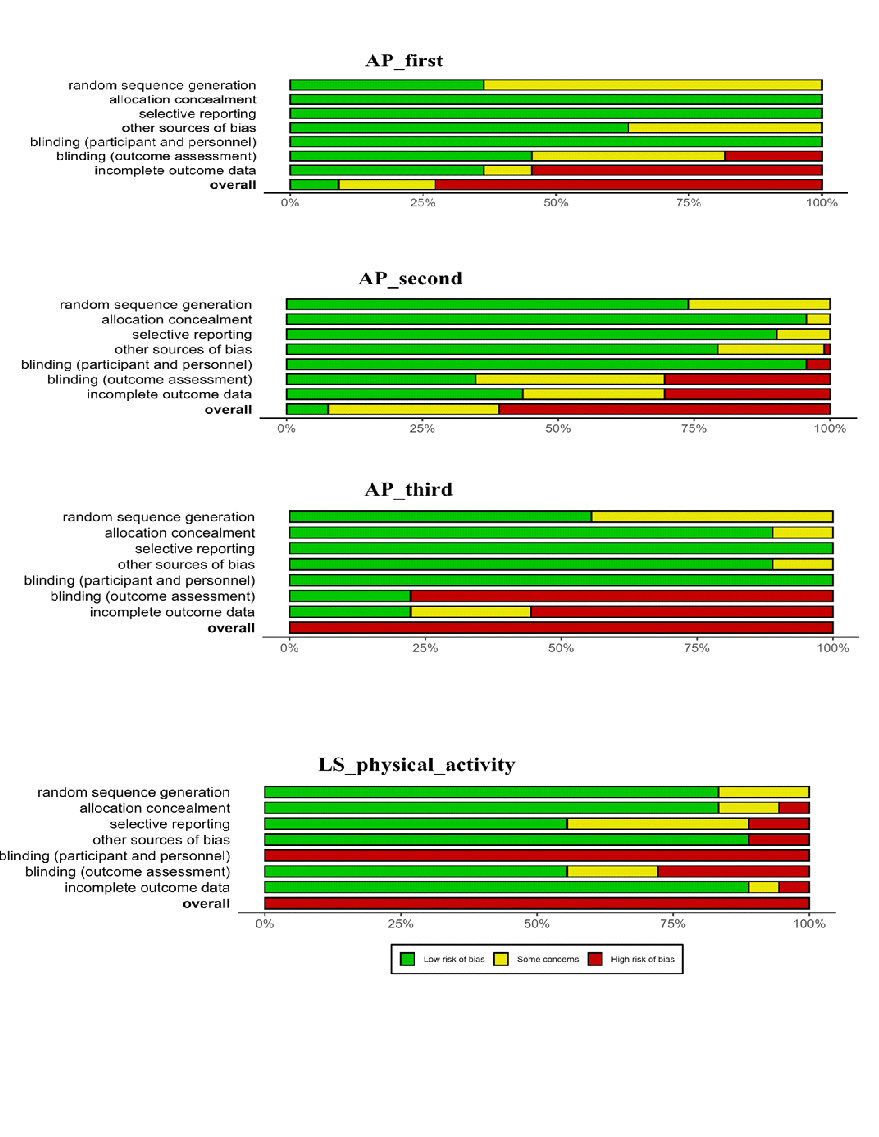
**

# **
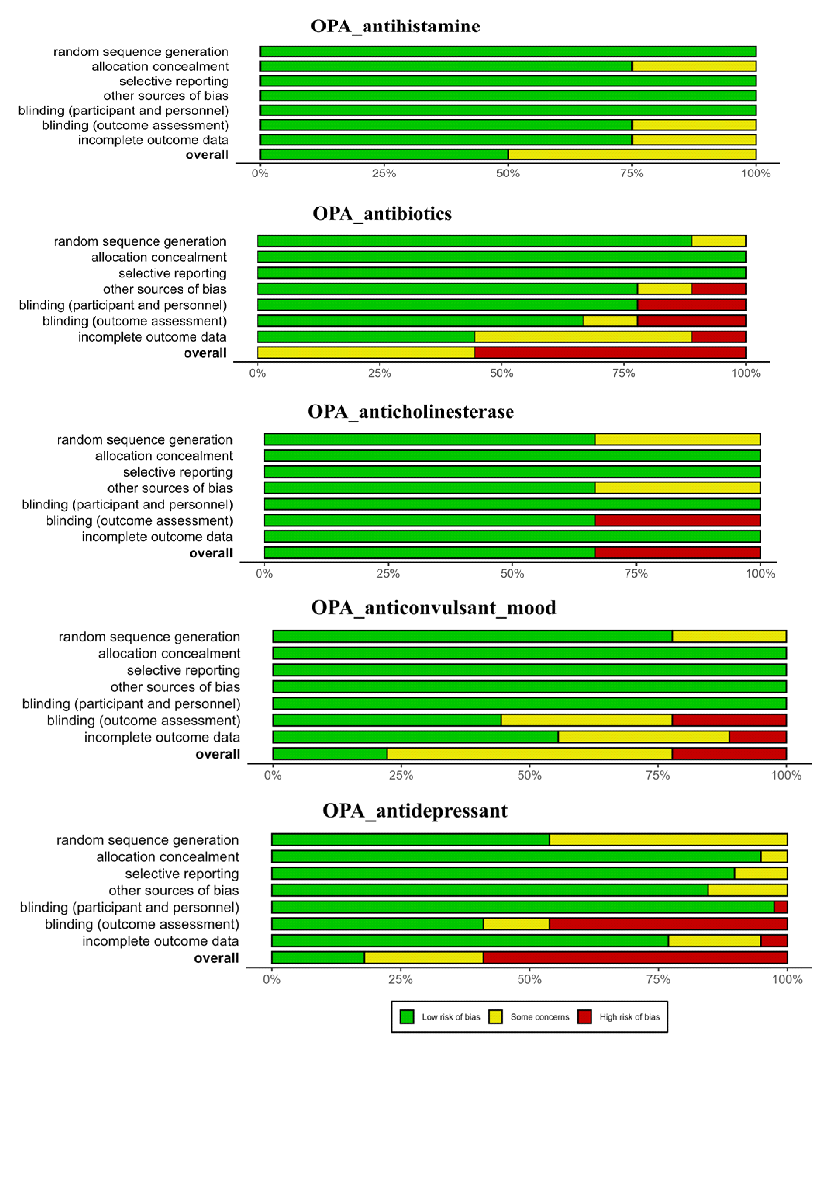

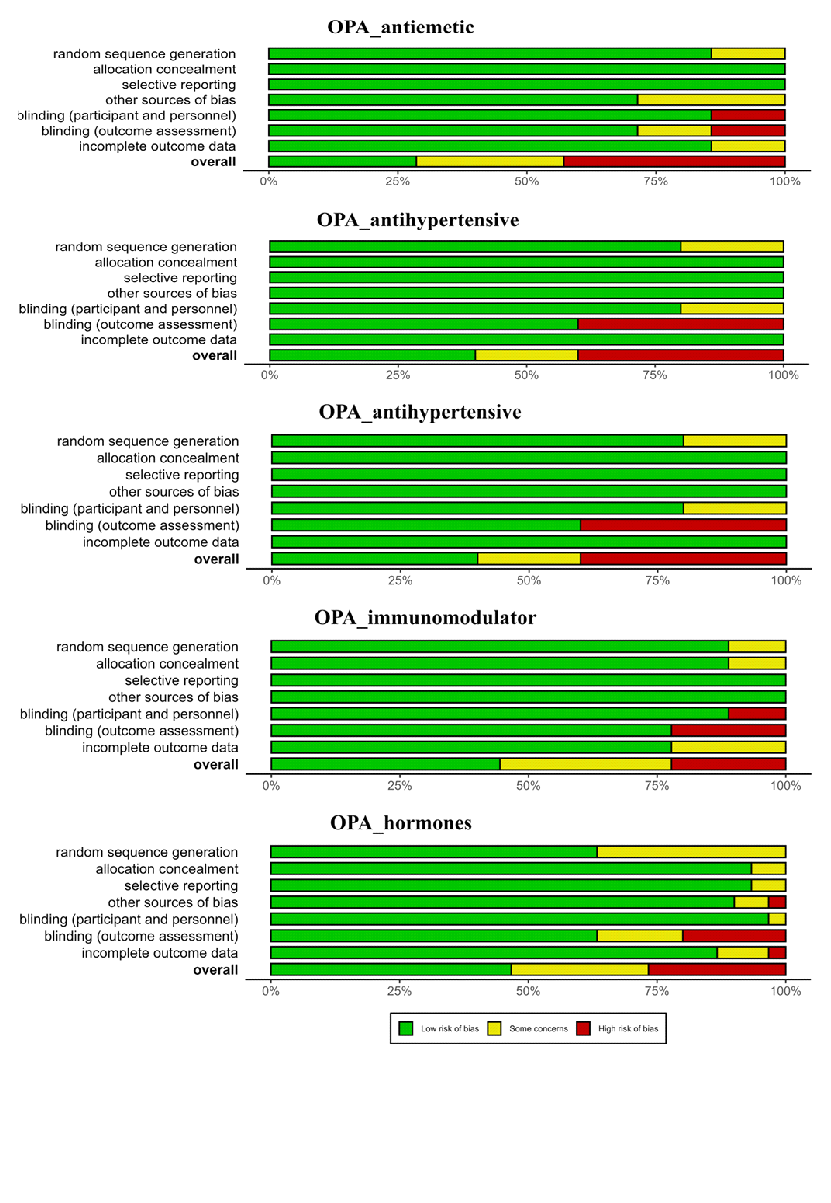
**


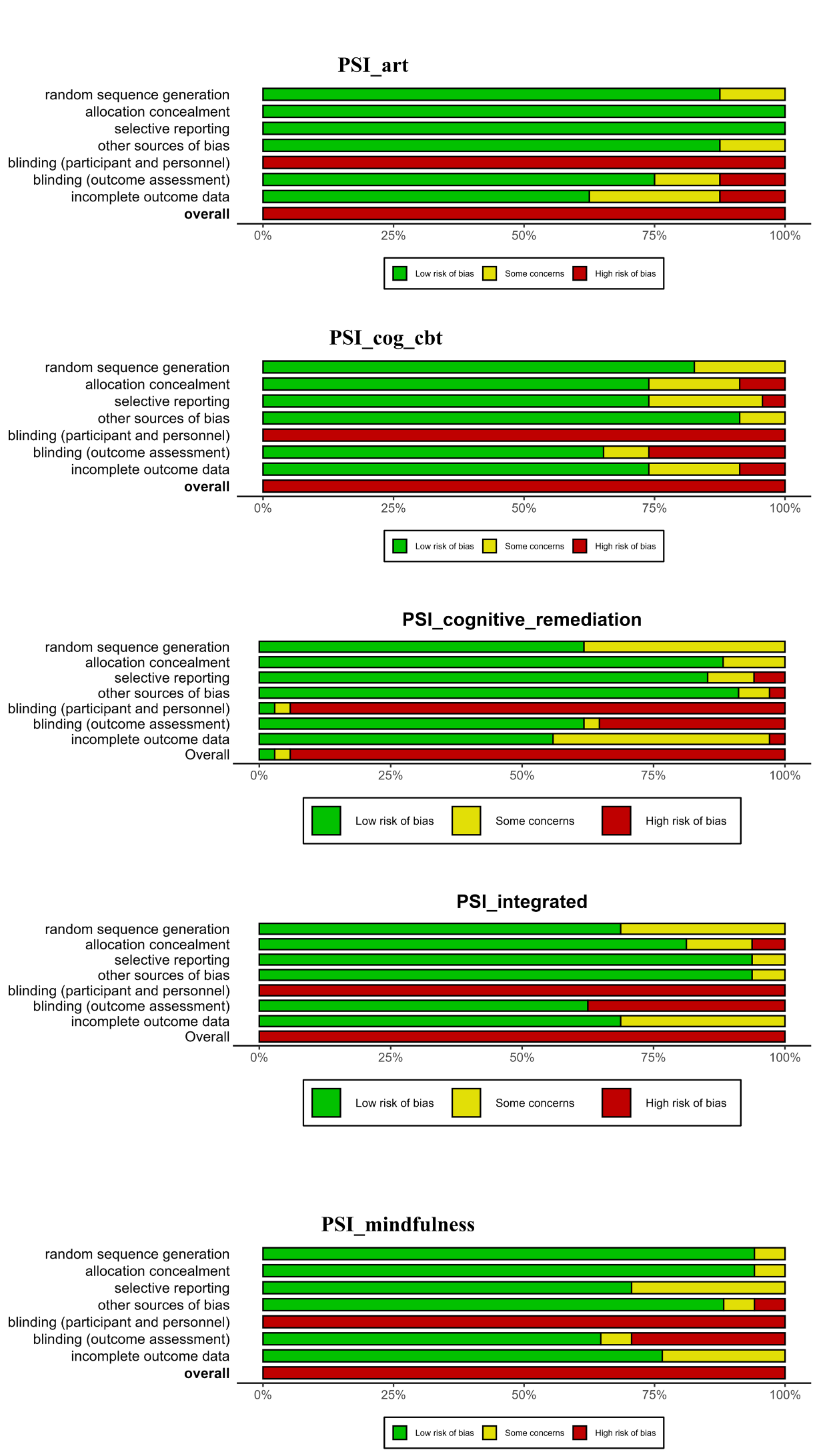


# **
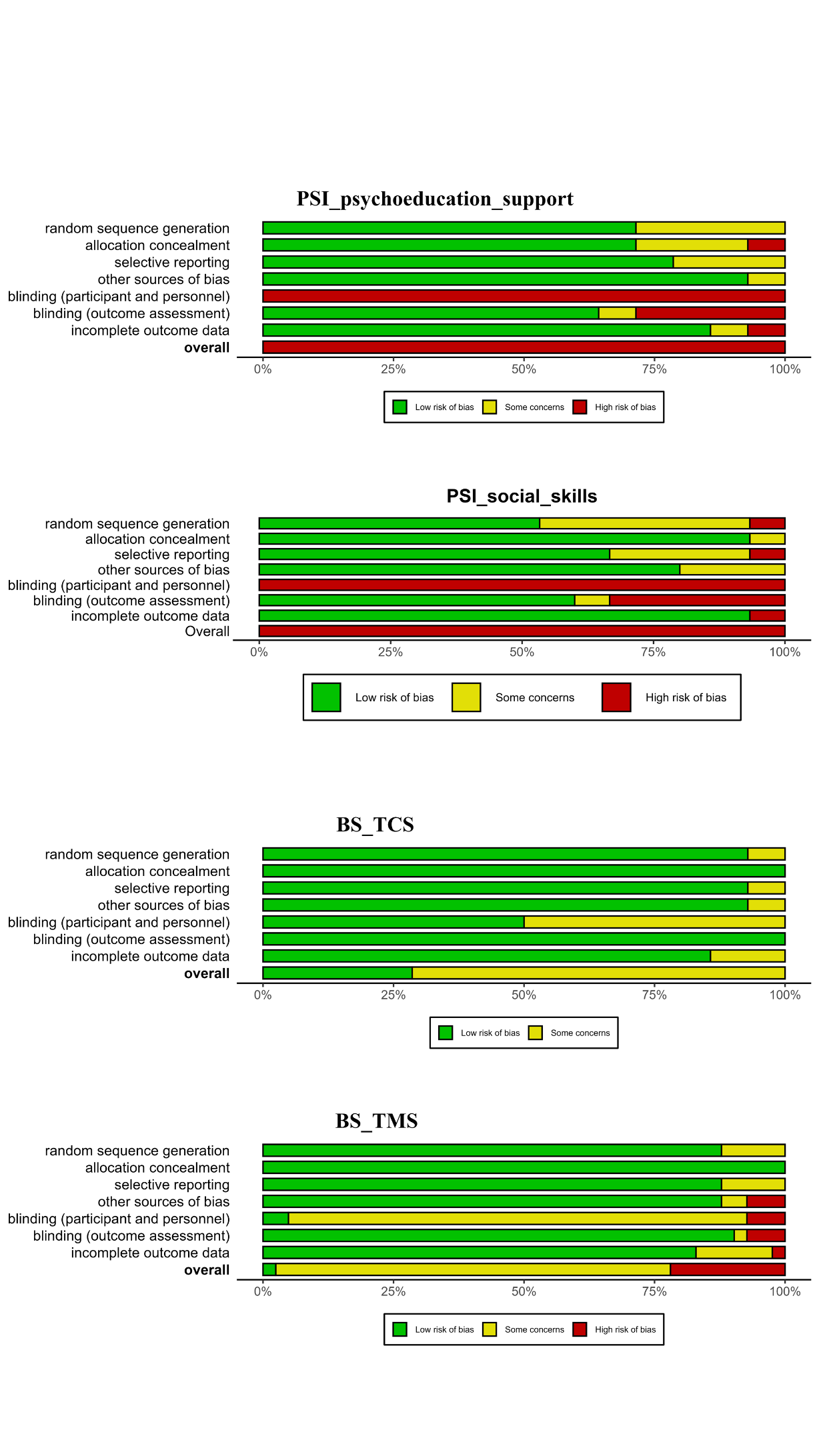
**

^a^ The RoB analysis is displayed for whole-sample studies only as high-quality studies were already assessed as overall “low RoB” studies.

# **5. List of the included studies**

**Supplementary Table 12. Characteristics of the included studies divided by intervention arm^a^**

| **^study name^** | **^n particpants^** | | **^Sample main dx^** | **^Age^** | | **^Male %^** | | **^sample TAU cat^** | **^Control treatment^** | **^Active treatment^** | | **^negative symptom scale^** | **^follow up time (weeks)^** | **^sample dropout (%)^** |
| --- | --- | --- | --- | --- | --- | --- | --- | --- | --- | --- | --- | --- | --- | --- |
|  | **^control^** | **^active^** |  | **^control^** | **^active^** | **^control^** | **^active^** |  |  | **^cat^** | **^name^** |  |  |  |
| **^High quality studies^** | | | | | | | | | | | | | | |
| ^Afshar 2009^ | ^16^ | ^16^ | ^SCZ^ | ^38,10^ | ^37,50^ | ^68,00^ | ^56,00^ | ^SA^ | ^placebo^ | ^OPA^ | ^other anticonvulsant^ | ^PANSS^ | ^8,0^ | ^11,11^ |
| ^Ahmed 2015^ | ^18^ | ^17^ | ^SCZ^ | ^40,64^ | ^40,38^ | ^89,00^ | ^86,00^ | ^mixed^ | ^placebo^ | ^PSI^ | ^computer assisted cognitive rehabilitation^ | ^PANSS^ | ^20,0^ | ^31,43^ |
| ^Akhondzadeh 2011^ | ^20^ | ^20^ | ^SCZ^ | ^32,60^ | ^34,12^ | ^90,00^ | ^90,00^ | ^SA^ | ^placebo^ | ^OPA^ | ^other pharma^ | ^PANSS^ | ^8,0^ | ^40,32^ |
| ^Arbabi 2012^ | ^22^ | ^20^ | ^SCZ^ | ^34,08^ | ^33,52^ | ^85,00^ | ^82,00^ | ^SA^ | ^placebo^ | ^OPA^ | ^armodafinil modafinil^ | ^PANSS^ | ^8,0^ | ^8,70^ |
| ^Attari 2017^ | ^20^ | ^10^ | ^SCZ^ | ^31,35^ | ^33,50^ | ^65,00^ | ^60,00^ | ^AA^ | ^none^ | ^OPA^ | ^aspirin^ | ^PANSS^ | ^6,0^ | ^0,00^ |
| ^Attari 2017^ | ^20^ | ^10^ | ^SCZ^ | ^31,35^ | ^33,45^ | ^65,00^ | ^70,00^ | ^AA^ | ^none^ | ^OPA^ | ^aspirin^ | ^PANSS^ | ^6,0^ | ^0,00^ |
| ^Bai 2023^ | ^47^ | ^47^ | ^SCZ^ | ^46,28^ | ^46,49^ | ^62,00^ | ^60,00^ | ^AA^ | ^placebo^ | ^OPA^ | ^pharma antihistamine^ | ^PANSS^ | ^4,0^ | ^5,32^ |
| ^Bais 2014^ | ^17^ | ^8^ | ^SCZ^ | ^37,30^ | ^33,90^ | ^62,00^ | ^53,00^ | ^AA^ | ^placebo^ | ^BS^ | ^TMS repetitive^ | ^PANSS^ | ^1,0^ | ^21,56^ |
| ^Bais 2014^ | ^18^ | ^8^ | ^SCZ^ | ^37,30^ | ^37,20^ | ^62,00^ | ^56,00^ | ^AA^ | ^placebo^ | ^BS^ | ^TMS repetitive^ | ^PANSS^ | ^1,0^ | ^21,56^ |
| ^Banazadeh 2022^ | ^27^ | ^27^ | ^SCZ^ | ^45,36^ | ^44,65^ | ^76,00^ | ^74,00^ | ^mixed^ | ^placebo^ | ^OPA^ | ^other nutraceutical^ | ^PANSS^ | ^4,0^ | ^11,11^ |
| ^Barnes 2017^ | ^35^ | ^33^ | ^SCZ^ | ^40,00^ | ^39,00^ | ^70,00^ | ^69,00^ | ^SA^ | ^placebo^ | ^AP^ | ^amisulpride^ | ^PANSS^ | ^12,0^ | ^22,05^ |
| ^Barr 2012^ | ^13^ | ^12^ | ^SCZ^ | ^47,92^ | ^44,46^ | ^1,00^ | ^1,00^ | ^AA^ | ^placebo^ | ^BS^ | ^TMS repetitive^ | ^PANSS^ | ^4,0^ | ^24,00^ |
| ^Barrowclough 2001^ | ^18^ | ^18^ | ^SCZ^ | ^31,10^ | ^31,10^ | ^92,00^ | ^92,00^ | ^ns^ | ^none^ | ^PSI^ | ^other PSI^ | ^PANSS^ | ^52,0^ | ^11,11^ |
| ^Barrowclough 2006^ | ^57^ | ^56^ | ^SCZ^ | ^38,83^ | ^38,83^ | ^73,00^ | ^73,00^ | ^AA^ | ^none^ | ^PSI^ | ^CBT cognitive behavioral therapy^ | ^PANSS^ | ^48,0^ | ^13,27^ |
| ^Basavaraju 2021^ | ^30^ | ^30^ | ^SCZ^ | ^NA^ | ^NA^ | ^ns^ | ^ns^ | ^mixed^ | ^placebo^ | ^BS^ | ^TMS theta burst^ | ^SANS^ | ^6,0^ | ^0,00^ |
| ^Behdani 2022^ | ^20^ | ^20^ | ^SCZ^ | ^47,30^ | ^45,20^ | ^68,00^ | ^68,00^ | ^AA^ | ^placebo^ | ^OPA^ | ^other anticonvulsant^ | ^PANSS^ | ^8,0^ | ^0,00^ |
| ^Bellucci 2003^ | ^17^ | ^17^ | ^schizoaffective^ | ^42,00^ | ^42,00^ | ^47,00^ | ^47,00^ | ^mixed^ | ^WL^ | ^PSI^ | ^computer assisted cognitive rehabilitation^ | ^SANS^ | ^8,0^ | ^0,00^ |
| ^Berk 2008^ | ^69^ | ^71^ | ^SCZ^ | ^36,10^ | ^37,20^ | ^70,00^ | ^70,00^ | ^AA^ | ^placebo^ | ^OPA^ | ^N-acetyl cysteine^ | ^PANSS^ | ^24,0^ | ^40,00^ |
| ^Bradley 2006^ | ^25^ | ^25^ | ^SCZ^ | ^34,00^ | ^33,60^ | ^32,00^ | ^28,00^ | ^mixed^ | ^none^ | ^PSI^ | ^psychoeducational medication management training^ | ^SANS^ | ^56,0^ | ^15,25^ |
| ^Brown 2019^ | ^18^ | ^34^ | ^SCZ^ | ^42,83^ | ^47,10^ | ^76,00^ | ^78,00^ | ^AA^ | ^placebo^ | ^OPA^ | ^intravenous sodium nitroprusside^ | ^PANSS^ | ^4,0^ | ^4,00^ |
| ^Brunelin 2012^ | ^15^ | ^15^ | ^SCZ^ | ^35,10^ | ^40,40^ | ^ns^ | ^ns^ | ^AA^ | ^placebo^ | ^BS^ | ^TCS direct^ | ^PANSS^ | ^12,0^ | ^0,00^ |
| ^Brunstein 2005^ | ^12^ | ^11^ | ^SCZ^ | ^42,30^ | ^35,30^ | ^64,00^ | ^58,00^ | ^mixed^ | ^placebo^ | ^OPA^ | ^other pharma^ | ^PANSS^ | ^6,0^ | ^13,00^ |
| ^Buchanan 1996^ | ^18^ | ^15^ | ^SCZ^ | ^32,80^ | ^36,80^ | ^53,00^ | ^83,00^ | ^SA^ | ^placebo^ | ^OPA^ | ^fluoxetine^ | ^SANS^ | ^8,0^ | ^0,00^ |
| ^Buchanan 2007^ | ^56^ | ^28^ | ^SCZ^ | ^43,40^ | ^44,40^ | ^ns^ | ^ns^ | ^mixed^ | ^placebo^ | ^OPA^ | ^D-cycloserine^ | ^SANS^ | ^16,0^ | ^19,39^ |
| ^Buchanan 2007^ | ^54^ | ^28^ | ^SCZ^ | ^43,40^ | ^42,60^ | ^ns^ | ^ns^ | ^mixed^ | ^placebo^ | ^OPA^ | ^other glutamatergic^ | ^SANS^ | ^16,0^ | ^19,39^ |
| ^Buchanan 2015a^ | ^17^ | ^11^ | ^SCZ^ | ^42,20^ | ^47,40^ | ^75,00^ | ^88,00^ | ^mixed^ | ^placebo^ | ^OPA^ | ^oxytocin^ | ^SANS^ | ^4,0^ | ^10,34^ |
| ^Buchanan 2015a^ | ^20^ | ^11^ | ^SCZ^ | ^42,20^ | ^45,80^ | ^75,00^ | ^70,00^ | ^mixed^ | ^placebo^ | ^OPA^ | ^anticholinesterase^ | ^SANS^ | ^4,0^ | ^10,34^ |
| ^Bugarski-Kirola 2022^ | ^172^ | ^174^ | ^SCZ^ | ^36,70^ | ^37,70^ | ^68,00^ | ^65,00^ | ^AA^ | ^placebo^ | ^AP^ | ^other antipsychotic^ | ^PANSS^ | ^26,0^ | ^14,14^ |
| ^Carpenter 2000^ | ^39^ | ^39^ | ^SCZ^ | ^40,00^ | ^40,00^ | ^75,00^ | ^75,00^ | ^AA^ | ^placebo^ | ^OPA^ | ^other pharma^ | ^SANS^ | ^8,0^ | ^0,00^ |
| ^Chaudhry 2020^ | ^45^ | ^47^ | ^SCZ^ | ^26,60^ | ^24,80^ | ^68,00^ | ^78,00^ | ^AA^ | ^placebo^ | ^OPA^ | ^other immunomodulator^ | ^PANSS^ | ^12,0^ | ^17,39^ |
| ^Cheng 2009^ | ^30^ | ^30^ | ^SCZ^ | ^28,97^ | ^31,20^ | ^50,00^ | ^43,00^ | ^SA^ | ^placebo^ | ^BS^ | ^other stimulation^ | ^PANSS^ | ^6,0^ | ^11,66^ |
| ^Chengappa 2018^ | ^34^ | ^34^ | ^SCZ^ | ^47,38^ | ^45,18^ | ^41,00^ | ^62,00^ | ^AA^ | ^placebo^ | ^OPA^ | ^other nutraceutical^ | ^PANSS^ | ^12,0^ | ^10,60^ |
| ^Chien 2015^ | ^57^ | ^57^ | ^SCZ^ | ^NA^ | ^NA^ | ^53,00^ | ^51,00^ | ^ns^ | ^none^ | ^PSI^ | ^supportive counselling^ | ^PANSS^ | ^24,0^ | ^3,51^ |
| ^Chien 2017^ | ^114^ | ^57^ | ^SCZ^ | ^NA^ | ^NA^ | ^65,00^ | ^61,00^ | ^ns^ | ^placebo^ | ^PSI^ | ^group therapy integrated psychological^ | ^PANSS^ | ^96,0^ | ^16,67^ |
| ^Chien 2017^ | ^114^ | ^57^ | ^SCZ^ | ^NA^ | ^NA^ | ^65,00^ | ^63,00^ | ^ns^ | ^placebo^ | ^PSI^ | ^mindfulness^ | ^PANSS^ | ^96,0^ | ^16,67^ |
| ^Cooper 2000^ | ^63^ | ^58^ | ^SCZ^ | ^41,60^ | ^43,00^ | ^72,00^ | ^66,00^ | ^OPA^ | ^placebo^ | ^AP^ | ^other antipsychotic^ | ^SANS^ | ^26,0^ | ^76,03^ |
| ^Correll 2015^ | ^90^ | ^61^ | ^SCZ^ | ^39,70^ | ^40,50^ | ^64,00^ | ^68,00^ | ^ns^ | ^placebo^ | ^AP^ | ^brexpiprazole^ | ^PANSS^ | ^6,0^ | ^2,04^ |
| ^Correll 2015^ | ^182^ | ^61^ | ^SCZ^ | ^39,70^ | ^39,60^ | ^64,00^ | ^61,00^ | ^ns^ | ^placebo^ | ^AP^ | ^brexpiprazole^ | ^PANSS^ | ^6,0^ | ^2,04^ |
| ^Correll 2015^ | ^180^ | ^61^ | ^SCZ^ | ^39,70^ | ^40,80^ | ^64,00^ | ^62,00^ | ^ns^ | ^placebo^ | ^AP^ | ^brexpiprazole^ | ^PANSS^ | ^6,0^ | ^2,04^ |
| ^Correll 2020^ | ^150^ | ^75^ | ^SCZ^ | ^41,40^ | ^42,40^ | ^83,00^ | ^73,00^ | ^none^ | ^placebo^ | ^AP^ | ^other antipsychotic^ | ^PANSS^ | ^4,0^ | ^20,22^ |
| ^Correll 2020^ | ^150^ | ^75^ | ^SCZ^ | ^41,40^ | ^43,50^ | ^83,00^ | ^75,00^ | ^none^ | ^placebo^ | ^AP^ | ^other antipsychotic^ | ^PANSS^ | ^4,0^ | ^20,22^ |
| ^Crawford 2012^ | ^140^ | ^69^ | ^SCZ^ | ^40,00^ | ^41,00^ | ^72,00^ | ^64,00^ | ^AA^ | ^WL^ | ^PSI^ | ^art therapy^ | ^PANSS^ | ^24,0^ | ^14,87^ |
| ^Crawford 2012^ | ^140^ | ^69^ | ^SCZ^ | ^40,00^ | ^42,00^ | ^72,00^ | ^64,00^ | ^AA^ | ^WL^ | ^PSI^ | ^group therapy social skills^ | ^PANSS^ | ^24,0^ | ^14,87^ |
| ^Curcic 2017^ | ^40^ | ^40^ | ^SCZ^ | ^41,75^ | ^39,95^ | ^48,00^ | ^58,00^ | ^AA^ | ^WL^ | ^LS^ | ^physical exercise^ | ^PANSS^ | ^12,0^ | ^0,00^ |
| ^Dâ€™Amato 2011^ | ^39^ | ^38^ | ^SCZ^ | ^32,20^ | ^33,40^ | ^76,00^ | ^74,00^ | ^AA^ | ^none^ | ^PSI^ | ^cognitive remediation^ | ^PANSS^ | ^7,0^ | ^0,00^ |
| ^Dagani 2016^ | ^16^ | ^16^ | ^SCZ^ | ^30,40^ | ^30,40^ | ^81,00^ | ^81,00^ | ^none^ | ^placebo^ | ^OPA^ | ^oxytocin^ | ^PANSS^ | ^4,0^ | ^3,13^ |
| ^De Lucena 2009^ | ^10^ | ^11^ | ^SCZ^ | ^34,73^ | ^34,60^ | ^100,00^ | ^80,00^ | ^SA^ | ^placebo^ | ^OPA^ | ^memantine^ | ^BPRS neg^ | ^12,0^ | ^0,00^ |
| ^Ding 2018^ | ^31^ | ^31^ | ^SCZ^ | ^49,70^ | ^42,40^ | ^45,00^ | ^42,00^ | ^AA^ | ^placebo^ | ^OPA^ | ^other antidepressant^ | ^PANSS NAA/NDE^ | ^8,0^ | ^12,90^ |
| ^Dlabac De Lange 2015^ | ^16^ | ^16^ | ^SCZ^ | ^32,30^ | ^41,80^ | ^75,00^ | ^88,00^ | ^AA^ | ^placebo^ | ^BS^ | ^TMS repetitive^ | ^PANSS^ | ^12,0^ | ^0,00^ |
| ^Duncan 2003^ | ^10^ | ^12^ | ^SCZ^ | ^54,40^ | ^48,70^ | ^100,00^ | ^100,00^ | ^AA^ | ^placebo^ | ^OPA^ | ^D-cycloserine^ | ^SANS^ | ^4,0^ | ^0,00^ |
| ^Fan 2017a^ | ^26^ | ^28^ | ^schizoaffective^ | ^44,40^ | ^41,50^ | ^76,00^ | ^82,00^ | ^SA^ | ^placebo^ | ^OPA^ | ^other pharma^ | ^PANSS^ | ^12,0^ | ^10,19^ |
| ^Farokhnia 2013^ | ^23^ | ^23^ | ^SCZ^ | ^33,38^ | ^32,23^ | ^52,00^ | ^43,00^ | ^SA^ | ^placebo^ | ^OPA^ | ^N-acetyl cysteine^ | ^PANSS^ | ^8,0^ | ^8,70^ |
| ^Farokhnia 2014^ | ^25^ | ^25^ | ^SCZ^ | ^33,64^ | ^32,20^ | ^88,00^ | ^84,00^ | ^SA^ | ^placebo^ | ^OPA^ | ^other pharma^ | ^PANSS^ | ^8,0^ | ^4,00^ |
| ^Farooq 2011^ | ^55^ | ^55^ | ^SCZ^ | ^30,00^ | ^29,00^ | ^86,00^ | ^86,00^ | ^none^ | ^none^ | ^PSI^ | ^supportive counselling^ | ^PANSS^ | ^52,1^ | ^13,63^ |
| ^Favrod 2019^ | ^40^ | ^40^ | ^SCZ^ | ^39,83^ | ^39,98^ | ^70,00^ | ^52,00^ | ^mixed^ | ^none^ | ^PSI^ | ^psychoeducational medication management training^ | ^SANS^ | ^24,0^ | ^20,00^ |
| ^Fekete 2021^ | ^23^ | ^23^ | ^SCZ^ | ^38,39^ | ^44,22^ | ^48,00^ | ^48,00^ | ^mixed^ | ^none^ | ^PSI^ | ^metacognitive training^ | ^PANSS^ | ^26,0^ | ^21,74^ |
| ^Fekete 2022^ | ^23^ | ^23^ | ^SCZ^ | ^38,39^ | ^44,22^ | ^48,00^ | ^48,00^ | ^mixed^ | ^none^ | ^PSI^ | ^metacognitive training^ | ^PANSS^ | ^24,0^ | ^21,70^ |
| ^Frohlich 2016^ | ^13^ | ^13^ | ^SCZ^ | ^40,00^ | ^43,48^ | ^85,00^ | ^69,00^ | ^AA^ | ^placebo^ | ^BS^ | ^TCS direct^ | ^PANSS^ | ^4,3^ | ^0,00^ |
| ^Garg 2016^ | ^24^ | ^23^ | ^SCZ^ | ^30,75^ | ^32,40^ | ^80,00^ | ^85,00^ | ^AA^ | ^placebo^ | ^BS^ | ^TMS repetitive^ | ^PANSS^ | ^4,0^ | ^14,85^ |
| ^Ghajar 2018a^ | ^36^ | ^37^ | ^SCZ^ | ^48,85^ | ^45,36^ | ^85,00^ | ^94,00^ | ^SA^ | ^placebo^ | ^OPA^ | ^other pharma^ | ^PANSS^ | ^8,0^ | ^9,57^ |
| ^Ghajar 2018b^ | ^30^ | ^30^ | ^SCZ^ | ^45,97^ | ^43,67^ | ^87,00^ | ^93,00^ | ^SA^ | ^placebo^ | ^OPA^ | ^other nutraceutical^ | ^PANSS^ | ^8,0^ | ^2,00^ |
| ^Ghaleiha 2010^ | ^23^ | ^20^ | ^SCZ^ | ^33,30^ | ^32,86^ | ^65,00^ | ^66,00^ | ^SA^ | ^placebo^ | ^OPA^ | ^buspirone^ | ^PANSS^ | ^8,0^ | ^0,00^ |
| ^Goff 2007a^ | ^104^ | ^105^ | ^SCZ^ | ^41,00^ | ^41,00^ | ^70,00^ | ^70,00^ | ^AA^ | ^placebo^ | ^OPA^ | ^lamotrigine^ | ^SANS^ | ^12,0^ | ^28,70^ |
| ^Goff 2007b^ | ^104^ | ^105^ | ^SCZ^ | ^41,60^ | ^41,60^ | ^74,00^ | ^74,00^ | ^AA^ | ^placebo^ | ^OPA^ | ^lamotrigine^ | ^SANS^ | ^12,0^ | ^28,70^ |
| ^Goff 2008^ | ^51^ | ^54^ | ^SCZ^ | ^43,70^ | ^42,00^ | ^80,00^ | ^86,00^ | ^AA^ | ^placebo^ | ^OPA^ | ^other glutamatergic^ | ^SANS^ | ^8,0^ | ^9,52^ |
| ^Gokcen 2020^ | ^16^ | ^16^ | ^SCZ^ | ^46,87^ | ^40,25^ | ^69,00^ | ^44,00^ | ^AA^ | ^none^ | ^LS^ | ^dance movement therapy^ | ^PANSS^ | ^8,0^ | ^11,11^ |
| ^Gomes 2018^ | ^12^ | ^12^ | ^SCZ^ | ^33,75^ | ^39,10^ | ^58,00^ | ^83,00^ | ^AA^ | ^placebo^ | ^BS^ | ^TCS direct^ | ^PANSS^ | ^2,0^ | ^4,16^ |
| ^Guan 2020^ | ^28^ | ^28^ | ^SCZ^ | ^56,00^ | ^51,90^ | ^100,00^ | ^100,00^ | ^SA^ | ^placebo^ | ^BS^ | ^TMS repetitive^ | ^PANSS^ | ^8,0^ | ^26,79^ |
| ^Gunduz-Bruce 2013^ | ^14^ | ^14^ | ^SCZ^ | ^41,50^ | ^44,30^ | ^71,00^ | ^71,00^ | ^SA^ | ^placebo^ | ^AP^ | ^other antipsychotic^ | ^SANS^ | ^12,0^ | ^0,00^ |
| ^Hansen 2012^ | ^31^ | ^31^ | ^SCZ^ | ^32,80^ | ^33,20^ | ^68,00^ | ^61,00^ | ^PSI^ | ^none^ | ^PSI^ | ^other PSI^ | ^PANSS^ | ^39,1^ | ^27,42^ |
| ^Hassanpour 2019^ | ^20^ | ^20^ | ^SCZ^ | ^49,30^ | ^43,20^ | ^100,00^ | ^80,00^ | ^AA^ | ^placebo^ | ^OPA^ | ^memantine^ | ^PANSS^ | ^8,0^ | ^0,00^ |
| ^He 2021^ | ^20^ | ^20^ | ^SCZ^ | ^49,30^ | ^45,40^ | ^0,00^ | ^0,00^ | ^ns^ | ^none^ | ^LS^ | ^horticultural therapy^ | ^PANSS^ | ^8,0^ | ^0,00^ |
| ^Heresco-Levy 2005^ | ^19^ | ^20^ | ^SCZ^ | ^44,90^ | ^44,90^ | ^64,00^ | ^64,00^ | ^SA^ | ^placebo^ | ^OPA^ | ^D-serine^ | ^PANSS^ | ^6,0^ | ^7,63^ |
| ^Holi 2004^ | ^11^ | ^11^ | ^SCZ^ | ^34,80^ | ^38,50^ | ^86,00^ | ^86,00^ | ^AA^ | ^placebo^ | ^BS^ | ^TMS repetitive^ | ^PANSS^ | ^2,0^ | ^0,00^ |
| ^Hosseini 2014^ | ^22^ | ^22^ | ^SCZ^ | ^33,68^ | ^34,09^ | ^77,00^ | ^86,00^ | ^SA^ | ^placebo^ | ^OPA^ | ^other hormone^ | ^PANSS^ | ^8,0^ | ^9,09^ |
| ^Hosseininasab 2021^ | ^33^ | ^33^ | ^SCZ^ | ^47,80^ | ^49,30^ | ^100,00^ | ^100,00^ | ^AA^ | ^placebo^ | ^OPA^ | ^other pharma^ | ^PANSS^ | ^16,0^ | ^12,12^ |
| ^Husain 2017^ | ^18^ | ^18^ | ^SCZ_spectrum_unspecified^ | ^30,50^ | ^34,10^ | ^56,00^ | ^44,00^ | ^AA^ | ^none^ | ^PSI^ | ^CBT cognitive behavioral therapy^ | ^PANSS^ | ^24,0^ | ^13,88^ |
| ^Iancu 2010^ | ^20^ | ^20^ | ^SCZ^ | ^38,80^ | ^35,50^ | ^70,00^ | ^75,00^ | ^AA^ | ^placebo^ | ^OPA^ | ^other antidepressant^ | ^PANSS^ | ^10,0^ | ^5,00^ |
| ^Ikai 2014^ | ^25^ | ^25^ | ^SCZ^ | ^48,20^ | ^53,50^ | ^68,00^ | ^64,00^ | ^AA^ | ^none^ | ^PSI^ | ^yoga^ | ^PANSS^ | ^8,0^ | ^28,00^ |
| ^Ikai 2017^ | ^28^ | ^28^ | ^SCZ^ | ^55,00^ | ^55,50^ | ^64,00^ | ^64,00^ | ^AA^ | ^none^ | ^PSI^ | ^yoga^ | ^PANSS^ | ^12,0^ | ^8,93^ |
| ^Iranpour 2016^ | ^21^ | ^21^ | ^SCZ^ | ^37,00^ | ^38,00^ | ^71,00^ | ^67,00^ | ^SA^ | ^placebo^ | ^OPA^ | ^other pharma^ | ^PANSS^ | ^8,0^ | ^4,76^ |
| ^Iwata 2019^ | ^196^ | ^69^ | ^SCZ^ | ^41,50^ | ^40,70^ | ^60,00^ | ^59,00^ | ^ns^ | ^placebo^ | ^AP^ | ^other antipsychotic^ | ^PANSS^ | ^6,0^ | ^22,80^ |
| ^Iwata 2019^ | ^194^ | ^69^ | ^SCZ^ | ^41,50^ | ^40,60^ | ^60,00^ | ^59,00^ | ^ns^ | ^placebo^ | ^AP^ | ^other antipsychotic^ | ^PANSS^ | ^6,0^ | ^22,80^ |
| ^Jeon 2018^ | ^28^ | ^28^ | ^SCZ^ | ^39,86^ | ^40,00^ | ^46,00^ | ^50,00^ | ^AA^ | ^placebo^ | ^BS^ | ^TCS direct^ | ^PANSS^ | ^12,0^ | ^30,35^ |
| ^Jin 2023^ | ^34^ | ^32^ | ^SCZ^ | ^47,47^ | ^47,60^ | ^19,00^ | ^18,00^ | ^SA^ | ^placebo^ | ^BS^ | ^TMS theta burst^ | ^PANSS^ | ^4,0^ | ^9,09^ |
| ^Kaltsatou 2015^ | ^16^ | ^15^ | ^SCZ^ | ^60,40^ | ^59,50^ | ^73,00^ | ^88,00^ | ^AA^ | ^none^ | ^LS^ | ^dance movement therapy^ | ^PANSS^ | ^34,8^ | ^0,00^ |
| ^Kane 2003^ | ^99^ | ^33^ | ^SCZ^ | ^NA^ | ^NA^ | ^84,00^ | ^70,00^ | ^SA^ | ^placebo^ | ^AP^ | ^risperidone^ | ^PANSS^ | ^12,0^ | ^55,75^ |
| ^Kane 2003^ | ^103^ | ^33^ | ^SCZ^ | ^NA^ | ^NA^ | ^84,00^ | ^80,00^ | ^SA^ | ^placebo^ | ^AP^ | ^risperidone^ | ^PANSS^ | ^12,0^ | ^55,75^ |
| ^Kane 2003^ | ^100^ | ^33^ | ^SCZ^ | ^NA^ | ^NA^ | ^84,00^ | ^68,00^ | ^SA^ | ^placebo^ | ^AP^ | ^risperidone^ | ^PANSS^ | ^12,0^ | ^55,75^ |
| ^Kang 2016^ | ^118^ | ^126^ | ^SCZ^ | ^45,90^ | ^45,90^ | ^50,00^ | ^45,00^ | ^AA^ | ^none^ | ^PSI^ | ^group therapy social skills^ | ^PANSS^ | ^52,0^ | ^0,00^ |
| ^Kaphzan 2014^ | ^25^ | ^25^ | ^SCZ^ | ^43,80^ | ^41,80^ | ^64,00^ | ^68,00^ | ^AA^ | ^placebo^ | ^OPA^ | ^pharma antiparkinson^ | ^PANSS^ | ^12,0^ | ^10,00^ |
| ^Karbalaee 2023^ | ^40^ | ^40^ | ^SCZ^ | ^36,57^ | ^35,74^ | ^60,00^ | ^60,00^ | ^SA^ | ^placebo^ | ^OPA^ | ^other immunomodulator^ | ^PANSS^ | ^8,0^ | ^12,50^ |
| ^Kardashev 2018^ | ^19^ | ^21^ | ^SCZ^ | ^33,00^ | ^32,20^ | ^90,00^ | ^89,00^ | ^AA^ | ^placebo^ | ^OPA^ | ^other hormone^ | ^SANS^ | ^8,0^ | ^2,63^ |
| ^Kasckow 2010^ | ^103^ | ^89^ | ^SCZ^ | ^52,50^ | ^52,50^ | ^78,00^ | ^78,00^ | ^AA^ | ^placebo^ | ^OPA^ | ^citalopram^ | ^PANSS^ | ^12,0^ | ^19,27^ |
| ^Kashani 2017^ | ^45^ | ^45^ | ^SCZ^ | ^36,61^ | ^34,85^ | ^0,00^ | ^0,00^ | ^SA^ | ^placebo^ | ^OPA^ | ^other hormone^ | ^PANSS^ | ^8,0^ | ^8,89^ |
| ^Kayo 2020^ | ^29^ | ^33^ | ^SCZ^ | ^35,80^ | ^37,21^ | ^64,00^ | ^79,00^ | ^SA^ | ^none^ | ^PSI^ | ^group therapy social skills^ | ^PANSS^ | ^24,0^ | ^34,73^ |
| ^Khalil 2018^ | ^30^ | ^30^ | ^SCZ^ | ^35,20^ | ^33,10^ | ^67,00^ | ^73,00^ | ^ns^ | ^none^ | ^PSI^ | ^psychoeducational medication management training^ | ^PANSS^ | ^26,0^ | ^16,67^ |
| ^Khodaie-Ardakani 2013^ | ^20^ | ^20^ | ^SCZ^ | ^37,90^ | ^36,70^ | ^55,00^ | ^60,00^ | ^SA^ | ^placebo^ | ^OPA^ | ^pharma antiemetic^ | ^PANSS^ | ^8,0^ | ^5,00^ |
| ^Khodaie-Ardakani 2014^ | ^20^ | ^20^ | ^SCZ^ | ^38,95^ | ^41,05^ | ^75,00^ | ^70,00^ | ^SA^ | ^placebo^ | ^OPA^ | ^minocycline^ | ^PANSS^ | ^8,0^ | ^5,00^ |
| ^Khodaie-Ardakani 2015^ | ^23^ | ^23^ | ^SCZ^ | ^32,40^ | ^31,40^ | ^100,00^ | ^100,00^ | ^SA^ | ^placebo^ | ^OPA^ | ^raloxifene^ | ^PANSS^ | ^8,0^ | ^8,70^ |
| ^Kimhy 2021^ | ^16^ | ^17^ | ^SCZ^ | ^37,24^ | ^36,56^ | ^65,00^ | ^63,00^ | ^AA^ | ^none^ | ^LS^ | ^physical exercise^ | ^SANS^ | ^12,0^ | ^21,21^ |
| ^Kirkpatrick 2017^ | ^78^ | ^42^ | ^SCZ^ | ^40,00^ | ^39,80^ | ^58,00^ | ^53,00^ | ^OPA^ | ^placebo^ | ^OPA^ | ^roluperidone^ | ^PANSS^ | ^12,0^ | ^ns^ |
| ^Kirkpatrick 2017^ | ^83^ | ^42^ | ^SCZ^ | ^40,00^ | ^40,60^ | ^58,00^ | ^58,00^ | ^OPA^ | ^placebo^ | ^OPA^ | ^roluperidone^ | ^PANSS^ | ^12,0^ | ^ns^ |
| ^Klein 1999^ | ^18^ | ^17^ | ^SCZ^ | ^29,50^ | ^30,20^ | ^35,00^ | ^39,00^ | ^AA^ | ^placebo^ | ^BS^ | ^TMS repetitive^ | ^PANSS^ | ^2,0^ | ^11,43^ |
| ^Kremer 2004^ | ^25^ | ^13^ | ^SCZ^ | ^42,30^ | ^44,10^ | ^68,00^ | ^46,00^ | ^AA^ | ^placebo^ | ^OPA^ | ^lamotrigine^ | ^PANSS^ | ^10,0^ | ^18,40^ |
| ^Kucerova 2012^ | ^19^ | ^11^ | ^SCZ^ | ^34,55^ | ^30,47^ | ^100,00^ | ^100,00^ | ^AA^ | ^placebo^ | ^BS^ | ^TMS repetitive^ | ^PANSS^ | ^3,0^ | ^0,00^ |
| ^Kulkarni 2008^ | ^56^ | ^46^ | ^SCZ^ | ^33,80^ | ^33,50^ | ^0,00^ | ^0,00^ | ^AA^ | ^placebo^ | ^OPA^ | ^other hormone^ | ^PANSS^ | ^4,0^ | ^14,70^ |
| ^Kulkarni 2015^ | ^59^ | ^31^ | ^SCZ^ | ^35,44^ | ^34,77^ | ^0,00^ | ^0,00^ | ^mixed^ | ^placebo^ | ^OPA^ | ^other hormone^ | ^PANSS^ | ^8,0^ | ^1,64^ |
| ^Kulkarni 2015^ | ^62^ | ^31^ | ^SCZ^ | ^35,44^ | ^35,29^ | ^0,00^ | ^0,00^ | ^mixed^ | ^placebo^ | ^OPA^ | ^other hormone^ | ^PANSS^ | ^8,0^ | ^1,64^ |
| ^Kulkrani 2016^ | ^26^ | ^30^ | ^SCZ^ | ^53,07^ | ^52,59^ | ^ns^ | ^ns^ | ^mixed^ | ^placebo^ | ^OPA^ | ^raloxifene^ | ^PANSS^ | ^12,0^ | ^17,85^ |
| ^Kumar 2020^ | ^50^ | ^50^ | ^SCZ^ | ^30,80^ | ^32,40^ | ^56,00^ | ^58,00^ | ^mixed^ | ^placebo^ | ^BS^ | ^TMS repetitive^ | ^PANSS^ | ^17,0^ | ^33,00^ |
| ^Laan 2010^ | ^33^ | ^37^ | ^SCZ^ | ^30,60^ | ^9,20^ | ^89,00^ | ^76,00^ | ^AA^ | ^placebo^ | ^OPA^ | ^aspirin^ | ^PANSS^ | ^12,0^ | ^17,14^ |
| ^Lane 2010^ | ^20^ | ^10^ | ^SCZ^ | ^31,50^ | ^30,70^ | ^45,00^ | ^60,00^ | ^AA^ | ^placebo^ | ^OPA^ | ^D-serine^ | ^SANS^ | ^6,0^ | ^15,00^ |
| ^Lane 2010^ | ^20^ | ^10^ | ^SCZ^ | ^31,50^ | ^30,40^ | ^45,00^ | ^60,00^ | ^AA^ | ^placebo^ | ^OPA^ | ^sarcosine^ | ^SANS^ | ^6,0^ | ^15,00^ |
| ^Lane 2013^ | ^25^ | ^27^ | ^SCZ^ | ^36,30^ | ^38,40^ | ^56,00^ | ^44,00^ | ^AA^ | ^placebo^ | ^OPA^ | ^sodium benzoate^ | ^PANSS^ | ^6,0^ | ^9,61^ |
| ^Lee 2013b^ | ^13^ | ^15^ | ^SCZ^ | ^35,07^ | ^44,74^ | ^73,00^ | ^69,00^ | ^AA^ | ^placebo^ | ^OPA^ | ^oxytocin^ | ^SANS^ | ^3,0^ | ^0,00^ |
| ^Lee 2015b^ | ^74^ | ^75^ | ^SCZ^ | ^30,00^ | ^30,90^ | ^61,00^ | ^59,00^ | ^mixed^ | ^placebo^ | ^OPA^ | ^other pharma^ | ^PANSS^ | ^11,0^ | ^36,24^ |
| ^Lerner 2013^ | ^38^ | ^41^ | ^SCZ^ | ^41,70^ | ^41,20^ | ^89,00^ | ^91,00^ | ^mixed^ | ^placebo^ | ^OPA^ | ^other nutraceutical^ | ^PANSS^ | ^6,0^ | ^12,22^ |
| ^Li 2022^ | ^35^ | ^30^ | ^SCZ^ | ^40,52^ | ^43,41^ | ^22,00^ | ^38,00^ | ^AA^ | ^placebo^ | ^OPA^ | ^other nutraceutical^ | ^PANSS^ | ^8,0^ | ^24,62^ |
| ^Lin 2017^ | ^21^ | ^21^ | ^SCZ^ | ^39,10^ | ^38,20^ | ^62,00^ | ^71,00^ | ^AA^ | ^placebo^ | ^OPA^ | ^sarcosine^ | ^PANSS^ | ^12,0^ | ^23,82^ |
| ^Lin 2018^ | ^20^ | ^10^ | ^SCZ^ | ^47,00^ | ^44,30^ | ^70,00^ | ^70,00^ | ^CA^ | ^placebo^ | ^OPA^ | ^sodium benzoate^ | ^PANSS^ | ^6,0^ | ^1,67^ |
| ^Lin 2018^ | ^20^ | ^10^ | ^SCZ^ | ^47,00^ | ^44,80^ | ^70,00^ | ^65,00^ | ^CA^ | ^placebo^ | ^OPA^ | ^sodium benzoate^ | ^PANSS^ | ^6,0^ | ^1,68^ |
| ^Lincoln 2012^ | ^40^ | ^40^ | ^SCZ^ | ^33,10^ | ^33,20^ | ^58,00^ | ^55,00^ | ^AA^ | ^WL^ | ^PSI^ | ^CBT cognitive behavioral therapy^ | ^PANSS^ | ^4,0^ | ^8,75^ |
| ^Lindenmayer 2019^ | ^15^ | ^13^ | ^SCZ^ | ^NA^ | ^NA^ | ^85,00^ | ^87,00^ | ^AA^ | ^placebo^ | ^BS^ | ^TCS direct^ | ^PANSS^ | ^4,0^ | ^25,00^ |
| ^Lisoni 2022^ | ^25^ | ^25^ | ^SCZ^ | ^44,44^ | ^40,95^ | ^64,00^ | ^92,00^ | ^mixed^ | ^placebo^ | ^BS^ | ^TCS direct^ | ^PANSS^ | ^3,0^ | ^0,00^ |
| ^Liu 2014^ | ^46^ | ^46^ | ^SCZ^ | ^27,70^ | ^27,05^ | ^60,00^ | ^64,00^ | ^SA^ | ^placebo^ | ^OPA^ | ^minocycline^ | ^PANSS^ | ^16,0^ | ^14,14^ |
| ^Longden 2022^ | ^24^ | ^26^ | ^SCZ^ | ^40,90^ | ^38,10^ | ^65,00^ | ^42,00^ | ^mixed^ | ^none^ | ^PSI^ | ^other PSI^ | ^PANSS^ | ^26,0^ | ^20,00^ |
| ^Lopez-Navarro 2015^ | ^22^ | ^22^ | ^SCZ^ | ^38,77^ | ^38,73^ | ^77,00^ | ^86,00^ | ^mixed^ | ^none^ | ^PSI^ | ^mindfulness^ | ^PANSS^ | ^26,0^ | ^0,00^ |
| ^Lu 2013^ | ^38^ | ^42^ | ^SCZ^ | ^52,35^ | ^51,66^ | ^76,00^ | ^71,00^ | ^mixed^ | ^none^ | ^PSI^ | ^music therapy^ | ^PANSS^ | ^13,0^ | ^6,25^ |
| ^Luther 2020^ | ^27^ | ^29^ | ^schizoaffective^ | ^46,30^ | ^46,00^ | ^59,00^ | ^44,00^ | ^mixed^ | ^none^ | ^PSI^ | ^supportive counselling^ | ^CAINS map^ | ^8,0^ | ^5,36^ |
| ^Martin 2016^ | ^44^ | ^24^ | ^SCZ_spectrum_unspecified^ | ^37,52^ | ^41,05^ | ^46,00^ | ^57,00^ | ^AA^ | ^WL^ | ^LS^ | ^dance movement therapy^ | ^SANS^ | ^10,0^ | ^23,44^ |
| ^Matsuda 2018^ | ^31^ | ^31^ | ^SCZ^ | ^37,77^ | ^36,39^ | ^58,00^ | ^55,00^ | ^AA^ | ^none^ | ^PSI^ | ^computer assisted cognitive rehabilitation^ | ^PANSS^ | ^12,0^ | ^33,87^ |
| ^Mazinani 2017^ | ^23^ | ^23^ | ^SCZ^ | ^45,30^ | ^44,80^ | ^100,00^ | ^100,00^ | ^SA^ | ^placebo^ | ^OPA^ | ^memantine^ | ^PANSS^ | ^12,0^ | ^21,74^ |
| ^McGuire 2018^ | ^43^ | ^45^ | ^SCZ^ | ^40,80^ | ^40,90^ | ^51,00^ | ^65,00^ | ^AA^ | ^placebo^ | ^OPA^ | ^other pharma^ | ^PANSS^ | ^6,0^ | ^5,68^ |
| ^Meskanen 2013^ | ^16^ | ^14^ | ^SCZ^ | ^52,60^ | ^50,40^ | ^71,00^ | ^50,00^ | ^AA^ | ^placebo^ | ^OPA^ | ^pharma antihistamine^ | ^SANS^ | ^5,0^ | ^0,00^ |
| ^Mico 2011^ | ^20^ | ^20^ | ^SCZ^ | ^34,00^ | ^35,90^ | ^55,00^ | ^65,00^ | ^SA^ | ^placebo^ | ^OPA^ | ^other antidepressant^ | ^PANSS^ | ^16,0^ | ^17,50^ |
| ^Miodownik 2019^ | ^20^ | ^18^ | ^SCZ^ | ^53,40^ | ^54,10^ | ^61,00^ | ^70,00^ | ^AA^ | ^placebo^ | ^OPA^ | ^other nutraceutical^ | ^PANSS^ | ^24,0^ | ^13,16^ |
| ^Miyaoka 2013^ | ^56^ | ^64^ | ^SCZ^ | ^46,40^ | ^46,40^ | ^64,00^ | ^58,00^ | ^AA^ | ^placebo^ | ^OPA^ | ^other nutraceutical^ | ^PANSS^ | ^4,0^ | ^18,33^ |
| ^Modabbernia 2013^ | ^20^ | ^20^ | ^SCZ^ | ^33,20^ | ^32,30^ | ^80,00^ | ^85,00^ | ^SA^ | ^placebo^ | ^OPA^ | ^oxytocin^ | ^PANSS^ | ^8,0^ | ^7,50^ |
| ^Modabbernia 2014^ | ^24^ | ^24^ | ^SCZ^ | ^32,80^ | ^32,70^ | ^67,00^ | ^72,00^ | ^mixed^ | ^placebo^ | ^OPA^ | ^other hormone^ | ^PANSS^ | ^8,0^ | ^25,00^ |
| ^Montag 2014^ | ^29^ | ^29^ | ^SCZ^ | ^38,80^ | ^37,40^ | ^79,00^ | ^66,00^ | ^mixed^ | ^none^ | ^PSI^ | ^art therapy^ | ^SANS^ | ^12,0^ | ^39,67^ |
| ^Morimoto 2018^ | ^16^ | ^15^ | ^SCZ^ | ^37,40^ | ^36,10^ | ^60,00^ | ^62,00^ | ^mixed^ | ^none^ | ^PSI^ | ^computer assisted cognitive rehabilitation^ | ^PANSS^ | ^12,0^ | ^0,00^ |
| ^Morozova 2014^ | ^21^ | ^26^ | ^SCZ^ | ^37,10^ | ^34,93^ | ^100,00^ | ^100,00^ | ^AA^ | ^placebo^ | ^OPA^ | ^other pharma^ | ^PANSS^ | ^4,0^ | ^0,00^ |
| ^Morozova 2017^ | ^40^ | ^40^ | ^SCZ^ | ^36,10^ | ^38,10^ | ^75,00^ | ^55,00^ | ^AA^ | ^placebo^ | ^OPA^ | ^other pharma^ | ^PANSS^ | ^6,0^ | ^0,00^ |
| ^Morrison 2014^ | ^37^ | ^37^ | ^SCZ^ | ^29,68^ | ^32,95^ | ^59,00^ | ^46,00^ | ^PSI^ | ^none^ | ^PSI^ | ^CBT cognitive behavioral therapy^ | ^PANSS^ | ^72,0^ | ^32,39^ |
| ^Morrison 2019^ | ^242^ | ^245^ | ^SCZ_spectrum_unspecified^ | ^42,80^ | ^42,20^ | ^71,00^ | ^73,00^ | ^mixed^ | ^none^ | ^PSI^ | ^CBT cognitive behavioral therapy^ | ^PANSS^ | ^90,0^ | ^12,73^ |
| ^Mortan Sevi 2020^ | ^12^ | ^7^ | ^SCZ^ | ^36,87^ | ^36,87^ | ^74,00^ | ^74,00^ | ^AA^ | ^none^ | ^PSI^ | ^CBT cognitive behavioral therapy^ | ^SANS^ | ^12,0^ | ^17,94^ |
| ^Mortan Sevi 2020^ | ^13^ | ^7^ | ^SCZ^ | ^36,87^ | ^36,87^ | ^74,00^ | ^74,00^ | ^AA^ | ^none^ | ^PSI^ | ^CBT cognitive behavioral therapy^ | ^SANS^ | ^12,0^ | ^17,94^ |
| ^Mortazavi 2015^ | ^15^ | ^15^ | ^SCZ^ | ^NA^ | ^NA^ | ^93,00^ | ^93,00^ | ^mixed^ | ^placebo^ | ^OPA^ | ^other nutraceutical^ | ^PANSS^ | ^6,0^ | ^3,33^ |
| ^Motamed 2022^ | ^20^ | ^20^ | ^SCZ^ | ^35,45^ | ^36,00^ | ^70,00^ | ^60,00^ | ^SA^ | ^placebo^ | ^OPA^ | ^other pharma^ | ^PANSS^ | ^8,0^ | ^10,00^ |
| ^Moazen-Zadeh 2020^ | ^39^ | ^39^ | ^SCZ^ | ^32,88^ | ^34,44^ | ^68,00^ | ^71,00^ | ^SA^ | ^placebo^ | ^OPA^ | ^other antidepressant^ | ^PANSS^ | ^8,0^ | ^12,82^ |
| ^Mueller 2017^ | ^28^ | ^33^ | ^SCZ^ | ^34,50^ | ^36,70^ | ^79,00^ | ^76,00^ | ^mixed^ | ^none^ | ^PSI^ | ^cognitive remediation^ | ^PANSS^ | ^52,0^ | ^19,67^ |
| ^Mueller 2020^ | ^32^ | ^26^ | ^SCZ^ | ^31,50^ | ^32,10^ | ^62,00^ | ^56,00^ | ^mixed^ | ^none^ | ^PSI^ | ^cognitive remediation^ | ^PANSS^ | ^52,0^ | ^10,34^ |
| ^Müller 2010^ | ^25^ | ^25^ | ^SCZ^ | ^30,90^ | ^26,20^ | ^64,00^ | ^56,00^ | ^SA^ | ^placebo^ | ^OPA^ | ^other immunomodulator^ | ^PANSS^ | ^6,0^ | ^26,00^ |
| ^Muscatello 2014^ | ^20^ | ^20^ | ^SCZ^ | ^33,50^ | ^36,50^ | ^40,00^ | ^25,00^ | ^SA^ | ^placebo^ | ^AP^ | ^ziprasidone^ | ^PANSS^ | ^16,0^ | ^17,50^ |
| ^Naeem 2015^ | ^59^ | ^57^ | ^SCZ_spectrum_unspecified^ | ^31,10^ | ^31,70^ | ^54,00^ | ^61,00^ | ^AA^ | ^none^ | ^PSI^ | ^CBT cognitive behavioral therapy^ | ^PANSS^ | ^16,0^ | ^12,07^ |
| ^Naeem 2016^ | ^18^ | ^15^ | ^SCZ^ | ^38,60^ | ^42,00^ | ^60,00^ | ^44,00^ | ^ns^ | ^none^ | ^PSI^ | ^CBT cognitive behavioral therapy^ | ^PANSS^ | ^16,0^ | ^15,15^ |
| ^Nasrallah 2013^ | ^122^ | ^43^ | ^SCZ^ | ^38,20^ | ^38,60^ | ^73,00^ | ^67,00^ | ^ns^ | ^placebo^ | ^AP^ | ^lurasidone^ | ^PANSS^ | ^6,0^ | ^34,50^ |
| ^Nasrallah 2013^ | ^119^ | ^43^ | ^SCZ^ | ^38,20^ | ^37,60^ | ^73,00^ | ^64,00^ | ^ns^ | ^placebo^ | ^AP^ | ^lurasidone^ | ^PANSS^ | ^6,0^ | ^34,50^ |
| ^Nasrallah 2013^ | ^124^ | ^43^ | ^SCZ^ | ^38,20^ | ^37,60^ | ^73,00^ | ^74,00^ | ^ns^ | ^placebo^ | ^AP^ | ^lurasidone^ | ^PANSS^ | ^6,0^ | ^34,50^ |
| ^Niitsu 2012^ | ^24^ | ^24^ | ^SCZ^ | ^36,30^ | ^38,60^ | ^62,00^ | ^61,00^ | ^AA^ | ^placebo^ | ^OPA^ | ^fluvoxamine^ | ^SANS^ | ^12,0^ | ^8,33^ |
| ^Nikbakhat 2016^ | ^34^ | ^34^ | ^SCZ^ | ^34,22^ | ^33,94^ | ^69,00^ | ^66,00^ | ^SA^ | ^placebo^ | ^OPA^ | ^other antidepressant^ | ^PANSS^ | ^8,0^ | ^5,88^ |
| ^Noroozian 2013^ | ^20^ | ^20^ | ^SCZ^ | ^33,70^ | ^33,80^ | ^75,00^ | ^80,00^ | ^SA^ | ^placebo^ | ^OPA^ | ^pharma antiemetic^ | ^PANSS^ | ^8,0^ | ^7,50^ |
| ^Oh 2023^ | ^38^ | ^32^ | ^SCZ^ | ^39,28^ | ^37,05^ | ^53,00^ | ^47,00^ | ^mixed^ | ^none^ | ^PSI^ | ^other PSI^ | ^PANSS^ | ^26,0^ | ^35,71^ |
| ^Palm 2016^ | ^10^ | ^10^ | ^SCZ^ | ^34,10^ | ^38,40^ | ^100,00^ | ^50,00^ | ^AA^ | ^placebo^ | ^BS^ | ^TCS direct^ | ^PANSS^ | ^4,0^ | ^0,00^ |
| ^Palma-Sevillano 2011^ | ^21^ | ^13^ | ^SCZ^ | ^23,77^ | ^24,00^ | ^85,00^ | ^76,00^ | ^ns^ | ^none^ | ^PSI^ | ^other PSI^ | ^PANSS^ | ^24,0^ | ^0,00^ |
| ^Park 2020^ | ^30^ | ^29^ | ^SCZ^ | ^40,86^ | ^38,37^ | ^66,00^ | ^60,00^ | ^ns^ | ^none^ | ^PSI^ | ^metacognitive training^ | ^SANS^ | ^14,0^ | ^0,00^ |
| ^Pawelczyk 2016^ | ^36^ | ^35^ | ^SCZ^ | ^23,30^ | ^23,20^ | ^66,00^ | ^53,00^ | ^AA^ | ^placebo^ | ^OPA^ | ^omega 3^ | ^PANSS^ | ^26,0^ | ^8,45^ |
| ^Pierre 2007^ | ^10^ | ^10^ | ^SCZ^ | ^49,80^ | ^49,70^ | ^90,00^ | ^100,00^ | ^AA^ | ^placebo^ | ^OPA^ | ^armodafinil modafinil^ | ^SANS^ | ^8,0^ | ^15,00^ |
| ^Poyurovsky 2007^ | ^31^ | ^28^ | ^SCZ^ | ^29,50^ | ^30,30^ | ^54,00^ | ^74,00^ | ^SA^ | ^placebo^ | ^AP^ | ^olanzapine^ | ^SANS^ | ^6,0^ | ^30,51^ |
| ^Prikryl 2007^ | ^11^ | ^11^ | ^SCZ^ | ^36,46^ | ^31,36^ | ^1,00^ | ^1,00^ | ^AA^ | ^placebo^ | ^BS^ | ^TMS repetitive^ | ^PANSS^ | ^3,0^ | ^0,00^ |
| ^Prikryl 2012^ | ^19^ | ^11^ | ^SCZ^ | ^34,55^ | ^30,47^ | ^100,00^ | ^100,00^ | ^AA^ | ^placebo^ | ^BS^ | ^TMS repetitive^ | ^PANSS^ | ^3,0^ | ^0,00^ |
| ^Prikryl 2013^ | ^25^ | ^20^ | ^SCZ^ | ^33,94^ | ^31,60^ | ^100,00^ | ^100,00^ | ^mixed^ | ^placebo^ | ^BS^ | ^TMS repetitive^ | ^SANS^ | ^3,0^ | ^11,11^ |
| ^Prikryl 2014^ | ^18^ | ^17^ | ^SCZ^ | ^34,58^ | ^30,40^ | ^100,00^ | ^100,00^ | ^mixed^ | ^placebo^ | ^BS^ | ^TMS repetitive^ | ^PANSS^ | ^3,0^ | ^12,50^ |
| ^Quan 2015^ | ^78^ | ^39^ | ^SCZ^ | ^46,87^ | ^46,87^ | ^72,00^ | ^56,00^ | ^AA^ | ^placebo^ | ^BS^ | ^TMS repetitive^ | ^PANSS^ | ^6,0^ | ^7,69^ |
| ^Rakitzi 2016^ | ^24^ | ^24^ | ^SCZ^ | ^33,80^ | ^31,30^ | ^67,00^ | ^67,00^ | ^mixed^ | ^none^ | ^PSI^ | ^group therapy integrated psychological^ | ^PANSS^ | ^13,0^ | ^31,25^ |
| ^Ray 2015^ | ^23^ | ^22^ | ^SCZ^ | ^29,30^ | ^31,35^ | ^ns^ | ^ns^ | ^AA^ | ^placebo^ | ^BS^ | ^TMS repetitive^ | ^PANSS^ | ^6,0^ | ^11,11^ |
| ^Rector 2003^ | ^24^ | ^18^ | ^SCZ^ | ^41,20^ | ^37,50^ | ^28,00^ | ^62,00^ | ^mixed^ | ^none^ | ^PSI^ | ^CBT cognitive behavioral therapy^ | ^PANSS^ | ^48,0^ | ^16,00^ |
| ^Ritsner 2011^ | ^19^ | ^21^ | ^SCZ^ | ^32,30^ | ^35,40^ | ^67,00^ | ^89,00^ | ^AA^ | ^placebo^ | ^OPA^ | ^other nutraceutical^ | ^PANSS^ | ^8,0^ | ^33,33^ |
| ^Ritsner 2014^ | ^29^ | ^31^ | ^SCZ^ | ^27,80^ | ^26,90^ | ^1,00^ | ^1,00^ | ^CA^ | ^placebo^ | ^OPA^ | ^other hormone^ | ^PANSS^ | ^8,0^ | ^13,33^ |
| ^Roberts 2014^ | ^33^ | ^33^ | ^schizoaffective^ | ^39,40^ | ^40,00^ | ^67,00^ | ^67,00^ | ^mixed^ | ^none^ | ^PSI^ | ^group therapy social skills^ | ^PANSS^ | ^38,6^ | ^9,10^ |
| ^Roffman 2018^ | ^29^ | ^26^ | ^SCZ^ | ^44,70^ | ^46,30^ | ^73,00^ | ^83,00^ | ^mixed^ | ^placebo^ | ^OPA^ | ^other nutraceutical^ | ^PANSS^ | ^12,0^ | ^9,09^ |
| ^Salimi 2008^ | ^25^ | ^25^ | ^SCZ^ | ^34,24^ | ^33,12^ | ^1,00^ | ^1,00^ | ^SA^ | ^placebo^ | ^OPA^ | ^other pharma^ | ^PANSS^ | ^8,0^ | ^6,00^ |
| ^Samaei 2020^ | ^26^ | ^26^ | ^SCZ^ | ^33,08^ | ^34,73^ | ^58,00^ | ^62,00^ | ^SA^ | ^placebo^ | ^OPA^ | ^other nutraceutical^ | ^PANSS^ | ^8,0^ | ^0,00^ |
| ^Schoemaker 2014^ | ^71^ | ^35^ | ^SCZ^ | ^38,10^ | ^37,40^ | ^66,00^ | ^58,00^ | ^mixed^ | ^placebo^ | ^OPA^ | ^other pharma^ | ^PANSS^ | ^12,0^ | ^10,75^ |
| ^Schoemaker 2014^ | ^73^ | ^35^ | ^SCZ^ | ^38,10^ | ^38,80^ | ^66,00^ | ^63,00^ | ^mixed^ | ^placebo^ | ^OPA^ | ^other pharma^ | ^PANSS^ | ^12,0^ | ^10,75^ |
| ^Shafti 2015^ | ^25^ | ^25^ | ^SCZ^ | ^39,84^ | ^41,00^ | ^100,00^ | ^100,00^ | ^SA^ | ^placebo^ | ^OPA^ | ^reboxetine^ | ^SANS^ | ^12,0^ | ^0,00^ |
| ^Shafti 2016b^ | ^18^ | ^18^ | ^SCZ^ | ^46,44^ | ^44,56^ | ^100,00^ | ^100,00^ | ^AA^ | ^placebo^ | ^OPA^ | ^anticholinesterase^ | ^PANSS^ | ^12,0^ | ^0,00^ |
| ^Sheikhmoonesi 2015^ | ^25^ | ^25^ | ^SCZ^ | ^47,32^ | ^46,68^ | ^80,00^ | ^80,00^ | ^AA^ | ^placebo^ | ^OPA^ | ^buspirone^ | ^PANSS^ | ^6,0^ | ^2,00^ |
| ^Shiina 2010^ | ^20^ | ^20^ | ^SCZ^ | ^35,15^ | ^34,96^ | ^50,00^ | ^45,00^ | ^SA^ | ^placebo^ | ^OPA^ | ^tropisetron^ | ^PANSS^ | ^8,0^ | ^17,50^ |
| ^Singh 2011^ | ^54^ | ^17^ | ^SCZ^ | ^15,70^ | ^15,10^ | ^45,00^ | ^56,00^ | ^mixed^ | ^placebo^ | ^AP^ | ^paliperidone^ | ^PANSS NAA/NDE^ | ^6,0^ | ^0,00^ |
| ^Singh 2011^ | ^48^ | ^17^ | ^SCZ^ | ^15,70^ | ^15,30^ | ^45,00^ | ^65,00^ | ^mixed^ | ^placebo^ | ^AP^ | ^paliperidone^ | ^PANSS NAA/NDE^ | ^6,0^ | ^0,00^ |
| ^Singh 2011^ | ^47^ | ^17^ | ^SCZ^ | ^15,70^ | ^15,50^ | ^45,00^ | ^70,00^ | ^mixed^ | ^placebo^ | ^AP^ | ^paliperidone^ | ^PANSS NAA/NDE^ | ^6,0^ | ^0,00^ |
| ^Singh 2020^ | ^15^ | ^15^ | ^SCZ^ | ^29,80^ | ^33,30^ | ^53,00^ | ^60,00^ | ^mixed^ | ^placebo^ | ^BS^ | ^TMS repetitive^ | ^PANSS^ | ^4,0^ | ^13,33^ |
| ^Sommer 2021^ | ^61^ | ^58^ | ^SCZ_spectrum_unspecified^ | ^NA^ | ^NA^ | ^ns^ | ^ns^ | ^AA^ | ^placebo^ | ^OPA^ | ^simvastatin^ | ^PANSS^ | ^96,0^ | ^41,27^ |
| ^Stauffer 2013^ | ^82^ | ^82^ | ^SCZ^ | ^42,80^ | ^43,70^ | ^78,00^ | ^77,00^ | ^AA^ | ^placebo^ | ^OPA^ | ^other glutamatergic^ | ^PANSS^ | ^16,0^ | ^34,13^ |
| ^Stone 2016^ | ^11^ | ^10^ | ^SCZ_spectrum_unspecified^ | ^40,00^ | ^34,00^ | ^80,00^ | ^70,00^ | ^AA^ | ^placebo^ | ^OPA^ | ^intravenous sodium nitroprusside^ | ^PANSS^ | ^4,0^ | ^4,55^ |
| ^Su 2022^ | ^27^ | ^20^ | ^SCZ^ | ^55,60^ | ^52,70^ | ^100,00^ | ^100,00^ | ^AA^ | ^placebo^ | ^BS^ | ^TMS repetitive^ | ^PANSS^ | ^4,0^ | ^19,15^ |
| ^Su 2023^ | ^26^ | ^17^ | ^SCZ^ | ^55,20^ | ^52,90^ | ^100,00^ | ^100,00^ | ^AA^ | ^placebo^ | ^BS^ | ^TMS repetitive^ | ^PANSS^ | ^4,0^ | ^0,00^ |
| ^Tajik-Esmaeeli 2017^ | ^36^ | ^36^ | ^SCZ^ | ^44,64^ | ^43,18^ | ^85,00^ | ^94,00^ | ^SA^ | ^placebo^ | ^OPA^ | ^simvastatin^ | ^PANSS^ | ^8,0^ | ^8,33^ |
| ^Talwar 2006^ | ^33^ | ^48^ | ^SCZ^ | ^38,70^ | ^35,40^ | ^77,00^ | ^70,00^ | ^AA^ | ^WL^ | ^PSI^ | ^music therapy^ | ^PANSS^ | ^12,0^ | ^14,80^ |
| ^Tiihonen 2003^ | ^34^ | ^34^ | ^SCZ^ | ^38,30^ | ^38,20^ | ^100,00^ | ^100,00^ | ^AA^ | ^placebo^ | ^OPA^ | ^lamotrigine^ | ^PANSS^ | ^14,0^ | ^25,00^ |
| ^Tsai 1999^ | ^10^ | ^10^ | ^SCZ^ | ^39,50^ | ^42,60^ | ^50,00^ | ^60,00^ | ^SA^ | ^placebo^ | ^OPA^ | ^D-serine^ | ^SANS^ | ^6,0^ | ^0,00^ |
| ^Tsai 2006^ | ^14^ | ^18^ | ^SCZ^ | ^31,80^ | ^30,90^ | ^61,00^ | ^29,00^ | ^AA^ | ^placebo^ | ^OPA^ | ^other pharma^ | ^SANS^ | ^6,0^ | ^2,78^ |
| ^Turner 2021^ | ^74^ | ^74^ | ^SCZ^ | ^39,90^ | ^37,80^ | ^66,00^ | ^68,00^ | ^mixed^ | ^placebo^ | ^OPA^ | ^other pharma^ | ^PANSS^ | ^24,0^ | ^24,32^ |
| ^Tyagi 2022^ | ^30^ | ^29^ | ^SCZ^ | ^33,31^ | ^32,17^ | ^59,00^ | ^70,00^ | ^mixed^ | ^placebo^ | ^BS^ | ^TMS theta burst^ | ^PANSS^ | ^2,0^ | ^15,25^ |
| ^Usall 2011^ | ^16^ | ^17^ | ^SCZ^ | ^62,66^ | ^60,14^ | ^0,00^ | ^0,00^ | ^AA^ | ^placebo^ | ^OPA^ | ^raloxifene^ | ^PANSS^ | ^12,0^ | ^6,25^ |
| ^Usall 2014^ | ^34^ | ^17^ | ^SCZ^ | ^44,15^ | ^40,02^ | ^67,00^ | ^79,00^ | ^AA^ | ^placebo^ | ^OPA^ | ^reboxetine^ | ^PANSS^ | ^26,1^ | ^28,89^ |
| ^Usall 2014^ | ^23^ | ^17^ | ^SCZ^ | ^44,15^ | ^42,47^ | ^67,00^ | ^78,00^ | ^AA^ | ^placebo^ | ^OPA^ | ^citalopram^ | ^PANSS^ | ^26,1^ | ^28,89^ |
| ^Usall 2016^ | ^38^ | ^32^ | ^SCZ^ | ^61,34^ | ^62,03^ | ^0,00^ | ^0,00^ | ^mixed^ | ^placebo^ | ^OPA^ | ^raloxifene^ | ^PANSS^ | ^24,0^ | ^18,57^ |
| ^Valencia 2007^ | ^49^ | ^49^ | ^SCZ^ | ^30,10^ | ^29,70^ | ^85,00^ | ^72,00^ | ^AA^ | ^none^ | ^PSI^ | ^other PSI^ | ^PANSS^ | ^48,0^ | ^16,30^ |
| ^Valencia 2010^ | ^54^ | ^53^ | ^SCZ^ | ^29,50^ | ^29,90^ | ^72,00^ | ^72,00^ | ^AA^ | ^none^ | ^PSI^ | ^group therapy social skills^ | ^PANSS^ | ^52,1^ | ^22,43^ |
| ^Valiengo 2020^ | ^50^ | ^50^ | ^SCZ^ | ^35,90^ | ^34,60^ | ^78,00^ | ^82,00^ | ^mixed^ | ^placebo^ | ^BS^ | ^TCS direct^ | ^PANSS^ | ^12,0^ | ^6,00^ |
| ^Vaskinn 2019^ | ^24^ | ^24^ | ^SCZ^ | ^30,80^ | ^29,90^ | ^58,00^ | ^75,00^ | ^mixed^ | ^none^ | ^PSI^ | ^emotional affect recognition^ | ^PANSS^ | ^12,0^ | ^33,33^ |
| ^Vass 2022^ | ^22^ | ^21^ | ^SCZ^ | ^42,47^ | ^36,71^ | ^48,00^ | ^57,00^ | ^AA^ | ^placebo^ | ^PSI^ | ^other PSI^ | ^PANSS^ | ^13,0^ | ^6,98^ |
| ^Vita 2011^ | ^16^ | ^16^ | ^SCZ^ | ^39,90^ | ^34,60^ | ^80,00^ | ^88,00^ | ^AA^ | ^none^ | ^PSI^ | ^group therapy integrated psychological^ | ^PANSS^ | ^24,0^ | ^3,13^ |
| ^Vreeland 2006^ | ^40^ | ^34^ | ^SCZ^ | ^NA^ | ^NA^ | ^38,00^ | ^50,00^ | ^mixed^ | ^none^ | ^PSI^ | ^psychoeducational medication management training^ | ^PANSS^ | ^24,0^ | ^17,57^ |
| ^Wang C 2013^ | ^129^ | ^132^ | ^SCZ^ | ^35,40^ | ^34,30^ | ^51,00^ | ^51,00^ | ^OPA^ | ^placebo^ | ^AP^ | ^olanzapine^ | ^PANSS^ | ^12,0^ | ^13,79^ |
| ^Wang L 2016^ | ^46^ | ^23^ | ^SCZ_spectrum_unspecified^ | ^25,00^ | ^24,10^ | ^54,00^ | ^50,00^ | ^mixed^ | ^none^ | ^PSI^ | ^other PSI^ | ^PANSS^ | ^24,0^ | ^6,87^ |
| ^Wang L 2016^ | ^46^ | ^23^ | ^SCZ_spectrum_unspecified^ | ^25,00^ | ^23,80^ | ^54,00^ | ^52,00^ | ^mixed^ | ^none^ | ^PSI^ | ^mindfulness^ | ^PANSS^ | ^24,0^ | ^6,87^ |
| ^Wang Y 2021^ | ^45^ | ^44^ | ^SCZ^ | ^47,02^ | ^47,40^ | ^61,00^ | ^60,00^ | ^AA^ | ^placebo^ | ^OPA^ | ^pharma antihistamine^ | ^PANSS^ | ^12,0^ | ^14,60^ |
| ^Weiner 2010^ | ^30^ | ^34^ | ^SCZ^ | ^44,10^ | ^48,30^ | ^74,00^ | ^63,00^ | ^SA^ | ^placebo^ | ^AP^ | ^risperidone^ | ^SANS^ | ^16,0^ | ^17,19^ |
| ^Wobrock 2015^ | ^76^ | ^81^ | ^SCZ^ | ^34,90^ | ^36,20^ | ^69,00^ | ^82,00^ | ^mixed^ | ^placebo^ | ^BS^ | ^TMS repetitive^ | ^PANSS^ | ^3,0^ | ^19,75^ |
| ^Wykes 2007^ | ^43^ | ^42^ | ^SCZ^ | ^36,00^ | ^36,00^ | ^73,00^ | ^73,00^ | ^AA^ | ^none^ | ^PSI^ | ^cognitive remediation^ | ^PANSS^ | ^40,0^ | ^21,18^ |
| ^Xiu 2020^ | ^40^ | ^20^ | ^SCZ^ | ^54,70^ | ^50,70^ | ^100,00^ | ^100,00^ | ^AA^ | ^placebo^ | ^BS^ | ^TMS repetitive^ | ^PANSS^ | ^32,0^ | ^25,83^ |
| ^Xiu 2020^ | ^40^ | ^20^ | ^SCZ^ | ^54,70^ | ^52,00^ | ^100,00^ | ^100,00^ | ^AA^ | ^placebo^ | ^BS^ | ^TMS repetitive^ | ^PANSS^ | ^32,0^ | ^25,83^ |
| ^Yassini 2014^ | ^20^ | ^20^ | ^SCZ^ | ^49,00^ | ^49,58^ | ^60,00^ | ^65,00^ | ^mixed^ | ^placebo^ | ^OPA^ | ^other antidepressant^ | ^SANS^ | ^12,0^ | ^0,00^ |
| ^Yeh 2023^ | ^17^ | ^18^ | ^SCZ^ | ^43,17^ | ^42,12^ | ^56,00^ | ^47,00^ | ^AA^ | ^placebo^ | ^BS^ | ^TCS alternate^ | ^PANSS^ | ^4,4^ | ^0,00^ |
| ^Zhang 2011^ | ^78^ | ^79^ | ^SCZ^ | ^45,40^ | ^45,20^ | ^100,00^ | ^100,00^ | ^AA^ | ^placebo^ | ^OPA^ | ^ginkgo biloba^ | ^PANSS^ | ^12,0^ | ^8,92^ |
| ^Zhang 2015^ | ^125^ | ^118^ | ^SCZ^ | ^39,70^ | ^18,20^ | ^53,00^ | ^51,00^ | ^mixed^ | ^none^ | ^PSI^ | ^other PSI^ | ^PANSS^ | ^52,0^ | ^19,34^ |
| ^Zhu 2016^ | ^55^ | ^55^ | ^SCZ^ | ^46,50^ | ^46,50^ | ^44,00^ | ^44,00^ | ^AA^ | ^none^ | ^LS^ | ^horticultural therapy^ | ^PANSS^ | ^12,0^ | ^5,45^ |
| ^Zhu 2020^ | ^78^ | ^79^ | ^SCZ^ | ^43,65^ | ^43,74^ | ^54,00^ | ^54,00^ | ^mixed^ | ^none^ | ^PSI^ | ^computer assisted cognitive rehabilitation^ | ^PANSS^ | ^12,0^ | ^19,75^ |
| ^Zhu 2021^ | ^32^ | ^32^ | ^SCZ^ | ^35,34^ | ^35,16^ | ^44,00^ | ^56,00^ | ^mixed^ | ^placebo^ | ^BS^ | ^TMS theta burst^ | ^PANSS^ | ^12,0^ | ^12,50^ |
| ^Amiri 2008^ | ^20^ | ^20^ | ^SCZ^ | ^33,65^ | ^32,70^ | ^75,00^ | ^70,00^ | ^SA^ | ^placebo^ | ^OPA^ | ^selegiline^ | ^PANSS^ | ^8,0^ | ^5,00^ |
| ^Böge 2021^ | ^21^ | ^19^ | ^SCZ_spectrum_unspecified^ | ^42,74^ | ^37,71^ | ^62,00^ | ^52,00^ | ^mixed^ | ^WL^ | ^PSI^ | ^mindfulness^ | ^PANSS^ | ^4,0^ | ^5,12^ |
| ^Brand 2023^ | ^52^ | ^50^ | ^SCZ^ | ^39,30^ | ^42,00^ | ^71,00^ | ^73,00^ | ^AA^ | ^placebo^ | ^OPA^ | ^raloxifene^ | ^PANSS^ | ^38,0^ | ^8,82^ |
| ^Chaudhry 2024^ | ^76^ | ^37^ | ^SCZ_spectrum_unspecified^ | ^32,90^ | ^32,70^ | ^55,00^ | ^52,00^ | ^AA^ | ^placebo^ | ^OPA^ | ^other statin^ | ^PANSS^ | ^24,0^ | ^22,59^ |
| ^Chaudhry 2024^ | ^73^ | ^37^ | ^SCZ_spectrum_unspecified^ | ^32,90^ | ^32,20^ | ^55,00^ | ^70,00^ | ^AA^ | ^placebo^ | ^OPA^ | ^ondansetron^ | ^PANSS^ | ^24,0^ | ^22,59^ |
| ^Citrome 2024^ | ^206^ | ^102^ | ^SCZ^ | ^42,10^ | ^41,50^ | ^64,00^ | ^64,00^ | ^none^ | ^placebo^ | ^AP^ | ^asenapine^ | ^PANSS NAA/NDE^ | ^6,0^ | ^1,50^ |
| ^Citrome 2024^ | ^206^ | ^102^ | ^SCZ^ | ^42,10^ | ^42,30^ | ^64,00^ | ^54,00^ | ^none^ | ^placebo^ | ^AP^ | ^asenapine^ | ^PANSS NAA/NDE^ | ^6,0^ | ^1,50^ |
| ^Hu 2023^ | ^47^ | ^42^ | ^SCZ^ | ^26,36^ | ^26,76^ | ^45,00^ | ^53,00^ | ^AA^ | ^placebo^ | ^BS^ | ^TMS repetitive^ | ^PANSS^ | ^4,0^ | ^0,00^ |
| ^Kalejahi 2023^ | ^24^ | ^24^ | ^SCZ^ | ^41,42^ | ^41,80^ | ^100,00^ | ^100,00^ | ^AA^ | ^placebo^ | ^OPA^ | ^vitamin D^ | ^PANSS^ | ^8,0^ | ^12,50^ |
| ^Kos 2024 1^ | ^17^ | ^17^ | ^SCZ^ | ^34,00^ | ^41,00^ | ^76,00^ | ^94,00^ | ^AA^ | ^placebo^ | ^BS^ | ^TCS direct^ | ^SANS^ | ^10,0^ | ^17,07^ |
| ^Kos 2024 2^ | ^32^ | ^16^ | ^SCZ^ | ^34,00^ | ^33,00^ | ^75,00^ | ^75,00^ | ^AA^ | ^placebo^ | ^BS^ | ^TMS theta burst^ | ^SANS^ | ^10,0^ | ^17,07^ |
| ^Lyu 2024^ | ^35^ | ^29^ | ^SCZ^ | ^56,38^ | ^57,00^ | ^75,00^ | ^64,00^ | ^AA^ | ^placebo^ | ^BS^ | ^TCS direct^ | ^PANSS^ | ^5,0^ | ^17,19^ |
| ^Mao 2023^ | ^30^ | ^30^ | ^SCZ^ | ^38,25^ | ^38,17^ | ^58,00^ | ^45,00^ | ^AA^ | ^placebo^ | ^BS^ | ^TMS repetitive^ | ^PANSS^ | ^4,0^ | ^11,67^ |
| ^Meyer-Lindenberg 2022^ | ^54^ | ^27^ | ^SCZ^ | ^41,70^ | ^43,20^ | ^63,00^ | ^59,00^ | ^none^ | ^placebo^ | ^OPA^ | ^other pharma^ | ^PANSS^ | ^12,0^ | ^31,48^ |
| ^Meyer-Lindenberg 2022^ | ^54^ | ^27^ | ^SCZ^ | ^41,70^ | ^43,00^ | ^63,00^ | ^57,00^ | ^none^ | ^placebo^ | ^OPA^ | ^other pharma^ | ^PANSS^ | ^12,0^ | ^31,48^ |
| ^Müller 2020^ | ^13^ | ^12^ | ^SCZ^ | ^17,08^ | ^17,46^ | ^58,00^ | ^54,00^ | ^AA^ | ^none^ | ^PSI^ | ^CBT cognitive behavioral therapy^ | ^PANSS^ | ^36,0^ | ^12,00^ |
| ^Perez-Aguado 2024^ | ^30^ | ^30^ | ^SCZ^ | ^42,07^ | ^36,20^ | ^80,00^ | ^80,00^ | ^mixed^ | ^none^ | ^PSI^ | ^music therapy^ | ^PANSS^ | ^22,0^ | ^5,00^ |
| ^Poulou 2025^ | ^25^ | ^24^ | ^SCZ^ | ^60,39^ | ^61,58^ | ^52,00^ | ^57,00^ | ^mixed^ | ^none^ | ^PSI^ | ^other PSI^ | ^PANSS^ | ^52,0^ | ^20,41^ |
| ^Saporta-Wiesel 2024 1^ | ^11^ | ^12^ | ^SCZ^ | ^37,83^ | ^39,45^ | ^67,00^ | ^91,00^ | ^mixed^ | ^placebo^ | ^OPA^ | ^oxytocin^ | ^PANSS^ | ^3,0^ | ^7,84^ |
| ^Saporta-Wiesel 2024 2^ | ^11^ | ^13^ | ^SCZ^ | ^36,30^ | ^33,09^ | ^77,00^ | ^100,00^ | ^mixed^ | ^placebo^ | ^OPA^ | ^oxytocin^ | ^PANSS^ | ^3,0^ | ^7,84^ |
| ^Shamabadi 2023^ | ^40^ | ^40^ | ^SCZ^ | ^33,63^ | ^36,43^ | ^63,00^ | ^57,00^ | ^SA^ | ^placebo^ | ^OPA^ | ^other nutraceutical^ | ^PANSS^ | ^8,0^ | ^25,00^ |
| ^Shen 2023^ | ^50^ | ^50^ | ^SCZ^ | ^59,76^ | ^59,24^ | ^70,00^ | ^66,00^ | ^mixed^ | ^none^ | ^PSI^ | ^mindfulness^ | ^PANSS^ | ^6,0^ | ^4,00^ |
| ^Shen 2024^ | ^20^ | ^20^ | ^SCZ^ | ^63,10^ | ^58,95^ | ^45,00^ | ^35,00^ | ^AA^ | ^none^ | ^LS^ | ^physical exercise^ | ^SANS^ | ^12,0^ | ^0,00^ |
| ^Shi 2024^ | ^30^ | ^30^ | ^SCZ^ | ^45,10^ | ^40,88^ | ^66,00^ | ^62,00^ | ^AA^ | ^placebo^ | ^PSI^ | ^other PSI^ | ^PANSS^ | ^12,0^ | ^8,33^ |
| ^Sinichi 2023^ | ^26^ | ^26^ | ^SCZ^ | ^41,23^ | ^38,38^ | ^58,00^ | ^77,00^ | ^AA^ | ^placebo^ | ^OPA^ | ^other pharma^ | ^PANSS^ | ^8,0^ | ^0,00^ |
| ^Tharoor 2023^ | ^50^ | ^50^ | ^SCZ^ | ^31,00^ | ^32,10^ | ^62,00^ | ^70,00^ | ^AA^ | ^placebo^ | ^OPA^ | ^other nutraceutical^ | ^SANS^ | ^24,0^ | ^30,00^ |
| ^Tuppurainen 2024^ | ^22^ | ^22^ | ^SCZ^ | ^37,00^ | ^38,00^ | ^100,00^ | ^100,00^ | ^AA^ | ^placebo^ | ^BS^ | ^TMS repetitive^ | ^PANSS^ | ^12,0^ | ^9,09^ |
| ^van Berckel 1999^ | ^13^ | ^13^ | ^SCZ^ | ^44,00^ | ^45,00^ | ^92,00^ | ^77,00^ | ^AA^ | ^placebo^ | ^OPA^ | ^D-cycloserine^ | ^PANSS^ | ^8,0^ | ^3,80^ |
| ^Voineskos 2021^ | ^37^ | ^33^ | ^SCZ^ | ^37,98^ | ^37,05^ | ^70,00^ | ^73,00^ | ^ns^ | ^placebo^ | ^BS^ | ^TMS repetitive^ | ^PANSS^ | ^4,0^ | ^13,58^ |
| ^Wang 2024^ | ^46^ | ^44^ | ^SCZ^ | ^27,00^ | ^27,00^ | ^51,00^ | ^51,00^ | ^SA^ | ^placebo^ | ^OPA^ | ^anti inflammatory any^ | ^PANSS^ | ^12,0^ | ^11,11^ |
| ^Weickert 2024^ | ^14^ | ^13^ | ^SCZ^ | ^37,70^ | ^39,80^ | ^46,00^ | ^64,00^ | ^AA^ | ^placebo^ | ^OPA^ | ^other pharma^ | ^PANSS^ | ^8,0^ | ^0,00^ |
| ^Xue 2024a^ | ^35^ | ^36^ | ^SCZ^ | ^48,67^ | ^45,50^ | ^83,00^ | ^85,00^ | ^AA^ | ^placebo^ | ^BS^ | ^TMS repetitive^ | ^PANSS^ | ^4,0^ | ^29,58^ |
| ^Yamanushi 2024^ | ^15^ | ^15^ | ^SCZ^ | ^48,46^ | ^46,57^ | ^62,00^ | ^64,00^ | ^mixed^ | ^none^ | ^PSI^ | ^computer assisted cognitive rehabilitation^ | ^PANSS^ | ^12,0^ | ^10,00^ |
| ^Yang 2022^ | ^21^ | ^19^ | ^SCZ^ | ^55,40^ | ^51,90^ | ^75,00^ | ^78,00^ | ^AA^ | ^placebo^ | ^OPA^ | ^N-acetyl cysteine^ | ^PANSS^ | ^8,0^ | ^15,00^ |
| ^Yeh 2024^ | ^30^ | ^30^ | ^SCZ^ | ^42,69^ | ^44,03^ | ^55,00^ | ^43,00^ | ^AA^ | ^placebo^ | ^BS^ | ^TCS direct^ | ^PANSS^ | ^5,0^ | ^1,67^ |
| ^Zarghami 2024^ | ^25^ | ^24^ | ^SCZ^ | ^36,57^ | ^37,71^ | ^79,00^ | ^84,00^ | ^SA^ | ^placebo^ | ^OPA^ | ^celecoxib^ | ^PANSS^ | ^5,0^ | ^6,10^ |
| ^Zhai 2023^ | ^16^ | ^16^ | ^SCZ^ | ^35,25^ | ^35,63^ | ^44,00^ | ^56,00^ | ^AA^ | ^placebo^ | ^BS^ | ^other stimulation^ | ^PANSS^ | ^3,0^ | ^18,75^ |
| ^Zhou 2023^ | ^21^ | ^17^ | ^SCZ^ | ^54,65^ | ^56,71^ | ^76,00^ | ^71,00^ | ^AA^ | ^placebo^ | ^BS^ | ^TCS direct^ | ^SANS^ | ^5,0^ | ^0,00^ |
| ^Zhou 2024^ | ^59^ | ^69^ | ^SCZ^ | ^46,70^ | ^48,40^ | ^61,00^ | ^49,00^ | ^AA^ | ^placebo^ | ^BS^ | ^TMS repetitive^ | ^PANSS^ | ^24,0^ | ^0,00^ |
| ^Byrne 2013^ | ^24^ | ^27^ | ^SCZ^ | ^47,71^ | ^45,14^ | ^100,00^ | ^100,00^ | ^AA^ | ^none^ | ^PSI^ | ^computer assisted cognitive rehabilitation^ | ^PANSS^ | ^6^ | ^39,22^ |
| ^Fan 2017b^ | ^11^ | ^12^ | ^SCZ^ | ^41,45^ | ^39,67^ | ^45,45^ | ^58,33^ | ^AA^ | ^none^ | ^PSI^ | ^computer assisted cognitive rehabilitation^ | ^PANSS^ | ^8^ | ^0.00^ |
| ^Kanie 2019^ | ^36^ | ^36^ | ^SCZ^ | ^37,50^ | ^35,50^ | ^53,13^ | ^62,50^ | ^mixed^ | ^none^ | ^PSI^ | ^group therapy social skills^ | ^PANSS^ | ^24^ | ^15,28^ |
| ^Maroño Souto 2018^ | ^31^ | ^30^ | ^SCZ^ | ^39,87^ | ^38,47^ | ^76,70^ | ^80,00^ | ^mixed^ | ^none^ | ^PSI^ | ^Emotional affect recognition^ | ^PANSS^ | ^12^ | ^1,66^ |
| ^McGurk 2016^ | ^26^ | ^28^ | ^SCZ^ | ^39,04^ | ^36,43^ | ^64,40^ | ^75,00^ | ^PSI^ | ^none^ | ^PSI^ | ^computer assisted cognitive rehabilitation^ | ^PANSS^ | ^72^ | ^35,19^ |
| ^O'Reilly 2019^ | ^33^ | ^32^ | ^SCZ^ | ^39,30^ | ^42,68^ | ^81,81^ | ^87,50^ | ^mixed^ | ^none^ | ^PSI^ | ^cognitive remediation^ | ^PANSS^ | ^32^ | ^0,00^ |
| **^Other studies^** | | | | | | | | | | | | | | |
| ^Abaoglu 2020^ | ^15^ | ^17^ | ^SCZ^ | ^38,89^ | ^40,33^ | ^65,00^ | ^53,00^ | ^mixed^ | ^WL^ | ^PSI^ | ^psychoeducational medication management training^ | ^PANSS^ | ^8,0^ | ^15,79^ |
| ^Acil 2008^ | ^15^ | ^15^ | ^SCZ^ | ^32,66^ | ^32,06^ | ^60,00^ | ^60,00^ | ^AA^ | ^none^ | ^LS^ | ^physical exercise^ | ^SANS^ | ^10,0^ | ^0,00^ |
| ^Akhondzadeh 2008^ | ^15^ | ^15^ | ^SCZ^ | ^33,86^ | ^32,33^ | ^67,00^ | ^60,00^ | ^SA^ | ^placebo^ | ^OPA^ | ^anticholinesterase^ | ^PANSS^ | ^12,0^ | ^0,00^ |
| ^Akhondzadeh 2009a^ | ^20^ | ^20^ | ^SCZ^ | ^33,05^ | ^32,05^ | ^60,00^ | ^65,00^ | ^SA^ | ^placebo^ | ^OPA^ | ^other pharma^ | ^PANSS^ | ^8,0^ | ^52,99^ |
| ^Akhondzadeh 2009b^ | ^15^ | ^15^ | ^SCZ^ | ^33,53^ | ^33,00^ | ^60,00^ | ^67,00^ | ^SA^ | ^placebo^ | ^OPA^ | ^antiemetic^ | ^PANSS^ | ^12,0^ | ^65,12^ |
| ^Arango 2000^ | ^16^ | ^16^ | ^SCZ^ | ^37,40^ | ^35,80^ | ^69,00^ | ^75,00^ | ^AA^ | ^placebo^ | ^OPA^ | ^fluoxetine^ | ^SANS^ | ^8,0^ | ^0,00^ |
| ^Arato 2002^ | ^67^ | ^24^ | ^SCZ^ | ^48,70^ | ^49,60^ | ^83,00^ | ^66,00^ | ^none^ | ^placebo^ | ^AP^ | ^ziprasidone^ | ^PANSS^ | ^48,0^ | ^64,38^ |
| ^Arato 2002^ | ^68^ | ^24^ | ^SCZ^ | ^48,70^ | ^49,80^ | ^83,00^ | ^71,00^ | ^none^ | ^placebo^ | ^AP^ | ^ziprasidone^ | ^PANSS^ | ^48,0^ | ^64,38^ |
| ^Arato 2002^ | ^72^ | ^24^ | ^SCZ^ | ^48,70^ | ^50,80^ | ^83,00^ | ^72,00^ | ^none^ | ^placebo^ | ^AP^ | ^ziprasidone^ | ^PANSS^ | ^48,0^ | ^64,38^ |
| ^Arvanitis 1997^ | ^52^ | ^9^ | ^SCZ^ | ^36,00^ | ^37,00^ | ^80,00^ | ^81,00^ | ^none^ | ^placebo^ | ^AP^ | ^haloperidol^ | ^SANS^ | ^6,0^ | ^58,40^ |
| ^Arvanitis 1997^ | ^53^ | ^9^ | ^SCZ^ | ^36,00^ | ^37,00^ | ^80,00^ | ^74,00^ | ^none^ | ^placebo^ | ^AP^ | ^quetiapine^ | ^SANS^ | ^6,0^ | ^58,40^ |
| ^Arvanitis 1997^ | ^48^ | ^9^ | ^SCZ^ | ^36,00^ | ^38,00^ | ^80,00^ | ^81,00^ | ^none^ | ^placebo^ | ^AP^ | ^quetiapine^ | ^SANS^ | ^6,0^ | ^58,40^ |
| ^Arvanitis 1997^ | ^52^ | ^9^ | ^SCZ^ | ^36,00^ | ^38,00^ | ^80,00^ | ^71,00^ | ^none^ | ^placebo^ | ^AP^ | ^quetiapine^ | ^SANS^ | ^6,0^ | ^58,40^ |
| ^Arvanitis 1997^ | ^51^ | ^9^ | ^SCZ^ | ^36,00^ | ^39,00^ | ^80,00^ | ^74,00^ | ^none^ | ^placebo^ | ^AP^ | ^quetiapine^ | ^SANS^ | ^6,0^ | ^58,40^ |
| ^Arvanitis 1997^ | ^54^ | ^9^ | ^SCZ^ | ^36,00^ | ^35,00^ | ^80,00^ | ^70,00^ | ^none^ | ^placebo^ | ^AP^ | ^quetiapine^ | ^SANS^ | ^6,0^ | ^58,40^ |
| ^Bark 2003^ | ^36^ | ^18^ | ^SCZ^ | ^38,55^ | ^35,00^ | ^44,00^ | ^67,00^ | ^AA^ | ^WL^ | ^LS^ | ^physical exercise^ | ^PANSS^ | ^8,0^ | ^0,00^ |
| ^Barzegar 2016^ | ^15^ | ^15^ | ^SCZ^ | ^NA^ | ^NA^ | ^ns^ | ^ns^ | ^ns^ | ^WL^ | ^PSI^ | ^group therapy social skills^ | ^SANS^ | ^8,0^ | ^0,00^ |
| ^Beasley 1996^ | ^65^ | ^17^ | ^SCZ^ | ^35,00^ | ^36,00^ | ^91,00^ | ^92,00^ | ^none^ | ^placebo^ | ^AP^ | ^olanzapine^ | ^BPRS neg^ | ^6,0^ | ^11,84^ |
| ^Beasley 1996^ | ^64^ | ^17^ | ^SCZ^ | ^35,00^ | ^37,00^ | ^91,00^ | ^88,00^ | ^none^ | ^placebo^ | ^AP^ | ^olanzapine^ | ^BPRS neg^ | ^6,0^ | ^11,84^ |
| ^Beasley 1996^ | ^69^ | ^17^ | ^SCZ^ | ^35,00^ | ^36,00^ | ^91,00^ | ^78,00^ | ^none^ | ^placebo^ | ^AP^ | ^olanzapine^ | ^BPRS neg^ | ^6,0^ | ^11,84^ |
| ^Beasley 1996^ | ^69^ | ^17^ | ^SCZ^ | ^35,00^ | ^36,00^ | ^91,00^ | ^90,00^ | ^none^ | ^placebo^ | ^AP^ | ^haloperidol^ | ^BPRS neg^ | ^6,0^ | ^11,84^ |
| ^Behere 2011^ | ^27^ | ^11^ | ^SCZ^ | ^33,60^ | ^31,30^ | ^68,00^ | ^67,00^ | ^AA^ | ^WL^ | ^PSI^ | ^yoga^ | ^PANSS^ | ^16,0^ | ^27,47^ |
| ^Behere 2011^ | ^17^ | ^11^ | ^SCZ^ | ^33,60^ | ^30,20^ | ^68,00^ | ^82,00^ | ^AA^ | ^WL^ | ^LS^ | ^physical exercise^ | ^PANSS^ | ^16,0^ | ^27,47^ |
| ^Berk 2009^ | ^20^ | ^20^ | ^SCZ^ | ^35,9^ | ^37,8^ | ^90,00^ | ^78,00^ | ^AA^ | ^placebo^ | ^OPA^ | ^mirtazapine^ | ^PANSS^ | ^6,0^ | ^5,00^ |
| ^Bio 2011^ | ^57^ | ^55^ | ^SCZ^ | ^30,80^ | ^28,20^ | ^84,00^ | ^74,00^ | ^none^ | ^WL^ | ^PSI^ | ^other PSI^ | ^PANSS^ | ^24,0^ | ^18,75^ |
| ^Borison 1996^ | ^55^ | ^54^ | ^SCZ^ | ^37,00^ | ^36,00^ | ^91,00^ | ^89,00^ | ^none^ | ^placebo^ | ^AP^ | ^other antipsychotic^ | ^SANS^ | ^6,0^ | ^56,82^ |
| ^Borras 2009^ | ^28^ | ^26^ | ^SCZ^ | ^41,00^ | ^41,00^ | ^38,00^ | ^38,00^ | ^mixed^ | ^WL^ | ^PSI^ | ^other PSI^ | ^PANSS^ | ^12,0^ | ^20,37^ |
| ^Boustani 2018^ | ^20^ | ^20^ | ^SCZ^ | ^33,60^ | ^33,85^ | ^65,00^ | ^65,00^ | ^AA^ | ^placebo^ | ^OPA^ | ^buspirone^ | ^PANSS^ | ^6,0^ | ^0,00^ |
| ^Breier 2018^ | ^30^ | ^30^ | ^SCZ^ | ^25,00^ | ^22,20^ | ^80,00^ | ^77,00^ | ^AA^ | ^placebo^ | ^OPA^ | ^N-acetyl cysteine^ | ^PANSS^ | ^52,0^ | ^46,67^ |
| ^Bugarski-Kirola 2014^ | ^80^ | ^27^ | ^SCZ^ | ^37,80^ | ^40,00^ | ^72,00^ | ^79,00^ | ^OPA^ | ^placebo^ | ^OPA^ | ^bitopertin^ | ^PANSS^ | ^10,0^ | ^27,09^ |
| ^Bugarski-Kirola 2014^ | ^77^ | ^27^ | ^SCZ^ | ^37,80^ | ^41,10^ | ^72,00^ | ^71,00^ | ^OPA^ | ^placebo^ | ^OPA^ | ^bitopertin^ | ^PANSS^ | ^10,0^ | ^27,09^ |
| ^Bugarski-Kirola 2014^ | ^72^ | ^27^ | ^SCZ^ | ^37,80^ | ^40,30^ | ^72,00^ | ^74,00^ | ^OPA^ | ^placebo^ | ^AP^ | ^olanzapine^ | ^PANSS^ | ^10,0^ | ^27,09^ |
| ^Caforio 2013^ | ^14^ | ^14^ | ^SCZ^ | ^27,80^ | ^30,70^ | ^86,00^ | ^64,00^ | ^AA^ | ^placebo^ | ^OPA^ | ^mirtazapine^ | ^PANSS^ | ^16,0^ | ^28,57^ |
| ^Cai 2014^ | ^15^ | ^15^ | ^SCZ^ | ^NA^ | ^NA^ | ^53,00^ | ^53,00^ | ^AA^ | ^none^ | ^PSI^ | ^other PSI^ | ^PANSS^ | ^5,0^ | ^0,00^ |
| ^Casey 2008^ | ^120^ | ^30^ | ^SCZ^ | ^40,80^ | ^41,10^ | ^77,00^ | ^81,00^ | ^OPA^ | ^placebo^ | ^AP^ | ^risperidone^ | ^PANSS^ | ^6,0^ | ^56,00^ |
| ^Casey 2008^ | ^115^ | ^30^ | ^SCZ^ | ^40,80^ | ^40,70^ | ^77,00^ | ^75,00^ | ^OPA^ | ^placebo^ | ^AP^ | ^other antipsychotic^ | ^PANSS^ | ^6,0^ | ^56,00^ |
| ^Casey 2008^ | ^120^ | ^30^ | ^SCZ^ | ^40,80^ | ^40,40^ | ^77,00^ | ^66,00^ | ^OPA^ | ^placebo^ | ^AP^ | ^other antipsychotic^ | ^PANSS^ | ^6,0^ | ^56,00^ |
| ^Casey 2008^ | ^115^ | ^30^ | ^SCZ^ | ^40,80^ | ^40,80^ | ^77,00^ | ^77,00^ | ^OPA^ | ^placebo^ | ^AP^ | ^other antipsychotic^ | ^PANSS^ | ^6,0^ | ^56,00^ |
| ^Chang 2008^ | ^30^ | ^32^ | ^SCZ^ | ^31,70^ | ^33,20^ | ^81,00^ | ^76,00^ | ^SA^ | ^placebo^ | ^AP^ | ^aripiprazole^ | ^SANS^ | ^8,0^ | ^9,68^ |
| ^Chaudhry 2012 1^ | ^15^ | ^15^ | ^SCZ^ | ^26,59^ | ^25,87^ | ^62,00^ | ^58,00^ | ^AA^ | ^placebo^ | ^OPA^ | ^minocycline^ | ^PANSS^ | ^48,0^ | ^34,72^ |
| ^Chaudhry 2012 2^ | ^56^ | ^58^ | ^SCZ^ | ^26,59^ | ^25,87^ | ^62,00^ | ^58,00^ | ^AA^ | ^placebo^ | ^OPA^ | ^minocycline^ | ^PANSS^ | ^48,0^ | ^34,72^ |
| ^Chaudhry 2014^ | ^12^ | ^6^ | ^SCZ^ | ^NA^ | ^NA^ | ^ns^ | ^ns^ | ^ns^ | ^placebo^ | ^OPA^ | ^simvastatin^ | ^PANSS^ | ^12,0^ | ^5,56^ |
| ^Chaudhry 2014^ | ^12^ | ^6^ | ^SCZ^ | ^NA^ | ^NA^ | ^ns^ | ^ns^ | ^ns^ | ^placebo^ | ^OPA^ | ^pharma antiemetic^ | ^PANSS^ | ^12,0^ | ^5,56^ |
| ^Chen 2008^ | ^96^ | ^98^ | ^SCZ^ | ^32,50^ | ^33,40^ | ^53,00^ | ^48,00^ | ^SA^ | ^placebo^ | ^OPA^ | ^other nutraceutical^ | ^PANSS^ | ^8,0^ | ^3,00^ |
| ^Chen 2009^ | ^60^ | ^60^ | ^SCZ^ | ^33,00^ | ^35,80^ | ^50,00^ | ^53,00^ | ^SA^ | ^placebo^ | ^OPA^ | ^other nutraceutical^ | ^PANSS^ | ^8,0^ | ^3,33^ |
| ^Cohen 1987^ | ^26^ | ^11^ | ^SCZ^ | ^36,10^ | ^36,10^ | ^ns^ | ^ns^ | ^none^ | ^placebo^ | ^AP^ | ^other antipsychotic^ | ^BPRS neg^ | ^2,0^ | ^31,48^ |
| ^Cutler 2008^ | ^149^ | ^75^ | ^SCZ^ | ^40,70^ | ^40,00^ | ^76,00^ | ^76,00^ | ^none^ | ^placebo^ | ^AP^ | ^ziprasidone^ | ^PANSS^ | ^4,0^ | ^37,13^ |
| ^Cutler 2008^ | ^295^ | ^75^ | ^SCZ^ | ^40,70^ | ^39,50^ | ^76,00^ | ^83,00^ | ^none^ | ^placebo^ | ^AP^ | ^other antipsychotic^ | ^PANSS^ | ^4,0^ | ^37,13^ |
| ^Dai 2022^ | ^26^ | ^16^ | ^SCZ^ | ^44,06^ | ^41,50^ | ^65,00^ | ^85,00^ | ^AA^ | ^none^ | ^LS^ | ^physical exercise^ | ^PANSS^ | ^8,0^ | ^ns^ |
| ^Dai 2022^ | ^25^ | ^16^ | ^SCZ^ | ^44,06^ | ^41,40^ | ^65,00^ | ^80,00^ | ^AA^ | ^none^ | ^LS^ | ^physical exercise^ | ^PANSS^ | ^8,0^ | ^ns^ |
| ^Daniels 1998^ | ^20^ | ^20^ | ^SCZ^ | ^33,70^ | ^33,70^ | ^68,00^ | ^68,00^ | ^ns^ | ^WL^ | ^PSI^ | ^group therapy integrated psychological^ | ^SANS^ | ^8,0^ | ^ns^ |
| ^Davidson 2007^ | ^123^ | ^30^ | ^SCZ^ | ^37,30^ | ^36,30^ | ^69,00^ | ^63,00^ | ^OPA^ | ^placebo^ | ^AP^ | ^paliperidone^ | ^PANSS^ | ^6,0^ | ^41,00^ |
| ^Davidson 2007^ | ^123^ | ^30^ | ^SCZ^ | ^37,30^ | ^36,20^ | ^69,00^ | ^64,00^ | ^OPA^ | ^placebo^ | ^AP^ | ^paliperidone^ | ^PANSS^ | ^6,0^ | ^41,00^ |
| ^Davidson 2007^ | ^113^ | ^30^ | ^SCZ^ | ^37,30^ | ^37,60^ | ^69,00^ | ^65,00^ | ^OPA^ | ^placebo^ | ^AP^ | ^paliperidone^ | ^PANSS^ | ^6,0^ | ^41,00^ |
| ^Davidson 2007^ | ^126^ | ^30^ | ^SCZ^ | ^37,30^ | ^36,80^ | ^69,00^ | ^68,00^ | ^OPA^ | ^placebo^ | ^AP^ | ^olanzapine^ | ^PANSS^ | ^6,0^ | ^41,00^ |
| ^De Lima 2023^ | ^17^ | ^18^ | ^SCZ^ | ^37,11^ | ^38,47^ | ^72,00^ | ^59,00^ | ^AA^ | ^placebo^ | ^OPA^ | ^fatty acid^ | ^BPRS neg^ | ^16,0^ | ^5,71^ |
| ^Den Boer 2000^ | ^82^ | ^78^ | ^SCZ^ | ^39,00^ | ^37,00^ | ^77,00^ | ^82,00^ | ^AA^ | ^placebo^ | ^OPA^ | ^other antidepressant^ | ^PANSS^ | ^8,0^ | ^1,87^ |
| ^Dickerson 2008^ | ^24^ | ^23^ | ^SCZ^ | ^46,90^ | ^46,90^ | ^43,00^ | ^43,00^ | ^SA^ | ^placebo^ | ^OPA^ | ^other pharma^ | ^PANSS^ | ^16,0^ | ^17,00^ |
| ^Doruk 2008^ | ^23^ | ^23^ | ^SCZ^ | ^31,90^ | ^29,70^ | ^73,00^ | ^55,00^ | ^SA^ | ^placebo^ | ^OPA^ | ^ginkgo biloba^ | ^SANS^ | ^12,0^ | ^8,70^ |
| ^Evins 2000^ | ^14^ | ^13^ | ^SCZ^ | ^NA^ | ^NA^ | ^78,00^ | ^78,00^ | ^SA^ | ^placebo^ | ^OPA^ | ^other glutamatergic^ | ^PANSS^ | ^8,0^ | ^5,00^ |
| ^Fan 2013^ | ^21^ | ^24^ | ^SCZ^ | ^43,80^ | ^49,20^ | ^80,00^ | ^80,00^ | ^AA^ | ^placebo^ | ^OPA^ | ^other hormone^ | ^PANSS^ | ^8,0^ | ^0,00^ |
| ^Findling 2012^ | ^73^ | ^37^ | ^SCZ^ | ^15,41^ | ^14,45^ | ^58,00^ | ^59,00^ | ^OPA^ | ^placebo^ | ^AP^ | ^quetiapine^ | ^PANSS^ | ^6,0^ | ^26,36^ |
| ^Findling 2012^ | ^74^ | ^37^ | ^SCZ^ | ^15,41^ | ^15,45^ | ^58,00^ | ^60,00^ | ^OPA^ | ^placebo^ | ^AP^ | ^quetiapine^ | ^PANSS^ | ^6,0^ | ^26,36^ |
| ^Gao 2021^ | ^20^ | ^20^ | ^SCZ^ | ^53,40^ | ^53,00^ | ^100,00^ | ^100,00^ | ^AA^ | ^none^ | ^LS^ | ^physical exercise^ | ^PANSS^ | ^12,0^ | ^0,00^ |
| ^Garety 2006^ | ^71^ | ^73^ | ^SCZ^ | ^26,60^ | ^26,00^ | ^74,00^ | ^55,00^ | ^ns^ | ^none^ | ^PSI^ | ^group therapy integrated psychological^ | ^PANSS^ | ^72,0^ | ^5,55^ |
| ^Garety 2008 1^ | ^106^ | ^112^ | ^SCZ^ | ^37,10^ | ^39,10^ | ^69,00^ | ^70,00^ | ^ns^ | ^none^ | ^PSI^ | ^CBT cognitive behavioral therapy^ | ^PANSS^ | ^104,0^ | ^17,43^ |
| ^Garety 2008 2^ | ^27^ | ^14^ | ^SCZ^ | ^35,60^ | ^38,60^ | ^68,00^ | ^78,00^ | ^ns^ | ^none^ | ^PSI^ | ^CBT cognitive behavioral therapy^ | ^PANSS^ | ^104,0^ | ^18,07^ |
| ^Garety 2008 2^ | ^28^ | ^14^ | ^SCZ^ | ^35,60^ | ^35,00^ | ^68,00^ | ^71,00^ | ^ns^ | ^none^ | ^PSI^ | ^other PSI^ | ^PANSS^ | ^104,0^ | ^18,07^ |
| ^Ghanizadeh 2014^ | ^20^ | ^16^ | ^SCZ^ | ^30,10^ | ^30,80^ | ^69,00^ | ^68,00^ | ^AA^ | ^placebo^ | ^OPA^ | ^other statin^ | ^PANSS^ | ^8,0^ | ^33,33^ |
| ^Gholipour 2012^ | ^15^ | ^8^ | ^SCZ^ | ^41,00^ | ^43,00^ | ^100,00^ | ^100,00^ | ^ns^ | ^none^ | ^PSI^ | ^other PSI^ | ^SANS^ | ^12,0^ | ^0,00^ |
| ^Gholipour 2012^ | ^15^ | ^8^ | ^SCZ^ | ^41,00^ | ^38,00^ | ^100,00^ | ^100,00^ | ^ns^ | ^none^ | ^LS^ | ^physical exercise^ | ^SANS^ | ^12,0^ | ^0,00^ |
| ^Goff 1995^ | ^20^ | ^21^ | ^SCZ^ | ^42,80^ | ^42,20^ | ^95,00^ | ^90,00^ | ^AA^ | ^placebo^ | ^OPA^ | ^fluoxetine^ | ^BPRS neg^ | ^6,0^ | ^4,65^ |
| ^Goff 1999^ | ^23^ | ^24^ | ^SCZ^ | ^41,20^ | ^46,80^ | ^83,00^ | ^65,00^ | ^AA^ | ^placebo^ | ^OPA^ | ^D-cycloserine^ | ^SANS^ | ^8,0^ | ^2,08^ |
| ^Goff 2005^ | ^27^ | ^28^ | ^SCZ^ | ^47,00^ | ^45,90^ | ^71,00^ | ^86,00^ | ^AA^ | ^placebo^ | ^OPA^ | ^D-cycloserine^ | ^PANSS^ | ^8,0^ | ^52,64^ |
| ^Goff 2019^ | ^49^ | ^46^ | ^SCZ^ | ^23,69^ | ^23,20^ | ^61,00^ | ^65,00^ | ^AA^ | ^placebo^ | ^OPA^ | ^citalopram^ | ^SANS^ | ^52,0^ | ^45,26^ |
| ^Govindaraj 2021^ | ^26^ | ^25^ | ^SCZ^ | ^32,92^ | ^33,62^ | ^76,00^ | ^58,00^ | ^AA^ | ^WL^ | ^PSI^ | ^yoga^ | ^SANS^ | ^6,0^ | ^9,80^ |
| ^Granholm 2005^ | ^39^ | ^37^ | ^SCZ^ | ^53,10^ | ^54,50^ | ^77,00^ | ^70,00^ | ^mixed^ | ^none^ | ^PSI^ | ^group therapy social skills^ | ^PANSS^ | ^24,0^ | ^14,47^ |
| ^Gu 2018^ | ^41^ | ^40^ | ^SCZ^ | ^57,40^ | ^56,07^ | ^ns^ | ^ns^ | ^AA^ | ^none^ | ^AP^ | ^paliperidone^ | ^PANSS^ | ^52,0^ | ^0,00^ |
| ^Gumley 2003^ | ^72^ | ^72^ | ^SCZ^ | ^36,70^ | ^35,80^ | ^71,00^ | ^75,00^ | ^mixed^ | ^none^ | ^PSI^ | ^CBT cognitive behavioral therapy^ | ^PANSS^ | ^52,0^ | ^7,64^ |
| ^Haas 2009^ | ^55^ | ^27^ | ^SCZ^ | ^15,50^ | ^15,70^ | ^65,00^ | ^55,00^ | ^OPA^ | ^placebo^ | ^AP^ | ^risperidone^ | ^PANSS^ | ^6,0^ | ^21,88^ |
| ^Haas 2009^ | ^51^ | ^27^ | ^SCZ^ | ^15,50^ | ^15,70^ | ^65,00^ | ^73,00^ | ^OPA^ | ^placebo^ | ^AP^ | ^risperidone^ | ^PANSS^ | ^6,0^ | ^21,88^ |
| ^Hajak 2004^ | ^10^ | ^10^ | ^SCZ^ | ^41,70^ | ^37,90^ | ^60,00^ | ^80,00^ | ^AA^ | ^placebo^ | ^BS^ | ^TMS repetitive^ | ^PANSS^ | ^1,4^ | ^0,00^ |
| ^Hayashi 1997^ | ^13^ | ^7^ | ^SCZ^ | ^59,90^ | ^64,60^ | ^54,00^ | ^62,00^ | ^AA^ | ^placebo^ | ^OPA^ | ^other antidepressant^ | ^SANS^ | ^5,0^ | ^0,00^ |
| ^Hayashi 1997^ | ^13^ | ^7^ | ^SCZ^ | ^59,90^ | ^63,70^ | ^54,00^ | ^62,00^ | ^AA^ | ^placebo^ | ^OPA^ | ^other antidepressant^ | ^SANS^ | ^5,0^ | ^0,00^ |
| ^Hayes 1995^ | ^34^ | ^34^ | ^SCZ^ | ^36,00^ | ^36,00^ | ^75,00^ | ^75,00^ | ^ns^ | ^none^ | ^PSI^ | ^group therapy social skills^ | ^SANS^ | ^36,0^ | ^29,00^ |
| ^Hill 2011^ | ^14^ | ^14^ | ^SCZ^ | ^46,50^ | ^45,50^ | ^87,00^ | ^76,00^ | ^ns^ | ^placebo^ | ^OPA^ | ^other nutraceutical^ | ^SANS^ | ^12,0^ | ^6,27^ |
| ^Hinkelmann 2013^ | ^19^ | ^8^ | ^SCZ^ | ^38,30^ | ^42,10^ | ^62,00^ | ^79,00^ | ^AA^ | ^placebo^ | ^OPA^ | ^reboxetine^ | ^PANSS^ | ^4,0^ | ^ns^ |
| ^Hinkelmann 2013^ | ^16^ | ^8^ | ^SCZ^ | ^38,30^ | ^38,50^ | ^62,00^ | ^56,00^ | ^AA^ | ^placebo^ | ^OPA^ | ^citalopram^ | ^PANSS^ | ^4,0^ | ^ns^ |
| ^Hirayasu 2017^ | ^10^ | ^10^ | ^SCZ^ | ^38,00^ | ^43,00^ | ^70,00^ | ^60,00^ | ^CA^ | ^placebo^ | ^OPA^ | ^bitopertin^ | ^PANSS^ | ^8,0^ | ^ns^ |
| ^Honer 2006^ | ^32^ | ^33^ | ^SCZ^ | ^34,90^ | ^39,40^ | ^74,00^ | ^74,00^ | ^SA^ | ^placebo^ | ^AP^ | ^risperidone^ | ^PANSS^ | ^8,0^ | ^4,41^ |
| ^Ishoy 2017^ | ^23^ | ^22^ | ^SCZ^ | ^34,50^ | ^37,10^ | ^45,00^ | ^48,00^ | ^AA^ | ^placebo^ | ^OPA^ | ^other pharma^ | ^PANSS^ | ^12,0^ | ^11,11^ |
| ^Jamilian 2014^ | ^30^ | ^30^ | ^SCZ^ | ^31,01^ | ^32,01^ | ^50,00^ | ^53,00^ | ^AA^ | ^placebo^ | ^OPA^ | ^omega 3^ | ^PANSS^ | ^8,0^ | ^0,00^ |
| ^Jarskog 2017^ | ^35^ | ^33^ | ^SCZ^ | ^36,10^ | ^41,90^ | ^77,00^ | ^75,00^ | ^ns^ | ^placebo^ | ^OPA^ | ^oxytocin^ | ^PANSS^ | ^12,0^ | ^17,58^ |
| ^Javadi 2018^ | ^34^ | ^34^ | ^SCZ^ | ^42,88^ | ^40,91^ | ^76,00^ | ^53,00^ | ^SA^ | ^none^ | ^OPA^ | ^fluvoxamine^ | ^SANS^ | ^10,0^ | ^0,00^ |
| ^Jayaram 2013^ | ^15^ | ^28^ | ^SCZ^ | ^29,50^ | ^28,33^ | ^58,00^ | ^80,00^ | ^ns^ | ^WL^ | ^PSI^ | ^yoga^ | ^SANS^ | ^6,0^ | ^36,79^ |
| ^Joffe 2009^ | ^20^ | ^21^ | ^SCZ^ | ^48,21^ | ^43,40^ | ^47,00^ | ^55,00^ | ^AA^ | ^placebo^ | ^OPA^ | ^mirtazapine^ | ^PANSS^ | ^6,0^ | ^4,76^ |
| ^Kane 2002^ | ^104^ | ^35^ | ^SCZ^ | ^38,50^ | ^38,90^ | ^70,00^ | ^65,00^ | ^SA^ | ^placebo^ | ^AP^ | ^haloperidol^ | ^PANSS^ | ^4,0^ | ^40,00^ |
| ^Kane 2002^ | ^102^ | ^35^ | ^SCZ^ | ^38,50^ | ^37,80^ | ^70,00^ | ^75,00^ | ^SA^ | ^placebo^ | ^AP^ | ^aripiprazole^ | ^PANSS^ | ^4,0^ | ^40,00^ |
| ^Kane 2002^ | ^102^ | ^35^ | ^SCZ^ | ^38,50^ | ^39,30^ | ^70,00^ | ^69,00^ | ^SA^ | ^placebo^ | ^AP^ | ^aripiprazole^ | ^PANSS^ | ^4,0^ | ^40,00^ |
| ^Kane 2010a^ | ^15^ | ^5^ | ^SCZ^ | ^46,00^ | ^44,90^ | ^80,00^ | ^73,00^ | ^AA^ | ^placebo^ | ^OPA^ | ^armodafinil modafinil^ | ^PANSS^ | ^4,0^ | ^18,33^ |
| ^Kane 2010a^ | ^15^ | ^5^ | ^SCZ^ | ^46,00^ | ^40,40^ | ^80,00^ | ^67,00^ | ^AA^ | ^placebo^ | ^OPA^ | ^armodafinil modafinil^ | ^PANSS^ | ^4,0^ | ^18,33^ |
| ^Kane 2010a^ | ^15^ | ^5^ | ^SCZ^ | ^46,00^ | ^41,40^ | ^80,00^ | ^73,00^ | ^AA^ | ^placebo^ | ^OPA^ | ^armodafinil modafinil^ | ^PANSS^ | ^4,0^ | ^18,33^ |
| ^Kane 2010b^ | ^112^ | ^41^ | ^SCZ^ | ^NA^ | ^NA^ | ^ns^ | ^ns^ | ^none^ | ^placebo^ | ^AP^ | ^haloperidol^ | ^PANSS^ | ^6,0^ | ^40,61^ |
| ^Kane 2010b^ | ^109^ | ^41^ | ^SCZ^ | ^NA^ | ^NA^ | ^ns^ | ^ns^ | ^none^ | ^placebo^ | ^AP^ | ^other antipsychotic^ | ^PANSS^ | ^6,0^ | ^40,61^ |
| ^Kane 2010b^ | ^105^ | ^41^ | ^SCZ^ | ^NA^ | ^NA^ | ^ns^ | ^ns^ | ^none^ | ^placebo^ | ^AP^ | ^other antipsychotic^ | ^PANSS^ | ^6,0^ | ^40,61^ |
| ^Kane 2012^ | ^71^ | ^24^ | ^SCZ^ | ^42,40^ | ^43,70^ | ^64,00^ | ^75,00^ | ^AA^ | ^placebo^ | ^OPA^ | ^armodafinil modafinil^ | ^PANSS^ | ^24,0^ | ^38,25^ |
| ^Kane 2012^ | ^70^ | ^24^ | ^SCZ^ | ^42,40^ | ^43,10^ | ^64,00^ | ^81,00^ | ^AA^ | ^placebo^ | ^OPA^ | ^armodafinil modafinil^ | ^PANSS^ | ^24,0^ | ^38,25^ |
| ^Kane 2012^ | ^72^ | ^24^ | ^SCZ^ | ^42,40^ | ^44,40^ | ^64,00^ | ^69,00^ | ^AA^ | ^placebo^ | ^OPA^ | ^armodafinil modafinil^ | ^PANSS^ | ^24,0^ | ^38,25^ |
| ^Kantrowitz 2020^ | ^19^ | ^11^ | ^SCZ^ | ^35,60^ | ^35,60^ | ^83,00^ | ^83,00^ | ^AA^ | ^placebo^ | ^OPA^ | ^other pharma^ | ^SANS^ | ^3,0^ | ^20,83^ |
| ^Kantrowitz 2020^ | ^19^ | ^11^ | ^SCZ^ | ^35,60^ | ^35,60^ | ^83,00^ | ^83,00^ | ^AA^ | ^placebo^ | ^OPA^ | ^other pharma^ | ^SANS^ | ^3,0^ | ^20,83^ |
| ^Katsumi 2017^ | ^22^ | ^22^ | ^SCZ^ | ^38,00^ | ^37,50^ | ^59,00^ | ^59,00^ | ^mixed^ | ^none^ | ^PSI^ | ^cognitive remediation^ | ^PANSS^ | ^12,0^ | ^0,00^ |
| ^Keck 1998^ | ^44^ | ^24^ | ^SCZ^ | ^39,00^ | ^44,60^ | ^85,00^ | ^68,00^ | ^OPA^ | ^placebo^ | ^AP^ | ^ziprasidone^ | ^SANS^ | ^4,0^ | ^45,32^ |
| ^Keck 1998^ | ^47^ | ^24^ | ^SCZ^ | ^39,00^ | ^47,50^ | ^85,00^ | ^83,00^ | ^OPA^ | ^placebo^ | ^AP^ | ^ziprasidone^ | ^SANS^ | ^4,0^ | ^45,32^ |
| ^Kelly 2015^ | ^29^ | ^23^ | ^SCZ^ | ^42,3^ | ^42,9^ | ^78^ | ^71^ | ^SA^ | ^placebo^ | ^OPA^ | ^minocycline^ | ^SANS^ | ^10^ | ^3,45^ |
| ^Khonsari 2021^ | ^20^ | ^20^ | ^SCZ^ | ^37,20^ | ^32,70^ | ^45,00^ | ^60,00^ | ^SA^ | ^none^ | ^LS^ | ^physical exercise^ | ^PANSS^ | ^8,0^ | ^0,00^ |
| ^Kianimehr 2014^ | ^25^ | ^25^ | ^SCZ^ | ^60,44^ | ^61,96^ | ^100,00^ | ^100,00^ | ^SA^ | ^placebo^ | ^OPA^ | ^raloxifene^ | ^PANSS^ | ^8,0^ | ^8,00^ |
| ^Ko 2008^ | ^15^ | ^15^ | ^SCZ^ | ^37,00^ | ^36,40^ | ^100,00^ | ^100,00^ | ^mixed^ | ^placebo^ | ^OPA^ | ^other hormone^ | ^PANSS^ | ^6,0^ | ^43,30^ |
| ^Kopelowicz 2003^ | ^45^ | ^47^ | ^SCZ^ | ^39,10^ | ^37,60^ | ^68,00^ | ^67,00^ | ^AA^ | ^none^ | ^PSI^ | ^group therapy social skills^ | ^PANSS^ | ^39,1^ | ^8,70^ |
| ^Krakvik 2013^ | ^23^ | ^22^ | ^SCZ^ | ^37,50^ | ^35,26^ | ^64,00^ | ^65,00^ | ^AA^ | ^WL^ | ^PSI^ | ^CBT cognitive behavioral therapy^ | ^SANS^ | ^31,0^ | ^37,70^ |
| ^Lane 2005^ | ^21^ | ^12^ | ^SCZ^ | ^34,10^ | ^31,80^ | ^52,00^ | ^48,00^ | ^SA^ | ^placebo^ | ^OPA^ | ^D-serine^ | ^PANSS^ | ^6,0^ | ^12,30^ |
| ^Lane 2005^ | ^21^ | ^12^ | ^SCZ^ | ^34,10^ | ^36,10^ | ^52,00^ | ^67,00^ | ^SA^ | ^placebo^ | ^OPA^ | ^sarcosine^ | ^PANSS^ | ^6,0^ | ^12,30^ |
| ^Lane 2006^ | ^10^ | ^10^ | ^SCZ^ | ^35,50^ | ^36,70^ | ^70,00^ | ^70,00^ | ^SA^ | ^placebo^ | ^OPA^ | ^sarcosine^ | ^PANSS^ | ^6,0^ | ^0,00^ |
| ^Lauriello 2008^ | ^106^ | ^33^ | ^SCZ^ | ^42,60^ | ^39,80^ | ^62,00^ | ^74,00^ | ^CA^ | ^placebo^ | ^AP^ | ^olanzapine^ | ^PANSS^ | ^8,0^ | ^33,91^ |
| ^Lauriello 2008^ | ^100^ | ^33^ | ^SCZ^ | ^42,60^ | ^41,50^ | ^62,00^ | ^72,00^ | ^CA^ | ^placebo^ | ^AP^ | ^olanzapine^ | ^PANSS^ | ^8,0^ | ^33,91^ |
| ^Lauriello 2008^ | ^100^ | ^33^ | ^SCZ^ | ^42,60^ | ^39,50^ | ^62,00^ | ^73,00^ | ^CA^ | ^placebo^ | ^AP^ | ^olanzapine^ | ^PANSS^ | ^8,0^ | ^33,91^ |
| ^Le Moigne 2021^ | ^115^ | ^59^ | ^SCZ^ | ^NA^ | ^NA^ | ^ns^ | ^ns^ | ^none^ | ^placebo^ | ^AP^ | ^risperidone^ | ^PANSS NAA/NDE^ | ^8,0^ | ^26,80^ |
| ^Le Moigne 2021^ | ^117^ | ^59^ | ^SCZ^ | ^NA^ | ^NA^ | ^ns^ | ^ns^ | ^none^ | ^placebo^ | ^AP^ | ^risperidone^ | ^PANSS NAA/NDE^ | ^8,0^ | ^26,80^ |
| ^Lecrubier 2006^ | ^70^ | ^12^ | ^SCZ^ | ^38,20^ | ^38,10^ | ^65,00^ | ^60,00^ | ^OPA^ | ^placebo^ | ^AP^ | ^olanzapine^ | ^SANS^ | ^26,0^ | ^58,36^ |
| ^Lecrubier 2006^ | ^70^ | ^12^ | ^SCZ^ | ^38,20^ | ^36,40^ | ^65,00^ | ^74,00^ | ^OPA^ | ^placebo^ | ^AP^ | ^olanzapine^ | ^SANS^ | ^26,0^ | ^58,36^ |
| ^Lecrubier 2006^ | ^70^ | ^12^ | ^SCZ^ | ^38,20^ | ^37,80^ | ^65,00^ | ^71,00^ | ^OPA^ | ^placebo^ | ^AP^ | ^amisulpride^ | ^SANS^ | ^26,0^ | ^58,36^ |
| ^Lee 1998^ | ^18^ | ^18^ | ^SCZ^ | ^39,70^ | ^40,10^ | ^56,00^ | ^56,00^ | ^SA^ | ^placebo^ | ^OPA^ | ^other antidepressant^ | ^PANSS^ | ^8,0^ | ^0,00^ |
| ^Lee 2012^ | ^15^ | ^11^ | ^SCZ^ | ^43,40^ | ^44,30^ | ^46,00^ | ^73,00^ | ^mixed^ | ^placebo^ | ^OPA^ | ^memantine^ | ^PANSS^ | ^12,0^ | ^0,00^ |
| ^Lee 2013a^ | ^17^ | ^18^ | ^SCZ^ | ^50,50^ | ^51,00^ | ^72,00^ | ^76,00^ | ^mixed^ | ^placebo^ | ^AP^ | ^aripiprazole^ | ^PANSS^ | ^12,0^ | ^17,14^ |
| ^Lee 2015a^ | ^18^ | ^20^ | ^SCZ^ | ^41,80^ | ^41,50^ | ^50,00^ | ^44,00^ | ^AA^ | ^none^ | ^LS^ | ^dance movement therapy^ | ^PANSS^ | ^12,0^ | ^0,00^ |
| ^Lee 2019^ | ^30^ | ^30^ | ^SCZ_spectrum_unspecified^ | ^51,15^ | ^54,43^ | ^ns^ | ^ns^ | ^mixed^ | ^none^ | ^PSI^ | ^mindfulness^ | ^PANSS^ | ^12,0^ | ^16,00^ |
| ^Levkovitz 2009^ | ^36^ | ^18^ | ^SCZ^ | ^25,50^ | ^24,80^ | ^88,00^ | ^77,00^ | ^AA^ | ^placebo^ | ^OPA^ | ^minocycline^ | ^PANSS^ | ^24,0^ | ^61,11^ |
| ^Li 2016^ | ^80^ | ^80^ | ^SCZ^ | ^37,70^ | ^38,10^ | ^55,00^ | ^52,00^ | ^AA^ | ^none^ | ^BS^ | ^ECT^ | ^PANSS^ | ^3,0^ | ^0,00^ |
| ^Li 2019^ | ^16^ | ^15^ | ^SCZ^ | ^29,55^ | ^26,33^ | ^73,00^ | ^53,00^ | ^AA^ | ^none^ | ^PSI^ | ^cognitive remediation^ | ^PANSS^ | ^4,0^ | ^16,13^ |
| ^Li 2021^ | ^51^ | ^50^ | ^SCZ^ | ^31,20^ | ^30,50^ | ^34,00^ | ^41,00^ | ^ns^ | ^none^ | ^PSI^ | ^psychoeducational medication management training^ | ^PANSS^ | ^NA^ | ^0,00^ |
| ^Liebermann 2009^ | ^69^ | ^67^ | ^SCZ^ | ^40,10^ | ^40,90^ | ^79,00^ | ^59,00^ | ^mixed^ | ^placebo^ | ^OPA^ | ^memantine^ | ^PANSS^ | ^8,0^ | ^15,22^ |
| ^Litman 2014^ | ^22^ | ^21^ | ^SCZ^ | ^40,20^ | ^35,30^ | ^95,00^ | ^100,00^ | ^none^ | ^placebo^ | ^AP^ | ^olanzapine^ | ^PANSS^ | ^4,0^ | ^23,60^ |
| ^Litman 2014^ | ^43^ | ^21^ | ^SCZ^ | ^40,20^ | ^40,10^ | ^95,00^ | ^93,00^ | ^none^ | ^placebo^ | ^OPA^ | ^other pharma^ | ^PANSS^ | ^4,0^ | ^23,60^ |
| ^Lohr 2013^ | ^12^ | ^12^ | ^SCZ^ | ^48,50^ | ^47,80^ | ^100,00^ | ^100,00^ | ^mixed^ | ^placebo^ | ^OPA^ | ^armodafinil modafinil^ | ^PANSS^ | ^8,0^ | ^0,00^ |
| ^Loo 1997^ | ^69^ | ^72^ | ^SCZ^ | ^36,00^ | ^33,00^ | ^75,00^ | ^67,00^ | ^mixed^ | ^placebo^ | ^AP^ | ^amisulpride^ | ^SANS^ | ^26,0^ | ^44,90^ |
| ^Magliano 2006^ | ^42^ | ^29^ | ^SCZ^ | ^34,10^ | ^36,90^ | ^83,00^ | ^69,00^ | ^ns^ | ^WL^ | ^PSI^ | ^psychoeducational medication management training^ | ^BPRS neg^ | ^24,0^ | ^12,68^ |
| ^Marder 1994^ | ^63^ | ^13^ | ^SCZ^ | ^37,10^ | ^39,30^ | ^86,00^ | ^86,00^ | ^none^ | ^placebo^ | ^AP^ | ^risperidone^ | ^PANSS^ | ^8,0^ | ^53,00^ |
| ^Marder 1994^ | ^64^ | ^13^ | ^SCZ^ | ^37,10^ | ^37,50^ | ^86,00^ | ^86,00^ | ^none^ | ^placebo^ | ^AP^ | ^risperidone^ | ^PANSS^ | ^8,0^ | ^53,00^ |
| ^Marder 1994^ | ^65^ | ^13^ | ^SCZ^ | ^37,10^ | ^36,20^ | ^86,00^ | ^94,00^ | ^none^ | ^placebo^ | ^AP^ | ^risperidone^ | ^PANSS^ | ^8,0^ | ^53,00^ |
| ^Marder 1994^ | ^64^ | ^13^ | ^SCZ^ | ^37,10^ | ^36,50^ | ^86,00^ | ^83,00^ | ^none^ | ^placebo^ | ^AP^ | ^risperidone^ | ^PANSS^ | ^8,0^ | ^53,00^ |
| ^Marder 1994^ | ^64^ | ^13^ | ^SCZ^ | ^37,10^ | ^37,40^ | ^86,00^ | ^91,00^ | ^none^ | ^placebo^ | ^AP^ | ^haloperidol^ | ^PANSS^ | ^8,0^ | ^53,00^ |
| ^Markiewicz 2019^ | ^26^ | ^19^ | ^SCZ^ | ^NA^ | ^NA^ | ^ns^ | ^ns^ | ^AA^ | ^none^ | ^PSI^ | ^group therapy integrated psychological^ | ^PANSS^ | ^12,0^ | ^0,00^ |
| ^Markiewicz 2021^ | ^30^ | ^30^ | ^SCZ^ | ^36,38^ | ^37,22^ | ^100,00^ | ^100,00^ | ^mixed^ | ^none^ | ^PSI^ | ^cognitive remediation^ | ^PANSS^ | ^13,0^ | ^26,66^ |
| ^Marklewicz-Gospodarek 2022^ | ^16^ | ^17^ | ^SCZ^ | ^39,35^ | ^36,00^ | ^100,00^ | ^100,00^ | ^mixed^ | ^none^ | ^PSI^ | ^psychoeducational medication management training^ | ^PANSS^ | ^12,0^ | ^ns^ |
| ^Mendella 2015^ | ^16^ | ^11^ | ^SCZ_spectrum_unspecified^ | ^24,80^ | ^25,00^ | ^82,00^ | ^69,00^ | ^mixed^ | ^none^ | ^PSI^ | ^cognitive remediation^ | ^PANSS^ | ^12,0^ | ^0,00^ |
| ^Moller 2004^ | ^39^ | ^46^ | ^SCZ^ | ^42,20^ | ^39,80^ | ^51,00^ | ^55,00^ | ^mixed^ | ^placebo^ | ^AP^ | ^other antipsychotic^ | ^PANSS^ | ^8,0^ | ^16,47^ |
| ^Morozova 2012^ | ^29^ | ^27^ | ^SCZ^ | ^35,80^ | ^34,60^ | ^100,00^ | ^100,00^ | ^SA^ | ^placebo^ | ^OPA^ | ^pharma antihistamine^ | ^PANSS^ | ^8,0^ | ^26,79^ |
| ^Muscatello 2011^ | ^20^ | ^20^ | ^SCZ^ | ^30,70^ | ^31,90^ | ^53,00^ | ^57,00^ | ^SA^ | ^placebo^ | ^AP^ | ^aripiprazole^ | ^SANS^ | ^24,0^ | ^22,50^ |
| ^Nachshoni 1994^ | ^15^ | ^15^ | ^SCZ^ | ^45,00^ | ^48,10^ | ^57,00^ | ^50,00^ | ^mixed^ | ^placebo^ | ^OPA^ | ^other anticonvulsant^ | ^SANS^ | ^7,0^ | ^6,66^ |
| ^Noorbala 1999^ | ^14^ | ^16^ | ^SCZ^ | ^NA^ | ^NA^ | ^53,00^ | ^53,00^ | ^SA^ | ^placebo^ | ^OPA^ | ^other glutamatergic^ | ^PANSS^ | ^8,0^ | ^0,00^ |
| ^Omranifard 2017^ | ^32^ | ^32^ | ^SCZ^ | ^34,20^ | ^32,30^ | ^47,00^ | ^60,00^ | ^AA^ | ^placebo^ | ^OPA^ | ^memantine^ | ^PANSS^ | ^12,0^ | ^6,25^ |
| ^Paikkatt 2015^ | ^15^ | ^15^ | ^SCZ^ | ^NA^ | ^NA^ | ^100,00^ | ^100,00^ | ^OPA^ | ^none^ | ^PSI^ | ^yoga^ | ^PANSS^ | ^4,0^ | ^6,67^ |
| ^Pandina 2010^ | ^160^ | ^55^ | ^SCZ^ | ^39,00^ | ^NA^ | ^67,00^ | ^ns^ | ^none^ | ^placebo^ | ^AP^ | ^paliperidone^ | ^PANSS^ | ^13,0^ | ^48,93^ |
| ^Pandina 2010^ | ^165^ | ^55^ | ^SCZ^ | ^39,00^ | ^NA^ | ^67,00^ | ^ns^ | ^none^ | ^placebo^ | ^AP^ | ^paliperidone^ | ^PANSS^ | ^13,0^ | ^48,93^ |
| ^Pandina 2010^ | ^163^ | ^55^ | ^SCZ^ | ^39,00^ | ^NA^ | ^67,00^ | ^ns^ | ^none^ | ^placebo^ | ^AP^ | ^paliperidone^ | ^PANSS^ | ^13,0^ | ^48,93^ |
| ^Poyurovsky 2003^ | ^11^ | ^13^ | ^SCZ^ | ^45,50^ | ^42,50^ | ^69,00^ | ^73,00^ | ^CA^ | ^placebo^ | ^OPA^ | ^other antidepressant^ | ^SANS^ | ^4,0^ | ^20,00^ |
| ^Rabany 2014^ | ^20^ | ^10^ | ^SCZ^ | ^35,90^ | ^33,10^ | ^80,00^ | ^65,00^ | ^AA^ | ^placebo^ | ^BS^ | ^TMS deep^ | ^PANSS^ | ^8,0^ | ^0,00^ |
| ^Rao 2021^ | ^45^ | ^44^ | ^SCZ^ | ^33,65^ | ^34,79^ | ^73,00^ | ^64,00^ | ^mixed^ | ^WL^ | ^PSI^ | ^yoga^ | ^SANS^ | ^12,0^ | ^23,60^ |
| ^Rapado-Castro 2015^ | ^62^ | ^59^ | ^SCZ^ | ^36,20^ | ^36,50^ | ^69,00^ | ^71,00^ | ^AA^ | ^placebo^ | ^OPA^ | ^N-acetyl cysteine^ | ^PANSS^ | ^24,0^ | ^0,00^ |
| ^Rapaport 2005^ | ^17^ | ^18^ | ^SCZ^ | ^47,30^ | ^44,10^ | ^76,00^ | ^89,00^ | ^AA^ | ^placebo^ | ^OPA^ | ^other immunomodulator^ | ^SANS^ | ^8,0^ | ^7,89^ |
| ^Rezaei 2013^ | ^20^ | ^20^ | ^SCZ^ | ^33,00^ | ^33,50^ | ^55,00^ | ^60,00^ | ^SA^ | ^placebo^ | ^OPA^ | ^memantine^ | ^PANSS^ | ^8,0^ | ^5,00^ |
| ^Rezaei 2017^ | ^42^ | ^42^ | ^SCZ^ | ^36,19^ | ^37,40^ | ^77,00^ | ^77,00^ | ^SA^ | ^placebo^ | ^OPA^ | ^other pharma^ | ^PANSS^ | ^8,0^ | ^4,54^ |
| ^Reznik 2000^ | ^14^ | ^16^ | ^SCZ^ | ^35,50^ | ^38,80^ | ^75,00^ | ^71,00^ | ^mixed^ | ^none^ | ^OPA^ | ^fluvoxamine^ | ^PANSS^ | ^8,0^ | ^0,00^ |
| ^Ritsner 2006^ | ^27,5^ | ^28^ | ^SCZ^ | ^NA^ | ^NA^ | ^ns^ | ^ns^ | ^mixed^ | ^placebo^ | ^OPA^ | ^dehydroepiandrosterone^ | ^PANSS^ | ^12,0^ | ^0,00^ |
| ^Ritsner 2010^ | ^14^ | ^4^ | ^SCZ^ | ^34,60^ | ^38,30^ | ^73,00^ | ^64,00^ | ^AA^ | ^placebo^ | ^OPA^ | ^other hormone^ | ^PANSS^ | ^8,0^ | ^0,00^ |
| ^Ritsner 2010^ | ^6^ | ^4^ | ^SCZ^ | ^34,60^ | ^34,30^ | ^73,00^ | ^83,00^ | ^AA^ | ^placebo^ | ^OPA^ | ^other hormone^ | ^PANSS^ | ^8,0^ | ^0,00^ |
| ^Ritsner 2010^ | ^13^ | ^4^ | ^SCZ^ | ^34,60^ | ^35,50^ | ^73,00^ | ^77,00^ | ^AA^ | ^placebo^ | ^OPA^ | ^dehydroepiandrosterone^ | ^PANSS^ | ^8,0^ | ^0,00^ |
| ^Rus-Calafell 2013^ | ^13^ | ^18^ | ^SCZ^ | ^42,39^ | ^37,54^ | ^83,00^ | ^77,00^ | ^mixed^ | ^none^ | ^PSI^ | ^group therapy social skills^ | ^PANSS^ | ^26,0^ | ^0,00^ |
| ^Sachs 2012^ | ^20^ | ^18^ | ^SCZ^ | ^31,72^ | ^27,20^ | ^0,00^ | ^1,00^ | ^AA^ | ^none^ | ^PSI^ | ^emotional affect recognition^ | ^PANSS^ | ^6,0^ | ^5,26^ |
| ^Salokongas 1996^ | ^45^ | ^45^ | ^SCZ^ | ^NA^ | ^NA^ | ^ns^ | ^ns^ | ^AA^ | ^placebo^ | ^OPA^ | ^citalopram^ | ^PANSS^ | ^12,0^ | ^13,00^ |
| ^Samadi 2017^ | ^18^ | ^20^ | ^SCZ^ | ^40,35^ | ^36,61^ | ^5,00^ | ^11,00^ | ^SA^ | ^placebo^ | ^OPA^ | ^ondansetron^ | ^PANSS^ | ^12,0^ | ^13,63^ |
| ^Shafti 2016a^ | ^25^ | ^25^ | ^SCZ^ | ^39,36^ | ^42,18^ | ^100,00^ | ^100,00^ | ^SA^ | ^placebo^ | ^OPA^ | ^armodafinil modafinil^ | ^SANS^ | ^8,0^ | ^0,00^ |
| ^Sheitman 2004^ | ^11^ | ^11^ | ^SCZ^ | ^42,00^ | ^41,50^ | ^91,00^ | ^91,00^ | ^AA^ | ^placebo^ | ^OPA^ | ^other hormone^ | ^PANSS^ | ^4,0^ | ^0,00^ |
| ^Shen 2014^ | ^77^ | ^26^ | ^SCZ^ | ^39,60^ | ^40,10^ | ^73,00^ | ^65,00^ | ^mixed^ | ^placebo^ | ^AP^ | ^olanzapine^ | ^PANSS^ | ^6,0^ | ^62,62^ |
| ^Shen 2014^ | ^82^ | ^26^ | ^SCZ^ | ^39,60^ | ^39,30^ | ^73,00^ | ^75,00^ | ^mixed^ | ^placebo^ | ^AP^ | ^other antipsychotic^ | ^PANSS^ | ^6,0^ | ^62,62^ |
| ^Shen 2014^ | ^77^ | ^26^ | ^SCZ^ | ^39,60^ | ^41,80^ | ^73,00^ | ^76,00^ | ^mixed^ | ^placebo^ | ^AP^ | ^other antipsychotic^ | ^PANSS^ | ^6,0^ | ^62,62^ |
| ^Shibre 2010^ | ^46^ | ^45^ | ^SCZ^ | ^34,50^ | ^36,10^ | ^100,00^ | ^100,00^ | ^ns^ | ^placebo^ | ^OPA^ | ^other pharma^ | ^PANSS^ | ^26,1^ | ^13,12^ |
| ^Shiloh 1997^ | ^16^ | ^12^ | ^SCZ^ | ^37,10^ | ^40,30^ | ^67,00^ | ^69,00^ | ^SA^ | ^placebo^ | ^AP^ | ^other antipsychotic^ | ^SANS^ | ^10,0^ | ^0,00^ |
| ^Silver 1992^ | ^15^ | ^15^ | ^SCZ^ | ^42,00^ | ^41,00^ | ^60,00^ | ^67,00^ | ^mixed^ | ^placebo^ | ^OPA^ | ^fluvoxamine^ | ^SANS^ | ^5,0^ | ^0,00^ |
| ^Small 1997^ | ^94^ | ^48^ | ^SCZ^ | ^36,00^ | ^38,00^ | ^67,00^ | ^78,00^ | ^mixed^ | ^placebo^ | ^AP^ | ^quetiapine^ | ^PANSS^ | ^6,0^ | ^55,33^ |
| ^Small 1997^ | ^96^ | ^48^ | ^SCZ^ | ^36,00^ | ^37,00^ | ^67,00^ | ^69,00^ | ^mixed^ | ^placebo^ | ^AP^ | ^quetiapine^ | ^PANSS^ | ^6,0^ | ^55,33^ |
| ^Smith 2016^ | ^45^ | ^46^ | ^SCZ^ | ^43,60^ | ^46,60^ | ^85,00^ | ^83,00^ | ^mixed^ | ^placebo^ | ^OPA^ | ^other pharma^ | ^PANSS^ | ^8,0^ | ^25,31^ |
| ^Spina 1994^ | ^17^ | ^17^ | ^SCZ^ | ^45,40^ | ^45,40^ | ^65,00^ | ^71,00^ | ^mixed^ | ^placebo^ | ^OPA^ | ^fluoxetine^ | ^SANS^ | ^12,0^ | ^11,74^ |
| ^Strous 2007^ | ^20^ | ^20^ | ^SCZ_spectrum_unspecified^ | ^32,20^ | ^35,70^ | ^70,00^ | ^65,00^ | ^SA^ | ^placebo^ | ^OPA^ | ^dehydroepiandrosterone^ | ^PANSS^ | ^12,0^ | ^22,50^ |
| ^Takahashi 2013^ | ^160^ | ^164^ | ^SCZ^ | ^44,00^ | ^46,00^ | ^51,00^ | ^64,00^ | ^ns^ | ^placebo^ | ^AP^ | ^paliperidone^ | ^PANSS NAA/NDE^ | ^13,0^ | ^53,25^ |
| ^Tiihonen 2005^ | ^26^ | ^26^ | ^SCZ^ | ^45,50^ | ^42,00^ | ^ns^ | ^ns^ | ^AA^ | ^placebo^ | ^OPA^ | ^other anticonvulsant^ | ^PANSS^ | ^12,0^ | ^30,77^ |
| ^Tollefson 1997^ | ^65^ | ^17^ | ^SCZ^ | ^36,00^ | ^NA^ | ^88,00^ | ^ns^ | ^mixed^ | ^placebo^ | ^AP^ | ^olanzapine^ | ^SANS^ | ^6,0^ | ^50,51^ |
| ^Tollefson 1997^ | ^64^ | ^17^ | ^SCZ^ | ^36,00^ | ^NA^ | ^88,00^ | ^ns^ | ^mixed^ | ^placebo^ | ^AP^ | ^olanzapine^ | ^SANS^ | ^6,0^ | ^50,51^ |
| ^Tollefson 1997^ | ^69^ | ^17^ | ^SCZ^ | ^36,00^ | ^NA^ | ^88,00^ | ^ns^ | ^mixed^ | ^placebo^ | ^AP^ | ^olanzapine^ | ^SANS^ | ^6,0^ | ^50,51^ |
| ^Tollefson 1997^ | ^69^ | ^17^ | ^SCZ^ | ^36,00^ | ^NA^ | ^88,00^ | ^ns^ | ^mixed^ | ^placebo^ | ^AP^ | ^haloperidol^ | ^SANS^ | ^6,0^ | ^50,51^ |
| ^Tollefson 1999^ | ^53^ | ^53^ | ^SCZ^ | ^38,00^ | ^38,60^ | ^66,00^ | ^76,00^ | ^SA^ | ^placebo^ | ^AP^ | ^olanzapine^ | ^PANSS^ | ^0,5^ | ^10,38^ |
| ^Truffinet 1999^ | ^63^ | ^34^ | ^SCZ^ | ^38,90^ | ^37,10^ | ^88,00^ | ^89,00^ | ^mixed^ | ^placebo^ | ^OPA^ | ^other pharma^ | ^PANSS^ | ^4,0^ | ^28,87^ |
| ^Tsai 1998^ | ^14^ | ^15^ | ^SCZ^ | ^31,70^ | ^33,90^ | ^67,00^ | ^43,00^ | ^AA^ | ^placebo^ | ^OPA^ | ^D-serine^ | ^SANS^ | ^6,0^ | ^6,90^ |
| ^Tsai 2004^ | ^17^ | ^21^ | ^SCZ^ | ^33,40^ | ^29,80^ | ^62,00^ | ^59,00^ | ^AA^ | ^placebo^ | ^OPA^ | ^sarcosine^ | ^SANS^ | ^6,0^ | ^5,26^ |
| ^Twamley 2012^ | ^38^ | ^31^ | ^SCZ^ | ^48,80^ | ^43,10^ | ^68,00^ | ^63,00^ | ^AA^ | ^none^ | ^PSI^ | ^cognitive remediation^ | ^PANSS^ | ^12,0^ | ^30,43^ |
| ^Umbricht 2014^ | ^82^ | ^27^ | ^SCZ^ | ^39,00^ | ^41,10^ | ^56,00^ | ^70,00^ | ^AA^ | ^placebo^ | ^OPA^ | ^bitopertin^ | ^PANSS^ | ^8,0^ | ^28,48^ |
| ^Umbricht 2014^ | ^81^ | ^27^ | ^SCZ^ | ^39,00^ | ^40,70^ | ^56,00^ | ^77,00^ | ^AA^ | ^placebo^ | ^OPA^ | ^bitopertin^ | ^PANSS^ | ^8,0^ | ^28,48^ |
| ^Umbricht 2014^ | ^79^ | ^27^ | ^SCZ^ | ^39,00^ | ^38,90^ | ^56,00^ | ^68,00^ | ^AA^ | ^placebo^ | ^OPA^ | ^bitopertin^ | ^PANSS^ | ^8,0^ | ^28,48^ |
| ^Vahia 2013^ | ^98^ | ^89^ | ^SCZ^ | ^51,60^ | ^53,40^ | ^79,00^ | ^77,00^ | ^AA^ | ^placebo^ | ^OPA^ | ^citalopram^ | ^PANSS^ | ^12,0^ | ^ns^ |
| ^Vincenzi 2014^ | ^30^ | ^30^ | ^SCZ^ | ^44,53^ | ^42,57^ | ^53,00^ | ^73,00^ | ^AA^ | ^placebo^ | ^OPA^ | ^other statin^ | ^PANSS^ | ^12,0^ | ^18,33^ |
| ^Wang 2022^ | ^33^ | ^26^ | ^SCZ^ | ^24,15^ | ^23,79^ | ^42,00^ | ^45,00^ | ^AA^ | ^placebo^ | ^BS^ | ^TMS theta burst^ | ^PANSS^ | ^2,0^ | ^0,00^ |
| ^Wang L 2020^ | ^25^ | ^25^ | ^SCZ^ | ^NA^ | ^NA^ | ^ns^ | ^ns^ | ^AA^ | ^placebo^ | ^BS^ | ^TMS theta burst^ | ^PANSS^ | ^8,0^ | ^0,00^ |
| ^Wang N 2020^ | ^42^ | ^42^ | ^SCZ^ | ^71,60^ | ^71,30^ | ^50,00^ | ^50,00^ | ^SA^ | ^none^ | ^OPA^ | ^other antidepressant^ | ^PANSS^ | ^8,0^ | ^0,00^ |
| ^Wang X 2018^ | ^21^ | ^21^ | ^SCZ^ | ^29,40^ | ^30,50^ | ^57,00^ | ^52,00^ | ^AA^ | ^placebo^ | ^OPA^ | ^intravenous sodium nitroprusside^ | ^PANSS^ | ^4,0^ | ^0,00^ |
| ^Wass 2011^ | ^10^ | ^10^ | ^SCZ^ | ^43,20^ | ^43,20^ | ^80,00^ | ^80,00^ | ^AA^ | ^placebo^ | ^OPA^ | ^other nutraceutical^ | ^PANSS^ | ^8,0^ | ^0,00^ |
| ^Weiser 2012^ | ^97^ | ^98^ | ^SCZ^ | ^39,75^ | ^39,39^ | ^71,00^ | ^76,00^ | ^AA^ | ^placebo^ | ^OPA^ | ^D-serine^ | ^PANSS^ | ^16,0^ | ^23,60^ |
| ^Weiser 2017^ | ^100^ | ^100^ | ^SCZ^ | ^55,80^ | ^56,60^ | ^0,00^ | ^0,00^ | ^AA^ | ^placebo^ | ^OPA^ | ^raloxifene^ | ^PANSS^ | ^16,0^ | ^13,00^ |
| ^WÃ¶lwer 2005^ | ^24^ | ^13^ | ^SCZ^ | ^35,20^ | ^36,70^ | ^84,00^ | ^58,00^ | ^CA^ | ^none^ | ^PSI^ | ^cognitive remediation^ | ^PANSS^ | ^6,0^ | ^23,76^ |
| ^WÃ¶lwer 2005^ | ^28^ | ^13^ | ^SCZ^ | ^35,20^ | ^31,50^ | ^84,00^ | ^89,00^ | ^CA^ | ^none^ | ^PSI^ | ^emotional affect recognition^ | ^PANSS^ | ^6,0^ | ^23,76^ |
| ^Xiao 2011^ | ^46^ | ^44^ | ^SCZ^ | ^55,21^ | ^46,02^ | ^49,00^ | ^49,00^ | ^SA^ | ^placebo^ | ^OPA^ | ^other nutraceutical^ | ^PANSS^ | ^8,0^ | ^11,11^ |
| ^Xiao 2012^ | ^55^ | ^54^ | ^SCZ^ | ^48,90^ | ^47,19^ | ^69,00^ | ^79,00^ | ^SA^ | ^placebo^ | ^OPA^ | ^pharma antiparkinson^ | ^PANSS^ | ^8,0^ | ^7,55^ |
| ^Zeinoddini 2014^ | ^40^ | ^40^ | ^SCZ^ | ^34,05^ | ^32,33^ | ^52,00^ | ^55,00^ | ^SA^ | ^placebo^ | ^OPA^ | ^other nutraceutical^ | ^PANSS^ | ^8,0^ | ^10,00^ |
| ^Zhang 2018^ | ^50^ | ^25^ | ^SCZ^ | ^33,68^ | ^33,14^ | ^48,00^ | ^50,00^ | ^none^ | ^placebo^ | ^OPA^ | ^minocycline^ | ^PANSS^ | ^12,0^ | ^24,00^ |
| ^Zhou 1999^ | ^27^ | ^27^ | ^SCZ^ | ^44,50^ | ^42,20^ | ^52,00^ | ^37,00^ | ^SA^ | ^placebo^ | ^OPA^ | ^ginkgo biloba^ | ^SANS^ | ^12,0^ | ^ns^ |
| ^Zhuo 2019^ | ^35^ | ^35^ | ^SCZ^ | ^30,63^ | ^28,97^ | ^54,00^ | ^63,00^ | ^AA^ | ^placebo^ | ^BS^ | ^TMS repetitive^ | ^PANSS^ | ^4,0^ | ^14,29^ |
| ^Zimbroff 1997^ | ^71^ | ^12^ | ^SCZ^ | ^38,70^ | ^38,10^ | ^78,00^ | ^83,00^ | ^none^ | ^placebo^ | ^AP^ | ^haloperidol^ | ^SANS^ | ^8,0^ | ^51,00^ |
| ^Zimbroff 1997^ | ^67^ | ^12^ | ^SCZ^ | ^38,70^ | ^39,90^ | ^78,00^ | ^81,00^ | ^none^ | ^placebo^ | ^AP^ | ^haloperidol^ | ^SANS^ | ^8,0^ | ^51,00^ |
| ^Zimbroff 1997^ | ^70^ | ^12^ | ^SCZ^ | ^38,70^ | ^39,00^ | ^78,00^ | ^76,00^ | ^none^ | ^placebo^ | ^AP^ | ^haloperidol^ | ^SANS^ | ^8,0^ | ^51,00^ |
| ^Zimbroff 1997^ | ^76^ | ^12^ | ^SCZ^ | ^38,70^ | ^37,80^ | ^78,00^ | ^80,00^ | ^none^ | ^placebo^ | ^AP^ | ^other antipsychotic^ | ^SANS^ | ^8,0^ | ^51,00^ |
| ^Zimbroff 1997^ | ^68^ | ^12^ | ^SCZ^ | ^38,70^ | ^40,40^ | ^78,00^ | ^76,00^ | ^none^ | ^placebo^ | ^AP^ | ^other antipsychotic^ | ^SANS^ | ^8,0^ | ^51,00^ |
| ^Zimbroff 1997^ | ^72^ | ^12^ | ^SCZ^ | ^38,70^ | ^39,50^ | ^78,00^ | ^69,00^ | ^none^ | ^placebo^ | ^AP^ | ^other antipsychotic^ | ^SANS^ | ^8,0^ | ^51,00^ |
| ^Zisook 2009^ | ^109^ | ^103^ | ^SCZ^ | ^51,70^ | ^53,14^ | ^79,00^ | ^78,00^ | ^AA^ | ^placebo^ | ^OPA^ | ^citalopram^ | ^PANSS^ | ^12,0^ | ^31,60^ |
| ^Zoccali 2007^ | ^30^ | ^30^ | ^SCZ^ | ^30,20^ | ^32,50^ | ^43,00^ | ^50,00^ | ^SA^ | ^placebo^ | ^OPA^ | ^lamotrigine^ | ^SANS^ | ^24,0^ | ^85,00^ |
| ^Albus 1986^ | ^10^ | ^10^ | ^SCZ^ | ^44,20^ | ^44,20^ | ^100,00^ | ^100,00^ | ^AA^ | ^placebo^ | ^OPA^ | ^other pharma^ | ^BPRS neg^ | ^4,0^ | ^0,00^ |
| ^Behrouzian 2022^ | ^44^ | ^44^ | ^SCZ^ | ^NA^ | ^NA^ | ^77,00^ | ^82,00^ | ^CA^ | ^placebo^ | ^OPA^ | ^other nutraceutical^ | ^PANSS^ | ^6,0^ | ^0,00^ |
| ^Bergson 2024^ | ^11^ | ^6^ | ^SCZ^ | ^34,00^ | ^40,50^ | ^70,00^ | ^82,00^ | ^PSI^ | ^none^ | ^PSI^ | ^other PSI^ | ^PANSS^ | ^16,0^ | ^8,51^ |
| ^Bergson 2024^ | ^12^ | ^6^ | ^SCZ^ | ^34,00^ | ^31,90^ | ^70,00^ | ^83,00^ | ^PSI^ | ^none^ | ^PSI^ | ^other PSI^ | ^PANSS^ | ^16,0^ | ^8,51^ |
| ^Böge 2024^ | ^25^ | ^23^ | ^SCZ^ | ^42,66^ | ^45,16^ | ^61,00^ | ^52,00^ | ^mixed^ | ^WL^ | ^PSI^ | ^mindfulness^ | ^PANSS^ | ^4,0^ | ^6,25^ |
| ^Calafell 2013^ | ^18^ | ^18^ | ^SCZ^ | ^42,39^ | ^37,54^ | ^83,00^ | ^77,00^ | ^PSI^ | ^none^ | ^PSI^ | ^group therapy social skills^ | ^PANSS^ | ^24,0^ | ^13,89^ |
| ^Chen 2023^ | ^60^ | ^60^ | ^SCZ^ | ^37,95^ | ^38,47^ | ^62,00^ | ^47,00^ | ^mixed^ | ^none^ | ^PSI^ | ^CBT cognitive behavioral therapy^ | ^PANSS^ | ^12,0^ | ^0,00^ |
| ^Chouinard 1993^ | ^24^ | ^4^ | ^SCZ^ | ^NA^ | ^NA^ | ^ns^ | ^ns^ | ^none^ | ^placebo^ | ^AP^ | ^risperidone^ | ^PANSS^ | ^8,0^ | ^48,10^ |
| ^Chouinard 1993^ | ^22^ | ^4^ | ^SCZ^ | ^NA^ | ^NA^ | ^ns^ | ^ns^ | ^none^ | ^placebo^ | ^AP^ | ^risperidone^ | ^PANSS^ | ^8,0^ | ^48,10^ |
| ^Chouinard 1993^ | ^22^ | ^4^ | ^SCZ^ | ^NA^ | ^NA^ | ^ns^ | ^ns^ | ^none^ | ^placebo^ | ^AP^ | ^risperidone^ | ^PANSS^ | ^8,0^ | ^48,10^ |
| ^Chouinard 1993^ | ^24^ | ^4^ | ^SCZ^ | ^NA^ | ^NA^ | ^ns^ | ^ns^ | ^none^ | ^placebo^ | ^AP^ | ^risperidone^ | ^PANSS^ | ^8,0^ | ^48,10^ |
| ^Chouinard 1993^ | ^21^ | ^4^ | ^SCZ^ | ^NA^ | ^NA^ | ^ns^ | ^ns^ | ^none^ | ^placebo^ | ^AP^ | ^haloperidol^ | ^PANSS^ | ^8,0^ | ^48,10^ |
| ^Davidson 2022^ | ^170^ | ^86^ | ^SCZ^ | ^41,00^ | ^41,00^ | ^62,00^ | ^62,00^ | ^none^ | ^placebo^ | ^OPA^ | ^roluperidone^ | ^PANSS^ | ^12,0^ | ^35,86^ |
| ^Davidson 2022^ | ^171^ | ^86^ | ^SCZ^ | ^41,00^ | ^41,00^ | ^62,00^ | ^60,00^ | ^none^ | ^placebo^ | ^OPA^ | ^roluperidone^ | ^PANSS^ | ^12,0^ | ^35,86^ |
| ^Emami 2023^ | ^32^ | ^32^ | ^SCZ^ | ^40,86^ | ^40,17^ | ^34,00^ | ^31,00^ | ^mixed^ | ^none^ | ^PSI^ | ^other PSI^ | ^SANS^ | ^12,0^ | ^9,37^ |
| ^Fitzgerald 2008^ | ^10^ | ^10^ | ^SCZ^ | ^33,20^ | ^37,20^ | ^75,00^ | ^83,00^ | ^AA^ | ^placebo^ | ^BS^ | ^TMS repetitive^ | ^PANSS^ | ^3,0^ | ^25,00^ |
| ^Hasson-Ohayon 2024^ | ^31^ | ^23^ | ^SCZ^ | ^NA^ | ^NA^ | ^ns^ | ^ns^ | ^mixed^ | ^WL^ | ^PSI^ | ^metacognitive training^ | ^PANSS^ | ^24,0^ | ^35,18^ |
| ^Herizchi 2024^ | ^30^ | ^30^ | ^SCZ^ | ^37,13^ | ^36,50^ | ^87,00^ | ^87,00^ | ^AA^ | ^placebo^ | ^OPA^ | ^rivastigmine^ | ^PANSS^ | ^8,0^ | ^8,33^ |
| ^Horiguchi 2023^ | ^27^ | ^26^ | ^SCZ^ | ^56,70^ | ^59,60^ | ^77,00^ | ^26,00^ | ^AA^ | ^placebo^ | ^OPA^ | ^other nutraceutical^ | ^PANSS^ | ^12,0^ | ^5,66^ |
| ^Husain 2024^ | ^16^ | ^10^ | ^SCZ^ | ^28,00^ | ^27,00^ | ^53,00^ | ^62,00^ | ^AA^ | ^placebo^ | ^OPA^ | ^sodium benzoate^ | ^PANSS^ | ^12,0^ | ^22,53^ |
| ^Husain 2024^ | ^14^ | ^10^ | ^SCZ^ | ^28,00^ | ^30,00^ | ^53,00^ | ^50,00^ | ^AA^ | ^placebo^ | ^OPA^ | ^N-acetyl cysteine^ | ^PANSS^ | ^12,0^ | ^22,53^ |
| ^Jockers-ScherÃ¼bl 2005^ | ^11^ | ^14^ | ^SCZ^ | ^40,80^ | ^40,00^ | ^57,00^ | ^36,00^ | ^AA^ | ^placebo^ | ^OPA^ | ^paroxetine^ | ^PANSS^ | ^12,0^ | ^8,00^ |
| ^Kong 2024^ | ^60^ | ^60^ | ^SCZ^ | ^44,60^ | ^45,22^ | ^100,00^ | ^100,00^ | ^mixed^ | ^none^ | ^PSI^ | ^dance movement therapy^ | ^PANSS^ | ^12,0^ | ^ns^ |
| ^Kruiper 2023^ | ^16^ | ^16^ | ^SCZ^ | ^37,63^ | ^36,31^ | ^88,00^ | ^88,00^ | ^AA^ | ^placebo^ | ^OPA^ | ^other pharma^ | ^PANSS^ | ^6,0^ | ^6,25^ |
| ^Li 2024^ | ^28^ | ^14^ | ^SCZ^ | ^30,29^ | ^34,68^ | ^57,00^ | ^43,00^ | ^AA^ | ^placebo^ | ^BS^ | ^TMS theta burst^ | ^PANSS^ | ^2,9^ | ^31,82^ |
| ^Li 2024^ | ^33^ | ^14^ | ^SCZ^ | ^30,29^ | ^32,06^ | ^57,00^ | ^30,00^ | ^AA^ | ^placebo^ | ^BS^ | ^TMS theta burst^ | ^PANSS^ | ^2,9^ | ^31,82^ |
| ^Markiewicz 2024^ | ^18^ | ^19^ | ^SCZ^ | ^37,84^ | ^36,61^ | ^100,00^ | ^100,00^ | ^AA^ | ^none^ | ^PSI^ | ^other PSI^ | ^PANSS^ | ^12,0^ | ^0,00^ |
| ^Mukai 2024^ | ^45^ | ^45^ | ^SCZ^ | ^35,60^ | ^37,30^ | ^81,00^ | ^82,00^ | ^none^ | ^placebo^ | ^AP^ | ^risperidone^ | ^PANSS^ | ^4,0^ | ^29,00^ |
| ^Mukai 2024^ | ^90^ | ^45^ | ^SCZ^ | ^35,60^ | ^38,40^ | ^81,00^ | ^74,00^ | ^none^ | ^placebo^ | ^OPA^ | ^other pharma^ | ^PANSS^ | ^4,0^ | ^29,00^ |
| ^Mullapudi 2023^ | ^30^ | ^30^ | ^SCZ^ | ^34,95^ | ^31,33^ | ^65,00^ | ^67,00^ | ^SA^ | ^none^ | ^PSI^ | ^yoga^ | ^SANS^ | ^24,0^ | ^31,67^ |
| ^Pawlak 2023^ | ^30^ | ^30^ | ^SCZ^ | ^40,20^ | ^36,70^ | ^50,00^ | ^71,00^ | ^AA^ | ^placebo^ | ^OPA^ | ^sarcosine^ | ^PANSS^ | ^24,0^ | ^3,33^ |
| ^Petersen 2005^ | ^275^ | ^272^ | ^SCZ^ | ^26,60^ | ^26,60^ | ^60,00^ | ^58,00^ | ^mixed^ | ^none^ | ^PSI^ | ^other PSI^ | ^SANS^ | ^104,0^ | ^32,54^ |
| ^Pourghasem 2022^ | ^15^ | ^8^ | ^SCZ^ | ^NA^ | ^NA^ | ^80,00^ | ^73,00^ | ^SA^ | ^placebo^ | ^OPA^ | ^aspirin^ | ^PANSS^ | ^8,0^ | ^0,00^ |
| ^Pourghasem 2022^ | ^15^ | ^8^ | ^SCZ^ | ^NA^ | ^NA^ | ^80,00^ | ^80,00^ | ^SA^ | ^placebo^ | ^OPA^ | ^other statin^ | ^PANSS^ | ^8,0^ | ^0,00^ |
| ^Pu 2023^ | ^67^ | ^67^ | ^SCZ^ | ^44,82^ | ^42,71^ | ^42,00^ | ^41,00^ | ^AA^ | ^placebo^ | ^OPA^ | ^anti inflammatory any^ | ^SANS^ | ^12,0^ | ^20,90^ |
| ^Rao 2024^ | ^50^ | ^50^ | ^SCZ^ | ^36,51^ | ^37,43^ | ^66,00^ | ^62,00^ | ^CA^ | ^none^ | ^PSI^ | ^psychoeducational medication management training^ | ^PANSS^ | ^6,0^ | ^0,00^ |
| ^Richardson 2007^ | ^43^ | ^47^ | ^SCZ^ | ^42,60^ | ^39,60^ | ^66,00^ | ^65,00^ | ^mixed^ | ^none^ | ^PSI^ | ^other PSI^ | ^SANS^ | ^26,0^ | ^55,00^ |
| ^Sayed 2024^ | ^60^ | ^60^ | ^SCZ^ | ^37,62^ | ^36,37^ | ^45,00^ | ^62,00^ | ^AA^ | ^none^ | ^PSI^ | ^group therapy integrated psychological^ | ^PANSS^ | ^8,0^ | ^0,00^ |
| ^ÅženormancÄ± 2020^ | ^20^ | ^19^ | ^SCZ^ | ^43,00^ | ^40,50^ | ^63,00^ | ^60,00^ | ^mixed^ | ^none^ | ^LS^ | ^physical exercise^ | ^SANS^ | ^12,0^ | ^0,00^ |
| ^Steuwe 2024^ | ^51^ | ^66^ | ^SCZ^ | ^41,08^ | ^39,46^ | ^65,00^ | ^65,00^ | ^mixed^ | ^none^ | ^PSI^ | ^CBT cognitive behavioral therapy^ | ^PANSS^ | ^52,0^ | ^28,20^ |
| ^Strzelecki 2018^ | ^29^ | ^30^ | ^SCZ^ | ^40,20^ | ^37,30^ | ^50,00^ | ^66,00^ | ^AA^ | ^placebo^ | ^OPA^ | ^sarcosine^ | ^PANSS^ | ^24,0^ | ^6,66^ |
| ^Tang 1994^ | ^38^ | ^38^ | ^SCZ^ | ^33,50^ | ^33,50^ | ^80,00^ | ^80,00^ | ^AA^ | ^none^ | ^PSI^ | ^music therapy^ | ^SANS^ | ^4,0^ | ^0,00^ |
| ^Thomas 2018^ | ^24^ | ^22^ | ^SCZ^ | ^35,73^ | ^34,54^ | ^41,00^ | ^54,00^ | ^mixed^ | ^none^ | ^PSI^ | ^computer assisted cognitive rehabilitation^ | ^SANS^ | ^12,0^ | ^21,74^ |
| ^Varambally 2024^ | ^55^ | ^55^ | ^SCZ^ | ^34,90^ | ^31,90^ | ^56,00^ | ^58,00^ | ^AA^ | ^none^ | ^PSI^ | ^yoga^ | ^SANS^ | ^24,0^ | ^36,36^ |
| ^Zhang 2000^ | ^43^ | ^39^ | ^SCZ^ | ^44,20^ | ^44,60^ | ^56,00^ | ^58,00^ | ^SA^ | ^placebo^ | ^OPA^ | ^gingko biloba^ | ^SANS^ | ^12,0^ | ^0,00^ |
| ^Zhu 2023^ | ^40^ | ^40^ | ^SCZ^ | ^48,90^ | ^50,80^ | ^40,00^ | ^45,00^ | ^SA^ | ^none^ | ^OPA^ | ^other nutraceutical^ | ^PANSS^ | ^6,0^ | ^0,00^ |
| ^Aloi 2020^ | ^20^ | ^21^ | ^SCZ^ | ^52,14^ | ^50,05^ | ^81,00^ | ^60,00^ | ^AA^ | ^none^ | ^PSI^ | ^group therapy integrated psychological^ | ^PANSS^ | ^52^ | ^0,00^ |
| ^Choi 2018^ | ^19^ | ^19^ | ^SCZ^ | ^50,89^ | ^49,58^ | ^52,63^ | ^63,16^ | ^AA^ | ^none^ | ^PSI^ | ^other psychosocial^ | ^PANSS^ | ^12^ | ^7,02^ |
| ^Fiszdon 2016^ | ^25^ | ^50^ | ^SCZ^ | ^49,00^ | ^47,22^ | ^64,00^ | ^78,00^ | ^ns^ | ^none^ | ^PSI^ | ^computer assisted cognitive rehabilitation^ | ^PANSS^ | ^16^ | ^26,70^ |
| ^Hegde 2012^ | ^11^ | ^12^ | ^SCZ^ | ^31,00^ | ^27,75^ | ^90,00^ | ^75,00^ | ^AA^ | ^none^ | ^PSI^ | ^cognitive remediation^ | ^PANSS^ | ^24^ | ^49,90^ |
| ^Iwata 2017^ | ^31^ | ^29^ | ^SCZ^ | ^34,50^ | ^34,20^ | ^25,80^ | ^24,14^ | ^AA^ | ^waiting list^ | ^PSI^ | ^cognitive remediation^ | ^PANSS^ | ^12^ | ^6,67^ |
| ^Popova 2014^ | ^29^ | ^24^ | ^SCZ^ | ^35,90^ | ^36,00^ | ^78,94^ | ^63,16^ | ^AA^ | ^none^ | ^PSI^ | ^computer assisted cognitive rehabilitation^ | ^PANSS^ | ^4^ | ^28,75^ |
| ^Popova 2014^ | ^29^ | ^27^ | ^SCZ^ | ^35,90^ | ^39,60^ | ^78,94^ | ^57,89^ | ^AA^ | ^none^ | ^PSI^ | ^emotional affect recognition^ | ^PANSS^ | ^4^ | ^28,75^ |
| ^Roncone 2004^ | ^10^ | ^10^ | ^SCZ^ | ^33,50^ | ^33,90^ | ^70,00^ | ^60,00^ | ^AA^ | ^none^ | ^PSI^ | ^social skills^ | ^BPRS^ | ^24^ | ^0,00^ |

^a^ Where rows are identical = different dosages within the same study.

Abbreviations: Diagnosis: SCZ = schizophrenia; SCA = schizoaffective; SSD = schizophrenia spectum, unspecified. TAU (= treatment as usual): SA = all participants use the same antipsychotic; AA = any antipsychotic, in monotherapy; CA = some/all participants use combinations of two or more antipsychotics; ns = not specified. Treatment categories: AP = antipsychotics; BS = brain stimulation; PSI = pscyhological interventions; LS = lifestyle intervention; OPA = other pharmacological interventions; WL: waiting list. Specific treatments: CCRT = computerized cognitive remediation therapy; TMS = transcranial magnetic stimulation; CBT = cognitive behavioral therapy; CACR = computer assisted cognitive rehabilitation; iv = intravenous; TCS = transcranial current stimulation; GTIP = group therapy integrated psychological; GTSS = group therapy social skills; PSI-E MMT = psychoeducational medication management training; PSST = psychosocial skill training. PANSS = Positive and Negative Syndrome Scale; SANS = Scale for the Assessment of Negative Symptoms; BPRS = Brief Psychiatric Rating Scale; BNSS = Brief Negative Symptom Scale.

**Example of an excluded study**

An example of a study that might have appeared to meet the inclusion criteria but that was the excluded was Bennett et al. (2023)^13^. In this study, the authors aimed to compare the efficacy of an intervention called “Engaging in Community Roles and Experiences (EnCoRE)” - a 12-week program of individual and group meetings that support learning and implementing skills to help individuals with schizophrenia increase engagement in personally-relevant social and community activities – to an active wellness education control condition.

The challenging part here was to determine whether the control condition was an actual control or not. Called “Health and Wellness”, it was a manualized curriculum with sessions on physical activity, nutrition, managing medication side effects, coping with stress, and tobacco use. As such, it was considered an active condition and the study was therefore excluded from the meta-analysis (as control conditions we considered nothing, placebo/sham or waiting list)

# **Supplementary Materials - References**

1. Kay SR, Fiszbein A, Opler LA. The positive and negative syndrome scale (PANSS) for schizophrenia. *Schizophr Bull*. 1987;13(2):261-76. doi:10.1093/schbul/13.2.261

2. Hopkins SC, Ogirala A, Loebel A, Koblan KS. Transformed PANSS Factors Intended to Reduce Pseudospecificity Among Symptom Domains and Enhance Understanding of Symptom Change in Antipsychotic-Treated Patients With Schizophrenia. *Schizophr Bull*. 2018;44(3):593-602. doi:10.1093/schbul/sbx101

3. Andreasen NC. The Scale for the Assessment of Negative Symptoms (SANS): conceptual and theoretical foundations. *Br J Psychiatry Suppl*. 1989;(7):49-58.

4. Overall JE, Gorham DR. The brief psychiatric rating scale. *Psychological reports*. 1962;10(3):799-812. doi:10.2466/pr0.1962.10.3

5. Kirkpatrick B, Strauss GP, Nguyen L, et al. The brief negative symptom scale: psychometric properties. *Schizophr Bull*. 2011;37(2):300-5. doi:10.1093/schbul/sbq059

6. Kring AM, Gur RE, Blanchard JJ, Horan WP, Reise SP. The Clinical Assessment Interview for Negative Symptoms (CAINS): final development and validation. *Am J Psychiatry*. 2013;170(2):165-72. doi:10.1176/appi.ajp.2012.12010109

7. Team TGD. GIMP [Internet]. Available from: <https://www.gimp.org>

8. SC N. Modern Medical Statistics. A Practical Guide. *Biometrics*. 2004;60(1):291. doi:10.1111/j.0006-341X.2004.172_6.x

9. VA HJTJCJCMLTPMW. *Cochrane Handbook for Systematic Reviews of Interventions*. Version 6.5 (updated August 2024) ed. Cochrane; 2024. <https://www.cochrane.org/handbook>

10. Guyatt GH, Oxman AD, Kunz R, et al. GRADE guidelines: 7. Rating the quality of evidence--inconsistency. *J Clin Epidemiol*. 2011;64(12):1294-302. doi:10.1016/j.jclinepi.2011.03.017

11. Higgins JP, Thompson SG, Deeks JJ, Altman DG. Measuring inconsistency in meta-analyses. *Bmj*. 2003;327(7414):557-60. doi:10.1136/bmj.327.7414.557

12. Guyatt G, Zhao Y, Mayer M, et al. GRADE guidance 36: updates to GRADE's approach to addressing inconsistency. *J Clin Epidemiol*. 2023;158:70-83. doi:10.1016/j.jclinepi.2023.03.003

13. Bennett ME, Brown CH, Fang LJ, Blanchard JJ. Increasing social and community participation in veterans living with schizophrenia: A treatment outcome study. Schizophr Res. 2023 Feb;252:262-270. doi: 10.1016/j.schres.2023.01.005.

**References of the included studies**

1. Abaoglu H, Mutlu E, Ak S, Aki E, Anil Yagcioglu AE. The Effect of Life Skills Training on Functioning in Schizophrenia: A Randomized Controlled Trial. Turk psikiyatri dergisi = Turkish journal of psychiatry. 2020;31(1):48-56.

2. Acil AA, Dogan S, Dogan O. The effects of physical exercises to mental state and quality of life in patients with schizophrenia. Journal of Psychiatric and Mental Health Nursing. 2008;15(10):808-15.

3. Afshar H, Roohafza H, Mousavi G, Golchin S, Toghianifar N, Sadeghi M, et al. Topiramate add-on treatment in schizophrenia: a randomised, double-blind, placebo-controlled clinical trial. Journal of Psychopharmacology. 2009;23(2):157-62.

4. Ahmed AO, Hunter KM, Goodrum NM, Batten N-J, Birgenheir D, Hardison E, et al. A randomized study of cognitive remediation for forensic and mental health patients with schizophrenia. Journal of Psychiatric Research. 2015;68:8-18.

5. Akhondzadeh S, Gerami M, Noroozian M, Karamghadiri N, Ghoreishi A, Abbasi S-H, et al. A 12-week, double-blind, placebo-controlled trial of donepezil adjunctive treatment to risperidone in chronic and stable schizophrenia. Progress in Neuro-Psychopharmacology & Biological Psychiatry. 2008;32(8):1810-5.

6. Akhondzadeh S, Ghayyoumi R, Rezaei F, Salehi B, Modabbernia A-H, Maroufi A, et al. Sildenafil adjunctive therapy to risperidone in the treatment of the negative symptoms of schizophrenia: a double-blind randomized placebo-controlled trial. Psychopharmacology. 2011;213(4):809-15.

7. Akhondzadeh S, Malek-Hosseini M. Effect of ritanserin on negative symptoms of schizophrenia: a double-blind randomized placebo-controlled trial. British Journal of Clinical Pharmacology. 2009;68(2):304-.

8. Akhondzadeh S, Mohammadi N, Noroozian M, Karamghadiri N, Ghoreishi A, Jamshidi A-H, et al. Added ondansetron for stable schizophrenia: A double blind, placebo controlled trial. Schizophrenia Research. 2009;107(2-3):206-12.

9. Albus M, von Gellhorn K, Münch U, Naber D, Ackenheil M. A double-blind study with ceruletide in chronic schizophrenic patients: biochemical and clinical results. Psychiatry research. 1986;19(1):1-7.

10. Aloi M, de Filippis R, Lavalle FG, Chiappetta E, Viganò C, Segura-Garcia C, et al. Effectiveness of integrated psychological therapy on clinical, neuropsychological, emotional and functional outcome in schizophrenia: a RCT study. Journal of Mental Health. 2020.

11. Amiri A, Noorbala AA, Nejatisafa AA, Ghoreishi A, Derakhshan MK, Khodaie‐Ardakani MR, et al. Efficacy of selegiline add on therapy to risperidone in the treatment of the negative symptoms of schizophrenia: A double‐blind randomized placebo‐controlled study. Human Psychopharmacology: Clinical and Experimental. 2008;23(2):79-86.

12. Arango C, Kirkpatrick B, Buchanan RW. Fluoxetine as an adjunct to conventional antipsychotic treatment of schizophrenia patients with residual symptoms. Journal of Nervous and Mental Disease. 2000;188(1):50-3.

13. Arato M, O'Connor R, Meltzer H, Group ZS. A 1-year, double-blind, placebo-controlled trial of ziprasidone 40, 80 and 160 mg/day in chronic schizophrenia: the Ziprasidone Extended Use in Schizophrenia (ZEUS) study. International Clinical Psychopharmacology. 2002;17(5):207-15.

14. Arbabi M, Bagheri M, Rezaei F, Ahmadi-Abhari S-A, Tabrizi M, Khalighi-Sigaroudi F, et al. A placebo-controlled study of the modafinil added to risperidone in chronic schizophrenia. Psychopharmacology. 2012;220(3):591-8.

15. Arvanitis LA, Miller BG, Borison RL, Pitts WM, Sharif ZA, Hamner MB, et al. Multiple fixed doses of ''Seroquel'' (quetiapine) in patients with acute exacerbation of schizophrenia: A comparison with haloperidol and placebo. Biological Psychiatry. 1997;42(4):233-46.

16. Attari A, Mojdeh A, Soltani FASK, Najarzadegan MR. Aspirin Inclusion in Antipsychotic Treatment on Severity of Symptoms in Schizophrenia: A Randimized Clinical Trial. Iranian Journal of Psychiatry and Behavioral Sciences. 2017;11(1).

17. Bai L, Liang W, Wang Y, Fan N, Zhang Q, Bian Y, et al. Effects of Adjunctive Betahistine Therapy on Lipid Metabolism in Patients with Chronic Schizophrenia: A Randomized Double-Blind Placebo-Controlled Study. Neuropsychiatr Dis Treat. 2023;19:453-60.

18. Bais L, Vercammen A, Stewart R, van Es F, Visser B, Aleman A, et al. Short and Long Term Effects of Left and Bilateral Repetitive Transcranial Magnetic Stimulation in Schizophrenia Patients with Auditory Verbal Hallucinations: A Randomized Controlled Trial. Plos One. 2014;9(10).

19. Banazadeh M, Mehrabani M, Banazadeh N, Dabaghzadeh F, Shahabi F. Evaluating the effect of black myrobalan on cognitive, positive, and negative symptoms in patients with chronic schizophrenia: A randomized, double-blind, placebo-controlled trial. Phytotherapy Research. 2022;36(1):543-50.

20. Bark N, Revheim N, Huq F, Khalderov V, Ganz ZW, Medalia A. The impact of cognitive remediation on psychiatric symptoms of schizophrenia. Schizophrenia Research. 2003;63(3):229-35.

21. Barnes TRE, Leeson VC, Paton C, Marston L, Davies L, Whittaker W, et al. Amisulpride augmentation in clozapine-unresponsive schizophrenia (AMICUS): a double-blind, placebo-controlled, randomised trial of clinical effectiveness and cost-effectiveness. Health Technology Assessment. 2017;21(49):1-+.

22. Barr MS, Farzan F, Tran LC, Fitzgerald PB, Daskalakis ZJ. A randomized controlled trial of sequentially bilateral prefrontal cortex repetitive transcranial magnetic stimulation in the treatment of negative symptoms in schizophrenia. Brain Stimulation. 2012;5(3):337-46.

23. Barrowclough C, Haddock G, Lobban F, Jones S, Siddle R, Roberts C, et al. Group cognitive-behavioural therapy for schizophrenia - Randomised controlled trial. British Journal of Psychiatry. 2006;189:527-32.

24. Barrowclough C, Haddock G, Tarrier N, Lewis SW, Moring J, O’Brien R, et al. Randomized controlled trial of motivational interviewing, cognitive behavior therapy, and family intervention for patients with comorbid schizophrenia and substance use disorders. American Journal of Psychiatry. 2001;158(10):1706-13.

25. Barzegar S, Ahadi M, Barzegar Z, Ghahari S. The Effectiveness of Social Skills Training on Reducing Negative Symptoms of Chronic Schizophrenia. International Journal of Medical Research & Health Sciences. 2016;5(7):323-7.

26. Basavaraju R, Ithal D, Thanki MV, Ramalingaiah AH, Thirthalli J, Reddy RP, et al. Intermittent theta burst stimulation of cerebellar vermis enhances fronto-cerebellar resting state functional connectivity in schizophrenia with predominant negative symptoms: A randomized controlled trial. Schizophrenia Research. 2021;238:108-20.

27. Beasley CM, Tollefson G, Tran P, Satterlee W, Sanger T, Hamilton S, et al. Olanzapine versus placebo and haloperidol - Acute phase results of the North American double-blind olanzapine trial. Neuropsychopharmacology. 1996;14(2):111-23.

28. Behdani F, Hassanzadeh B, Eslamzadeh M, Moradi M, Hebrani P, Dadgarmoghaddam M, et al. Can levetiracetam improve clinical symptoms in schizophrenic patients? A randomized placebo-controlled clinical trial. Int Clin Psychopharmacol. 2022;37(4):159-65.

29. Behere RV, Arasappa R, Jagannathan A, Varambally S, Venkatasubramanian G, Thirthalli J, et al. Effect of yoga therapy on facial emotion recognition deficits, symptoms and functioning in patients with schizophrenia. Acta Psychiatrica Scandinavica. 2011;123(2):147-53.

30. Behrouzian F, Nazarinasab M, Sadegh AM, Abdi L, Sabzevarizadeh M. Effects of zinc sulfate on schizophrenia symptoms in patients undergoing atypical antipsychotic pharmacotherapy. Journal of Family Medicine and Primary Care. 2022;11(12):7795-9.

31. Bellucci DM, Glaberman K, Haslam N. Computer-assisted cognitive rehabilitation reduces negative symptoms in the severely mentally ill. Schizophrenia Research. 2003;59(2-3):225-32.

32. Bergson Z, Ahmed AO, Bell J, Butler PD, Gordon J, Seitz AR, et al. Visual remediation of contrast processing impairments in schizophrenia: A preliminary clinical trial. Schizophrenia Research. 2024;274:396-405.

33. Berk M, Copolov D, Dean O, Lu K, Jeavons S, Schapkaitz I, et al. N-acetyl cysteine as a glutathione precursor for schizophrenia - A double-blind, randomized, placebo-controlled trial. Biological Psychiatry. 2008;64(5):361-8.

34. Berk M, Gama CS, Sundram S, Hustig H, Koopowitz L, D'Souza R, et al. Mirtazapine add-on therapy in the treatment of schizophrenia with atypical antipsychotics: a double-blind, randomised, placebo-controlled clinical trial. Human Psychopharmacology-Clinical and Experimental. 2009;24(3):233-8.

35. Bio DS, Gattaz WF. Vocational rehabilitation improves cognition and negative symptoms in schizophrenia. Schizophrenia Research. 2011;126(1-3):265-9.

36. Boege K, Bergmann N, Zierhut M, Hahne I, Braun A, Kraft J, et al. The relationship between mindfulness and empathy with the oxytocinergic system in persons with schizophrenia spectrum disorders - A proof-of-concept randomized controlled trial (OXYGEN). International Journal of Clinical and Health Psychology. 2024;24(3).

37. Böge K, Hahne I, Bergmann N, Wingenfeld K, Zierhut M, Thomas N, et al. Mindfulness-based group therapy for in-patients with schizophrenia spectrum disorders–Feasibility, acceptability, and preliminary outcomes of a rater-blinded randomized controlled trial. Schizophrenia Research. 2021;228:134-44.

38. Borison RL, Arvanitis LA, Miller BG, Alphs LD, Carman JS, Diamond B, et al. ICI 204,636, an atypical antipsychotic: Efficacy and safety in a multicenter, placebo-controlled trial in patients with schizophrenia. Journal of Clinical Psychopharmacology. 1996;16(2):158-69.

39. Borras L, Boucherie M, Mohr S, Lecomte T, Perroud N, Huguelet P. Increasing self-esteem: Efficacy of a group intervention for individuals with severe mental disorders. European Psychiatry. 2009;24(5):307-16.

40. Boustani H, Pakseresht S, Haghdoust M-R, Malekpoor N. The effect of adding buspirone to atypical antipsychotics in treating the negative symptoms of schizophrenic patients. Minerva Psichiatrica. 2018;59(1):22-8.

41. Bradley GM, Couchman GM, Perlesz A, Nguyen AT, Singh B, Riess C. Multiple-family group treatment for English- and Vietnamese-speaking families living with schizophrenia. Psychiatric Services. 2006;57(4):521-30.

42. Brand BA, de Boer JN, Marcelis MC, Grootens KP, Luykx JJ, Sommer IE. The Direct and Long-Term Effects of Raloxifene as Adjunctive Treatment for Schizophrenia-Spectrum Disorders: A Double-Blind, Randomized Clinical Trial. Schizophrenia Bulletin. 2023;49(6):1579-90.

43. Breier A, Liffick E, Hummer TA, Vohs JL, Yang Z, Mehdiyoun NF, et al. Effects of 12-month, double-blind N-acetyl cysteine on symptoms, cognition and brain morphology in early phase schizophrenia spectrum disorders. Schizophrenia Research. 2018;199:395-402.

44. Brown D, Daniels K, Pichereau S, Sand M. A Phase IC Study Evaluating the Safety, Tolerability, Pharmacokinetics, and Cognitive Outcomes of BI 409306 in Patients with Mild-to-Moderate Schizophrenia. Neurology and Therapy. 2018;7(1):129-39.

45. Brunelin J, Mondino M, Gassab L, Haesebaert F, Gaha L, Suaud-Chagny M-F, et al. Examining Transcranial Direct-Current Stimulation (tDCS) as a Treatment for Hallucinations in Schizophrenia. American Journal of Psychiatry. 2012;169(7):719-24.

46. Brunstein MG, Ghisolfi ES, Ramos FLP, Lara DR. A clinical trial of adjuvant allopurinol therapy for moderately refractory schizophrenia. Journal of Clinical Psychiatry. 2005;66(2):213-9.

47. Buchanan RW, Javitt DC, Marder SR, Schooler NR, Gold JM, McMahon RP, et al. The Cognitive and Negative Symptoms in Schizophrenia, Trial (CONSIST): The efficacy of glutamatergic agents for negative symptoms and cognitive impairments. American Journal of Psychiatry. 2007;164(10):1593-602.

48. Buchanan RW, Kelly D, Strauss GP, Gold JM, McMahon RP, Wehring HJ, et al. OXYTOCIN AND GALANTAMINE FOR THE TREATMENT OF NEGATIVE SYMPTOMS AND COGNITIVE IMPAIRMENTS IN SCHIZOPHRENIA. Schizophrenia Bulletin. 2015;41:S304-S5.

49. Buchanan RW, Kirkpatrick B, Bryant N, Ball P, Breier A. Fluoxetine augmentation of clozapine treatment in patients with schizophrenia. American Journal of Psychiatry. 1996;153(12):1625-7.

50. Bugarski-Kirola D, Arango C, Fava M, Nasrallah H, Liu IY, Abbs B, et al. Pimavanserin for negative symptoms of schizophrenia: results from the ADVANCE phase 2 randomised, placebo-controlled trial in North America and Europe. Lancet Psychiatry. 2022;9(1):46-58.

51. Bugarski-Kirola D, Wang A, Abi-Saab D, Blaettler T. A phase II/III trial of bitopertin monotherapy compared with placebo in patients with an acute exacerbation of schizophrenia - Results from the CandleLyte study. European Neuropsychopharmacology. 2014;24(7):1024-36.

52. Burton CZ, Vella L, Twamley EW. Clinical and cognitive insight in a compensatory cognitive training intervention. American journal of psychiatric rehabilitation. 2011;14(4):307-26.

53. Byrne LK, Peng D, McCabe M, Mellor D, Zhang J, Zhang T, et al. Does practice make perfect? Results from a Chinese feasibility study of cognitive remediation in schizophrenia. Neuropsychological Rehabilitation. 2013;23(4):580-96.

54. Caforio G, Di Giorgio A, Rampino A, Rizzo M, Romano R, Taurisano P, et al. Mirtazapine Add-On Improves Olanzapine Effect on Negative Symptoms of Schizophrenia. Journal of Clinical Psychopharmacology. 2013;33(6):810-2.

55. Cai C, Yu L, Rong L, Zhong H. Effectiveness of humor intervention for patients with schizophrenia: A randomized controlled trial. Journal of Psychiatric Research. 2014;59:174-8.

56. Carpenter WT, Breier A, Buchanan RW, Kirkpatrick B, Shepard P, Weiner E. Mazindol treatment of negative symptoms. Neuropsychopharmacology. 2000;23(4):365-74.

57. Casey DE, Sands EE, Heisterberg J, Yang H-M. Efficacy and safety of bifeprunox in patients with an acute exacerbation of schizophrenia: results from a randomized, double-blind, placebo-controlled, multicenter, dose-finding study. Psychopharmacology. 2008;200(3):317-31.

58. Chang JS, Ahn YM, Park HJ, Lee KY, Kim SH, Kang UG, et al. Aripiprazole augmentation in clozapine-treated patients with refractory schizophrenia: An 8-week, randomized, double-blind, placebo-controlled trial. Journal of Clinical Psychiatry. 2008;69(5):720-31.

59. Chaudhry IB, Hallak J, Husain N, Laganis C, Minhas F, Stirling J, et al. Minocycline benefits negative symptoms in early schizophrenia; A randomised double-blind placebo-controlled clinical trial in patients on a standard treatment. Early Intervention in Psychiatry. 2012;6:100-.

60. Chaudhry IB, Husain MO, Khoso AB, Husain MI, Buch MH, Kiran T, et al. A randomised clinical trial of methotrexate points to possible efficacy and adaptive immune dysfunction in psychosis. Translational Psychiatry. 2020;10(1).

61. Chaudhry IB, Husain MO, Khoso AB, Kiran T, Husain MI, Qurashi I, et al. Beneficial adjunctive effects of the 5HT3 receptor antagonist ondansetron on symptoms, function and cognition in early phase schizophrenia in a double-blind, 2 x 2 factorial design, randomised controlled comparison with simvastatin. Journal of Psychopharmacology. 2024;38(9):818-26.

62. Chaudhry IB, Husain N, Drake R, Dunn G, Husain MO, Kazmi A, et al. Add-on clinical effects of simvastatin and ondansetron in patients with schizophrenia stabilized on antipsychotic treatment: pilot study. Therapeutic advances in psychopharmacology. 2014;4(3):110-6.

63. Chen X-L, Deng X-T, Sun F-G, Huang Q-J. Effect of cognitive behavioral group therapy on rehabilitation of community patients with schizophrenia: A short-term randomized control trial. World Journal of Psychiatry. 2023;13(8):583-92.

64. Chen Z-h, Wang G-h, Wang X-p, Chen R-y, Wang H-l, Yang M-h, et al. Effects of warm-supplementing kidney yang (WSKY) capsule added on risperidone on cognition in chronic schizophrenic patients: a randomized, double-blind, placebo-controlled, multi-center clinical trial. Human Psychopharmacology-Clinical and Experimental. 2008;23(6):465-70.

65. Chen Z-h, Wang G-h, Wang X-p, Huo Y-x, Yang M-h, Li L, et al. Effect of Warm-Supplementing Kidney Yang (WSKY) added to risperidone on quality of life in patients with schizophrenia: a randomized controlled trial. Clinical Rehabilitation. 2009;23(11):963-72.

66. Cheng J, Wang G, Xiao L, Wang H, Wang X, Li C. Electro-acupuncture versus sham electro-acupuncture for auditory hallucinations in patients with schizophrenia: a randomized controlled trial. Clinical Rehabilitation. 2009;23(7):579-88.

67. Chengappa KNR, Brar JS, Gannon JM, Schlicht PJ. Adjunctive Use of a Standardized Extract of Withania somnifera (Ashwagandha) to Treat Symptom Exacerbation in Schizophrenia: A Randomized, Double-Blind, Placebo-Controlled Study. Journal of Clinical Psychiatry. 2018;79(5).

68. Chien WT, Bressington D, Yip A, Karatzias T. An international multi-site, randomized controlled trial of a mindfulness-based psychoeducation group programme for people with schizophrenia. Psychological Medicine. 2017;47(12):2081-96.

69. Chien WT, Mui JHC, Cheung EFC, Gray R. Effects of motivational interviewing-based adherence therapy for schizophrenia spectrum disorders: a randomized controlled trial. Trials. 2015;16.

70. Cho JM, Lee K. Effects of motivation interviewing using a group art therapy program on negative symptoms of schizophrenia. Archives of Psychiatric Nursing. 2018;32(6):878-84.

71. Choi K-H, Kang J, Kim S-M, Lee S-H, Park S-C, Lee W-H, et al. Cognitive remediation in middle-aged or older inpatients with chronic schizophrenia: A randomized controlled trial in Korea. Frontiers in psychology. 2018;8:2364.

72. Chouinard G, Jones B, Remington G, Bloom D, Addington D, MacEWAN GW, et al. A Canadian multicenter placebo-controlled study of fixed doses of risperidone and haloperidol in the treatment of chronic schizophrenic patients. LWW; 1993. p. 25-40.

73. Cohen JD, Vanputten T, Marder S, Berger PA, Stahl SM. THE EFFICACY OF PIQUINDONE, A NEW ATYPICAL NEUROLEPTIC, IN THE TREATMENT OF THE POSITIVE AND NEGATIVE SYMPTOMS OF SCHIZOPHRENIA. Journal of Clinical Psychopharmacology. 1987;7(5):324-9.

74. Cooper SJ, Butler A, Tweed J, Welch C, Raniwalla J. Zotepine in the prevention of recurrence: a randomised, double-blind, placebo-controlled study for chronic schizophrenia. Psychopharmacology. 2000;150(3):237-43.

75. Correll CU, Davis RE, Weingart M, Saillard J, O'Gorman C, Kane JM, et al. Efficacy and Safety of Lumateperone for Treatment of Schizophrenia A Randomized Clinical Trial. Jama Psychiatry. 2020;77(4):349-58.

76. Correll CU, Skuban A, Ouyang J, Hobart M, Pfister S, McQuade RD, et al. Efficacy and Safety of Brexpiprazole for the Treatment of Acute Schizophrenia: A 6-Week Randomized, Double-Blind, Placebo-Controlled Trial. American Journal of Psychiatry. 2015;172(9):870-80.

77. Crawford MJ, Killaspy H, Barnes TR, Barrett B, Byford S, Clayton K, et al. Group art therapy as an adjunctive treatment for people with schizophrenia: a randomised controlled trial (MATISSE). Health Technology Assessment. 2012;16(8):1-+.

78. Curcic D, Stojmenovic T, Djukic-Dejanovic S, Dikic N, MilicaVesic V, Radivojevic N, et al. POSITIVE IMPACT OF PRESCRIBED PHYSICAL ACTIVITY ON SYMPTOMS OF SCHIZOPHRENIA: RANDOMIZED CLINICAL TRIAL. Psychiatria Danubina. 2017;29(4):459-65.

79. Cutler AJ, Kalali AH, Weiden PJ, Hamilton J, Wolfgang CD. Four-week, double-blind, placebo- and ziprasidone-controlled trial of lloperidone in patients with acute exacerbations of schizophrenia. Journal of Clinical Psychopharmacology. 2008;28(2):S20-S8.

80. d'Amato T, Bation R, Cochet A, Jalenques I, Galland F, Giraud-Baro E, et al. A randomized, controlled trial of computer-assisted cognitive remediation for schizophrenia. schizophrenia Research. 2011;125(2-3):284-90.

81. Dagani J, Sisti D, Abelli M, Di Paolo L, Pini S, Raimondi S, et al. Do we need oxytocin to treat schizophrenia? A randomized clinical trial. Schizophrenia Research. 2016;172(1-3):158-64.

82. Dai Y, Ding H, Lu X, Wu X, Xu C, Jiang T, et al. CCRT and aerobic exercise: a randomised controlled study of processing speed, cognitive flexibility, and serum BDNF expression in schizophrenia. Schizophrenia (Heidelb). 2022;8(1):84.

83. Daniels L. A group cognitive-behavioral and process-oriented approach to treating the social impairment and negative symptoms associated with chronic mental illness. The Journal of psychotherapy practice and research. 1998;7(2):167-76.

84. Davidson M, Emsley R, Michelle K, Ford L, Pan G, Lim P, et al. Efficacy, safety and early response of paliperidone extended-release tablets (paliperidone ER): Results of a 6-week, randomized, placebo-controlled study. Schizophrenia Research. 2007;93(1-3):117-30.

85. Davidson M, Saoud J, Staner C, Noel N, Werner S, Luthringer E, et al. Efficacy and safety of roluperidone for the treatment of negative symptoms of schizophrenia. Schizophrenia bulletin. 2022;48(3):609-19.

86. De Lima DN, Jr., Costa Filho CWL, Frota IJ, de Oliveira ALB, Menezes CES, Chaves Filho AJM, et al. alpha-Lipoic Acid as Adjunctive Treatment for Schizophrenia: A Randomized Double-Blind Study. J Clin Psychopharmacol. 2023;43(1):39-45.

87. de Lucena D, Fernandes BS, Berk M, Dodd S, Medeiros DW, Pedrini M, et al. Improvement of Negative and Positive Symptoms in Treatment-Refractory Schizophrenia: A Double-Blind, Randomized, Placebo-Controlled Trial With Memantine as Add-On Therapy to Clozapine. Journal of Clinical Psychiatry. 2009;70(10):1416-23.

88. Den Boer JA, Vahlne JO, Post P, Heck AH, Daubenton F, Olbrich R. Ritanserin as add-on medication to neuroleptic therapy for patients with chronic or subchronic schizophrenia. Human Psychopharmacology-Clinical and Experimental. 2000;15(3):179-89.

89. Dickerson FB, Stallings CR, Boronow JJ, Origoni AE, Sullens A, Yolken RH. Double blind trial of adjunctive valacyclovir in individuals with schizophrenia who are seropositive for cytomegalovirus. Schizophrenia Research. 2009;107(2-3):147-9.

90. Ding N, Li Z, Liu Z. Escitalopram augmentation improves negative symptoms of treatment resistant schizophrenia patients - A randomized controlled trial. Neuroscience Letters. 2018;681:68-72.

91. Dlabac-de Lange JJ, Bais L, van Es FD, Visser BGJ, Reinink E, Bakker B, et al. Efficacy of bilateral repetitive transcranial magnetic stimulation for negative symptoms of schizophrenia: results of a multicenter double-blind randomized controlled trial. Psychological Medicine. 2015;45(6):1263-75.

92. Doruk A, Uzun O, Ozsahin A. A placebo-controlled study of extract of ginkgo biloba added to clozapine in patients with treatment-resistant schizophrenia. International Clinical Psychopharmacology. 2008;23(4):223-7.

93. Duncan EJ, Szilagyi S, Schwartz M, Kunzova A, Negi S, Stephanides M, et al. Effects of D-cycloserine on negative symptoms in schizophrenia. Biological Psychiatry. 2003;53(8):184S-S.

94. Emami M, Kheirabadi G, Fallahi M. The Effect of Lieberman Community Return Program on Reducing Positive and Negative Symptoms and Improving Social Skills in Patients with Schizophrenia. Advanced Biomedical Research. 2023;12(1).

95. Evins AE, Fitzgerald SM, Wine L, Rosselli R, Goff DC. Placebo-controlled trial of glycine added to clozapine in schizophrenia. American Journal of Psychiatry. 2000;157(5):826-8.

96. Fan F, Zou Y, Tan Y, Hong LE, Tan S. Computerized cognitive remediation therapy effects on resting state brain activity and cognition in schizophrenia. Scientific reports. 2017;7(1):4758.

97. Fan X, Liu E, Freudenreich O, Copeland P, Hayden D, Ghebremichael M, et al. No Effect of Adjunctive, Repeated-Dose Intranasal Insulin Treatment on Psychopathology and Cognition in Patients With Schizophrenia. Journal of Clinical Psychopharmacology. 2013;33(2):226-30.

98. Fan X, Song X, Zhao M, Jarskog LF, Natarajan R, Shukair N, et al. The effect of adjunctive telmisartan treatment on psychopathology and cognition in patients with schizophrenia. Acta Psychiatrica Scandinavica. 2017;136(5):465-72.

99. Farokhnia M, Azarkolah A, Adinehfar F, Khodaie-Ardakani M-R, Hosseini S-M-R, Yekehtaz H, et al. N-Acetylcysteine as an Adjunct to Risperidone for Treatment of Negative Symptoms in Patients With Chronic Schizophrenia: A Randomized, Double-Blind, Placebo-Controlled Study. Clinical Neuropharmacology. 2013;36(6):185-92.

100. Farokhnia M, Sabzabadi M, Pourmahmoud H, Khodaie-Ardakani M-R, Hosseini S-M-R, Yekehtaz H, et al. A double-blind, placebo controlled, randomized trial of riluzole as an adjunct to risperidone for treatment of negative symptoms in patients with chronic schizophrenia. Psychopharmacology. 2014;231(3):533-42.

101. Farooq S, Nazar Z, Irfan M, Akhter J, Gul E, Irfan U, et al. Schizophrenia medication adherence in a resource-poor setting: randomised controlled trial of supervised treatment in out-patients for schizophrenia (STOPS). British Journal of Psychiatry. 2011;199(6):467-72.

102. Favrod J, Nguyen A, Chaix J, Pellet J, Frobert L, Fankhauser C, et al. Improving Pleasure and Motivation in Schizophrenia: A Randomized Controlled Clinical Trial. Psychotherapy and Psychosomatics. 2019;88(2):84-95.

103. Fekete Z. Efficacy of the Metacognitive Training on symptom severity, neurocognition and social cognition in a sample of patients diagnosed with schizophrenia. Data Archiving and Networked Services (DANS-KNAW). 2021.

104. Findling RL, McKenna K, Earley WR, Stankowski J, Pathak S. Efficacy and Safety of Quetiapine in Adolescents with Schizophrenia Investigated in a 6-Week, Double-Blind, Placebo-Controlled Trial. Journal of Child and Adolescent Psychopharmacology. 2012;22(5):327-42.

105. Fiszdon J, Choi K, Bell M, Choi J, Silverstein S. Cognitive remediation for individuals with psychosis: efficacy and mechanisms of treatment effects. Psychological medicine. 2016;46(16):3275-89.

106. Fitzgerald PB, Herring S, Hoy K, McQueen S, Segrave R, Kulkarni J, et al. A study of the effectiveness of bilateral transcranial magnetic stimulation in the treatment of the negative symptoms of schizophrenia. Brain Stimulation. 2008;1(1):27-32.

107. Froehlich F, Burrello TN, Mellin JM, Cordle AL, Lustenberger CM, Gilmore JH, et al. Exploratory study of once-daily transcranial direct current stimulation (tDCS) as a treatment for auditory hallucinations in schizophrenia. European Psychiatry. 2016;33:54-60.

108. Gao H, Luo C, Tu S-J, Lu R-P, Jiang L-N, Qiao H-J, et al. The Effect of Yijinjing on the Cognitive Function of Patients With Chronic Schizophrenia. Frontiers in Psychiatry. 2021;12.

109. Garety PA, Craig TK, Dunn G, Fornells-Ambrojo M, Colbert S, Rahaman N, et al. Specialised care for early psychosis: symptoms, social functioning and patient satisfaction: randomised controlled trial. The British Journal of Psychiatry. 2006;188(1):37-45.

110. Garety PA, Fowler DG, Freeman D, Bebbington P, Dunn G, Kuipers E. Cognitive-behavioural therapy and family intervention for relapse prevention and symptom reduction in psychosis: randomised controlled trial. The British Journal of Psychiatry. 2008;192(6):412-23.

111. Garg S, Sinha VK, Tikka SK, Mishra P, Goyal N. The efficacy of cerebellar vermal deep high frequency (theta range) repetitive transcranial magnetic stimulation (rTMS) in schizophrenia: A randomized rater blind-sham controlled study. Psychiatry Research. 2016;243:413-20.

112. Ghajar A, Gholamian F, Tabatabei-Motlagh M, Afarideh M, Rezaei F, Ghazizadeh-Hashemi M, et al. Citicoline (CDP-choline) add-on therapy to risperidone for treatment of negative symptoms in patients with stable schizophrenia: A double-blind, randomized placebo-controlled trial. Human Psychopharmacology-Clinical and Experimental. 2018;33(4).

113. Ghajar A, Khoaie-Ardakani M-R, Shahmoradi Z, Alavi A-R, Afarideh M, Shalbafan M-R, et al. L-carnosine as an add-on to risperidone for treatment of negative symptoms in patients with stable schizophrenia: A double-blind, randomized placebo-controlled trial. Psychiatry Research. 2018;262:94-101.

114. Ghaleiha A, Noorbala AA, Farnaghi F, Hajiazim M, Akhondzadeh S. A Double-Blind, Randomized, and Placebo-Controlled Trial of Buspirone Added to Risperidone in Patients With Chronic Schizophrenia. Journal of Clinical Psychopharmacology. 2010;30(6):678-82.

115. Ghanizadeh A, Rezaee Z, Dehbozorgi S, Berk M, Akhondzadeh S. Lovastatin for the adjunctive treatment of schizophrenia: A preliminary randomized double-blind placebo-controlled trial. Psychiatry Research. 2014;219(3):431-5.

116. Gholipour A, Abolghasemi S, Gholinia K, Taheri S. Token Reinforcement Therapeutic Approach is More Effective than Exercise for Controlling Negative Symptoms of Schizophrenic Patients: A Randomized Controlled Trial. International journal of preventive medicine. 2012;3(7):466-70.

117. Goff DC. D-cycloserine treatment for negative symptoms in schizophrenia. Biological Psychiatry. 2005;57(8):5S-S.

118. Goff DC, Bagnell AL, Perlis RH. Glutamatergic augmentation strategies for cognitive impairment in schizophrenia. Psychiatric Annals. 1999;29(11):649-54.

119. Goff DC, Cather C, Gottlieb JD, Evins AE, Walsh J, Raeke L, et al. Once-weekly D-cycloserine effects on negative symptoms and cognition in schizophrenia: An exploratory study. Schizophrenia Research. 2008;106(2-3):320-7.

120. Goff DC, Freudenreich O, Cather C, Holt D, Bello I, Diminich E, et al. Citalopram in first episode schizophrenia: The DECIFER trial. Schizophrenia Research. 2019;208:331-7.

121. Goff DC, Keefe R, Citrome L, Davy K, Krystal JH, Large C, et al. Lamotrigine as add-on therapy in schizophrenia - Results of 2 placebo-controlled trials. Journal of Clinical Psychopharmacology. 2007;27(6):582-9.

122. Goff DC, Midha KK, Saridsegal O, Hubbard JW, Amico E. A PLACEBO-CONTROLLED TRIAL OF FLUOXETINE ADDED TO NEUROLEPTIC IN PATIENTS WITH SCHIZOPHRENIA. Psychopharmacology. 1995;117(4):417-23.

123. Gokcen A, Ekici G, Abaoglu H, Sen DT. The healing effect of goal-oriented dance and movement therapy in schizophrenia: A rater-blinded randomized controlled trial. Arts in Psychotherapy. 2020;71.

124. Gomes JS, Trevizol AP, Ducos DV, Gadelha A, Ortiz BB, Fonseca AO, et al. Effects of transcranial direct current stimulation on working memory and negative symptoms in schizophrenia: a phase II randomized sham-controlled trial. Schizophrenia research Cognition. 2018;12:20-8.

125. Govindaraj R, Naik SS, Mehta UM, Sharma M, Varambally S, Gangadhar BN. Yoga therapy for social cognition in schizophrenia: An experimental medicine-based randomized controlled trial. Asian Journal of Psychiatry. 2021;62.

126. Granholm E, McQuaid JR, McClure FS, Auslander LA, Perivoliotis D, Pedrelli P, et al. A randomized, controlled trial of cognitive behavioral social skills training for middle-aged and older outpatients with chronic schizophrenia. American Journal of Psychiatry. 2005;162(3):520-9.

127. Gu Y, Peng H, Dai J, Gao H, Yang X, Sheng J, et al. Evaluation of paliperidone on social function in patients with chronic schizophrenia. General Psychiatry. 2018;31(2).

128. Guan HY, Zhao JM, Wang KQ, Su XR, Pan YF, Guo JM, et al. High-frequency neuronavigated rTMS effect on clinical symptoms and cognitive dysfunction: a pilot double-blind, randomized controlled study in Veterans with schizophrenia. Translational Psychiatry. 2020;10(1).

129. Gumley A, O'Grady M, McNay L, Reilly J, Power K, Norrie J. Early intervention for relapse in schizophrenia: results of a 12-month randomized controlled trial of cognitive behavioural therapy. Psychological Medicine. 2003;33(3):419-31.

130. Gunduz-Bruce H, Oliver S, Gueorguieva R, Forselius-Bielen K, D'Souza DC, Zimolo Z, et al. Efficacy of pimozide augmentation for clozapine partial responders with schizophrenia. Schizophrenia Research. 2013;143(2-3):344-7.

131. Haas M, Unis AS, Armenteros J, Copenhaver MD, Quiroz JA, Kushner SF. A 6-Week, Randomized, Double-Blind, Placebo-Controlled Study of the Efficacy and Safety of Risperidone in Adolescents with Schizophrenia. Journal of Child and Adolescent Psychopharmacology. 2009;19(6):611-21.

132. Hajak G, Marienhagen J, Langguth B, Werner S, Binder H, Eichhammer P. High-frequency repetitive transcranial magnetic stimulation in schizophrenia: a combined treatment and neuroimaging study. Psychological Medicine. 2004;34(7):1157-63.

133. Hansen JP, Østergaard B, Nordentoft M, Hounsgaard L. Cognitive adaptation training combined with assertive community treatment: a randomised longitudinal trial. Schizophrenia research. 2012;135(1-3):105-11.

134. Hassanpour F, Zarghami M, Mouodi S, Moosazadeh M, Barzegar F, Bagheri M, et al. Adjunctive Memantine Treatment of Schizophrenia A Double-Blind, Randomized Placebo-Controlled Study. Journal of Clinical Psychopharmacology. 2019;39(6):634-8.

135. Hasson-Ohayon I, Igra L, Lavi-Rotenberg A, Goldzweig G, Lysaker PH. Findings from a randomized controlled trial of Metacognitive Reflection and Insight Therapy for people with schizophrenia: Effects on metacognition and symptoms. Psychology and Psychotherapy-Theory Research and Practice. 2024;97:75-90.

136. Hayashi T, Yokota N, Takahashi T, Tawara Y, Nishikawa T, Yano T, et al. Benefits of trazodone and mianserin for patients with late-life chronic schizophrenia and tardive dyskinesia: an add-on, double-blind, placebo-controlled study. International Clinical Psychopharmacology. 1997;12(4):199-205.

137. Hayes RL, Halford WK, Varghese FT. SOCIAL SKILLS TRAINING WITH CHRONIC-SCHIZOPHRENIC PATIENTS - EFFECTS ON NEGATIVE SYMPTOMS AND COMMUNITY FUNCTIONING. Behavior Therapy. 1995;26(3):433-49.

138. He H, Li T, Zhou F, Yang Q, Hu L, Yu Y. The Therapeutic Effect of Edible Horticultural Therapy on Extrapyramidal Symptoms in Patients with Schizophrenia. Hortscience. 2021;56(9):1125-9.

139. Hegde S, Rao SL, Raguram A, Gangadhar BN. Addition of home-based cognitive retraining to treatment as usual in first episode schizophrenia patients: A randomized controlled study. Indian journal of psychiatry. 2012;54(1):15-22.

140. Heresco-Levy U, Javitt DC, Ebstein R, Vass A, Lichtenberg P, Bar G, et al. D-serine efficacy as add-on pharmacotherapy to risperidone and olanzapine for treatment-refractory schizophrenia. Biological Psychiatry. 2005;57(6):577-85.

141. Herizchi S, Shafiee-Kandjani AR, Farahbakhsh M, Jahangiri Z, Ghanbarzadeh Javid S, Azizi H. Efficacy of Rivastigmine Augmentation on Positive and Negative Symptoms, General Psychopathology, and Quality of Life in Patients with Chronic Schizophrenia: A Randomized Controlled Trial. Psychopharmacology bulletin. 2024;54(2):15-27.

142. Hill M, Shannahan K, Jasinski S, Macklin EA, Raeke L, Roffman JL, et al. Folate supplementation in schizophrenia: A possible role for MTHFR genotype. Schizophrenia Research. 2011;127(1-3):41-5.

143. Hinkelmann K, Yassouridis A, Kellner M, Jahn H, Wiedemann K, Raedler TJ. No Effects of Antidepressants on Negative Symptoms in Schizophrenia. Journal of Clinical Psychopharmacology. 2013;33(5):686-90.

144. Hirayasu Y, Sato S-I, Shuto N, Nakano M, Higuchi T. Efficacy and Safety of Bitopertin in Patients with Schizophrenia and Predominant Negative Symptoms: Subgroup Analysis of Japanese Patients from the Global Randomized Phase 2 Trial. Psychiatry Investigation. 2017;14(1):63-73.

145. Holi MM, Eronen M, Toivonen K, Toivonen P, Marttunen M, Naukkarinen H. Left prefrontal repetitive transcranial magnetic stimulation in schizophrenia. Schizophrenia Bulletin. 2004;30(2):429-34.

146. Honer WG, Thornton AE, Chen EYH, Chan RCK, Wong JOY, Bergmann A, et al. Clozapine alone versus clozapine and risperidone with refractory schizophrenia. New England Journal of Medicine. 2006;354(5):472-82.

147. Horiguchi J, Wake R, Murotani K, Seno H, Miyaoka T, Inoue K. A multicenter, double-blind, randomized, controlled study of patients with treatment-resistant schizophrenia treated with yokukansan for 12 weeks. Psychiatry and Clinical Neurosciences Reports. 2023;2(4).

148. Hosseini SMR, Farokhnia M, Rezaei F, Gougol A, Yekehtaz H, Iranpour N, et al. Intranasal desmopressin as an adjunct to risperidone for negative symptoms of schizophrenia: A randomized, double-blind, placebo-controlled, clinical trial. European Neuropsychopharmacology. 2014;24(6):846-55.

149. Hosseininasab M, Zarghami M, Mazhari S, Salehifar E, Moosazadeh M, Fariborzifar A, et al. Nanocurcumin as an Add-on to Antipsychotic Drugs for Treatment of Negative Symptoms in Patients With Chronic Schizophrenia A Randomized, Double-Blind, Placebo-Controlled Study. Journal of Clinical Psychopharmacology. 2021;41(1):25-30.

150. Hu Q, Jiao X, Zhou J, Tang Y, Zhang T, Song C, et al. Low-frequency repetitive transcranial magnetic stimulation over the right orbitofrontal cortex for patients with first-episode schizophrenia: A randomized, double-blind, sham-controlled trial. Psychiatry Research. 2023;330.

151. Husain MO, Chaudhry IB, Khoso AB, Husain MI, Ansari MA, Mehmood N, et al. Add-on Sodium Benzoate and N-Acetylcysteine in Patients With Early Schizophrenia Spectrum Disorder: A Multicenter, Double-Blind, Randomized Placebo-Controlled Feasibility Trial. Schizophrenia bulletin open. 2024;5(1):sgae004-sgae.

152. Husain MO, Chaudhry IB, Mehmood N, Rehman Ru, Kazmi A, Hamirani M, et al. Pilot randomised controlled trial of culturally adapted cognitive behavior therapy for psychosis (CaCBTp) in Pakistan. Bmc Health Services Research. 2017;17.

153. Iancu I, Tschernihovsky E, Bodner E, Piconne AS, Lowengrub K. Escitalopram in the treatment of negative symptoms in patients with chronic schizophrenia: A randomized double-blind placebo-controlled trial. Psychiatry Research. 2010;179(1):19-23.

154. Ikai S, Suzuki T, Uchida H, Saruta J, Tsukinoki K, Fujii Y, et al. Effects of weekly one-hour Hatha yoga therapy on resilience and stress levels in patients with schizophrenia-spectrum disorders: an eight-week randomized controlled trial. The Journal of Alternative and Complementary Medicine. 2014;20(11):823-30.

155. Ikai S, Uchida H, Mizuno Y, Tani H, Nagaoka M, Tsunoda K, et al. Effects of chair yoga therapy on physical fitness in patients with psychiatric disorders: A 12-week single-blind randomized controlled trial. Journal of Psychiatric Research. 2017;94:194-201.

156. Iranpour N, Zandifar A, Farokhnia M, Goguol A, Yekehtaz H, Khodaie-Ardakani M-R, et al. The effects of pioglitazone adjuvant therapy on negative symptoms of patients with chronic schizophrenia: a double-blind and placebo-controlled trial. Human Psychopharmacology-Clinical and Experimental. 2016;31(2):103-12.

157. Ishoy PL, Fagerlund B, Broberg BV, Bak N, Knop FK, Glenthoj BY, et al. No cognitive-enhancing effect of GLP-1 receptor agonism in antipsychotic-treated, obese patients with schizophrenia. Acta Psychiatrica Scandinavica. 2017;136(1):52-62.

158. Iwata K, Matsuda Y, Sato S, Furukawa S, Watanabe Y, Hatsuse N, et al. Efficacy of cognitive rehabilitation using computer software with individuals living with schizophrenia: A randomized controlled trial in Japan. Psychiatric Rehabilitation Journal. 2017;40(1):4.

159. Iwata N, Ishigooka J, Kim W-H, Yoon B-H, Lin S-K, Sulaiman AH, et al. Efficacy and safety of blonanserin transdermal patch in patients with schizophrenia: A 6-week randomized, double-blind, placebo-controlled, multicenter study. Schizophrenia Research. 2020;215:408-15.

160. Jamilian H, Solhi H, Jamilian M. Randomized, placebo-controlled clinical trial of omega-3 as supplemental treatment in schizophrenia. Global journal of health science. 2014;6(7 Spec No):103-8.

161. Jarskog LF, Pedersen CA, Johnson JL, Hamer RM, Rau SW, Elliott T, et al. A 12-week randomized controlled trial of twice-daily intranasal oxytocin for social cognitive deficits in people with schizophrenia. Schizophrenia Research. 2017;185:88-95.

162. Javadi AHS, Shafikhani AA, Zamir SM, Khanshir ZF. Evaluation of the Effect of Fluvoxamine in Patients With Schizophrenia Under Risperidone Treatment: A Clinical Trial. Journal of Clinical Psychopharmacology. 2018;38(2):119-24.

163. Jayaram N, Varambally S, Behere RV, Venkatasubramanian G, Arasappa R, Christopher R, et al. Effect of yoga therapy on plasma oxytocin and facial emotion recognition deficits in patients of schizophrenia. Indian Journal of Psychiatry. 2013;55(7):S409-S13.

164. Jeon D-W, Jung D-U, Kim S-J, Shim J-C, Moon J-J, Seo Y-S, et al. Adjunct transcranial direct current stimulation improves cognitive function in patients with schizophrenia: A double-blind 12-week study. Schizophrenia Research. 2018;197:378-85.

165. Jin Y, Tong J, Huang Y, Shi D, Zhu N, Zhu M, et al. Effectiveness of accelerated intermittent theta burst stimulation for social cognition and negative symptoms among individuals with schizophrenia: A randomized controlled trial. Psychiatry Res. 2023;320:115033.

166. Jockers-Scherübl M, Bauer A, Godemann F, Reischies F, Selig F, Schlattmann P. Negative symptoms of schizophrenia are improved by the addition of paroxetine to neuroleptics: A double-blind placebo-controlled study. Pharmacopsychiatry. 2003;36(05):139.

167. Joffe G, Terevnikov V, Joffe M, Stenberg J-H, Burkin M, Tiihonen J. Add-on mirtazapine enhances antipsychotic effect of first generation antipsychotics in schizophrenia: A double-blind, randomized, placebo-controlled trial. Schizophrenia Research. 2009;108(1-3):245-51.

168. Kalejahi P, Kheirouri S, Noorazar SG. Effect of Vitamin D Supplementation on Cardio-metabolic Indices and the Severity of Symptoms in Male Patients With Chronic Schizophrenia. Crescent Journal of Medical and Biological Sciences. 2023;10(3):125-31.

169. Kaltsatou A, Kouidi E, Fountoulakis K, Sipka C, Theochari V, Kandylis D, et al. Effects of exercise training with traditional dancing on functional capacity and quality of life in patients with schizophrenia: a randomized controlled study. Clinical rehabilitation. 2015;29(9):882-91.

170. Kane JM, Carson WH, Saha AR, McQuade RD, Ingenito GG, Zimbroff DL, et al. Efficacy and safety of aripiprazole and haloperidol versus placebo in patients with schizophrenia and schizoaffective disorder. Journal of Clinical Psychiatry. 2002;63(9):763-71.

171. Kane JM, Cohen M, Zhao J, Alphs L, Panagides J. Efficacy and Safety of Asenapine in a Placebo- and Haloperidol-Controlled Trial in Patients With Acute Exacerbation of Schizophrenia. Journal of Clinical Psychopharmacology. 2010;30(2):106-15.

172. Kane JM, D'Souza DC, Patkar AA, Youakim JM, Tiller JM, Yang R, et al. Armodafinil as Adjunctive Therapy in Adults With Cognitive Deficits Associated With Schizophrenia: A 4-Week, Double-Blind, Placebo-Controlled Study. Journal of Clinical Psychiatry. 2010;71(11):1475-81.

173. Kane JM, Eerdekens M, Lindenmayer JP, Keith SJ, Lesem M, Karcher K. Long-acting injectable risperidone: Efficacy and safety of the first long-acting atypical antipsychotic. American Journal of Psychiatry. 2003;160(6):1125-32.

174. Kang R, Wu Y, Li Z, Jiang J, Gao Q, Yu Y, et al. Effect of Community-Based Social Skills Training and Tai-Chi Exercise on Outcomes in Patients with Chronic Schizophrenia: A Randomized, One-Year Study. Psychopathology. 2016;49(5):345-55.

175. Kanie A, Kikuchi A, Haga D, Tanaka Y, Ishida A, Yorozuya Y, et al. The feasibility and efficacy of social cognition and interaction training for outpatients with schizophrenia in Japan: a multicenter randomized clinical trial. Frontiers in Psychiatry. 2019;10:589.

176. Kantrowitz JT, Javitt DC, Freedman R, Sehatpour P, Kegeles LS, Carlson M, et al. Double blind, two dose, randomized, placebo-controlled, cross-over clinical trial of the positive allosteric modulator at the alpha7 nicotinic cholinergic receptor AVL-3288 in schizophrenia patients. Neuropsychopharmacology. 2020;45(8):1339-45.

177. Kaphzan H, Ben-Shachar D, Klein E. Entacapone augmentation of antipsychotic treatment in schizophrenic patients with negative symptoms; a double-blind placebo-controlled study. International Journal of Neuropsychopharmacology. 2014;17(2):337-40.

178. Karaman IGY, Kasal MI, Ingec C, Yastibas C, Gulyuksel F, Gulec M. Effect of Adjunct Psychosocial Skills Training on Social Functioning of Schizophrenia Patients Who Get Occupational Therapy in a Community Mental Health Center: A Comparative Study. Noropsikiyatri Arsivi-Archives of Neuropsychiatry. 2020;57(3):248-53.

179. Karbalaee M, Jameie M, Amanollahi M, TaghaviZanjani F, Parsaei M, Basti FA, et al. Efficacy and safety of adjunctive therapy with fingolimod in patients with schizophrenia: A randomized, double-blind, placebo-controlled clinical trial. Schizophr Res. 2023;254:92-8.

180. Kardashev A, Ratner Y, Ritsner MS. Add-On Pregnenolone with L-Theanine to Antipsychotic Therapy Relieves Negative and Anxiety Symptoms of Schizophrenia: An 8-Week, Randomized, Double-Blind, Placebo-Controlled Trial. Clinical schizophrenia & related psychoses. 2018;12(1):31-41.

181. Kasckow J, Fellows I, Golshan S, Solorzano E, Meeks T, Zisook S. Treatment of Subsyndromal Depressive Symptoms in Middle-Age and Older Patients With Schizophrenia: Effect of Age on Response. American Journal of Geriatric Psychiatry. 2010;18(9):853-7.

182. Kashani L, Shams N, Moazen-Zadeh E, Karkhaneh-Yousefi M-A, Sadighi G, Khodaie-Ardakani M-R, et al. Pregnenolone as an adjunct to risperidone for treatment of women with schizophrenia: A randomized double-blind placebo-controlled clinical trial. Journal of Psychiatric Research. 2017;94:70-7.

183. Katsumi A, Hoshino H, Fujimoto S, Yabe H, Ikebuchi E, Nakagome K, et al. Effects of cognitive remediation on cognitive and social functions in individuals with schizophrenia. Neuropsychological rehabilitation. 2017.

184. Kayo M, Scemes S, Savoia MG, Bichuette A, Abreu AC, dA Silva EP, et al. A randomized controlled trial of social skills training for patients with treatment-resistant schizophrenia with predominantly negative symptoms. Psychiatry Research. 2020;287.

185. Keck P, Buffenstein A, Ferguson J, Feighner J, Jaffe W, Harrigan EP, et al. Ziprasidone 40 and 120 mg/day in the acute exacerbation of schizophrenia and schizoaffective disorder: a 4-week placebo controlled trial. Psychopharmacology. 1998;140(2):173-84.

186. Kelly DL, Sullivan KM, McEvoy JP, McMahon RP, Wehring HJ, Gold JM, et al. Adjunctive Minocycline in Clozapine-Treated Schizophrenia Patients With Persistent Symptoms. Journal of Clinical Psychopharmacology. 2015;35(4):374-81.

187. Khalil AH, Elnahas G, Ramy H, Aziz KA, Elkholy H, El-Ghamry R. Impact of a culturally adapted behavioural family psychoeducational programme in patients with schizophrenia in Egypt. International Journal of Psychiatry in Clinical Practice. 2019;23(1):62-71.

188. Khodaie-Ardakani M-R, Khosravi M, Zarinfard R, Nejati S, Mohsenian A, Tabrizi M, et al. A Placebo-Controlled Study of Raloxifene Added to Risperidone in Men with Chronic Schizophrenia. Acta medica Iranica. 2015;53(6):337-45.

189. Khodaie-Ardakani M-R, Mirshafiee O, Farokhnia M, Tajdini M, Hosseini S-M-R, Modabbernia A, et al. Minocycline add-on to risperidone for treatment of negative symptoms in patients with stable schizophrenia: Randomized double-blind placebo-controlled study. Psychiatry Research. 2014;215(3):540-6.

190. Khodaie-Ardakani M-R, Seddighi S, Modabbernia A, Rezaei F, Salehi B, Ashrafi M, et al. Granisetron as an add-on to risperidone for treatment of negative symptoms in patients with stable schizophrenia: Randomized double-blind placebo-controlled study. Journal of Psychiatric Research. 2013;47(4):472-8.

191. Khonsari NM, Badrfam R, Mohammdi MR, Rastad H, Etemadi F, Vafaei Z, et al. Effect of Aerobic Exercise as Adjunct Therapy on the Improvement of Negative Symptoms and Cognitive Impairment in Patients With Schizophrenia. Journal of Psychosocial Nursing and Mental Health Services. 2021.

192. Kianimehr G, Fatehi F, Hashempoor S, Khodaei-Ardakani M-R, Rezaei F, Nazari A, et al. Raloxifene adjunctive therapy for postmenopausal women suffering from chronic schizophrenia: a randomized double-blind and placebo controlled trial. Daru-Journal of Pharmaceutical Sciences. 2014;22.

193. Kimhy D, Tay C, Vakhrusheva J, Beck-Felts K, Ospina LH, Ifrah C, et al. Enhancement of aerobic fitness improves social functioning in individuals with schizophrenia. European Archives of Psychiatry and Clinical Neuroscience. 2021;271(2):367-76.

194. Kirkpatrick B, Saoud JB, Strauss GP, Ahmed AO, Tatsumi K, Opler M, et al. The brief negative symptom scale (BNSS): Sensitivity to treatment effects. Schizophrenia Research. 2018;197:269-73.

195. Klein E, Kolsky Y, Puyerovsky M, Koren D, Chistyakov A, Feinsod M. Right prefrontal slow repetitive transcranial magnetic stimulation in schizophrenia: A double-blind sham-controlled pilot study. Biological Psychiatry. 1999;46(10):1451-4.

196. Ko Y-H, Lew Y-M, Jung S-W, Joe S-H, Lee C-H, Jung H-G, et al. Short-term testosterone augmentation in male schizophrenics - A randomized, double-blind, placebo-controlled trial. Journal of Clinical Psychopharmacology. 2008;28(4):375-83.

197. Kong Y, Min H, Zhu X, Zhang L, Hu J. Clinical study of dance art therapy on hospitalized patients with chronic schizophrenia. Medicine. 2024;103(24).

198. Kopelowicz A, Zarate R, Smith VG, Mintz J, Liberman RP. Disease management in Latinos with schizophrenia: A family-assisted, skills training approach. Schizophrenia Bulletin. 2003;29(2):211-28.

199. Kos C, Bais L, Klaasen N, Opmeer E, Liemburg E, Wardenaar KJ, et al. Effects of right prefrontal theta-burst transcranial magnetic stimulation or transcranial direct current stimulation on apathy in patients with schizophrenia: A multicenter RCT. Psychiatry Research. 2024;333.

200. Krakvik B, Grawe RW, Hagen R, Stiles TC. Cognitive Behaviour Therapy for Psychotic Symptoms: A Randomized Controlled Effectiveness Trial. Behavioural and Cognitive Psychotherapy. 2013;41(5):511-24.

201. Kremer I, Vass A, Gorelik I, Bar G, Blanaru M, Javitt DC, et al. Placebo-controlled trial of lamotrigine added to conventional and atypical antipsychotics in schizophrenia. Biological Psychiatry. 2004;56(6):441-6.

202. Kruiper C, Sommer IEC, Koster M, Bakker PR, Durston S, Oranje B. Clonidine augmentation in patients with schizophrenia: A double-blind, randomized placebo-controlled trial. Schizophrenia Research. 2023;255:148-54.

203. Kucerova HP, Prikryl R, Ustohal L. Does repetitive transcranial magnetic stimulation have a positive effect on working memory and neuronal activation in treatment of negative symptoms of schizophrenia? International Journal of Neuropsychopharmacology. 2012;15:130-.

204. Kulkarni J, de Castella A, Fitzgerald PB, Gurvich CT, Bailey M, Bartholomeusz C, et al. Estrogen in severe mental illness - A potential new treatment approach. Archives of General Psychiatry. 2008;65(8):955-60.

205. Kulkarni J, Gavrilidis E, Gwini SM, Worsley R, Grigg J, Warren A, et al. Effect of Adjunctive Raloxifene Therapy on Severity of Refractory Schizophrenia in Women A Randomized Clinical Trial. Jama Psychiatry. 2016;73(9):947-54.

206. Kulkarni J, Gavrilidis E, Wang W, Worsley R, Fitzgerald PB, Gurvich C, et al. Estradiol for treatment-resistant schizophrenia: a large-scale randomized-controlled trial in women of child-bearing age. Molecular Psychiatry. 2015;20(6):695-702.

207. Kumar N, Vishnubhatla S, Wadhawan AN, Minhas S, Gupta P. A randomized, double blind, sham-controlled trial of repetitive transcranial magnetic stimulation (rTMS) in the treatment of negative symptoms in schizophrenia. Brain Stimulation. 2020;13(3):840-9.

208. Laan W, Grobbee DE, Selten J-P, Heijnen CJ, Kahn RS, Burger H. Adjuvant Aspirin Therapy Reduces Symptoms of Schizophrenia Spectrum Disorders: Results From a Randomized, Double-Blind, Placebo-Controlled Trial. Journal of Clinical Psychiatry. 2010;71(5):520-7.

209. Lane H-Y, Huang C-L, Wu P-L, Liu Y-C, Chang Y-C, Lin P-Y, et al. Glycine transporter I inhibitor, N-methylglycine (Sarcosine), added to clozapine for the treatment of schizophrenia. Biological Psychiatry. 2006;60(6):645-9.

210. Lane H-Y, Lin C-H, Green MF, Hellemann G, Huang C-C, Chen P-W, et al. Add-on Treatment of Benzoate for Schizophrenia A Randomized, Double-blind, Placebo-Controlled Trial of D-Amino Acid Oxidase Inhibitor. Jama Psychiatry. 2013;70(12):1267-75.

211. Lane H-Y, Lin C-H, Huang Y-J, Liao C-H, Chang Y-C, Tsai GE. A randomized, double-blind, placebo-controlled comparison study of sarcosine (N-methylglycine) and D-serine add-on treatment for schizophrenia. International Journal of Neuropsychopharmacology. 2010;13(4):451-60.

212. Lane HY, Chang YC, Liu YC, Chiu CC, Tsai GE. Sarcosine or D-serine add-on treatment for acute exacerbation of schizophrenia - A randomized, double-blind, placebo-controlled study. Archives of General Psychiatry. 2005;62(11):1196-204.

213. Lauriello J, Lambert T, Andersen S, Lin D, Taylor CC, McDonnell D. An 8-week, double-blind, randomized, placebo-controlled study of olanzapine long-acting injection in acutely ill patients with schizophrenia. Journal of Clinical Psychiatry. 2008;69(5):790.

214. Le Moigne A, Csernansky J, Leadbetter RA, Andorn AC, Graham JA, Heath AT, et al. PANSS Individual Item and Marder Dimension Analyses From a Pivotal Trial of RBP-7000 (Monthly Extended-Release Risperidone) in Schizophrenia Patients. Journal of Clinical Psychiatry. 2021;82(5).

215. Lecrubier Y, Quintin P, Bouhassira M, Perrin E, Lancrenon S. The treatment of negative symptoms and deficit states of chronic schizophrenia: olanzapine compared to amisulpride and placebo in a 6-month double-blind controlled clinical trial. Acta Psychiatrica Scandinavica. 2006;114(5):319-27.

216. Lee BJ, Lee SJ, Kim MK, Lee JG, Park SW, Kim GM, et al. Effect of Aripiprazole on Cognitive Function and Hyperprolactinemia in Patients with Schizophrenia Treated with Risperidone. Clinical Psychopharmacology and Neuroscience. 2013;11(2):60-6.

217. Lee H-J, Jang S-H, Lee S-Y, Hwang K-S. Effectiveness of dance/movement therapy on affect and psychotic symptoms in patients with schizophrenia. Arts in Psychotherapy. 2015;45:64-8.

218. Lee JG, Lee SW, Lee BJ, Park SW, Kim GM, Kim YH. Adjunctive Memantine Therapy for Cognitive Impairment in Chronic Schizophrenia: A Placebo-Controlled Pilot Study. Psychiatry Investigation. 2012;9(2):166-73.

219. Lee K-H. A randomized controlled trial of mindfulness in patients with schizophrenia. Psychiatry Research. 2019;275:137-42.

220. Lee MR, Wehring HJ, McMahon RP, Linthicum J, Cascella N, Liu F, et al. Effects of adjunctive intranasal oxytocin on olfactory identification and clinical symptoms in schizophrenia: Results from a randomized double blind placebo controlled pilot study. Schizophrenia Research. 2013;145(1-3):110-5.

221. Lee MS, Kim YK, Lee SK, Suh KY. A double-blind study of adjunctive sertraline in haloperidol-stabilized patients with chronic schizophrenia. Journal of Clinical Psychopharmacology. 1998;18(5):399-403.

222. Lee S-Y, Chen S-L, Chang Y-H, Chen P-S, Huang S-Y, Tzeng N-S, et al. ALDH2 polymorphism, associated with attenuating negative symptoms in patients with schizophrenia treated with add-on dextromethorphan. Journal of Psychiatric Research. 2015;69:50-6.

223. Lerner V, Miodownik C, Gibel A, Sirota P, Bush I, Elliot H, et al. The Retinoid X Receptor Agonist Bexarotene Relieves Positive Symptoms of Schizophrenia: A 6-Week, Randomized, Double-Blind, Placebo-Controlled Multicenter Trial. Journal of Clinical Psychiatry. 2013;74(12):1224-32.

224. Levkovitz Y, Mendlovich S. A Double Blind Randomized Study of Minocycline for the Treatment of Negative and Cognitive Symptoms in Early-Phase Schizophrenia. Biological Psychiatry. 2009;65(8):91S-S.

225. Li H, Guo W, Liu F, Chen J, Su Q, Zhang Z, et al. Enhanced baseline activity in the left ventromedial putamen predicts individual treatment response in drug-naive, first-episode schizophrenia: Results from two independent study samples. Ebiomedicine. 2019;46:248-55.

226. Li J, Ye F, Xiao W, Tang X, Sha W, Zhang X, et al. Increased serum brain-derived neurotrophic factor levels following electroconvulsive therapy or antipsychotic treatment in patients with schizophrenia. European Psychiatry. 2016;36:23-8.

227. Li M, Lang B. The effects of systematic psychological nursing on the sleep quality of schizophrenic patients with sleep disorders. American Journal of Translational Research. 2021;13(6):7263-9.

228. Li M, Qiu Y, Zhang J, Zhang Y, Liu Y, Zhao Y, et al. Improvement of adjunctive berberine treatment on negative symptoms in patients with schizophrenia. European Archives of Psychiatry and Clinical Neuroscience. 2022.

229. Li X, Xiang Q, Cen H, Zhai Z, Gao T, Lu C, et al. Efficacy of Cortical-Hippocampal Target Intermittent Theta Burst Stimulation (iTBS) on Associative Memory of Schizophrenia: A Double-Blind, Randomized Sham-Controlled Trial. Neuropsychiatric Disease and Treatment. 2024;20:1941-55.

230. Lieberman JA, Papadakis K, Csernansky J, Litman R, Volavka J, Jia XD, et al. A Randomized, Placebo-Controlled Study of Memantine as Adjunctive Treatment in Patients with Schizophrenia. Neuropsychopharmacology. 2009;34(5):1322-9.

231. Lin C-H, Lin C-H, Chang Y-C, Huang Y-J, Chen P-W, Yang H-T, et al. Sodium Benzoate, a D-Amino Acid Oxidase Inhibitor, Added to Clozapine for the Treatment of Schizophrenia: A Randomized, Double-Blind, Placebo-Controlled Trial. Biological Psychiatry. 2018;84(6):422-32.

232. Lin C-Y, Liang S-Y, Chang Y-C, Ting S-Y, Kao C-L, Wu Y-H, et al. Adjunctive sarcosine plus benzoate improved cognitive function in chronic schizophrenia patients with constant clinical symptoms: A randomised, double-blind, placebo-controlled trial. World Journal of Biological Psychiatry. 2017;18(5):357-68.

233. Lincoln TM, Ziegler M, Mehl S, Kesting M-L, Luellmann E, Westermann S, et al. Moving From Efficacy to Effectiveness in Cognitive Behavioral Therapy for Psychosis: A Randomized Clinical Practice Trial. Journal of Consulting and Clinical Psychology. 2012;80(4):674-86.

234. Lindenmayer J, Kulsa MKC, Sultana T, Kaur A, Yang R, Ljuri I, et al. Transcranial direct-current stimulation in ultra-treatment-resistant schizophrenia. Brain stimulation. 2019;12(1):54-61.

235. Lisoni J, Baldacci G, Nibbio G, Zucchetti A, Butti Lemmi Gigli E, Savorelli A, et al. Effects of bilateral, bipolar-nonbalanced, frontal transcranial Direct Current Stimulation (tDCS) on negative symptoms and neurocognition in a sample of patients living with schizophrenia: Results of a randomized double-blind sham-controlled trial. J Psychiatr Res. 2022;155:430-42.

236. Litman RE, Smith MA, Desai DG, Simpson T, Sweitzer D, Kanes SJ. The Selective Neurokinin 3 Antagonist AZD2624 Does Not Improve Symptoms or Cognition in Schizophrenia A Proof-of-Principle Study. Journal of Clinical Psychopharmacology. 2014;34(2):199-204.

237. Liu F, Guo X, Wu R, Ou J, Zheng Y, Zhang B, et al. Minocycline supplementation for treatment of negative symptoms in early-phase schizophrenia: A double blind, randomized, controlled trial. Schizophrenia Research. 2014;153(1-3):169-76.

238. Lohr JB, Liu L, Caligiuri MP, Kash TP, May TA, Murphy JD, et al. Modafinil improves antipsychotic-induced parkinsonism but not excessive daytime sleepiness, psychiatric symptoms or cognition in schizophrenia and schizoaffective disorder: A randomized, double-blind, placebo-controlled study. Schizophrenia Research. 2013;150(1):289-96.

239. Longden E, Corstens D, Bowe S, Pyle M, Emsley R, Peters S, et al. A psychological intervention for engaging dialogically with auditory hallucinations (Talking With Voices): A single-site, randomised controlled feasibility trial. Schizophr Res. 2022;250:172-9.

240. Loo H, PoirierLittre MF, Theron M, Rein W, Fleurot O. Amisulpride versus placebo in the medium-term treatment of the negative symptoms of schizophrenia. British Journal of Psychiatry. 1997;170:18-22.

241. Lopez-Navarro E, Del Canto C, Belber M, Mayol A, Fernandez-Alonso O, Lluis J, et al. Mindfulness improves psychological quality of life in community-based patients with severe mental health problems: A pilot randomized clinical trial. Schizophrenia Research. 2015;168(1-2):530-6.

242. Lu S-F, Lo C-HK, Sung H-C, Hsieh T-C, Yu S-C, Chang S-C. Effects of group music intervention on psychiatric symptoms and depression in patient with schizophrenia. Complementary Therapies in Medicine. 2013;21(6):682-8.

243. Luther L, Fischer MW, Johnson-Kwochka AV, Minor KS, Holden R, Lapish CL, et al. Mobile Enhancement of Motivation in Schizophrenia: A Pilot Randomized Controlled Trial of a Personalized Text Message Intervention for Motivation Deficits. Journal of Consulting and Clinical Psychology. 2020;88(10):923-36.

244. Lyu X, Li Z, Chen S, Gu S, Zhou Z, Yang R, et al. Transcranial direct current stimulation improves tardive dyskinesia in long-term hospitalized patients with chronic schizophrenia. Clinical Neurophysiology. 2024;166:20-30.

245. Magliano L, Fiorillo A, Malangone C, De Rosa C, Maj M. A memorial tribute: patient functioning and family burden in a controlled, real-world trial of family psychoeducation for schizophrenia. Psychiatric services. 2006;57(12):1784-91.

246. Mao J, Fan K, Zhang Y, Wen N, Fang X, Ye X, et al. 10 Hz repetitive transcranial magnetic stimulation (rTMS) may improve cognitive function: An exploratory study of schizophrenia patients with auditory hallucinations. Heliyon. 2023;9(9).

247. Marder SR, Meibach RC. RISPERIDONE IN THE TREATMENT OF SCHIZOPHRENIA. American Journal of Psychiatry. 1994;151(6):825-35.

248. Markiewicz R, Markiewicz-Gospodarek A, Dobrowolska B, Loza B. Improving Clinical, Cognitive, and Psychosocial Dysfunctions in Patients with Schizophrenia: A Neurofeedback Randomized Control Trial. Neural Plasticity. 2021;2021.

249. Markiewicz R, Markiewicz-Gospodarek A, Koziol M, Szulecka B, Olajossy M, Plech T. Evaluation of the effectiveness of rehabilitation of people diagnosed with schizophrenia using clinical tools, psychological tests, QEEG, and the brain-derived neurotrophic factor (BDNF). Psychiatria Polska. 2019;53(6):1275-92.

250. Markiewicz R, Markiewicz-Gospodarek A, Trubalski M, Loza B. Neurocognitive, Clinical and Reelin Activity in Rehabilitation Using Neurofeedback Therapy in Patients with Schizophrenia. Journal of Clinical Medicine. 2024;13(14).

251. Maroño Souto Y, Vázquez Campo M, Díaz Llenderrozas F, Rodríguez Álvarez M, Mateos R, García Caballero A. Randomized clinical trial with e-MotionalTraining® 1.0 for social cognition rehabilitation in schizophrenia. Frontiers in psychiatry. 2018;9:40.

252. Martin LAL, Koch SC, Hirjak D, Fuchs T. Overcoming Disembodiment: The Effect of Movement Therapy on Negative Symptoms in Schizophrenia A Multicenter Randomized Controlled Trial. Frontiers in Psychology. 2016;7.

253. Matsuda Y, Morimoto T, Furukawa S, Sato S, Hatsuse N, Iwata K, et al. Feasibility and effectiveness of a cognitive remediation programme with original computerised cognitive training and group intervention for schizophrenia: a multicentre randomised trial. Neuropsychological Rehabilitation. 2018;28(3):387-97.

254. Mazinani R, Nejati S, Khodaei M. Effects of memantine added to risperidone on the symptoms of schizophrenia: A randomized double-blind, placebo-controlled clinical trial. Psychiatry Research. 2017;247:291-5.

255. McGuire P, Robson P, Cubala WJ, Vasile D, Morrison PD, Barron R, et al. Cannabidiol (CBD) as an Adjunctive Therapy in Schizophrenia: A Multicenter Randomized Controlled Trial. American Journal of Psychiatry. 2018;175(3):225-31.

256. McGurk SR, Mueser KT, Xie H, Feldman K, Shaya Y, Klein L, et al. Cognitive remediation for vocational rehabilitation nonresponders. Schizophrenia research. 2016;175(1-3):48-56.

257. Mendella PD, Burton CZ, Tasca GA, Roy P, St Louis L, Twamley EW. Compensatory cognitive training for people with first-episode schizophrenia: Results from a pilot randomized controlled trial. Schizophrenia Research. 2015;162(1-3):108-11.

258. Meskanen K, Ekelund H, Laitinen J, Neuvonen PJ, Haukka J, Panula P, et al. A randomized clinical trial of histamine 2 receptor antagonism in treatment resistant schizophrenia. European Neuropsychopharmacology. 2013;23:S461-S.

259. Meyer-Lindenberg A, Nielsen J, Such P, Lemming OM, Zambori J, Buller R, et al. A double-blind, randomized, placebo-controlled proof of concept study of the efficacy and safety of Lu AF11167 for persistent negative symptoms in people with schizophrenia. European Neuropsychopharmacology. 2022;61:4-14.

260. Mico U, Bruno A, Pandolfo G, Romeo VM, Mallamace D, D'Arrigo C, et al. Duloxetine as adjunctive treatment to clozapine in patients with schizophrenia: a randomized, placebo-controlled trial. International Clinical Psychopharmacology. 2011;26(6):303-10.

261. Miodownik C, Lerner V, Kudkaeva N, Lerner PP, Pashinian A, Bersudsky Y, et al. Curcumin as Add-On to Antipsychotic Treatment in Patients With Chronic Schizophrenia: A Randomized, Double-Blind, Placebo-Controlled Study. Clinical Neuropharmacology. 2019;42(4):117-22.

262. Miyaoka T, Furuya M, Horiguchi J, Wake R, Hashioka S, Toyama M, et al. Efficacy and safety of yokukansan (TJ-54) for treatment-resistant schizophrenia: a randomised placebo-controlled trial. European Neuropsychopharmacology. 2013;23:S476-S.

263. Modabbernia A, Heidari P, Soleimani R, Sobhani A, Roshan ZA, Taslimi S, et al. Melatonin for prevention of metabolic side-effects of olanzapine in patients with first-episode schizophrenia: Randomized double-blind placebo-controlled study. Journal of Psychiatric Research. 2014;53:133-40.

264. Modabbernia A, Rezaei F, Salehi B, Jafarinia M, Ashrafi M, Tabrizi M, et al. Intranasal Oxytocin as an Adjunct to Risperidone in Patients with Schizophrenia An 8-Week, Randomized, Double-Blind, Placebo-Controlled Study. Cns Drugs. 2013;27(1):57-65.

265. Moller HJ, Riedel M, Muller N, Fischer W, Kohnen R. Zotepine versus placebo in the treatment of schizophrenic patients with stable primary negative symptoms: A randomized double-blind multicenter trial. Pharmacopsychiatry. 2004;37(6):270-8.

266. Montag C, Haase L, Seidel D, Bayerl M, Gallinat J, Herrmann U, et al. A Pilot RCT of Psychodynamic Group Art Therapy for Patients in Acute Psychotic Episodes: Feasibility, Impact on Symptoms and Mentalising Capacity. Plos One. 2014;9(11).

267. Morimoto T, Matsuda Y, Matsuoka K, Yasuno F, Ikebuchi E, Kameda H, et al. Computer-assisted cognitive remediation therapy increases hippocampal volume in patients with schizophrenia: a randomized controlled trial. BMC psychiatry. 2018;18:1-8.

268. Morozova M, Burminskiy D, Rupchev G, Lepilkina T, Potanin S, Beniashvili A, et al. 5-HT6 Receptor Antagonist as an Adjunct Treatment Targeting Residual Symptoms in Patients With Schizophrenia: Unexpected Sex-Related Effects (Double-Blind Placebo-Controlled Trial). Journal of Clinical Psychopharmacology. 2017;37(2):169-75.

269. Morozova MA, Beniashvili AG, Lepilkina TA, Rupchev GE. DOUBLE-BLIND PLACEBO-CONTROLLED RANDOMIZED EFFICACY AND SAFETY TRIAL OF ADD-ON TREATMENT OF DIMEBON PLUS RISPERIDONE IN SCHIZOPHRENIC PATIENTS DURING TRANSITION FROM ACUTE PSYCHOTIC EPISODE TO REMISSION. Psychiatria Danubina. 2012;24(2):159-66.

270. Morozova MA, Lepilkina TA, Rupchev GE, Beniashvily AG, Burminskiy DS, Potanin SS, et al. Add-on clinical effects of selective antagonist of 5HT6 receptors AVN-211 (CD-008-0173) in patients with schizophrenia stabilized on antipsychotic treatment: pilot study. Cns Spectrums. 2014;19(4):316-23.

271. Morrison AP, Pyle M, Gumley A, Schwannauer M, Turkington D, MacLennan G, et al. Cognitive-behavioural therapy for clozapine-resistant schizophrenia: the FOCUS RCT. Health Technology Assessment. 2019;23(7):1-+.

272. Morrison AP, Turkington D, Pyle M, Spencer H, Brabban A, Dunn G, et al. Cognitive therapy for people with schizophrenia spectrum disorders not taking antipsychotic drugs: a single-blind randomised controlled trial. Lancet. 2014;383(9926):1395-403.

273. Mortan Sevi O, Tekinsav Sutcu S, Yesilyurt S, Turan Eroglu S, Gunes B. Comparison of the Effectiveness of Two Cognitive-Behavioral Group Therapy Programs for Schizophrenia: Results of a Short-Term Randomized Control Trial. Community Mental Health Journal. 2020;56(2):222-8.

274. Mortazavi M, Farzin D, Zarhghami M, Hosseini S, Mansoori P, Nateghi G. Efficacy of Zinc Sulfate as an Add-on Therapy to Risperidone Versus Risperidone Alone in Patients With Schizophrenia: A Double-Blind Randomized Placebo-Controlled Trial. Iranian Journal of Psychiatry and Behavioral Sciences. 2015;9(3).

275. Motamed M, Karimi H, Sanjari Moghaddam H, Taherzadeh Boroujeni S, Sanatian Z, Hasanzadeh A, et al. Risperidone combination therapy with adalimumab for treatment of chronic schizophrenia: a randomized, double-blind, placebo-controlled clinical trial. International clinical psychopharmacology. 2022.

276. Mozen-Zadeh E, Bayanati S, Ziafat K, Rezaei F, Mesgarpour B, Akhondzadeh S. Vortioxetine as adjunctive therapy to risperidone for treatment of patients with chronic schizophrenia: A randomised, double-blind, placebo-controlled clinical trial. Journal of Psychopharmacology. 2020;34(5):506-13.

277. Mueller DR, Khalesi Z, Benzing V, Castiglione CI, Roder V. Does Integrated Neurocognitive Therapy (INT) reduce severe negative symptoms in schizophrenia outpatients? Schizophrenia Research. 2017;188:92-7.

278. Mueller DR, Khalesi Z, Roder V. Can Cognitive Remediation in Groups Prevent Relapses? Results of a 1-Year Follow-up Randomized Controlled Trial. Journal of Nervous and Mental Disease. 2020;208(5):362-70.

279. Mukai Y, Lupinacci R, Marder S, Snow-Adami L, Voss T, Smith SM, et al. Effects of PDE10A inhibitor MK-8189 in people with an acute episode of schizophrenia: A randomized proof-of-concept clinical trial. Schizophrenia Research. 2024;270:37-43.

280. Mullapudi T, Debnath M, Govindaraj R, Raj P, Banerjee M, Varambally S. Effects of a six-month yoga intervention on the immune-inflammatory pathway in antipsychotic-stabilized schizophrenia patients: A randomized controlled trial. Asian Journal of Psychiatry. 2023;86.

281. Müller H, Kommescher M, Güttgemanns J, Wessels H, Walger P, Lehmkuhl G, et al. Cognitive behavioral therapy in adolescents with early-onset psychosis: a randomized controlled pilot study. European Child & Adolescent Psychiatry. 2020;29(7):1011-22.

282. Müller N, Krause D, Dehning S, Musil R, Schennach-Wolff R, Obermeier M, et al. Celecoxib treatment in an early stage of schizophrenia: results of a randomized, double-blind, placebo-controlled trial of celecoxib augmentation of amisulpride treatment. Schizophrenia research. 2010;121(1-3):118-24.

283. Muscatello MRA, Bruno A, Pandolfo G, Mico U, Scimeca G, Di Nardo F, et al. Effect of aripiprazole augmentation of clozapine in schizophrenia: A double-blind, placebo-controlled study. Schizophrenia Research. 2011;127(1-3):93-9.

284. Muscatello MRA, Pandolfo G, Mico U, Castronuovo EL, Abenavoli E, Scimeca G, et al. Augmentation of Clozapine With Ziprasidone in Refractory Schizophrenia A Double-Blind, Placebo-Controlled Study. Journal of Clinical Psychopharmacology. 2014;34(1):129-33.

285. Nachshoni T, Levin Y, Levy A, Kritz A, Neumann M. A DOUBLE-BLIND TRIAL OF CARBAMAZEPINE IN NEGATIVE SYMPTOM SCHIZOPHRENIA. Biological Psychiatry. 1994;35(1):22-6.

286. Naeem F, Johal R, McKenna C, Rathod S, Ayub M, Lecomte T, et al. Cognitive Behavior Therapy for psychosis based Guided Self-help (CBTp-GSH) delivered by frontline mental health professionals: Results of a feasibility study. Schizophrenia Research. 2016;173(1-2):69-74.

287. Naeem F, Saeed S, Irfan M, Kiran T, Mehmood N, Gul M, et al. Brief culturally adapted CBT for psychosis (CaCBTp): A randomized controlled trial from a low income country. Schizophrenia Research. 2015;164(1-3):143-8.

288. Nasrallah HA, Silva R, Phillips D, Cucchiaro J, Hsu J, Xu J, et al. Lurasidone for the treatment of acutely psychotic patients with schizophrenia: A 6-week, randomized, placebo-controlled study. Journal of Psychiatric Research. 2013;47(5):670-7.

289. Niitsu T, Fujisaki M, Shiina A, Yoshida T, Hasegawa T, Kanahara N, et al. A Randomized, Double-Blind, Placebo-Controlled Trial of Fluvoxamine in Patients With Schizophrenia A Preliminary Study. Journal of Clinical Psychopharmacology. 2012;32(5):593-601.

290. Nikbakhat MR, Arabzadeh S, Zeinoddini A, Khalili Z, Rezaei F, Mohammadinejad P, et al. Duloxetine Add-On to Risperidone for Treatment of Negative Symptoms in Patients with Stable Schizophrenia: Randomized Double-Blind Placebo-Controlled Study. Pharmacopsychiatry. 2016;49(4):162-9.

291. Noorbala AA, Akhondzadeh S, Davari-Ashtiani R, Amini-Nooshabadi H. Piracetam in the treatment of schizophrenia: implications for the glutamate hypothesis of schizophrenia. Journal of Clinical Pharmacy and Therapeutics. 1999;24(5):369-74.

292. Noroozian M, Ghasemi S, Hosseini S-M-R, Modabbernia A, Khodaie-Ardakani M-R, Mirshafiee O, et al. A placebo-controlled study of tropisetron added to risperidone for the treatment of negative symptoms in chronic and stable schizophrenia. Psychopharmacology. 2013;228(4):595-602.

293. O’Reilly K, Donohoe G, O’Sullivan D, Coyle C, Corvin A, O’Flynn P, et al. A randomized controlled trial of cognitive remediation for a national cohort of forensic patients with schizophrenia or schizoaffective disorder. BMC psychiatry. 2019;19(1):27.

294. Oh J, Lee E, Cha EJ, Seo HJ, Choi KH. Community-based multi-site randomized controlled trial of behavioral activation for patients with negative symptoms of schizophrenia. Schizophr Res. 2023;252:118-26.

295. Omranifard V, Rajabi F, Mohammadian-Sichani M, Maracy MR. The effect of add-on memantine on positive, negative and depressive symptoms of schizophrenia: a double-blind, randomized, controlled trial. Actas Espanolas De Psiquiatria. 2017;45(3):108-15.

296. Paikkatt B, Singh AR, Singh PK, Jahan M, Ranjan JK. Efficacy of Yoga therapy for the management of psychopathology of patients having chronic schizophrenia. Indian Journal of Psychiatry. 2015;57(4):355-60.

297. Palm U, Keeser D, Hasan A, Kupka MJ, Blautzik J, Sarubin N, et al. Prefrontal Transcranial Direct Current Stimulation for Treatment of Schizophrenia With Predominant Negative Symptoms: A Double-Blind, Sham-Controlled Proof-of-Concept Study. Schizophrenia Bulletin. 2016;42(5):1253-61.

298. Palma-Sevillano C, Canete-Crespillo J, Farriols-Hernando N, Cebria-Andreu J, Michael M, Alonso-Fernandez I, et al. Randomised controlled trial of cognitive-motivational therapy program for the initial phase of schizophrenia: a 6-month assessment. European Journal of Psychiatry. 2011;25(2):68-80.

299. Pandina GJ, Lindenmayer J-P, Lull J, Lim P, Gopal S, Herben V, et al. A randomized, placebo-controlled study to assess the efficacy and safety of 3 doses of paliperidone palmitate in adults with acutely exacerbated schizophrenia. Journal of Clinical Psychopharmacology. 2010;30(3):235-44.

300. Park S, Lee HK, Kim H. Effects of a Korean version of the metacognitive training program for outpatients with schizophrenia on theory of mind, positive symptoms, and interpersonal relationships. Behavioural and Cognitive Psychotherapy. 2020;48(1):14-24.

301. Pawelczyk T, Grancow-Grabka M, Kotlicka-Antczak M, Trafalska E, Pawelczyk A. A randomized controlled study of the efficacy of six-month supplementation with concentrated fish oil rich in omega-3 polyunsaturated fatty acids in first episode schizophrenia. Journal of Psychiatric Research. 2016;73:34-44.

302. Pawlak A, Kaczmarek B, Wysokinski A, Strzelecki D. Sarcosine May Induce EGF Production or Inhibit the Decline in EGF Concentrations in Patients with Chronic Schizophrenia (Results of the PULSAR Study). Pharmaceuticals. 2023;16(11).

303. Perez-Aguado O, Lacamara S, Ruiz JC, Dasi C, Soldevila-Matias P, Fuentes-Dura I. Effects of group music therapy on symptoms and functional recovery in outpatients with chronic psychoses: A randomized controlled trial. Nordic Journal of Music Therapy. 2024;33(2):122-41.

304. Petersen L, Jeppesen P, Thorup A, Abel M-B, Øhlenschlæger J, Christensen TØ, et al. A randomised multicentre trial of integrated versus standard treatment for patients with a first episode of psychotic illness. Bmj. 2005;331(7517):602.

305. Pierre JM, Peloian JH, Wirshing DA, Wirshing WC, Marder SR. A randomized, double-blind, placebo-controlled trial on modafinil for negative symptoms in schizophrenia. Journal of Clinical Psychiatry. 2007;68(5):705-10.

306. Popova P, Popov TG, Wienbruch C, Carolus AM, Miller GA, Rockstroh BS. Changing facial affect recognition in schizophrenia: effects of training on brain dynamics. NeuroImage: Clinical. 2014;6:156-65.

307. Poulou A, Anagnostopoulos F, Vatakis A, Mellon RC, Mueller DR. The implementation and effectiveness of Integrated Psychological Therapy (IPT) in chronic middle-aged inpatients with schizophrenia. Schizophrenia Research-Cognition. 2025;39.

308. Pourghasem M, Sadighi G, Mirabzadeh A. Comparing the effects of adjunct aspirin and simvastatin on psychopathology among inpatients with schizophrenia. Iranian Rehabilitation Journal. 2022;20(1):33-42.

309. Poyurovsky M, Fuchs C, Pashinian A, Levi A, Faragian S, Maayan R, et al. Attenuating effect of reboxetine on appetite and weight gain in olanzapine-treated schizophrenia patients: a double-blind placebo-controlled study. Psychopharmacology. 2007;192(3):441-8.

310. Poyurovsky M, Fuchs C, Pashinian A, Levi A, Weizman R, Weizman A. Reducing antipsychotic-induced weight gain in schizophrenia: a double-blind placebo-controlled study of reboxetine–betahistine combination. Psychopharmacology. 2013;226(3):615-22.

311. Prikryl R, Kasparek T, Skotakova S, Ustohal L, Kucerova H, Ceskova E. Treatment of negative symptoms of schizophrenia using repetitive transcranial magnetic stimulation in a double-blind, randomized controlled study. Schizophrenia Research. 2007;95(1-3):151-7.

312. Prikryl R, Mikl M, Kucerova HP, Ustohal L, Kasparek T, Marecek R, et al. Does repetitive transcranial magnetic stimulation have a positive effect on working memory and neuronal activation in treatment of negative symptoms of schizophrenia? Neuroendocrinology Letters. 2012;33(1):90-7.

313. Prikryl R, Ustohal L, Kucerova HP, Kasparek T, Jarkovsky J, Hublova V, et al. Repetitive transcranial magnetic stimulation reduces cigarette consumption in schizophrenia patients. Progress in Neuro-Psychopharmacology & Biological Psychiatry. 2014;49:30-5.

314. Prikryl R, Ustohal L, Kucerova HP, Kasparek T, Venclikova S, Vrzalova M, et al. A detailed analysis of the effect of repetitive transcranial magnetic stimulation on negative symptoms of schizophrenia: A double-blind trial. Schizophrenia Research. 2013;149(1-3):167-73.

315. Pu Z, Wen H, Jiang H, Hou Q, Yan H. Berberine improves negative symptoms and cognitive function in patients with chronic schizophrenia via anti-inflammatory effect: a randomized clinical trial. Chinese Medicine. 2023;18(1).

316. Quan WX, Zhu XL, Qiao H, Zhang WF, Tan SP, Zhou DF, et al. The effects of high-frequency repetitive transcranial magnetic stimulation (rTMS) on negative symptoms of schizophrenia and the follow-up study. Neuroscience Letters. 2015;584:197-201.

317. Rabany L, Deutsch L, Levkovitz Y. Double-blind, randomized sham controlled study of deep-TMS add-on treatment for negative symptoms and cognitive deficits in schizophrenia. Journal of Psychopharmacology. 2014;28(7):686-90.

318. Rakitzi S, Georgila P, Efthimiou K, Mueller DR. Efficacy and feasibility of the Integrated Psychological Therapy for outpatients with schizophrenia in Greece: Final results of a RCT. Psychiatry Research. 2016;242:137-43.

319. Rao NP, Ramachandran P, Jacob A, Joseph A, Thonse U, Nagendra B, et al. Add on yoga treatment for negative symptoms of schizophrenia: A multi-centric, randomized controlled trial. Schizophrenia Research. 2021;231:90-7.

320. Rao P, Li S, Zhong T. The Effectiveness of the 4P Nursing Model Combined with Amisulpride and Clozapine in the Management of Psychiatric Patients. Alternative therapies in health and medicine. 2024.

321. Rapado-Castro M, Berk M, Venugopal K, Bush AI, Dodd S, Dean OM. Towards stage specific treatments: Effects of duration of illness on therapeutic response to adjunctive treatment with N-acetyl cysteine in schizophrenia. Progress in Neuro-Psychopharmacology & Biological Psychiatry. 2015;57:69-75.

322. Rapaport MH, Delrahim KK, Bresee CJ, Maddux RE, Ahmadpour O, Dolnak D. Celecoxib augmentation of continuously ill patients with schizophrenia. Biological psychiatry. 2005;57(12):1594-6.

323. Ray P, Sinha VK, Tikka SK. Adjuvant low-frequency rTMS in treating auditory hallucinations in recent-onset schizophrenia: a randomized controlled study investigating the effect of high-frequency priming stimulation. Annals of General Psychiatry. 2015;14.

324. Rector NA, Seeman MV, Segal ZV. Cognitive therapy for schizophrenia: a preliminary randomized controlled trial. Schizophrenia Research. 2003;63(1-2):1-11.

325. Rezaei F, Mesgarpour B, Jeddian A, Zeionoddini A, Mohammadinejad P, Salardini E, et al. Cilostazol adjunctive therapy in treatment of negative symptoms in chronic schizophrenia: Randomized, double-blind, placebo-controlled study. Human Psychopharmacology-Clinical and Experimental. 2017;32(4).

326. Rezaei F, Mohammad-karimi M, Seddighi S, Modabbernia A, Ashrafi M, Salehi B, et al. Memantine Add-On to Risperidone for Treatment of Negative Symptoms in Patients With Stable Schizophrenia Randomized, Double-Blind, Placebo-Controlled Study. Journal of Clinical Psychopharmacology. 2013;33(3):336-42.

327. Reznik I, Benatov R, Sirota P. Long-term efficacy and safety of quetiapine in treatment-refractory schizophrenia: A case report. International Journal of Psychiatry in Clinical Practice. 2000;4(1):77-80.

328. Richardson P, Jones K, Evans C, Stevens P, Rowe A. Exploratory RCT of art therapy as an adjunctive treatment in schizophrenia. Journal of Mental Health. 2007;16(4):483-91.

329. Ritsner M, Gibel A, Ram E, Maayan R, Weizman A. Alterations in DHEA metabolism in schizophrenia: Two-month case-control study. European Neuropsychopharmacology. 2006;16(2):137-46.

330. Ritsner M, Kardashev A, Ratner Y. Adjunctive pregnenolone and L-theanine as a novel therapeutic strategy in schizophrenia. European Neuropsychopharmacology. 2014;24:S529-S.

331. Ritsner MS. THE CLINICAL AND THERAPEUTIC POTENTIALS OF DEHYDROEPIANDROSTERONE AND PREGNENOLONE IN SCHIZOPHRENIA. Neuroscience. 2011;191:91-100.

332. Ritsner MS, Bawakny H, Kreinin A. Pregnenolone treatment reduces severity of negative symptoms in recent-onset schizophrenia: An 8-week, double-blind, randomized add-on two-center trial. Psychiatry and Clinical Neurosciences. 2014;68(6):432-40.

333. Ritsner MS, Gibel A, Shleifer T, Boguslavsky I, Zayed A, Maayan R, et al. Pregnenolone and Dehydroepiandrosterone as an Adjunctive Treatment in Schizophrenia and Schizoaffective Disorder: An 8-Week, Double-Blind, Randomized, Controlled, 2-Center, Parallel-Group Trial. Journal of Clinical Psychiatry. 2010;71(10):1351-62.

334. Roberts DL, Combs DR, Willoughby M, Mintz J, Gibson C, Rupp B, et al. A randomized, controlled trial of Social Cognition and Interaction Training (SCIT) for outpatients with schizophrenia spectrum disorders. British Journal of Clinical Psychology. 2014;53(3):281-98.

335. Roffman JL, Lamberti JS, Achtyes E, Macklin EA, Galendez GC, Raeke LH, et al. Randomized Multicenter Investigation of Folate Plus Vitamin B-12 Supplementation in Schizophrenia. Jama Psychiatry. 2013;70(5):481-9.

336. Roncone R, Mazza M, Frangou I, De Risio A, Ussorio D, Tozzini C, et al. Rehabilitation of theory of mind deficit in schizophrenia: a pilot study of metacognitive strategies in group treatment. Neuropsychological rehabilitation. 2004;14(4):421-35.

337. Rus-Calafell M, Gutierrez-Maldonado J, Ortega-Bravo M, Ribas-Sabate J, Caqueo-Urizar A. A brief cognitive-behavioural social skills training for stabilised outpatients with schizophrenia: A preliminary study. Schizophrenia Research. 2013;143(2-3):327-36.

338. Rus-Calafell M, Gutiérrez-Maldonado J, Ortega-Bravo M, Ribas-Sabaté J, Caqueo-Urízar A. A brief cognitive–behavioural social skills training for stabilised outpatients with schizophrenia: A preliminary study. Schizophrenia research. 2013;143(2-3):327-36.

339. Sachs G, Winklbaur B, Jagsch R, Lasser I, Kryspin-Exner I, Frommann N, et al. Training of affect recognition (TAR) in schizophrenia-Impact on functional outcome. Schizophrenia Research. 2012;138(2-3):262-7.

340. Salimi S, Fotouhi A, Ghoreishi A, Derakhshan M-K, Khodaie-Ardakani M-R, Mohammadi M-R, et al. A placebo controlled study of the propentofylline added to risperidone in chronic schizophrenia. Progress in Neuro-Psychopharmacology & Biological Psychiatry. 2008;32(3):726-32.

341. Salokangas RKR, Saarijarvi S, Taiminen T, Kallioniemi H, Lehto H, Niemi H, et al. Citalopram as an adjuvant in chronic schizophrenia: A double-blind placebo-controlled study. Acta Psychiatrica Scandinavica. 1996;94(3):175-80.

342. Samadi R, Soluti S, Daneshmand R, Assari S, Manteghi AA. Efficacy of Risperidone Augmentation with Ondansetron in the Treatment of Negative and Depressive Symptoms in Schizophrenia: A Randomized Clinical Trial. Iran J Med Sci. 2017;42(1):14-23.

343. Samaei A, Moradi K, Bagheri S, Ashraf-Ganjouei A, Alikhani R, Mousavi SB, et al. Resveratrol Adjunct Therapy for Negative Symptoms in Patients With Stable Schizophrenia: A Double-Blind, Randomized Placebo-Controlled Trial. International Journal of Neuropsychopharmacology. 2020;23(12):775-82.

344. Saporta-Wiesel L, Feldman R, Levi L, Davidson M, Burshtein S, Gur R, et al. Intranasal Oxytocin Combined With Social Skills Training for Schizophrenia: An Add-on Randomized Controlled Trial. Schizophrenia bulletin open. 2024;5(1):sgae022-sgae.

345. Sayed SM, Ahmed GK, Abdullah SO, Khalifa SM. Effect of group psychotherapy on self-care skills in schizophrenia. Egyptian Journal of Neurology Psychiatry and Neurosurgery. 2024;60(1).

346. Schoemaker JH, Jansen WT, Schipper J, Szegedi A. The Selective Glycine Uptake Inhibitor Org 25935 as an Adjunctive Treatment to Atypical Antipsychotics in Predominant Persistent Negative Symptoms of Schizophrenia Results From the GIANT Trial. Journal of Clinical Psychopharmacology. 2014;34(2):190-8.

347. Şenormancı G, Korkmaz N, Şenormancı Ö, Uğur S, Topsaç M, Gültekin O. Effects of exercise on resilience, insight and functionality in patients with chronic schizophrenia in a psychiatric nursing home setting: a randomized controlled trial. Issues in Mental Health Nursing. 2021;42(7):690-8.

348. Shafti SS, Akbari S. Intractability of Deficit Syndrome of Schizophrenia Against Adjunctive Modafinil. Journal of Clinical Psychopharmacology. 2016;36(1):45-9.

349. Shafti SS, Jafarabad MS, Azizi R. Amelioration of deficit syndrome of schizophrenia by norepinephrine reuptake inhibitor. Therapeutic Advances in Psychopharmacology. 2015;5(5):263-70.

350. Shafti SS, Khoei AA. Effectiveness of rivastigmine on positive, negative, and cognitive symptoms of schizophrenia: a double-blind clinical trial. Therapeutic Advances in Psychopharmacology. 2016;6(5):308-16.

351. Shamabadi A, Fattollahzadeh-Noor S, Fallahpour B, A. Basti F, Ardakani M-RK, Akhondzadeh S. L-Theanine adjunct to risperidone in the treatment of chronic schizophrenia inpatients: a randomized, double-blind, placebo-controlled clinical trial. Psychopharmacology. 2023;240(12):2631-40.

352. Sheikhmoonesi F, Zarghami M, Saravi SFB, Khalilian A, Ala S. A triple-blinded, randomized, placebo-controlled trial to examine the efficacy of buspirone added to typical antipsychotic drugs in patients with chronic schizophrenia. Journal of Research in Medical Sciences. 2015;20(2):140-5.

353. Sheitman BB, Knable MB, Jarskog LF, Chakos M, Boyce LH, Early J, et al. Secretin for refractory schizophrenia. Schizophrenia Research. 2004;66(2-3):177-81.

354. Shen H, Lian A, Wu Y, Zhou J, Liu Y, Zhu L, et al. Shen-based Qigong Exercise improves cognitive impairment in stable schizophrenia patients in rehabilitation wards: a randomized controlled study. Bmc Psychiatry. 2024;24(1).

355. Shen H, Zhang L, Li Y, Zheng D, Du L, Xu F, et al. Mindfulness-based intervention improves residual negative symptoms and cognitive impairment in schizophrenia: a randomized controlled follow-up study. Psychological Medicine. 2023;53(4):1390-9.

356. Shen JHQ, Zhao Y, Rosenzweig-Lipson S, Popp D, Williams JBW, Giller E, et al. A 6-week randomized, double-blind, placebo-controlled, comparator referenced trial of vabicaserin in acute schizophrenia. Journal of Psychiatric Research. 2014;53:14-22.

357. Shi S, Cui S, Yao Y, Ge M, Yang M, Sheng X, et al. Smartphone video games improve cognitive function in patients with chronic schizophrenia: a randomized controlled trial. European Archives of Psychiatry and Clinical Neuroscience. 2024;274(4):929-39.

358. Shibre T, Alem A, Abdulahi A, Araya M, Beyero T, Medhin G, et al. Trimethoprim as Adjuvant Treatment in Schizophrenia: A Double-Blind, Randomized, Placebo-Controlled Clinical Trial. Schizophrenia Bulletin. 2010;36(4):846-51.

359. Shiina A, Shirayama Y, Niitsu T, Hashimoto T, Yoshida T, Hasegawa T, et al. A randomised, double-blind, placebo-controlled trial of tropisetron in patients with schizophrenia. Annals of General Psychiatry. 2010;9(1):27.

360. Shiloh R, Zemishlany Z, Aizenberg D, Radwan M, Schwartz B, Dorfman-Etrog P, et al. Sulpiride augmentation in people with schizophrenia partially responsive to clozapine - A double-blind, placebo-controlled study. British Journal of Psychiatry. 1997;171:569-73.

361. Silver H, Nassar A. FLUVOXAMINE IMPROVES NEGATIVE SYMPTOMS IN TREATED CHRONIC-SCHIZOPHRENIA - AN ADD-ON DOUBLE-BLIND, PLACEBO-CONTROLLED STUDY. Biological Psychiatry. 1992;31(7):698-704.

362. Singh J, Robb A, Vijapurkar U, Nuamah I, Hough D. A Randomized, Double-Blind Study of Paliperidone Extended-Release in Treatment of Acute Schizophrenia in Adolescents. Biological Psychiatry. 2011;70(12):1179-87.

363. Singh S, Kumar N, Verma R, Nehra A. The safety and efficacy of adjunctive 20-Hz repetitive transcranial magnetic stimulation for treatment of negative symptoms in patients with schizophrenia: A double-blinded, randomized, sham-controlled study. Indian Journal of Psychiatry. 2020;62(1):21-9.

364. Sinichi F, Hosseini FF, Fayyazi-Bordbar M, Sinichi M, Jamali J, Mohammadpour A. Pentoxifylline as adjunctive therapy in cognitive deficits and symptoms of schizophrenia: A randomized double-blind placebo-controlled clinical trial. Journal of Psychopharmacology. 2023;37(10):1003-10.

365. Small JG, Hirsch SR, Arvanitis LA, Miller BG, Link CGG, Murphy AL, et al. Quetiapine in patients with schizophrenia - A high- and low-dose double-blind comparison with placebo. Archives of General Psychiatry. 1997;54(6):549-57.

366. Smith RC, Amiaz R, Si T-M, Maayan L, Jin H, Boules S, et al. Varenicline Effects on Smoking, Cognition, and Psychiatric Symptoms in Schizophrenia: A Double-Blind Randomized Trial. Plos One. 2016;11(1).

367. Sommer IE, Gangadin SS, de Witte LD, Koops S, van Baal C, Bahn S, et al. Simvastatin Augmentation for Patients With Early-Phase Schizophrenia-Spectrum Disorders: A Double-Blind, Randomized Placebo-Controlled Trial. Schizophrenia Bulletin. 2021;47(4):1108-15.

368. Spina E, Dedomenico P, Ruello C, Longobardo N, Gitto C, Ancione M, et al. ADJUNCTIVE FLUOXETINE IN THE TREATMENT OF NEGATIVE SYMPTOMS IN CHRONIC-SCHIZOPHRENIC PATIENTS. International Clinical Psychopharmacology. 1994;9(4):281-5.

369. Steuwe C, Carvalho Fernando S, Runte I, Bender S, Heiler W, Klein F, et al. Cognitive Behavioral Psychotherapy as an Add-on in Comprehensive Outpatient Care of Non-affective Psychoses: A Multicenter Randomized-Controlled Effectiveness Trial in a Naturalistic Setting. Schizophrenia bulletin. 2024.

370. Stone JM, Morrison PD, Koychev I, Gao F, Reilly TJ, Kolanko M, et al. The effect of sodium nitroprusside on psychotic symptoms and spatial working memory in patients with schizophrenia: a randomized, double-blind, placebo-controlled trial. Psychological Medicine. 2016;46(16):3443-50.

371. Strous RD, Stryjer R, Maayan R, Gal G, Viglin D, Katz E, et al. Analysis of clinical symptomatology, extrapyramidal symptoms and neurocognitive dysfunction following dehydroepiandrosterone (DHEA) administration in olanzapine treated schizophrenia patients: a randomized, double-blind placebo controlled trial. Psychoneuroendocrinology. 2007;32(2):96-105.

372. Strzelecki D, Urban‐Kowalczyk M, Wysokiński A. Serum levels of interleukin 6 in schizophrenic patients during treatment augmentation with sarcosine (results of the PULSAR study). Human Psychopharmacology: Clinical and Experimental. 2018;33(2):e2652.

373. Su X, Wang X, Pan X, Zhang X, Lu X, Zhao L, et al. Effect of Repetitive Transcranial Magnetic Stimulation in Inducing Weight Loss in Patients with Chronic Schizophrenia: A Randomized, Double-Blind Controlled 4-Week Study. Curr Neuropharmacol. 2023;21(2):417-23.

374. Su X, Zhao L, Shang Y, Chen Y, Liu X, Wang X, et al. Repetitive transcranial magnetic stimulation for psychiatric symptoms in long-term hospitalized veterans with schizophrenia: A randomized double-blind controlled trial. Front Psychiatry. 2022;13:873057.

375. Tajik-Esmaeeli S, Moazen-Zadeh E, Abbasi N, Shariat SV, Rezaei F, Salehi B, et al. Simvastatin adjunct therapy for negative symptoms of schizophrenia: a randomized double-blind placebo-controlled trial. International Clinical Psychopharmacology. 2017;32(2):87-94.

376. Takahashi N, Takahashi M, Saito T, Iizumi M, Saito Y, Shimizu H, et al. Randomized, placebo-controlled, double-blind study assessing the efficacy and safety of paliperidone palmitate in Asian patients with schizophrenia. Neuropsychiatric Disease and Treatment. 2013;9:1889-97.

377. Talwar N, Crawford MJ, Maratos A, Nur U, McDermott O, Procter S. Music therapy for in-patients with schizophrenia: exploratory randomised controlled trial. The British journal of psychiatry. 2006;189(5):405-9.

378. Tang W, Yao X, Zheng Z. Rehabilitative effect of music therapy for residual schizophrenia: A one-month randomised controlled trial in Shanghai. The British journal of psychiatry. 1994;165(S24):38-44.

379. Tas C, Danaci AE, Cubukcuoglu Z, Bruene M. Impact of family involvement on social cognition training in clinically stable outpatients with schizophrenia - A randomized pilot study. Psychiatry Research. 2012;195(1-2):32-8.

380. Tharoor H, Maran S, Chandan AK, Pari M, Rao S, Durairaj J. Cognitive and negative symptoms in schizophrenia with L-Carnosine adjuvant therapy - A randomized double-blind placebo-controlled study. Pharmacology Research & Perspectives. 2023;11(2).

381. Thomas ML, Bismark AW, Joshi YB, Tarasenko M, Treichler EB, Hochberger WC, et al. Targeted cognitive training improves auditory and verbal outcomes among treatment refractory schizophrenia patients mandated to residential care. Schizophrenia Research. 2018;202:378-84.

382. Tiihonen J, Hallikainen T, Ryynanen OP, Repo-Tiihonen E, Kotilainen I, Eronen M, et al. Lamotrigine in treatment-resistant schizophrenia: A randomized placebo-controlled crossover trial. Biological Psychiatry. 2003;54(11):1241-8.

383. Tiihonen J, Halonen P, Wahlbeck K, Repo-Tiihonen E, Hyvarinen S, Eronen M, et al. Topiramate add-on in treatment-resistant schizophrenia: A randomized, double-blind, placebo-controlled, crossover trial. Journal of Clinical Psychiatry. 2005;66(8):1012-5.

384. Tollefson GD, Dellva MA, Mattler CA, Kane JM, Wirshing DA, Kinon BJ, et al. Controlled, double-blind investigation of the clozapine discontinuation symptoms with conversion to either olanzapine or placebo. Journal of Clinical Psychopharmacology. 1999;19(5):435-43.

385. Tollefson GD, Sanger TM. Negative symptoms: A path analytic approach to a double-blind, placebo- and haloperidol-controlled clinical trial with olanzapine. American Journal of Psychiatry. 1997;154(4):466-74.

386. Truffinet P, Tamminga CA, Fabre LF, Meltzer HY, Riviere ME, Papillon-Downey C. Placebo-controlled study of the D-4/5-HT2A antagonist fananserin in the treatment of schizophrenia. American Journal of Psychiatry. 1999;156(3):419-25.

387. Tsai GC, Lane HY, Yang PC, Chong MY, Lange N. Glycine transporter I inhibitor, N-methylglycine (Sarcosine), added to antipsychotics for the treatment of schizophrenia. Biological Psychiatry. 2004;55(5):452-6.

388. Tsai GC, Yang PC, Chung LC, Lange N, Coyle JT. D-serine added to antipsychotics for the treatment of schizophrenia. Biological Psychiatry. 1998;44(11):1081-9.

389. Tsai GCE, Yang PC, Chung LC, Tsai IC, Tsai CW, Coyle JT. D-Serine added to clozapine for the treatment of schizophrenia. American Journal of Psychiatry. 1999;156(11):1822-5.

390. Tsai GE, Yang PC, Chang YC, Chong MY. D-alanine added to antipsychotics for the treatment of schizophrenia. Biological Psychiatry. 2006;59(3):230-4.

391. Tuppurainen H, Maatta S, Kononen M, Julkunen P, Kautiainen H, Hyvarinen S, et al. Navigated and individual α-peak-frequency-guided transcranial magnetic stimulation in male patients with treatment-refractory schizophrenia. Journal of Psychiatry & Neuroscience. 2024;49(2):E87-E95.

392. Turner A, Baker A, Dean OM, Walker AJ, Dodd S, Cotton SM, et al. Adjunctive Garcinia mangostana Linn. (Mangosteen) Pericarp for Schizophrenia: A 24-Week Double-blind, Randomized, Placebo Controlled Efficacy Trial: Pericarpe d'appoint Garcinia mangostana Linn (mangoustan) pour la schizophrenie : un essai d'efficacite de 24 semaines, a double insu, randomise et controle par placebo. Canadian Journal of Psychiatry-Revue Canadienne De Psychiatrie. 2021;66(4):354-66.

393. Twamley EW, Vella L, Burton CZ, Heaton RK, Jeste DV. Compensatory cognitive training for psychosis: effects in a randomized controlled trial. J Clin Psychiatry. 2012;73(9):1212-9.

394. Tyagi P, Dhyani M, Khattri S, Tejan V, Tikka SK, Garg S. "Efficacy of intensive bilateral Temporo-Parietal Continuous theta-burst Stimulation for Auditory VErbal hallucinations (TPC-SAVE) in schizophrenia: A randomized sham-controlled trial"(☆). Asian J Psychiatr. 2022;74:103176.

395. Umbricht D, Keefe RSE, Murray S, Lowe DA, Porter R, Garibaldi G, et al. A Randomized, Placebo-Controlled Study Investigating the Nicotinic alpha 7 Agonist, RG3487, for Cognitive Deficits in Schizophrenia. Neuropsychopharmacology. 2014;39(7):1568-77.

396. Usall J, Huerta-Ramos E, Iniesta R, Cobo J, Araya S, Roca M, et al. Raloxifene as an Adjunctive Treatment for Postmenopausal Women With Schizophrenia: A Double-Blind, Randomized, Placebo-Controlled Trial. Journal of Clinical Psychiatry. 2011;72(11):1552-7.

397. Usall J, Huerta-Ramos E, Labad J, Cobo J, Nunez C, Creus M, et al. Raloxifene as an Adjunctive Treatment for Postmenopausal Women With Schizophrenia: A 24-Week Double-Blind, Randomized, Parallel, Placebo-Controlled Trial. Schizophrenia Bulletin. 2016;42(2):309-17.

398. Usall J, Lopez-Carrilero R, Iniesta R, Roca M, Caballero M, Rodriguez-Jimenez R, et al. Double-Blind, Placebo-Controlled Study of the Efficacy of Reboxetine and Citalopram as Adjuncts to Atypical Antipsychotics for Negative Symptoms of Schizophrenia. Journal of Clinical Psychiatry. 2014;75(6):608-15.

399. Vahia IV, Lanouette NM, Golshan S, Fellows I, Mohamed S, Kasckow JW, et al. Adding antidepressants to antipsychotics for treatment of subsyndromal depressive symptoms in schizophrenia: Impact on positive and negative symptoms. Indian Journal of Psychiatry. 2013;55(2):144-8.

400. Valencia M, Luisa Rascon M, Juarez F, Escamilla R, Saracco R, Liberman RP. Application in Mexico of Psychosocial Rehabilitation with Schizophrenia Patients. Psychiatry-Interpersonal and Biological Processes. 2010;73(3):248-63.

401. Valencia M, Rascon ML, Juarez F, Murow E. A psychosocial skills training approach in Mexican out-patients with schizophrenia. Psychological Medicine. 2007;37(10):1393-402.

402. Valiengo LdCL, Goerigk S, Gordon PC, Padberg F, Serpa MH, Koebe S, et al. Efficacy and Safety of Transcranial Direct Current Stimulation for Treating Negative Symptoms in Schizophrenia A Randomized Clinical Trial. Jama Psychiatry. 2020;77(2):121-9.

403. Van Berckel B, Evenblij C, Van Loon B, Maas M, Van der Geld M, Wynne H, et al. D-cycloserine increases positive symptoms in chronic schizophrenic patients when administered in addition to antipsychotics: a double-blind, parallel, placebo-controlled study. Neuropsychopharmacology. 1999;21(2):203-10.

404. Varambally S, Holla B, Venkatasubramanian G, Mullapudi T, Raj P, Shivakumar V, et al. Clinical effects of a yoga-based intervention for patients with schizophrenia - A six-month randomized controlled trial. Schizophrenia Research. 2024;269:144-51.

405. Vaskinn A, Lovgren A, Egeland MK, Feyer FK, Ostefjells T, Andreassen OA, et al. A randomized controlled trial of training of affect recognition (TAR) in schizophrenia shows lasting effects for theory of mind. European Archives of Psychiatry and Clinical Neuroscience. 2019;269(5):611-20.

406. Vass E, Simon V, Csukly G, Fekete Z, Kis B, Simon L. Virtual reality-based theory of mind intervention in schizophrenia: Preliminary efficacy results. Compr Psychiatry. 2022;119:152350.

407. Vincenzi B, Stock S, Borba CPC, Cleary SM, Oppenheim CE, Petruzzi LJ, et al. A randomized placebo-controlled pilot study of pravastatin as an adjunctive therapy in schizophrenia patients: Effect on inflammation, psychopathology, cognition and lipid metabolism. Schizophrenia Research. 2014;159(2-3):395-403.

408. Vita A, De Peri L, Barlati S, Cacciani P, Cisima M, Deste G, et al. Psychopathologic, neuropsychological and functional outcome measures during cognitive rehabilitation in schizophrenia: a prospective controlled study in a real-world setting. European Psychiatry. 2011;26(5):276-83.

409. Voineskos AN, Blumberger DM, Schifani C, Hawco C, Dickie EW, Rajji TK, et al. Effects of repetitive transcranial magnetic stimulation on working memory performance and brain structure in people with schizophrenia spectrum disorders: a double-blind, randomized, sham-controlled trial. Biological Psychiatry: Cognitive Neuroscience and Neuroimaging. 2021;6(4):449-58.

410. Vreeland B, Minsky S, Yanos PT, Menza M, Gara M, Kim E, et al. Efficacy of the team solutions program for educating patients about illness management and treatment. Psychiatr Serv. 2006;57(6):822-8.

411. Wang C-H, Li Y, Yang J, Su L-Y, Geng Y-G, Li H, et al. A randomized controlled trial of olanzapine improving memory deficits in Han Chinese patients with first-episode schizophrenia. Schizophrenia Research. 2013;144(1-3):129-35.

412. Wang L, Chen X, Wu Y, He K, Xu F, Xiao G, et al. Intermittent theta burst stimulation (iTBS) adjustment effects of schizophrenia: Results from an exploratory outcome of a randomized double-blind controlled study. Schizophrenia Research. 2020;216:550-3.

413. Wang L-Q, Chien WT, Yip LK, Karatzias T. A randomized controlled trial of a mindfulness-based intervention program for people with schizophrenia: 6-month follow-up. Neuropsychiatric disease and treatment. 2016:3097-110.

414. Wang N, Zheng N, Dong M, He J. Paroxetine combined with olanzapine in the treatment of schizophrenia. Pakistan Journal of Medical Sciences. 2020;36(3):516-20.

415. Wang X, Zhao J, Hu Y, Jiao Z, Lu Y, Ding M, et al. Sodium nitroprusside treatment for psychotic symptoms and cognitive deficits of schizophrenia: A randomized, double-blind, placebo-controlled trial. Psychiatry Research. 2018;269:271-7.

416. Wang Y, Huang X, Fan H, An H, Ma T, Zhang Q, et al. High-Dose Betahistine Improves Cognitive Function in Patients With Schizophrenia: A Randomized Double-Blind Placebo-Controlled Trial. Frontiers in Psychiatry. 2021;12.

417. Wang Y, Wang G, Gong M, Yang Y, Ling Y, Fang X, et al. Systemic inflammatory biomarkers in Schizophrenia are changed by ECT administration and related to the treatment efficacy. Bmc Psychiatry. 2024;24(1).

418. Wass C, Klamer D, Katsarogiannis E, Palsson E, Svensson L, Fejgin K, et al. L-lysine as adjunctive treatment in patients with schizophrenia: a single-blinded, randomized, cross-over pilot study. Bmc Medicine. 2011;9.

419. Weickert TW, Jacomb I, Lenroot R, Lappin J, Weinberg D, Brooks WS, et al. Adjunctive canakinumab reduces peripheral inflammation markers and improves positive symptoms in people with schizophrenia and inflammation: A randomized control trial. Brain Behavior and Immunity. 2024;115:191-200.

420. Weiner E, Conley RR, Ball MP, Feldman S, Gold JM, Kelly DL, et al. Adjunctive Risperidone for Partially Responsive People with Schizophrenia Treated with Clozapine. Neuropsychopharmacology. 2010;35(11):2274-83.

421. Weiser M. The Effect of Estrogen in Treatment Resistant Schizophrenia: Results From a Randomized Controlled Trial. Neuropsychopharmacology. 2017;42:S76-S.

422. Weiser M, Heresco-Levy U, Davidson M, Javitt DC, Werbeloff N, Gershon AA, et al. A multicenter, add-on randomized controlled trial of low-dose d-serine for negative and cognitive symptoms of schizophrenia. The Journal of clinical psychiatry. 2012;73(6):e728-34.

423. Wobrock T, Guse B, Cordes J, Woelwer W, Winterer G, Gaebel W, et al. Left Prefrontal High-Frequency Repetitive Transcranial Magnetic Stimulation for the Treatment of Schizophrenia with Predominant Negative Symptoms: A Sham-Controlled, Randomized Multicenter Trial. Biological Psychiatry. 2015;77(11):979-88.

424. Wölwer W, Frommann N, Halfmann S, Piaszek A, Streit M, Gaebel W. Remediation of impairments in facial affect recognition in schizophrenia: efficacy and specificity of a new training program. Schizophrenia research. 2005;80(2-3):295-303.

425. Wykes T, Reeder C, Landau S, Everitt B, Knapp M, Patel A, et al. Cognitive remediation therapy in schizophrenia: randomised controlled trial. The British journal of psychiatry. 2007;190(5):421-7.

426. Xiao S, Xue H, Li G, Yuan C, Li X, Chen C, et al. Therapeutic effects of cerebrolysin added to risperidone in patients with schizophrenia dominated by negative symptoms. Australian and New Zealand Journal of Psychiatry. 2012;46(2):153-60.

427. Xiao S-F, Xue H-B, Li X, Chen C, Li G-J, Yuan C-M, et al. A double-blind, placebo-controlled study of traditional Chinese medicine sarsasapogenin added to risperidone in patients with negative symptoms dominated schizophrenia. Neuroscience Bulletin. 2011;27(4):258-68.

428. Xiu MH, Guan HY, Zhao JM, Wang KQ, Pan YF, Su XR, et al. Cognitive Enhancing Effect of High-Frequency Neuronavigated rTMS in Chronic Schizophrenia Patients With Predominant Negative Symptoms: A Double-Blind Controlled 32-Week Follow-up Study. Schizophrenia Bulletin. 2020;46(5):1219-30.

429. Xue F, Wang X-F, Kong F-N, Yin T-L, Wang Y-H, Shi L-D, et al. Effects of bilateral repetitive transcranial magnetic stimulation on prospective memory in patients with schizophrenia: A double-blind randomized controlled clinical trial. Neuropsychopharmacology Reports. 2024;44(1):97-108.

430. Yamanushi A, Shimada T, Koizumi A, Kobayashi M. Effect of Computer-Assisted Cognitive Remediation Therapy on Cognition among Patients with Schizophrenia: A Pilot Randomized Controlled Trial. Biomedicines. 2024;12(7).

431. Yang Y, Jin X, Xue Y, Li X, Chen Y, Kang N, et al. Right superior frontal gyrus: A potential neuroimaging biomarker for predicting short-term efficacy in schizophrenia. Neuroimage-Clinical. 2024;42.

432. Yeh T-C, Lin Y-Y, Tzeng N-S, Kao Y-C, Chung Y-A, Chang C-C, et al. Effects of online high-definition transcranial direct current stimulation over left dorsolateral prefrontal cortex on predominant negative symptoms and EEG functional connectivity in patients with schizophrenia: a randomized, double-blind, controlled trial. Psychiatry and Clinical Neurosciences. 2024.

433. Yeh TC, Huang CC, Chung YA, Park SY, Im JJ, Lin YY, et al. Online Left-Hemispheric In-Phase Frontoparietal Theta tACS Modulates Theta-Band EEG Source-Based Large-Scale Functional Network Connectivity in Patients with Schizophrenia: A Randomized, Double-Blind, Sham-Controlled Clinical Trial. Biomedicines. 2023;11(2).

434. Zarghami M, Dodangi N, Azari P, Khalilian A. Antipsychotic Effects of Celecoxib Add-On Haloperidol in Schizophrenia: A Randomized Double-Blind Placebo-Controlled Clinical Trial. Iranian Journal of Psychiatry and Behavioral Sciences. 2024;18(1).

435. Zeinoddini A, Ahadi M, Farokhnia M, Rezaei F, Tabrizi M, Akhondzadeh S. L-lysine as an adjunct to risperidone in patients with chronic schizophrenia: A double-blind, placebo-controlled, randomized trial. Journal of Psychiatric Research. 2014;59:125-31.

436. Zhai Z, Ren L, Song Z, Xiang Q, Zhuo K, Zhang S, et al. The efficacy of low-intensity transcranial ultrasound stimulation on negative symptoms in schizophrenia: A double-blind, randomized sham-controlled study. Brain Stimulation. 2023;16(3):790-2.

437. Zhang H, Li C, Zhao L, Zhan G. Single-blind, randomized controlled trial of effectiveness of Naikan therapy as an adjunctive treatment for schizophrenia over a one-year follow-up period. Shanghai archives of psychiatry. 2015;27(4):220-7.

438. Zhang L, Zheng H, Wu R, Zhu F, Kosten TR, Zhang X-Y, et al. Minocycline adjunctive treatment to risperidone for negative symptoms in schizophrenia: Association with pro-inflammatory cytokine levels. Progress in Neuro-Psychopharmacology & Biological Psychiatry. 2018;85:69-76.

439. Zhang W-F, Tan Y-L, Zhang X-Y, Chan RCK, Wu H-R, Zhou D-F. Extract of Ginkgo biloba Treatment for Tardive Dyskinesia in Schizophrenia: A Randomized, Double-Blind, Placebo-Controlled Trial. Journal of Clinical Psychiatry. 2011;72(5):615-21.

440. Zhang XY, Zhou DF, Su JM, Zhang PY. The effect of extract of ginkgo biloba added to haloperidol on superoxide dismutase in inpatients with chronic schizophrenia. Journal of Clinical Psychopharmacology. 2001;21(1):85-8.

441. Zhou D, Xie H, Chen L, Zhu Z, Zhang C, Jiang J. The cognitive improvement in patients with schizophrenia following low-intensity repetitive transcranial magnetic stimulation could last for 6 months: A randomized controlled trial. Psychiatry Research. 2024;332.

442. Zhou DF, Zhang XY, Su JM, Nan ZG, Cui Y, Liu J, et al. The effects of classic antipsychotic haloperidol plus the extract of Ginkgo biloba on superoxide dismutase in patients with chronic refractory schizophrenia. Chinese Medical Journal. 1999;112(12):1093-6.

443. Zhou Y, Xia X, Zhao X, Yang R, Wu Y, Liu J, et al. Efficacy and safety of Transcranial Direct Current Stimulation (tDCS) on cognitive function in chronic schizophrenia with Tardive Dyskinesia (TD): a randomized, double-blind, sham-controlled, clinical trial. Bmc Psychiatry. 2023;23(1).

444. Zhu C, Wang X-Y, Zhao J, Long B, Xiao X, Pan L-Y, et al. Effect of transdermal drug delivery therapy on anxiety symptoms in schizophrenic patients. Frontiers in Neuroscience. 2023;17.

445. Zhu S, Wan H, Lu Z, Wu H, Zhang Q, Qian X, et al. Treatment Effect of Antipsychotics in Combination with Horticultural Therapy on Patients with Schizophrenia: A Randomized, Double-blind, Placebo-controlled Study. Shanghai archives of psychiatry. 2016;28(4):195-203.

446. Zhu X, Fan H, Fan F, Zhao Y, Tan Y, Yang F, et al. Improving social functioning in community-dwelling patients with schizophrenia: a randomized controlled computer cognitive remediation therapy trial with six months follow-up. Psychiatry Research. 2020;287.

447. Zhu X, Song H, Chang R, Chen B, Song Y, Liu J, et al. Combining compensatory cognitive training and medication self-management skills training, in inpatients with schizophrenia: A three-arm parallel, single-blind, randomized controlled trial. General Hospital Psychiatry. 2021;69:94-103.

448. Zhuo K, Tang Y, Song Z, Wang Y, Wang J, Qian Z, et al. Repetitive transcranial magnetic stimulation as an adjunctive treatment for negative symptoms and cognitive impairment in patients with schizophrenia: a randomized, double-blind, sham-controlled trial. Neuropsychiatric Disease and Treatment. 2019;15:1141-50.

449. Zimbroff DL, Kane JM, Tamminga CA, Daniel DG, Mack RJ, Wozniak PJ, et al. Controlled, dose-response study of sertindole and haloperidol in the treatment of schizophrenia. American Journal of Psychiatry. 1997;154(6):782-91.

450. Zisook S, Kasckow JW, Golshan S, Fellows I, Solorzano E, Lehman D, et al. Citalopram Augmentation for Subsyndromal Symptoms of Depression in Middle-Aged and Older Outpatients With Schizophrenia and Schizoaffective Disorder: A Randomized Controlled Trial. Journal of Clinical Psychiatry. 2009;70(4):562-71.

451. Zoccali R, Muscatello MR, Bruno A, Cambria R, Mico U, Spina E, et al. The effect of lamotrigine augmentation of clozapine in a sample of treatment-resistant schizophrenic patients: A double-blind, placebo-controlled study. Schizophrenia Research. 2007;93(1-3):109-16.​​
