# Supplementary material for: Interventions for negative symptoms in schizophrenia: efficacy and clinical interpretability in a meta-analysis of 451 randomized controlled trials
Source: Mol Psychiatry. 2026 Mar 23;31(8):4259–69. doi: 10.1038/s41380-026-03543-1 (PMC13364721; doi:10.1038/s41380-026-03543-1)

**Egger's tests and Funnel plots and for each of the 24 subcategories of high-quality studies**

**antipsychotic_second**

| **Meta-Analysis Summary** | |
| --- | --- |
| Data Type | Raw |
| Outcome Type | Continuous |
| Effect Size Measure | Hedges' g |
| Model | Random-effects |
| Weight | Inverse-variance^a^ |
| Estimation Method | REML |
| Standard Error Adjustment | None |
| a. Random-effects weights including both within- and between-study variance. | |

| **Case Processing Summary** | | |
| --- | --- | --- |
|  | N | Percent |
| Included | 25 | 100,0% |
| Missing | 0 | 0,0% |
| Invalid^a^ | 0 | 0,0% |
| Total | 25 | 100,0% |
| a. Nonpositive variance or standard error, or insufficient study size. | | |

| **Effect Size Estimates** | | | | | | |
| --- | --- | --- | --- | --- | --- | --- |
|  | Effect Size | Std. Error | Z | Sig. (2-tailed) | 95% Confidence Interval | |
|  |  |  |  |  | Lower | Upper |
| Overall | -,278 | ,0470 | -5,924 | <,001 | -,370 | -,186 |

| **Egger's Regression-Based Test**^a^ | | | | | | |
| --- | --- | --- | --- | --- | --- | --- |
| Parameter | Coefficient | Std. Error | t | Sig. (2-tailed) | 95% Confidence Interval | |
|  |  |  |  |  | Lower | Upper |
| (Intercept) | -,301 | ,1637 | -1,837 | ,079 | -,639 | ,038 |
| SE^b^ | ,130 | ,9135 | ,143 | ,888 | -1,759 | 2,020 |
| a. Random-effects meta-regression | | | | | | |
| b. Standard error of effect size | | | | | | |

**lifestyle_physical_activity**

| **Meta-Analysis Summary** | |
| --- | --- |
| Data Type | Raw |
| Outcome Type | Continuous |
| Effect Size Measure | Hedges' g |
| Model | Random-effects |
| Weight | Inverse-variance^a^ |
| Estimation Method | REML |
| Standard Error Adjustment | None |
| a. Random-effects weights including both within- and between-study variance. | |

| **Case Processing Summary** | | |
| --- | --- | --- |
|  | N | Percent |
| Included | 7 | 100,0% |
| Missing | 0 | 0,0% |
| Invalid^a^ | 0 | 0,0% |
| Total | 7 | 100,0% |
| a. Nonpositive variance or standard error, or insufficient study size. | | |

| **Effect Size Estimates** | | | | | | |
| --- | --- | --- | --- | --- | --- | --- |
|  | Effect Size | Std. Error | Z | Sig. (2-tailed) | 95% Confidence Interval | |
|  |  |  |  |  | Lower | Upper |
| Overall | -,601 | ,1386 | -4,332 | <,001 | -,872 | -,329 |

| **Egger's Regression-Based Test**^a^ | | | | | | |
| --- | --- | --- | --- | --- | --- | --- |
| Parameter | Coefficient | Std. Error | t | Sig. (2-tailed) | 95% Confidence Interval | |
|  |  |  |  |  | Lower | Upper |
| (Intercept) | -,794 | ,6857 | -1,157 | ,299 | -2,556 | ,969 |
| SE^b^ | ,682 | 2,3320 | ,292 | ,782 | -5,313 | 6,676 |
| a. Random-effects meta-regression | | | | | | |
| b. Standard error of effect size | | | | | | |

**pharma_antibiotics**

| **Meta-Analysis Summary** | |
| --- | --- |
| Data Type | Raw |
| Outcome Type | Continuous |
| Effect Size Measure | Hedges' g |
| Model | Random-effects |
| Weight | Inverse-variance^a^ |
| Estimation Method | REML |
| Standard Error Adjustment | None |
| a. Random-effects weights including both within- and between-study variance. | |

| **Case Processing Summary** | | |
| --- | --- | --- |
|  | N | Percent |
| Included | 3 | 100,0% |
| Missing | 0 | 0,0% |
| Invalid^a^ | 0 | 0,0% |
| Total | 3 | 100,0% |
| a. Nonpositive variance or standard error, or insufficient study size. | | |

| **Effect Size Estimates** | | | | | | |
| --- | --- | --- | --- | --- | --- | --- |
|  | Effect Size | Std. Error | Z | Sig. (2-tailed) | 95% Confidence Interval | |
|  |  |  |  |  | Lower | Upper |
| Overall | -,944 | ,3875 | -2,436 | ,015 | -1,704 | -,184 |

| **Egger's Regression-Based Test**^a^ | | | | | | |
| --- | --- | --- | --- | --- | --- | --- |
| Parameter | Coefficient | Std. Error | t | Sig. (2-tailed) | 95% Confidence Interval | |
|  |  |  |  |  | Lower | Upper |
| (Intercept) | -,850 | 2,4207 | -,351 | ,785 | -31,607 | 29,908 |
| SE^b^ | -,295 | 7,2228 | -,041 | ,974 | -92,070 | 91,480 |
| a. Random-effects meta-regression | | | | | | |
| b. Standard error of effect size | | | | | | |

**pharma_anticonvulsant_mood**

| **Meta-Analysis Summary** | |
| --- | --- |
| Data Type | Raw |
| Outcome Type | Continuous |
| Effect Size Measure | Hedges' g |
| Model | Random-effects |
| Weight | Inverse-variance^a^ |
| Estimation Method | REML |
| Standard Error Adjustment | None |
| a. Random-effects weights including both within- and between-study variance. | |

| **Case Processing Summary** | | |
| --- | --- | --- |
|  | N | Percent |
| Included | 6 | 100,0% |
| Missing | 0 | 0,0% |
| Invalid^a^ | 0 | 0,0% |
| Total | 6 | 100,0% |
| a. Nonpositive variance or standard error, or insufficient study size. | | |

| **Effect Size Estimates** | | | | | | |
| --- | --- | --- | --- | --- | --- | --- |
|  | Effect Size | Std. Error | Z | Sig. (2-tailed) | 95% Confidence Interval | |
|  |  |  |  |  | Lower | Upper |
| Overall | -,322 | ,2537 | -1,270 | ,204 | -,819 | ,175 |

| **Egger's Regression-Based Test**^a^ | | | | | | |
| --- | --- | --- | --- | --- | --- | --- |
| Parameter | Coefficient | Std. Error | t | Sig. (2-tailed) | 95% Confidence Interval | |
|  |  |  |  |  | Lower | Upper |
| (Intercept) | ,867 | ,2342 | 3,703 | ,021 | ,217 | 1,518 |
| SE^b^ | -4,691 | 1,0855 | -4,321 | ,012 | -7,704 | -1,677 |
| a. Random-effects meta-regression | | | | | | |
| b. Standard error of effect size | | | | | | |

**pharma_antidepressant**

| **Meta-Analysis Summary** | |
| --- | --- |
| Data Type | Raw |
| Outcome Type | Continuous |
| Effect Size Measure | Hedges' g |
| Model | Random-effects |
| Weight | Inverse-variance^a^ |
| Estimation Method | REML |
| Standard Error Adjustment | None |
| a. Random-effects weights including both within- and between-study variance. | |

| **Case Processing Summary** | | |
| --- | --- | --- |
|  | N | Percent |
| Included | 14 | 100,0% |
| Missing | 0 | 0,0% |
| Invalid^a^ | 0 | 0,0% |
| Total | 14 | 100,0% |
| a. Nonpositive variance or standard error, or insufficient study size. | | |

| **Effect Size Estimates** | | | | | | |
| --- | --- | --- | --- | --- | --- | --- |
|  | Effect Size | Std. Error | Z | Sig. (2-tailed) | 95% Confidence Interval | |
|  |  |  |  |  | Lower | Upper |
| Overall | -,756 | ,2190 | -3,455 | <,001 | -1,186 | -,327 |

| **Egger's Regression-Based Test**^a^ | | | | | | |
| --- | --- | --- | --- | --- | --- | --- |
| Parameter | Coefficient | Std. Error | t | Sig. (2-tailed) | 95% Confidence Interval | |
|  |  |  |  |  | Lower | Upper |
| (Intercept) | 1,334 | ,8412 | 1,586 | ,139 | -,498 | 3,167 |
| SE^b^ | -6,959 | 2,7454 | -2,535 | ,026 | -12,941 | -,977 |
| a. Random-effects meta-regression | | | | | | |
| b. Standard error of effect size | | | | | | |

**pharma_antiemetic**

| **Meta-Analysis Summary** | |
| --- | --- |
| Data Type | Raw |
| Outcome Type | Continuous |
| Effect Size Measure | Hedges' g |
| Model | Random-effects |
| Weight | Inverse-variance^a^ |
| Estimation Method | REML |
| Standard Error Adjustment | None |
| a. Random-effects weights including both within- and between-study variance. | |

| **Case Processing Summary** | | |
| --- | --- | --- |
|  | N | Percent |
| Included | 4 | 100,0% |
| Missing | 0 | 0,0% |
| Invalid^a^ | 0 | 0,0% |
| Total | 4 | 100,0% |
| a. Nonpositive variance or standard error, or insufficient study size. | | |

| **Effect Size Estimates** | | | | | | |
| --- | --- | --- | --- | --- | --- | --- |
|  | Effect Size | Std. Error | Z | Sig. (2-tailed) | 95% Confidence Interval | |
|  |  |  |  |  | Lower | Upper |
| Overall | -,980 | ,5507 | -1,780 | ,075 | -2,059 | ,099 |

| **Egger's Regression-Based Test**^a^ | | | | | | |
| --- | --- | --- | --- | --- | --- | --- |
| Parameter | Coefficient | Std. Error | t | Sig. (2-tailed) | 95% Confidence Interval | |
|  |  |  |  |  | Lower | Upper |
| (Intercept) | 2,101 | 1,7412 | 1,207 | ,351 | -5,391 | 9,593 |
| SE^b^ | -9,551 | 5,2790 | -1,809 | ,212 | -32,264 | 13,163 |
| a. Random-effects meta-regression | | | | | | |
| b. Standard error of effect size | | | | | | |

**pharma_antihistamine**

| **Meta-Analysis Summary** | |
| --- | --- |
| Data Type | Raw |
| Outcome Type | Continuous |
| Effect Size Measure | Hedges' g |
| Model | Random-effects |
| Weight | Inverse-variance^a^ |
| Estimation Method | REML |
| Standard Error Adjustment | None |
| a. Random-effects weights including both within- and between-study variance. | |

| **Case Processing Summary** | | |
| --- | --- | --- |
|  | N | Percent |
| Included | 3 | 100,0% |
| Missing | 0 | 0,0% |
| Invalid^a^ | 0 | 0,0% |
| Total | 3 | 100,0% |
| a. Nonpositive variance or standard error, or insufficient study size. | | |

| **Effect Size Estimates** | | | | | | |
| --- | --- | --- | --- | --- | --- | --- |
|  | Effect Size | Std. Error | Z | Sig. (2-tailed) | 95% Confidence Interval | |
|  |  |  |  |  | Lower | Upper |
| Overall | -,146 | ,1374 | -1,063 | ,288 | -,415 | ,123 |

| **Egger's Regression-Based Test**^a^ | | | | | | |
| --- | --- | --- | --- | --- | --- | --- |
| Parameter | Coefficient | Std. Error | t | Sig. (2-tailed) | 95% Confidence Interval | |
|  |  |  |  |  | Lower | Upper |
| (Intercept) | ,329 | ,5873 | ,560 | ,675 | -7,134 | 7,792 |
| SE^b^ | -2,052 | 2,4677 | -,832 | ,558 | -33,407 | 29,303 |
| a. Random-effects meta-regression | | | | | | |
| b. Standard error of effect size | | | | | | |

**pharma_antihypertensive**

| **Meta-Analysis Summary** | |
| --- | --- |
| Data Type | Raw |
| Outcome Type | Continuous |
| Effect Size Measure | Hedges' g |
| Model | Random-effects |
| Weight | Inverse-variance^a^ |
| Estimation Method | REML |
| Standard Error Adjustment | None |
| a. Random-effects weights including both within- and between-study variance. | |

| **Case Processing Summary** | | |
| --- | --- | --- |
|  | N | Percent |
| Included | 3 | 100,0% |
| Missing | 0 | 0,0% |
| Invalid^a^ | 0 | 0,0% |
| Total | 3 | 100,0% |
| a. Nonpositive variance or standard error, or insufficient study size. | | |

| **Effect Size Estimates** | | | | | | |
| --- | --- | --- | --- | --- | --- | --- |
|  | Effect Size | Std. Error | Z | Sig. (2-tailed) | 95% Confidence Interval | |
|  |  |  |  |  | Lower | Upper |
| Overall | -,140 | ,1814 | -,769 | ,442 | -,495 | ,216 |

| **Egger's Regression-Based Test**^a^ | | | | | | |
| --- | --- | --- | --- | --- | --- | --- |
| Parameter | Coefficient | Std. Error | t | Sig. (2-tailed) | 95% Confidence Interval | |
|  |  |  |  |  | Lower | Upper |
| (Intercept) | -,572 | ,9622 | -,594 | ,659 | -12,798 | 11,655 |
| SE^b^ | 1,400 | 3,0623 | ,457 | ,727 | -37,510 | 40,310 |
| a. Random-effects meta-regression | | | | | | |
| b. Standard error of effect size | | | | | | |

**pharma_glutamatergic**

| **Meta-Analysis Summary** | |
| --- | --- |
| Data Type | Raw |
| Outcome Type | Continuous |
| Effect Size Measure | Hedges' g |
| Model | Random-effects |
| Weight | Inverse-variance^a^ |
| Estimation Method | REML |
| Standard Error Adjustment | None |
| a. Random-effects weights including both within- and between-study variance. | |

| **Case Processing Summary** | | |
| --- | --- | --- |
|  | N | Percent |
| Included | 10 | 100,0% |
| Missing | 0 | 0,0% |
| Invalid^a^ | 0 | 0,0% |
| Total | 10 | 100,0% |
| a. Nonpositive variance or standard error, or insufficient study size. | | |

| **Effect Size Estimates** | | | | | | |
| --- | --- | --- | --- | --- | --- | --- |
|  | Effect Size | Std. Error | Z | Sig. (2-tailed) | 95% Confidence Interval | |
|  |  |  |  |  | Lower | Upper |
| Overall | -,342 | ,2620 | -1,304 | ,192 | -,855 | ,172 |

| **Egger's Regression-Based Test**^a^ | | | | | | |
| --- | --- | --- | --- | --- | --- | --- |
| Parameter | Coefficient | Std. Error | t | Sig. (2-tailed) | 95% Confidence Interval | |
|  |  |  |  |  | Lower | Upper |
| (Intercept) | 1,013 | ,4521 | 2,241 | ,055 | -,029 | 2,056 |
| SE^b^ | -4,733 | 1,5156 | -3,123 | ,014 | -8,228 | -1,238 |
| a. Random-effects meta-regression | | | | | | |
| b. Standard error of effect size | | | | | | |

**pharma_hormones**

| **Meta-Analysis Summary** | |
| --- | --- |
| Data Type | Raw |
| Outcome Type | Continuous |
| Effect Size Measure | Hedges' g |
| Model | Random-effects |
| Weight | Inverse-variance^a^ |
| Estimation Method | REML |
| Standard Error Adjustment | None |
| a. Random-effects weights including both within- and between-study variance. | |

| **Case Processing Summary** | | |
| --- | --- | --- |
|  | N | Percent |
| Included | 19 | 100,0% |
| Missing | 0 | 0,0% |
| Invalid^a^ | 0 | 0,0% |
| Total | 19 | 100,0% |
| a. Nonpositive variance or standard error, or insufficient study size. | | |

| **Effect Size Estimates** | | | | | | |
| --- | --- | --- | --- | --- | --- | --- |
|  | Effect Size | Std. Error | Z | Sig. (2-tailed) | 95% Confidence Interval | |
|  |  |  |  |  | Lower | Upper |
| Overall | -,395 | ,1066 | -3,710 | <,001 | -,604 | -,187 |

| **Egger's Regression-Based Test**^a^ | | | | | | |
| --- | --- | --- | --- | --- | --- | --- |
| Parameter | Coefficient | Std. Error | t | Sig. (2-tailed) | 95% Confidence Interval | |
|  |  |  |  |  | Lower | Upper |
| (Intercept) | -,737 | ,4661 | -1,582 | ,132 | -1,721 | ,246 |
| SE^b^ | 1,175 | 1,5503 | ,758 | ,459 | -2,096 | 4,445 |
| a. Random-effects meta-regression | | | | | | |
| b. Standard error of effect size | | | | | | |

**pharma_immunomodulator**

| **Meta-Analysis Summary** | |
| --- | --- |
| Data Type | Raw |
| Outcome Type | Continuous |
| Effect Size Measure | Hedges' g |
| Model | Random-effects |
| Weight | Inverse-variance^a^ |
| Estimation Method | REML |
| Standard Error Adjustment | None |
| a. Random-effects weights including both within- and between-study variance. | |

| **Case Processing Summary** | | |
| --- | --- | --- |
|  | N | Percent |
| Included | 7 | 100,0% |
| Missing | 0 | 0,0% |
| Invalid^a^ | 0 | 0,0% |
| Total | 7 | 100,0% |
| a. Nonpositive variance or standard error, or insufficient study size. | | |

| **Effect Size Estimates** | | | | | | |
| --- | --- | --- | --- | --- | --- | --- |
|  | Effect Size | Std. Error | Z | Sig. (2-tailed) | 95% Confidence Interval | |
|  |  |  |  |  | Lower | Upper |
| Overall | -,468 | ,1055 | -4,438 | <,001 | -,675 | -,261 |

| **Egger's Regression-Based Test**^a^ | | | | | | |
| --- | --- | --- | --- | --- | --- | --- |
| Parameter | Coefficient | Std. Error | t | Sig. (2-tailed) | 95% Confidence Interval | |
|  |  |  |  |  | Lower | Upper |
| (Intercept) | -,663 | ,4349 | -1,524 | ,188 | -1,781 | ,455 |
| SE^b^ | ,719 | 1,5584 | ,461 | ,664 | -3,287 | 4,725 |
| a. Random-effects meta-regression | | | | | | |
| b. Standard error of effect size | | | | | | |

**pharma_other**

| **Meta-Analysis Summary** | |
| --- | --- |
| Data Type | Raw |
| Outcome Type | Continuous |
| Effect Size Measure | Hedges' g |
| Model | Random-effects |
| Weight | Inverse-variance^a^ |
| Estimation Method | REML |
| Standard Error Adjustment | None |
| a. Random-effects weights including both within- and between-study variance. | |

| **Case Processing Summary** | | |
| --- | --- | --- |
|  | N | Percent |
| Included | 24 | 100,0% |
| Missing | 0 | 0,0% |
| Invalid^a^ | 0 | 0,0% |
| Total | 24 | 100,0% |
| a. Nonpositive variance or standard error, or insufficient study size. | | |

| **Effect Size Estimates** | | | | | | |
| --- | --- | --- | --- | --- | --- | --- |
|  | Effect Size | Std. Error | Z | Sig. (2-tailed) | 95% Confidence Interval | |
|  |  |  |  |  | Lower | Upper |
| Overall | -,343 | ,1940 | -1,769 | ,077 | -,724 | ,037 |

| **Egger's Regression-Based Test**^a^ | | | | | | |
| --- | --- | --- | --- | --- | --- | --- |
| Parameter | Coefficient | Std. Error | t | Sig. (2-tailed) | 95% Confidence Interval | |
|  |  |  |  |  | Lower | Upper |
| (Intercept) | -,665 | ,6884 | -,967 | ,344 | -2,093 | ,762 |
| SE^b^ | 1,143 | 2,3356 | ,489 | ,629 | -3,701 | 5,987 |
| a. Random-effects meta-regression | | | | | | |
| b. Standard error of effect size | | | | | | |

**pharma_statin**

| **Meta-Analysis Summary** | |
| --- | --- |
| Data Type | Raw |
| Outcome Type | Continuous |
| Effect Size Measure | Hedges' g |
| Model | Random-effects |
| Weight | Inverse-variance^a^ |
| Estimation Method | REML |
| Standard Error Adjustment | None |
| a. Random-effects weights including both within- and between-study variance. | |

| **Case Processing Summary** | | |
| --- | --- | --- |
|  | N | Percent |
| Included | 3 | 100,0% |
| Missing | 0 | 0,0% |
| Invalid^a^ | 0 | 0,0% |
| Total | 3 | 100,0% |
| a. Nonpositive variance or standard error, or insufficient study size. | | |

| **Effect Size Estimates** | | | | | | |
| --- | --- | --- | --- | --- | --- | --- |
|  | Effect Size | Std. Error | Z | Sig. (2-tailed) | 95% Confidence Interval | |
|  |  |  |  |  | Lower | Upper |
| Overall | -,387 | ,1703 | -2,273 | ,023 | -,721 | -,053 |

| **Egger's Regression-Based Test**^a^ | | | | | | |
| --- | --- | --- | --- | --- | --- | --- |
| Parameter | Coefficient | Std. Error | t | Sig. (2-tailed) | 95% Confidence Interval | |
|  |  |  |  |  | Lower | Upper |
| (Intercept) | 1,677 | 1,0265 | 1,634 | ,350 | -11,365 | 14,719 |
| SE^b^ | -9,972 | 4,9905 | -1,998 | ,295 | -73,383 | 53,438 |
| a. Random-effects meta-regression | | | | | | |
| b. Standard error of effect size | | | | | | |

**pharma_stimulant**

| **Meta-Analysis Summary** | |
| --- | --- |
| Data Type | Raw |
| Outcome Type | Continuous |
| Effect Size Measure | Hedges' g |
| Model | Random-effects |
| Weight | Inverse-variance^a^ |
| Estimation Method | REML |
| Standard Error Adjustment | None |
| a. Random-effects weights including both within- and between-study variance. | |

| **Case Processing Summary** | | |
| --- | --- | --- |
|  | N | Percent |
| Included | 5 | 100,0% |
| Missing | 0 | 0,0% |
| Invalid^a^ | 0 | 0,0% |
| Total | 5 | 100,0% |
| a. Nonpositive variance or standard error, or insufficient study size. | | |

| **Effect Size Estimates** | | | | | | |
| --- | --- | --- | --- | --- | --- | --- |
|  | Effect Size | Std. Error | Z | Sig. (2-tailed) | 95% Confidence Interval | |
|  |  |  |  |  | Lower | Upper |
| Overall | -,803 | ,4636 | -1,732 | ,083 | -1,711 | ,106 |

| **Egger's Regression-Based Test**^a^ | | | | | | |
| --- | --- | --- | --- | --- | --- | --- |
| Parameter | Coefficient | Std. Error | t | Sig. (2-tailed) | 95% Confidence Interval | |
|  |  |  |  |  | Lower | Upper |
| (Intercept) | ,122 | 2,0603 | ,059 | ,956 | -6,435 | 6,679 |
| SE^b^ | -2,878 | 6,1920 | -,465 | ,674 | -22,584 | 16,828 |
| a. Random-effects meta-regression | | | | | | |
| b. Standard error of effect size | | | | | | |

**pharma_vitamins_nutraceutic**

| **Meta-Analysis Summary** | |
| --- | --- |
| Data Type | Raw |
| Outcome Type | Continuous |
| Effect Size Measure | Hedges' g |
| Model | Random-effects |
| Weight | Inverse-variance^a^ |
| Estimation Method | REML |
| Standard Error Adjustment | None |
| a. Random-effects weights including both within- and between-study variance. | |

| **Case Processing Summary** | | |
| --- | --- | --- |
|  | N | Percent |
| Included | 24 | 100,0% |
| Missing | 0 | 0,0% |
| Invalid^a^ | 0 | 0,0% |
| Total | 24 | 100,0% |
| a. Nonpositive variance or standard error, or insufficient study size. | | |

| **Effect Size Estimates** | | | | | | |
| --- | --- | --- | --- | --- | --- | --- |
|  | Effect Size | Std. Error | Z | Sig. (2-tailed) | 95% Confidence Interval | |
|  |  |  |  |  | Lower | Upper |
| Overall | -,421 | ,0833 | -5,053 | <,001 | -,584 | -,258 |

| **Egger's Regression-Based Test**^a^ | | | | | | |
| --- | --- | --- | --- | --- | --- | --- |
| Parameter | Coefficient | Std. Error | t | Sig. (2-tailed) | 95% Confidence Interval | |
|  |  |  |  |  | Lower | Upper |
| (Intercept) | -,014 | ,3179 | -,043 | ,966 | -,673 | ,646 |
| SE^b^ | -1,515 | 1,1486 | -1,319 | ,201 | -3,897 | ,867 |
| a. Random-effects meta-regression | | | | | | |
| b. Standard error of effect size | | | | | | |

**psych_art**

| **Meta-Analysis Summary** | |
| --- | --- |
| Data Type | Raw |
| Outcome Type | Continuous |
| Effect Size Measure | Hedges' g |
| Model | Random-effects |
| Weight | Inverse-variance^a^ |
| Estimation Method | REML |
| Standard Error Adjustment | None |
| a. Random-effects weights including both within- and between-study variance. | |

| **Case Processing Summary** | | |
| --- | --- | --- |
|  | N | Percent |
| Included | 4 | 100,0% |
| Missing | 0 | 0,0% |
| Invalid^a^ | 0 | 0,0% |
| Total | 4 | 100,0% |
| a. Nonpositive variance or standard error, or insufficient study size. | | |

| **Effect Size Estimates** | | | | | | |
| --- | --- | --- | --- | --- | --- | --- |
|  | Effect Size | Std. Error | Z | Sig. (2-tailed) | 95% Confidence Interval | |
|  |  |  |  |  | Lower | Upper |
| Overall | -,422 | ,2187 | -1,927 | ,054 | -,850 | ,007 |

| **Egger's Regression-Based Test**^a^ | | | | | | |
| --- | --- | --- | --- | --- | --- | --- |
| Parameter | Coefficient | Std. Error | t | Sig. (2-tailed) | 95% Confidence Interval | |
|  |  |  |  |  | Lower | Upper |
| (Intercept) | ,949 | ,5506 | 1,723 | ,227 | -1,420 | 3,318 |
| SE^b^ | -6,348 | 2,5999 | -2,441 | ,135 | -17,534 | 4,839 |
| a. Random-effects meta-regression | | | | | | |
| b. Standard error of effect size | | | | | | |

**psych_cog_cbt**

| **Meta-Analysis Summary** | |
| --- | --- |
| Data Type | Raw |
| Outcome Type | Continuous |
| Effect Size Measure | Hedges' g |
| Model | Random-effects |
| Weight | Inverse-variance^a^ |
| Estimation Method | REML |
| Standard Error Adjustment | None |
| a. Random-effects weights including both within- and between-study variance. | |

| **Case Processing Summary** | | |
| --- | --- | --- |
|  | N | Percent |
| Included | 13 | 100,0% |
| Missing | 0 | 0,0% |
| Invalid^a^ | 0 | 0,0% |
| Total | 13 | 100,0% |
| a. Nonpositive variance or standard error, or insufficient study size. | | |

| **Effect Size Estimates** | | | | | | |
| --- | --- | --- | --- | --- | --- | --- |
|  | Effect Size | Std. Error | Z | Sig. (2-tailed) | 95% Confidence Interval | |
|  |  |  |  |  | Lower | Upper |
| Overall | -,365 | ,1166 | -3,130 | ,002 | -,593 | -,136 |

| **Egger's Regression-Based Test**^a^ | | | | | | |
| --- | --- | --- | --- | --- | --- | --- |
| Parameter | Coefficient | Std. Error | t | Sig. (2-tailed) | 95% Confidence Interval | |
|  |  |  |  |  | Lower | Upper |
| (Intercept) | ,092 | ,2942 | ,312 | ,761 | -,556 | ,739 |
| SE^b^ | -1,797 | 1,0904 | -1,648 | ,128 | -4,197 | ,603 |
| a. Random-effects meta-regression | | | | | | |
| b. Standard error of effect size | | | | | | |

**psych_cognitive_remediation**

| **Meta-Analysis Summary** | |
| --- | --- |
| Data Type | Raw |
| Outcome Type | Continuous |
| Effect Size Measure | Hedges' g |
| Model | Random-effects |
| Weight | Inverse-variance^a^ |
| Estimation Method | REML |
| Standard Error Adjustment | None |
| a. Random-effects weights including both within- and between-study variance. | |

| **Case Processing Summary** | | |
| --- | --- | --- |
|  | N | Percent |
| Included | 18 | 100,0% |
| Missing | 0 | 0,0% |
| Invalid^a^ | 0 | 0,0% |
| Total | 18 | 100,0% |
| a. Nonpositive variance or standard error, or insufficient study size. | | |

| **Effect Size Estimates** | | | | | | |
| --- | --- | --- | --- | --- | --- | --- |
|  | Effect Size | Std. Error | Z | Sig. (2-tailed) | 95% Confidence Interval | |
|  |  |  |  |  | Lower | Upper |
| Overall | -,040 | ,0628 | -,636 | ,525 | -,163 | ,083 |

| **Egger's Regression-Based Test^a^** | | | | | | |
| --- | --- | --- | --- | --- | --- | --- |
| Parameter | Coefficient | Std. Error | t | Sig. (2-tailed) | 95% Confidence Interval | |
|  |  |  |  |  | Lower | Upper |
| (Intercept) | ,081 | ,2763 | ,293 | ,773 | -,505 | ,667 |
| SE^b^ | -,466 | 1,0375 | -,449 | ,659 | -2,666 | 1,733 |
| a. Random-effects meta-regression | | | | | | |
| b. Standard error of effect size | | | | | | |


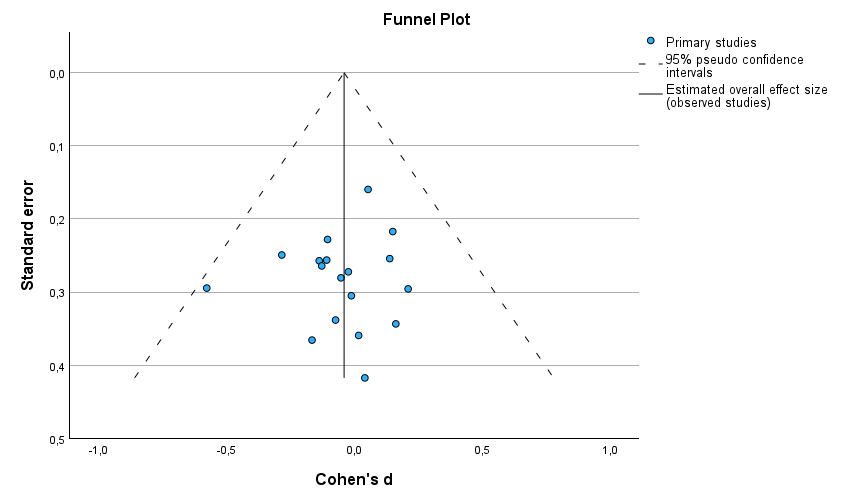


**psych_mindfulness**

| **Meta-Analysis Summary** | |
| --- | --- |
| Data Type | Raw |
| Outcome Type | Continuous |
| Effect Size Measure | Hedges' g |
| Model | Random-effects |
| Weight | Inverse-variance^a^ |
| Estimation Method | REML |
| Standard Error Adjustment | None |
| a. Random-effects weights including both within- and between-study variance. | |

| **Case Processing Summary** | | |
| --- | --- | --- |
|  | N | Percent |
| Included | 7 | 100,0% |
| Missing | 0 | 0,0% |
| Invalid^a^ | 0 | 0,0% |
| Total | 7 | 100,0% |
| a. Nonpositive variance or standard error, or insufficient study size. | | |

| **Effect Size Estimates** | | | | | | |
| --- | --- | --- | --- | --- | --- | --- |
|  | Effect Size | Std. Error | Z | Sig. (2-tailed) | 95% Confidence Interval | |
|  |  |  |  |  | Lower | Upper |
| Overall | -,731 | ,1727 | -4,231 | <,001 | -1,069 | -,392 |

| **Egger's Regression-Based Test**^a^ | | | | | | |
| --- | --- | --- | --- | --- | --- | --- |
| Parameter | Coefficient | Std. Error | t | Sig. (2-tailed) | 95% Confidence Interval | |
|  |  |  |  |  | Lower | Upper |
| (Intercept) | -,006 | ,5838 | -,011 | ,992 | -1,507 | 1,494 |
| SE^b^ | -2,564 | 2,0048 | -1,279 | ,257 | -7,718 | 2,590 |
| a. Random-effects meta-regression | | | | | | |
| b. Standard error of effect size | | | | | | |

**psych_psychoeducation_support**

| **Meta-Analysis Summary** | |
| --- | --- |
| Data Type | Raw |
| Outcome Type | Continuous |
| Effect Size Measure | Hedges' g |
| Model | Random-effects |
| Weight | Inverse-variance^a^ |
| Estimation Method | REML |
| Standard Error Adjustment | None |
| a. Random-effects weights including both within- and between-study variance. | |

| **Case Processing Summary** | | |
| --- | --- | --- |
|  | N | Percent |
| Included | 8 | 100,0% |
| Missing | 0 | 0,0% |
| Invalid^a^ | 0 | 0,0% |
| Total | 8 | 100,0% |
| a. Nonpositive variance or standard error, or insufficient study size. | | |

| **Effect Size Estimates** | | | | | | |
| --- | --- | --- | --- | --- | --- | --- |
|  | Effect Size | Std. Error | Z | Sig. (2-tailed) | 95% Confidence Interval | |
|  |  |  |  |  | Lower | Upper |
| Overall | -,357 | ,1807 | -1,978 | ,048 | -,711 | -,003 |

| **Egger's Regression-Based Test**^a^ | | | | | | |
| --- | --- | --- | --- | --- | --- | --- |
| Parameter | Coefficient | Std. Error | t | Sig. (2-tailed) | 95% Confidence Interval | |
|  |  |  |  |  | Lower | Upper |
| (Intercept) | -1,159 | 1,4049 | -,825 | ,441 | -4,597 | 2,278 |
| SE^b^ | 3,333 | 5,7798 | ,577 | ,585 | -10,810 | 17,475 |
| a. Random-effects meta-regression | | | | | | |
| b. Standard error of effect size | | | | | | |

**psych_social_skills**

| **Meta-Analysis Summary** | |
| --- | --- |
| Data Type | Raw |
| Outcome Type | Continuous |
| Effect Size Measure | Hedges' g |
| Model | Random-effects |
| Weight | Inverse-variance^a^ |
| Estimation Method | REML |
| Standard Error Adjustment | None |
| a. Random-effects weights including both within- and between-study variance. | |

| **Case Processing Summary** | | |
| --- | --- | --- |
|  | N | Percent |
| Included | 6 | 100,0% |
| Missing | 0 | 0,0% |
| Invalid^a^ | 0 | 0,0% |
| Total | 6 | 100,0% |
| a. Nonpositive variance or standard error, or insufficient study size. | | |

| **Effect Size Estimates** | | | | | | |
| --- | --- | --- | --- | --- | --- | --- |
|  | Effect Size | Std. Error | Z | Sig. (2-tailed) | 95% Confidence Interval | |
|  |  |  |  |  | Lower | Upper |
| Overall | ,013 | ,0739 | ,173 | ,862 | -,132 | ,158 |

| **Egger's Regression-Based Test^a^** | | | | | | |
| --- | --- | --- | --- | --- | --- | --- |
| Parameter | Coefficient | Std. Error | t | Sig. (2-tailed) | 95% Confidence Interval | |
|  |  |  |  |  | Lower | Upper |
| (Intercept) | -,273 | ,2772 | -,985 | ,381 | -1,042 | ,497 |
| SE^b^ | 1,638 | 1,5315 | 1,070 | ,345 | -2,614 | 5,890 |
| a. Random-effects meta-regression | | | | | | |
| b. Standard error of effect size | | | | | | |


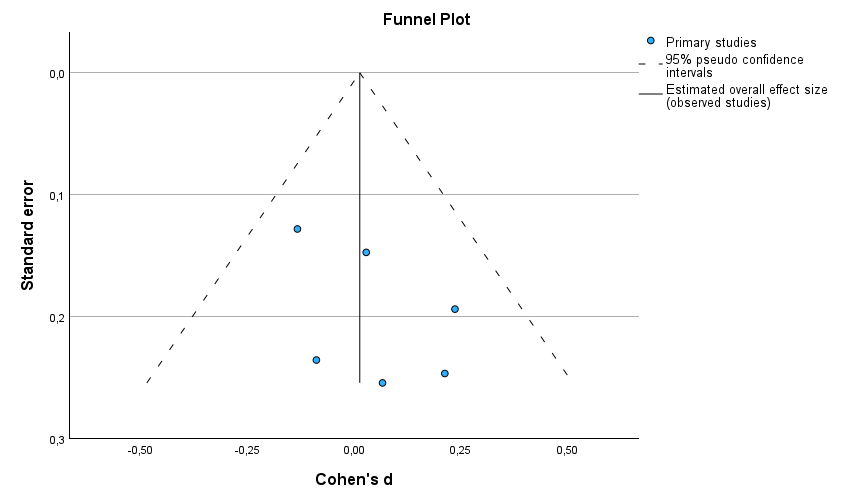


**stimulation_TCS**

| **Meta-Analysis Summary** | |
| --- | --- |
| Data Type | Raw |
| Outcome Type | Continuous |
| Effect Size Measure | Hedges' g |
| Model | Random-effects |
| Weight | Inverse-variance^a^ |
| Estimation Method | REML |
| Standard Error Adjustment | None |
| a. Random-effects weights including both within- and between-study variance. | |

| **Case Processing Summary** | | |
| --- | --- | --- |
|  | N | Percent |
| Included | 14 | 100,0% |
| Missing | 0 | 0,0% |
| Invalid^a^ | 0 | 0,0% |
| Total | 14 | 100,0% |
| a. Nonpositive variance or standard error, or insufficient study size. | | |

| **Effect Size Estimates** | | | | | | |
| --- | --- | --- | --- | --- | --- | --- |
|  | Effect Size | Std. Error | Z | Sig. (2-tailed) | 95% Confidence Interval | |
|  |  |  |  |  | Lower | Upper |
| Overall | -,514 | ,1770 | -2,903 | ,004 | -,861 | -,167 |

| **Egger's Regression-Based Test**^a^ | | | | | | |
| --- | --- | --- | --- | --- | --- | --- |
| Parameter | Coefficient | Std. Error | t | Sig. (2-tailed) | 95% Confidence Interval | |
|  |  |  |  |  | Lower | Upper |
| (Intercept) | ,259 | ,7988 | ,325 | ,751 | -1,481 | 2,000 |
| SE^b^ | -2,351 | 2,3664 | -,994 | ,340 | -7,507 | 2,805 |
| a. Random-effects meta-regression | | | | | | |
| b. Standard error of effect size | | | | | | |

**stimulation_TMS**

| **Meta-Analysis Summary** | |
| --- | --- |
| Data Type | Raw |
| Outcome Type | Continuous |
| Effect Size Measure | Hedges' g |
| Model | Random-effects |
| Weight | Inverse-variance^a^ |
| Estimation Method | REML |
| Standard Error Adjustment | None |
| a. Random-effects weights including both within- and between-study variance. | |

| **Case Processing Summary** | | |
| --- | --- | --- |
|  | N | Percent |
| Included | 32 | 100,0% |
| Missing | 0 | 0,0% |
| Invalid^a^ | 0 | 0,0% |
| Total | 32 | 100,0% |
| a. Nonpositive variance or standard error, or insufficient study size. | | |

| **Effect Size Estimates** | | | | | | |
| --- | --- | --- | --- | --- | --- | --- |
|  | Effect Size | Std. Error | Z | Sig. (2-tailed) | 95% Confidence Interval | |
|  |  |  |  |  | Lower | Upper |
| Overall | -,234 | ,0857 | -2,729 | ,006 | -,402 | -,066 |

| **Egger's Regression-Based Test**^a^ | | | | | | |
| --- | --- | --- | --- | --- | --- | --- |
| Parameter | Coefficient | Std. Error | t | Sig. (2-tailed) | 95% Confidence Interval | |
|  |  |  |  |  | Lower | Upper |
| (Intercept) | -,163 | ,3606 | -,452 | ,655 | -,899 | ,574 |
| SE^b^ | -,240 | 1,1804 | -,204 | ,840 | -2,651 | 2,170 |
| a. Random-effects meta-regression | | | | | | |
| b. Standard error of effect size | | | | | | |

**psych_integrated**

| **Meta-Analysis Summary** | |
| --- | --- |
| Data Type | Raw |
| Outcome Type | Continuous |
| Effect Size Measure | Hedges' g |
| Model | Random-effects |
| Weight | Inverse-variance^a^ |
| Estimation Method | REML |
| Standard Error Adjustment | None |
| a. Random-effects weights including both within- and between-study variance. | |

| **Case Processing Summary** | | |
| --- | --- | --- |
|  | N | Percent |
| Included | 7 | 100,0% |
| Missing | 0 | 0,0% |
| Invalid^a^ | 0 | 0,0% |
| Total | 7 | 100,0% |
| a. Nonpositive variance or standard error, or insufficient study size. | | |

| **Effect Size Estimates** | | | | | | |
| --- | --- | --- | --- | --- | --- | --- |
|  | Effect Size | Std. Error | Z | Sig. (2-tailed) | 95% Confidence Interval | |
|  |  |  |  |  | Lower | Upper |
| Overall | ,020 | ,1088 | ,181 | ,856 | -,194 | ,233 |

| **Egger's Regression-Based Test^a^** | | | | | | |
| --- | --- | --- | --- | --- | --- | --- |
| Parameter | Coefficient | Std. Error | t | Sig. (2-tailed) | 95% Confidence Interval | |
|  |  |  |  |  | Lower | Upper |
| (Intercept) | -,013 | ,4748 | -,027 | ,980 | -1,233 | 1,208 |
| SE^b^ | ,136 | 1,7507 | ,077 | ,941 | -4,365 | 4,636 |
| a. Random-effects meta-regression | | | | | | |
| b. Standard error of effect size | | | | | | |


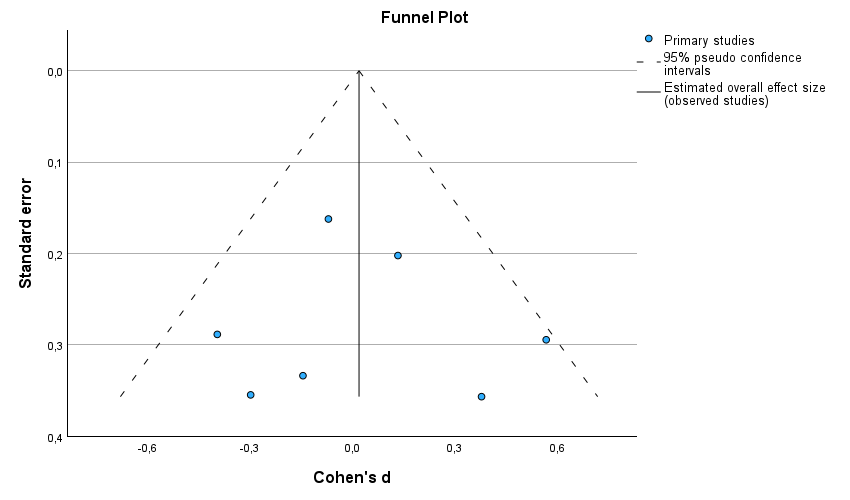

Supplement: Supplementary file 2 — Supplementary Materials 2 Funnel_Eggers_high_quality_studies [file 41380_2026_3543_MOESM2_ESM.docx]
