# Supplementary material for: Interventions for negative symptoms in schizophrenia: efficacy and clinical interpretability in a meta-analysis of 451 randomized controlled trials
Source: Mol Psychiatry. 2026 Mar 23;31(8):4259–69. doi: 10.1038/s41380-026-03543-1 (PMC13364721; doi:10.1038/s41380-026-03543-1)
Supplement: Supplementary file 3 — Supplementary Materials 3 - Funnel_Eggers_whole_sample_studies [file 41380_2026_3543_MOESM3_ESM.docx]

**Egger's tests and Funnel plots for each of the 27 subcategories of whole-sample studies**

**antipsychotic_first**

| **Meta-Analysis Summary** | |
| --- | --- |
| Data Type | Raw |
| Outcome Type | Continuous |
| Effect Size Measure | Hedges' g |
| Model | Random-effects |
| Weight | Inverse-variance^a^ |
| Estimation Method | REML |
| Standard Error Adjustment | None |
| a. Random-effects weights including both within- and between-study variance. | |

| **Case Processing Summary** | | |
| --- | --- | --- |
|  | N | Percent |
| Included | 11 | 100,0% |
| Missing | 0 | 0,0% |
| Invalid^a^ | 0 | 0,0% |
| Total | 11 | 100,0% |
| a. Nonpositive variance or standard error, or insufficient study size. | | |

| **Effect Size Estimates** | | | | | | |
| --- | --- | --- | --- | --- | --- | --- |
|  | Effect Size | Std. Error | Z | Sig. (2-tailed) | 95% Confidence Interval | |
|  |  |  |  |  | Lower | Upper |
| Overall | -,235 | ,0923 | -2,545 | ,011 | -,416 | -,054 |

| **Egger's Regression-Based Test**^a^ | | | | | | |
| --- | --- | --- | --- | --- | --- | --- |
| Parameter | Coefficient | Std. Error | t | Sig. (2-tailed) | 95% Confidence Interval | |
|  |  |  |  |  | Lower | Upper |
| (Intercept) | -,593 | ,3463 | -1,712 | ,121 | -1,376 | ,191 |
| SE^b^ | 1,301 | 1,2013 | 1,083 | ,307 | -1,417 | 4,019 |
| a. Random-effects meta-regression | | | | | | |
| b. Standard error of effect size | | | | | | |

**antipsychotic_second**

| **Meta-Analysis Summary** | |
| --- | --- |
| Data Type | Raw |
| Outcome Type | Continuous |
| Effect Size Measure | Hedges' g |
| Model | Random-effects |
| Weight | Inverse-variance^a^ |
| Estimation Method | REML |
| Standard Error Adjustment | None |
| a. Random-effects weights including both within- and between-study variance. | |

| **Case Processing Summary** | | |
| --- | --- | --- |
|  | N | Percent |
| Included | 92 | 100,0% |
| Missing | 0 | 0,0% |
| Invalid^a^ | 0 | 0,0% |
| Total | 92 | 100,0% |
| a. Nonpositive variance or standard error, or insufficient study size. | | |

| **Effect Size Estimates** | | | | | | |
| --- | --- | --- | --- | --- | --- | --- |
|  | Effect Size | Std. Error | Z | Sig. (2-tailed) | 95% Confidence Interval | |
|  |  |  |  |  | Lower | Upper |
| Overall | -,350 | ,0282 | -12,438 | <,001 | -,405 | -,295 |

| **Egger's Regression-Based Test**^a^ | | | | | | |
| --- | --- | --- | --- | --- | --- | --- |
| Parameter | Coefficient | Std. Error | t | Sig. (2-tailed) | 95% Confidence Interval | |
|  |  |  |  |  | Lower | Upper |
| (Intercept) | -,227 | ,0847 | -2,683 | ,009 | -,395 | -,059 |
| SE^b^ | -,592 | ,3854 | -1,535 | ,128 | -1,357 | ,174 |
| a. Random-effects meta-regression | | | | | | |
| b. Standard error of effect size | | | | | | |

**antipsychotic_third**

| **Meta-Analysis Summary** | |
| --- | --- |
| Data Type | Raw |
| Outcome Type | Continuous |
| Effect Size Measure | Hedges' g |
| Model | Random-effects |
| Weight | Inverse-variance^a^ |
| Estimation Method | REML |
| Standard Error Adjustment | None |
| a. Random-effects weights including both within- and between-study variance. | |

| **Case Processing Summary** | | |
| --- | --- | --- |
|  | N | Percent |
| Included | 9 | 100,0% |
| Missing | 0 | 0,0% |
| Invalid^a^ | 0 | 0,0% |
| Total | 9 | 100,0% |
| a. Nonpositive variance or standard error, or insufficient study size. | | |

| **Effect Size Estimates** | | | | | | |
| --- | --- | --- | --- | --- | --- | --- |
|  | Effect Size | Std. Error | Z | Sig. (2-tailed) | 95% Confidence Interval | |
|  |  |  |  |  | Lower | Upper |
| Overall | -,287 | ,0739 | -3,887 | <,001 | -,432 | -,142 |

| **Egger's Regression-Based Test**^a^ | | | | | | |
| --- | --- | --- | --- | --- | --- | --- |
| Parameter | Coefficient | Std. Error | t | Sig. (2-tailed) | 95% Confidence Interval | |
|  |  |  |  |  | Lower | Upper |
| (Intercept) | -,242 | ,4028 | -,600 | ,567 | -1,194 | ,711 |
| SE^b^ | -,210 | 1,8161 | -,115 | ,911 | -4,504 | 4,085 |
| a. Random-effects meta-regression | | | | | | |
| b. Standard error of effect size | | | | | | |

**lifestyle_physical_activity**

| **Meta-Analysis Summary** | |
| --- | --- |
| Data Type | Raw |
| Outcome Type | Continuous |
| Effect Size Measure | Hedges' g |
| Model | Random-effects |
| Weight | Inverse-variance^a^ |
| Estimation Method | REML |
| Standard Error Adjustment | None |
| a. Random-effects weights including both within- and between-study variance. | |

| **Case Processing Summary** | | |
| --- | --- | --- |
|  | N | Percent |
| Included | 18 | 100,0% |
| Missing | 0 | 0,0% |
| Invalid^a^ | 0 | 0,0% |
| Total | 18 | 100,0% |
| a. Nonpositive variance or standard error, or insufficient study size. | | |

| **Effect Size Estimates** | | | | | | |
| --- | --- | --- | --- | --- | --- | --- |
|  | Effect Size | Std. Error | Z | Sig. (2-tailed) | 95% Confidence Interval | |
|  |  |  |  |  | Lower | Upper |
| Overall | -,617 | ,1023 | -6,027 | <,001 | -,817 | -,416 |

| **Egger's Regression-Based Test**^a^ | | | | | | |
| --- | --- | --- | --- | --- | --- | --- |
| Parameter | Coefficient | Std. Error | t | Sig. (2-tailed) | 95% Confidence Interval | |
|  |  |  |  |  | Lower | Upper |
| (Intercept) | -,199 | ,5485 | -,363 | ,722 | -1,362 | ,964 |
| SE^b^ | -1,304 | 1,6764 | -,778 | ,448 | -4,858 | 2,250 |
| a. Random-effects meta-regression | | | | | | |
| b. Standard error of effect size | | | | | | |

**pharma_antibiotics**

| **Meta-Analysis Summary** | |
| --- | --- |
| Data Type | Raw |
| Outcome Type | Continuous |
| Effect Size Measure | Hedges' g |
| Model | Random-effects |
| Weight | Inverse-variance^a^ |
| Estimation Method | REML |
| Standard Error Adjustment | None |
| a. Random-effects weights including both within- and between-study variance. | |

| **Case Processing Summary** | | |
| --- | --- | --- |
|  | N | Percent |
| Included | 9 | 100,0% |
| Missing | 0 | 0,0% |
| Invalid^a^ | 0 | 0,0% |
| Total | 9 | 100,0% |
| a. Nonpositive variance or standard error, or insufficient study size. | | |

| **Effect Size Estimates** | | | | | | |
| --- | --- | --- | --- | --- | --- | --- |
|  | Effect Size | Std. Error | Z | Sig. (2-tailed) | 95% Confidence Interval | |
|  |  |  |  |  | Lower | Upper |
| Overall | -,605 | ,1549 | -3,903 | <,001 | -,908 | -,301 |

| **Egger's Regression-Based Test**^a^ | | | | | | |
| --- | --- | --- | --- | --- | --- | --- |
| Parameter | Coefficient | Std. Error | t | Sig. (2-tailed) | 95% Confidence Interval | |
|  |  |  |  |  | Lower | Upper |
| (Intercept) | ,323 | ,5093 | ,634 | ,546 | -,881 | 1,527 |
| SE^b^ | -3,371 | 1,8151 | -1,857 | ,106 | -7,663 | ,921 |
| a. Random-effects meta-regression | | | | | | |
| b. Standard error of effect size | | | | | | |

**pharma_anticholinesterase**

| **Meta-Analysis Summary** | |
| --- | --- |
| Data Type | Raw |
| Outcome Type | Continuous |
| Effect Size Measure | Hedges' g |
| Model | Random-effects |
| Weight | Inverse-variance^a^ |
| Estimation Method | REML |
| Standard Error Adjustment | None |
| a. Random-effects weights including both within- and between-study variance. | |

| **Case Processing Summary** | | |
| --- | --- | --- |
|  | N | Percent |
| Included | 3 | 100,0% |
| Missing | 0 | 0,0% |
| Invalid^a^ | 0 | 0,0% |
| Total | 3 | 100,0% |
| a. Nonpositive variance or standard error, or insufficient study size. | | |

| **Effect Size Estimates** | | | | | | |
| --- | --- | --- | --- | --- | --- | --- |
|  | Effect Size | Std. Error | Z | Sig. (2-tailed) | 95% Confidence Interval | |
|  |  |  |  |  | Lower | Upper |
| Overall | -,415 | ,3479 | -1,193 | ,233 | -1,097 | ,267 |

| **Egger's Regression-Based Test**^a^ | | | | | | |
| --- | --- | --- | --- | --- | --- | --- |
| Parameter | Coefficient | Std. Error | t | Sig. (2-tailed) | 95% Confidence Interval | |
|  |  |  |  |  | Lower | Upper |
| (Intercept) | 3,898 | 5,1573 | ,756 | ,588 | -61,632 | 69,428 |
| SE^b^ | -11,672 | 13,9071 | -,839 | ,555 | -188,378 | 165,035 |
| a. Random-effects meta-regression | | | | | | |
| b. Standard error of effect size | | | | | | |

**pharma_anticonvulsant_mood**

| **Meta-Analysis Summary** | |
| --- | --- |
| Data Type | Raw |
| Outcome Type | Continuous |
| Effect Size Measure | Hedges' g |
| Model | Random-effects |
| Weight | Inverse-variance^a^ |
| Estimation Method | REML |
| Standard Error Adjustment | None |
| a. Random-effects weights including both within- and between-study variance. | |

| **Case Processing Summary** | | |
| --- | --- | --- |
|  | N | Percent |
| Included | 9 | 100,0% |
| Missing | 0 | 0,0% |
| Invalid^a^ | 0 | 0,0% |
| Total | 9 | 100,0% |
| a. Nonpositive variance or standard error, or insufficient study size. | | |

| **Effect Size Estimates** | | | | | | |
| --- | --- | --- | --- | --- | --- | --- |
|  | Effect Size | Std. Error | Z | Sig. (2-tailed) | 95% Confidence Interval | |
|  |  |  |  |  | Lower | Upper |
| Overall | -,352 | ,2143 | -1,642 | ,101 | -,772 | ,068 |

| **Egger's Regression-Based Test**^a^ | | | | | | |
| --- | --- | --- | --- | --- | --- | --- |
| Parameter | Coefficient | Std. Error | t | Sig. (2-tailed) | 95% Confidence Interval | |
|  |  |  |  |  | Lower | Upper |
| (Intercept) | ,829 | ,5197 | 1,595 | ,155 | -,400 | 2,058 |
| SE^b^ | -4,356 | 1,8567 | -2,346 | ,051 | -8,747 | ,034 |
| a. Random-effects meta-regression | | | | | | |
| b. Standard error of effect size | | | | | | |

**pharma_antidepressant**

| **Meta-Analysis Summary** | |
| --- | --- |
| Data Type | Raw |
| Outcome Type | Continuous |
| Effect Size Measure | Hedges' g |
| Model | Random-effects |
| Weight | Inverse-variance^a^ |
| Estimation Method | REML |
| Standard Error Adjustment | None |
| a. Random-effects weights including both within- and between-study variance. | |

| **Case Processing Summary** | | |
| --- | --- | --- |
|  | N | Percent |
| Included | 39 | 100,0% |
| Missing | 0 | 0,0% |
| Invalid^a^ | 0 | 0,0% |
| Total | 39 | 100,0% |
| a. Nonpositive variance or standard error, or insufficient study size. | | |

| **Effect Size Estimates** | | | | | | |
| --- | --- | --- | --- | --- | --- | --- |
|  | Effect Size | Std. Error | Z | Sig. (2-tailed) | 95% Confidence Interval | |
|  |  |  |  |  | Lower | Upper |
| Overall | -,593 | ,1105 | -5,362 | <,001 | -,809 | -,376 |

| **Egger's Regression-Based Test**^a^ | | | | | | |
| --- | --- | --- | --- | --- | --- | --- |
| Parameter | Coefficient | Std. Error | t | Sig. (2-tailed) | 95% Confidence Interval | |
|  |  |  |  |  | Lower | Upper |
| (Intercept) | ,026 | ,3755 | ,070 | ,945 | -,735 | ,787 |
| SE^b^ | -2,000 | 1,1655 | -1,716 | ,095 | -4,362 | ,362 |
| a. Random-effects meta-regression | | | | | | |
| b. Standard error of effect size | | | | | | |

**pharma_antiemetic**

| **Meta-Analysis Summary** | |
| --- | --- |
| Data Type | Raw |
| Outcome Type | Continuous |
| Effect Size Measure | Hedges' g |
| Model | Random-effects |
| Weight | Inverse-variance^a^ |
| Estimation Method | REML |
| Standard Error Adjustment | None |
| a. Random-effects weights including both within- and between-study variance. | |

| **Case Processing Summary** | | |
| --- | --- | --- |
|  | N | Percent |
| Included | 7 | 100,0% |
| Missing | 0 | 0,0% |
| Invalid^a^ | 0 | 0,0% |
| Total | 7 | 100,0% |
| a. Nonpositive variance or standard error, or insufficient study size. | | |

| **Effect Size Estimates** | | | | | | |
| --- | --- | --- | --- | --- | --- | --- |
|  | Effect Size | Std. Error | Z | Sig. (2-tailed) | 95% Confidence Interval | |
|  |  |  |  |  | Lower | Upper |
| Overall | -1,016 | ,3282 | -3,096 | ,002 | -1,660 | -,373 |

| **Egger's Regression-Based Test**^a^ | | | | | | |
| --- | --- | --- | --- | --- | --- | --- |
| Parameter | Coefficient | Std. Error | t | Sig. (2-tailed) | 95% Confidence Interval | |
|  |  |  |  |  | Lower | Upper |
| (Intercept) | ,420 | 1,3230 | ,317 | ,764 | -2,981 | 3,820 |
| SE^b^ | -3,957 | 3,5476 | -1,115 | ,315 | -13,076 | 5,162 |
| a. Random-effects meta-regression | | | | | | |
| b. Standard error of effect size | | | | | | |

**pharma_antihistamine**

| **Meta-Analysis Summary** | |
| --- | --- |
| Data Type | Raw |
| Outcome Type | Continuous |
| Effect Size Measure | Hedges' g |
| Model | Random-effects |
| Weight | Inverse-variance^a^ |
| Estimation Method | REML |
| Standard Error Adjustment | None |
| a. Random-effects weights including both within- and between-study variance. | |

| **Case Processing Summary** | | |
| --- | --- | --- |
|  | N | Percent |
| Included | 4 | 100,0% |
| Missing | 0 | 0,0% |
| Invalid^a^ | 0 | 0,0% |
| Total | 4 | 100,0% |
| a. Nonpositive variance or standard error, or insufficient study size. | | |

| **Effect Size Estimates** | | | | | | |
| --- | --- | --- | --- | --- | --- | --- |
|  | Effect Size | Std. Error | Z | Sig. (2-tailed) | 95% Confidence Interval | |
|  |  |  |  |  | Lower | Upper |
| Overall | -,236 | ,1228 | -1,921 | ,055 | -,477 | ,005 |

| **Egger's Regression-Based Test**^a^ | | | | | | |
| --- | --- | --- | --- | --- | --- | --- |
| Parameter | Coefficient | Std. Error | t | Sig. (2-tailed) | 95% Confidence Interval | |
|  |  |  |  |  | Lower | Upper |
| (Intercept) | ,468 | ,5737 | ,816 | ,500 | -2,000 | 2,937 |
| SE^b^ | -2,934 | 2,3361 | -1,256 | ,336 | -12,986 | 7,117 |
| a. Random-effects meta-regression | | | | | | |
| b. Standard error of effect size | | | | | | |

**pharma_antihypertensive**

| **Meta-Analysis Summary** | |
| --- | --- |
| Data Type | Raw |
| Outcome Type | Continuous |
| Effect Size Measure | Hedges' g |
| Model | Random-effects |
| Weight | Inverse-variance^a^ |
| Estimation Method | REML |
| Standard Error Adjustment | None |
| a. Random-effects weights including both within- and between-study variance. | |

| **Case Processing Summary** | | |
| --- | --- | --- |
|  | N | Percent |
| Included | 5 | 100,0% |
| Missing | 0 | 0,0% |
| Invalid^a^ | 0 | 0,0% |
| Total | 5 | 100,0% |
| a. Nonpositive variance or standard error, or insufficient study size. | | |

| **Effect Size Estimates** | | | | | | |
| --- | --- | --- | --- | --- | --- | --- |
|  | Effect Size | Std. Error | Z | Sig. (2-tailed) | 95% Confidence Interval | |
|  |  |  |  |  | Lower | Upper |
| Overall | -,102 | ,1432 | -,714 | ,475 | -,383 | ,178 |

| **Egger's Regression-Based Test**^a^ | | | | | | |
| --- | --- | --- | --- | --- | --- | --- |
| Parameter | Coefficient | Std. Error | t | Sig. (2-tailed) | 95% Confidence Interval | |
|  |  |  |  |  | Lower | Upper |
| (Intercept) | -,354 | ,9204 | -,385 | ,726 | -3,283 | 2,575 |
| SE^b^ | ,796 | 2,8753 | ,277 | ,800 | -8,354 | 9,947 |
| a. Random-effects meta-regression | | | | | | |
| b. Standard error of effect size | | | | | | |

**pharma_glutamatergic**

| **Meta-Analysis Summary** | |
| --- | --- |
| Data Type | Raw |
| Outcome Type | Continuous |
| Effect Size Measure | Hedges' g |
| Model | Random-effects |
| Weight | Inverse-variance^a^ |
| Estimation Method | REML |
| Standard Error Adjustment | None |
| a. Random-effects weights including both within- and between-study variance. | |

| **Case Processing Summary** | | |
| --- | --- | --- |
|  | N | Percent |
| Included | 23 | 100,0% |
| Missing | 0 | 0,0% |
| Invalid^a^ | 0 | 0,0% |
| Total | 23 | 100,0% |
| a. Nonpositive variance or standard error, or insufficient study size. | | |

| **Effect Size Estimates** | | | | | | |
| --- | --- | --- | --- | --- | --- | --- |
|  | Effect Size | Std. Error | Z | Sig. (2-tailed) | 95% Confidence Interval | |
|  |  |  |  |  | Lower | Upper |
| Overall | -,483 | ,1690 | -2,855 | ,004 | -,814 | -,151 |

| **Egger's Regression-Based Test**^a^ | | | | | | |
| --- | --- | --- | --- | --- | --- | --- |
| Parameter | Coefficient | Std. Error | t | Sig. (2-tailed) | 95% Confidence Interval | |
|  |  |  |  |  | Lower | Upper |
| (Intercept) | ,859 | ,3827 | 2,245 | ,036 | ,063 | 1,655 |
| SE^b^ | -4,558 | 1,2441 | -3,664 | ,001 | -7,145 | -1,971 |
| a. Random-effects meta-regression | | | | | | |
| b. Standard error of effect size | | | | | | |

**pharma_hormones**

| **Meta-Analysis Summary** | |
| --- | --- |
| Data Type | Raw |
| Outcome Type | Continuous |
| Effect Size Measure | Hedges' g |
| Model | Random-effects |
| Weight | Inverse-variance^a^ |
| Estimation Method | REML |
| Standard Error Adjustment | None |
| a. Random-effects weights including both within- and between-study variance. | |

| **Case Processing Summary** | | |
| --- | --- | --- |
|  | N | Percent |
| Included | 30 | 100,0% |
| Missing | 0 | 0,0% |
| Invalid^a^ | 0 | 0,0% |
| Total | 30 | 100,0% |
| a. Nonpositive variance or standard error, or insufficient study size. | | |

| **Effect Size Estimates** | | | | | | |
| --- | --- | --- | --- | --- | --- | --- |
|  | Effect Size | Std. Error | Z | Sig. (2-tailed) | 95% Confidence Interval | |
|  |  |  |  |  | Lower | Upper |
| Overall | -,283 | ,0803 | -3,523 | <,001 | -,440 | -,125 |

| **Egger's Regression-Based Test**^a^ | | | | | | |
| --- | --- | --- | --- | --- | --- | --- |
| Parameter | Coefficient | Std. Error | t | Sig. (2-tailed) | 95% Confidence Interval | |
|  |  |  |  |  | Lower | Upper |
| (Intercept) | -,413 | ,2636 | -1,565 | ,129 | -,953 | ,127 |
| SE^b^ | ,434 | ,8374 | ,519 | ,608 | -1,281 | 2,150 |
| a. Random-effects meta-regression | | | | | | |
| b. Standard error of effect size | | | | | | |

**pharma_immunomodulator**

| **Meta-Analysis Summary** | |
| --- | --- |
| Data Type | Raw |
| Outcome Type | Continuous |
| Effect Size Measure | Hedges' g |
| Model | Random-effects |
| Weight | Inverse-variance^a^ |
| Estimation Method | REML |
| Standard Error Adjustment | None |
| a. Random-effects weights including both within- and between-study variance. | |

| **Case Processing Summary** | | |
| --- | --- | --- |
|  | N | Percent |
| Included | 9 | 100,0% |
| Missing | 0 | 0,0% |
| Invalid^a^ | 0 | 0,0% |
| Total | 9 | 100,0% |
| a. Nonpositive variance or standard error, or insufficient study size. | | |

| **Effect Size Estimates** | | | | | | |
| --- | --- | --- | --- | --- | --- | --- |
|  | Effect Size | Std. Error | Z | Sig. (2-tailed) | 95% Confidence Interval | |
|  |  |  |  |  | Lower | Upper |
| Overall | -,558 | ,1840 | -3,033 | ,002 | -,919 | -,197 |

| **Egger's Regression-Based Test**^a^ | | | | | | |
| --- | --- | --- | --- | --- | --- | --- |
| Parameter | Coefficient | Std. Error | t | Sig. (2-tailed) | 95% Confidence Interval | |
|  |  |  |  |  | Lower | Upper |
| (Intercept) | -1,529 | ,6680 | -2,289 | ,056 | -3,109 | ,050 |
| SE^b^ | 3,380 | 2,2635 | 1,493 | ,179 | -1,973 | 8,732 |
| a. Random-effects meta-regression | | | | | | |
| b. Standard error of effect size | | | | | | |

**pharma_other**

| **Meta-Analysis Summary** | |
| --- | --- |
| Data Type | Raw |
| Outcome Type | Continuous |
| Effect Size Measure | Hedges' g |
| Model | Random-effects |
| Weight | Inverse-variance^a^ |
| Estimation Method | REML |
| Standard Error Adjustment | None |
| a. Random-effects weights including both within- and between-study variance. | |

| **Case Processing Summary** | | |
| --- | --- | --- |
|  | N | Percent |
| Included | 44 | 100,0% |
| Missing | 0 | 0,0% |
| Invalid^a^ | 0 | 0,0% |
| Total | 44 | 100,0% |
| a. Nonpositive variance or standard error, or insufficient study size. | | |

| **Effect Size Estimates** | | | | | | |
| --- | --- | --- | --- | --- | --- | --- |
|  | Effect Size | Std. Error | Z | Sig. (2-tailed) | 95% Confidence Interval | |
|  |  |  |  |  | Lower | Upper |
| Overall | -,459 | ,1413 | -3,249 | ,001 | -,736 | -,182 |

| **Egger's Regression-Based Test**^a^ | | | | | | |
| --- | --- | --- | --- | --- | --- | --- |
| Parameter | Coefficient | Std. Error | t | Sig. (2-tailed) | 95% Confidence Interval | |
|  |  |  |  |  | Lower | Upper |
| (Intercept) | -,951 | ,4630 | -2,054 | ,046 | -1,885 | -,017 |
| SE^b^ | 1,734 | 1,5540 | 1,116 | ,271 | -1,402 | 4,870 |
| a. Random-effects meta-regression | | | | | | |
| b. Standard error of effect size | | | | | | |

**pharma_statin**

| **Meta-Analysis Summary** | |
| --- | --- |
| Data Type | Raw |
| Outcome Type | Continuous |
| Effect Size Measure | Hedges' g |
| Model | Random-effects |
| Weight | Inverse-variance^a^ |
| Estimation Method | REML |
| Standard Error Adjustment | None |
| a. Random-effects weights including both within- and between-study variance. | |

| **Case Processing Summary** | | |
| --- | --- | --- |
|  | N | Percent |
| Included | 7 | 100,0% |
| Missing | 0 | 0,0% |
| Invalid^a^ | 0 | 0,0% |
| Total | 7 | 100,0% |
| a. Nonpositive variance or standard error, or insufficient study size. | | |

| **Effect Size Estimates** | | | | | | |
| --- | --- | --- | --- | --- | --- | --- |
|  | Effect Size | Std. Error | Z | Sig. (2-tailed) | 95% Confidence Interval | |
|  |  |  |  |  | Lower | Upper |
| Overall | -,294 | ,1259 | -2,332 | ,020 | -,540 | -,047 |

| **Egger's Regression-Based Test**^a^ | | | | | | |
| --- | --- | --- | --- | --- | --- | --- |
| Parameter | Coefficient | Std. Error | t | Sig. (2-tailed) | 95% Confidence Interval | |
|  |  |  |  |  | Lower | Upper |
| (Intercept) | -,230 | ,4043 | -,570 | ,593 | -1,270 | ,809 |
| SE^b^ | -,240 | 1,4214 | -,169 | ,872 | -3,894 | 3,413 |
| a. Random-effects meta-regression | | | | | | |
| b. Standard error of effect size | | | | | | |

**pharma_stimulant**

| **Meta-Analysis Summary** | |
| --- | --- |
| Data Type | Raw |
| Outcome Type | Continuous |
| Effect Size Measure | Hedges' g |
| Model | Random-effects |
| Weight | Inverse-variance^a^ |
| Estimation Method | REML |
| Standard Error Adjustment | None |
| a. Random-effects weights including both within- and between-study variance. | |

| **Case Processing Summary** | | |
| --- | --- | --- |
|  | N | Percent |
| Included | 13 | 100,0% |
| Missing | 0 | 0,0% |
| Invalid^a^ | 0 | 0,0% |
| Total | 13 | 100,0% |
| a. Nonpositive variance or standard error, or insufficient study size. | | |

| **Effect Size Estimates** | | | | | | |
| --- | --- | --- | --- | --- | --- | --- |
|  | Effect Size | Std. Error | Z | Sig. (2-tailed) | 95% Confidence Interval | |
|  |  |  |  |  | Lower | Upper |
| Overall | -,491 | ,1962 | -2,504 | ,012 | -,876 | -,107 |

| **Egger's Regression-Based Test**^a^ | | | | | | |
| --- | --- | --- | --- | --- | --- | --- |
| Parameter | Coefficient | Std. Error | t | Sig. (2-tailed) | 95% Confidence Interval | |
|  |  |  |  |  | Lower | Upper |
| (Intercept) | ,018 | ,6078 | ,029 | ,977 | -1,320 | 1,355 |
| SE^b^ | -1,510 | 1,7060 | -,885 | ,395 | -5,265 | 2,245 |
| a. Random-effects meta-regression | | | | | | |
| b. Standard error of effect size | | | | | | |

**pharma_vitamins_nutraceutic**

| **Meta-Analysis Summary** | |
| --- | --- |
| Data Type | Raw |
| Outcome Type | Continuous |
| Effect Size Measure | Hedges' g |
| Model | Random-effects |
| Weight | Inverse-variance^a^ |
| Estimation Method | REML |
| Standard Error Adjustment | None |
| a. Random-effects weights including both within- and between-study variance. | |

| **Case Processing Summary** | | |
| --- | --- | --- |
|  | N | Percent |
| Included | 45 | 100,0% |
| Missing | 0 | 0,0% |
| Invalid^a^ | 0 | 0,0% |
| Total | 45 | 100,0% |
| a. Nonpositive variance or standard error, or insufficient study size. | | |

| **Effect Size Estimates** | | | | | | |
| --- | --- | --- | --- | --- | --- | --- |
|  | Effect Size | Std. Error | Z | Sig. (2-tailed) | 95% Confidence Interval | |
|  |  |  |  |  | Lower | Upper |
| Overall | -,351 | ,0721 | -4,871 | <,001 | -,493 | -,210 |

| **Egger's Regression-Based Test**^a^ | | | | | | |
| --- | --- | --- | --- | --- | --- | --- |
| Parameter | Coefficient | Std. Error | t | Sig. (2-tailed) | 95% Confidence Interval | |
|  |  |  |  |  | Lower | Upper |
| (Intercept) | 5,703E-5 | ,2536 | ,000 | 1,000 | -,511 | ,512 |
| SE^b^ | -1,287 | ,8946 | -1,438 | ,158 | -3,091 | ,518 |
| a. Random-effects meta-regression | | | | | | |
| b. Standard error of effect size | | | | | | |

**psych_art**

| **Meta-Analysis Summary** | |
| --- | --- |
| Data Type | Raw |
| Outcome Type | Continuous |
| Effect Size Measure | Hedges' g |
| Model | Random-effects |
| Weight | Inverse-variance^a^ |
| Estimation Method | REML |
| Standard Error Adjustment | None |
| a. Random-effects weights including both within- and between-study variance. | |

| **Case Processing Summary** | | |
| --- | --- | --- |
|  | N | Percent |
| Included | 8 | 100,0% |
| Missing | 0 | 0,0% |
| Invalid^a^ | 0 | 0,0% |
| Total | 8 | 100,0% |
| a. Nonpositive variance or standard error, or insufficient study size. | | |

| **Effect Size Estimates** | | | | | | |
| --- | --- | --- | --- | --- | --- | --- |
|  | Effect Size | Std. Error | Z | Sig. (2-tailed) | 95% Confidence Interval | |
|  |  |  |  |  | Lower | Upper |
| Overall | -,574 | ,2009 | -2,859 | ,004 | -,968 | -,181 |

| **Egger's Regression-Based Test**^a^ | | | | | | |
| --- | --- | --- | --- | --- | --- | --- |
| Parameter | Coefficient | Std. Error | t | Sig. (2-tailed) | 95% Confidence Interval | |
|  |  |  |  |  | Lower | Upper |
| (Intercept) | ,683 | 1,1269 | ,606 | ,567 | -2,075 | 3,440 |
| SE^b^ | -5,604 | 4,9473 | -1,133 | ,301 | -17,709 | 6,502 |
| a. Random-effects meta-regression | | | | | | |
| b. Standard error of effect size | | | | | | |

**psych_cog_cbt**

| **Meta-Analysis Summary** | |
| --- | --- |
| Data Type | Raw |
| Outcome Type | Continuous |
| Effect Size Measure | Hedges' g |
| Model | Random-effects |
| Weight | Inverse-variance^a^ |
| Estimation Method | REML |
| Standard Error Adjustment | None |
| a. Random-effects weights including both within- and between-study variance. | |

| **Case Processing Summary** | | |
| --- | --- | --- |
|  | N | Percent |
| Included | 23 | 100,0% |
| Missing | 0 | 0,0% |
| Invalid^a^ | 0 | 0,0% |
| Total | 23 | 100,0% |
| a. Nonpositive variance or standard error, or insufficient study size. | | |

| **Effect Size Estimates** | | | | | | |
| --- | --- | --- | --- | --- | --- | --- |
|  | Effect Size | Std. Error | Z | Sig. (2-tailed) | 95% Confidence Interval | |
|  |  |  |  |  | Lower | Upper |
| Overall | -,304 | ,0796 | -3,813 | <,001 | -,460 | -,148 |

| **Egger's Regression-Based Test**^a^ | | | | | | |
| --- | --- | --- | --- | --- | --- | --- |
| Parameter | Coefficient | Std. Error | t | Sig. (2-tailed) | 95% Confidence Interval | |
|  |  |  |  |  | Lower | Upper |
| (Intercept) | ,120 | ,1865 | ,644 | ,527 | -,268 | ,508 |
| SE^b^ | -1,742 | ,7223 | -2,411 | ,025 | -3,244 | -,240 |
| a. Random-effects meta-regression | | | | | | |
| b. Standard error of effect size | | | | | | |

**psych_cognitive_remediation**

| **Meta-Analysis Summary** | |
| --- | --- |
| Data Type | Raw |
| Outcome Type | Continuous |
| Effect Size Measure | Hedges' g |
| Model | Random-effects |
| Weight | Inverse-variance^a^ |
| Estimation Method | REML |
| Standard Error Adjustment | None |
| a. Random-effects weights including both within- and between-study variance. | |

| **Case Processing Summary** | | |
| --- | --- | --- |
|  | N | Percent |
| Included | 34 | 100,0% |
| Missing | 0 | 0,0% |
| Invalid^a^ | 0 | 0,0% |
| Total | 34 | 100,0% |
| a. Nonpositive variance or standard error, or insufficient study size. | | |

| **Effect Size Estimates** | | | | | | |
| --- | --- | --- | --- | --- | --- | --- |
|  | Effect Size | Std. Error | Z | Sig. (2-tailed) | 95% Confidence Interval | |
|  |  |  |  |  | Lower | Upper |
| Overall | -,034 | ,0493 | -,693 | ,488 | -,131 | ,062 |

| **Egger's Regression-Based Test^a^** | | | | | | |
| --- | --- | --- | --- | --- | --- | --- |
| Parameter | Coefficient | Std. Error | t | Sig. (2-tailed) | 95% Confidence Interval | |
|  |  |  |  |  | Lower | Upper |
| (Intercept) | -,117 | ,2089 | -,560 | ,579 | -,543 | ,309 |
| SE^b^ | ,297 | ,7269 | ,408 | ,686 | -1,184 | 1,777 |
| a. Random-effects meta-regression | | | | | | |
| b. Standard error of effect size | | | | | | |


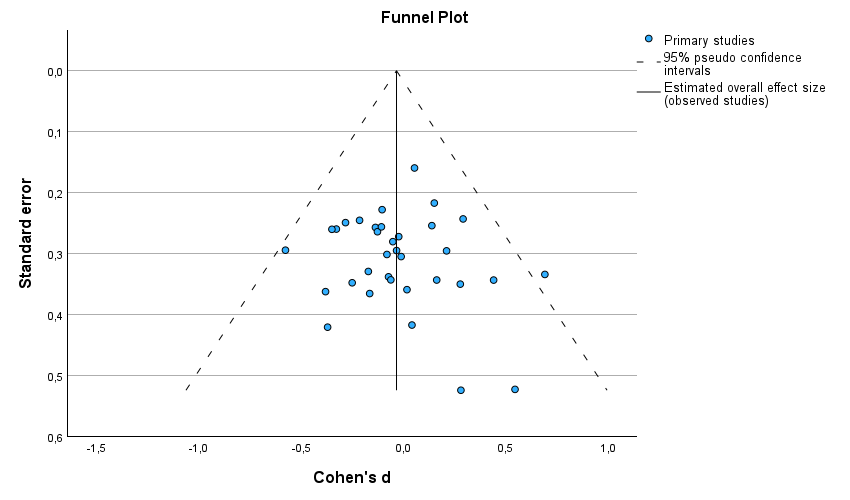


**psych_integrated**

| **Meta-Analysis Summary** | |
| --- | --- |
| Data Type | Raw |
| Outcome Type | Continuous |
| Effect Size Measure | Hedges' g |
| Model | Random-effects |
| Weight | Inverse-variance^a^ |
| Estimation Method | REML |
| Standard Error Adjustment | None |
| a. Random-effects weights including both within- and between-study variance. | |

| **Case Processing Summary** | | |
| --- | --- | --- |
|  | N | Percent |
| Included | 16 | 100,0% |
| Missing | 0 | 0,0% |
| Invalid^a^ | 0 | 0,0% |
| Total | 16 | 100,0% |
| a. Nonpositive variance or standard error, or insufficient study size. | | |

| **Effect Size Estimates** | | | | | | |
| --- | --- | --- | --- | --- | --- | --- |
|  | Effect Size | Std. Error | Z | Sig. (2-tailed) | 95% Confidence Interval | |
|  |  |  |  |  | Lower | Upper |
| Overall | -,013 | ,0509 | -,256 | ,798 | -,113 | ,087 |

| **Egger's Regression-Based Test^a^** | | | | | | |
| --- | --- | --- | --- | --- | --- | --- |
| Parameter | Coefficient | Std. Error | t | Sig. (2-tailed) | 95% Confidence Interval | |
|  |  |  |  |  | Lower | Upper |
| (Intercept) | -,076 | ,1445 | -,527 | ,607 | -,386 | ,234 |
| SE^b^ | ,321 | ,6570 | ,488 | ,633 | -1,088 | 1,730 |
| a. Random-effects meta-regression | | | | | | |
| b. Standard error of effect size | | | | | | |


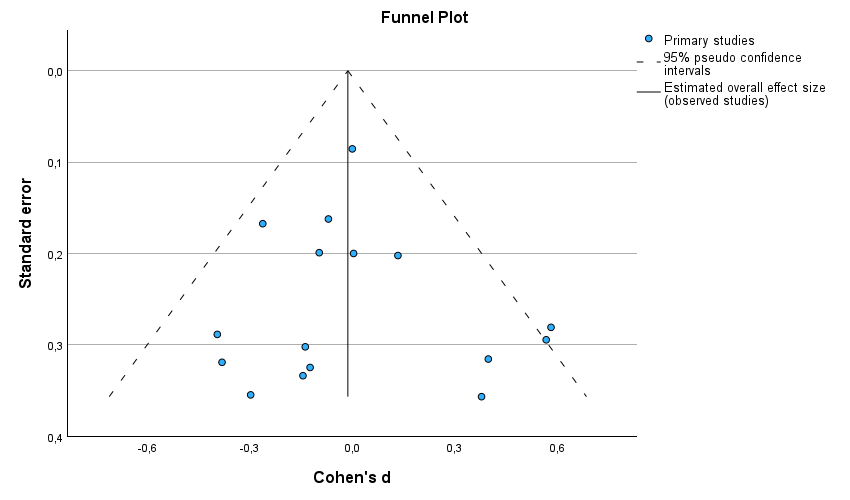


**psych_mindfulness**

| **Meta-Analysis Summary** | |
| --- | --- |
| Data Type | Raw |
| Outcome Type | Continuous |
| Effect Size Measure | Hedges' g |
| Model | Random-effects |
| Weight | Inverse-variance^a^ |
| Estimation Method | REML |
| Standard Error Adjustment | None |
| a. Random-effects weights including both within- and between-study variance. | |

| **Case Processing Summary** | | |
| --- | --- | --- |
|  | N | Percent |
| Included | 17 | 100,0% |
| Missing | 0 | 0,0% |
| Invalid^a^ | 0 | 0,0% |
| Total | 17 | 100,0% |
| a. Nonpositive variance or standard error, or insufficient study size. | | |

| **Effect Size Estimates** | | | | | | |
| --- | --- | --- | --- | --- | --- | --- |
|  | Effect Size | Std. Error | Z | Sig. (2-tailed) | 95% Confidence Interval | |
|  |  |  |  |  | Lower | Upper |
| Overall | -,806 | ,2440 | -3,305 | <,001 | -1,285 | -,328 |

| **Egger's Regression-Based Test**^a^ | | | | | | |
| --- | --- | --- | --- | --- | --- | --- |
| Parameter | Coefficient | Std. Error | t | Sig. (2-tailed) | 95% Confidence Interval | |
|  |  |  |  |  | Lower | Upper |
| (Intercept) | ,784 | ,9549 | ,821 | ,424 | -1,251 | 2,819 |
| SE^b^ | -5,725 | 3,3395 | -1,714 | ,107 | -12,843 | 1,393 |
| a. Random-effects meta-regression | | | | | | |
| b. Standard error of effect size | | | | | | |

**psych_psychoeducation_support**

| **Meta-Analysis Summary** | |
| --- | --- |
| Data Type | Raw |
| Outcome Type | Continuous |
| Effect Size Measure | Hedges' g |
| Model | Random-effects |
| Weight | Inverse-variance^a^ |
| Estimation Method | REML |
| Standard Error Adjustment | None |
| a. Random-effects weights including both within- and between-study variance. | |

| **Case Processing Summary** | | |
| --- | --- | --- |
|  | N | Percent |
| Included | 14 | 100,0% |
| Missing | 0 | 0,0% |
| Invalid^a^ | 0 | 0,0% |
| Total | 14 | 100,0% |
| a. Nonpositive variance or standard error, or insufficient study size. | | |

| **Effect Size Estimates** | | | | | | |
| --- | --- | --- | --- | --- | --- | --- |
|  | Effect Size | Std. Error | Z | Sig. (2-tailed) | 95% Confidence Interval | |
|  |  |  |  |  | Lower | Upper |
| Overall | -,394 | ,1131 | -3,483 | <,001 | -,616 | -,172 |

| **Egger's Regression-Based Test**^a^ | | | | | | |
| --- | --- | --- | --- | --- | --- | --- |
| Parameter | Coefficient | Std. Error | t | Sig. (2-tailed) | 95% Confidence Interval | |
|  |  |  |  |  | Lower | Upper |
| (Intercept) | -,243 | ,5853 | -,416 | ,685 | -1,519 | 1,032 |
| SE^b^ | -,562 | 2,1358 | -,263 | ,797 | -5,215 | 4,092 |
| a. Random-effects meta-regression | | | | | | |
| b. Standard error of effect size | | | | | | |

**psych_social_skills**

| **Meta-Analysis Summary** | |
| --- | --- |
| Data Type | Raw |
| Outcome Type | Continuous |
| Effect Size Measure | Hedges' g |
| Model | Random-effects |
| Weight | Inverse-variance^a^ |
| Estimation Method | REML |
| Standard Error Adjustment | None |
| a. Random-effects weights including both within- and between-study variance. | |

| **Case Processing Summary** | | |
| --- | --- | --- |
|  | N | Percent |
| Included | 16 | 100,0% |
| Missing | 0 | 0,0% |
| Invalid^a^ | 0 | 0,0% |
| Total | 16 | 100,0% |
| a. Nonpositive variance or standard error, or insufficient study size. | | |

| **Effect Size Estimates** | | | | | | |
| --- | --- | --- | --- | --- | --- | --- |
|  | Effect Size | Std. Error | Z | Sig. (2-tailed) | 95% Confidence Interval | |
|  |  |  |  |  | Lower | Upper |
| Overall | -,013 | ,0509 | -,256 | ,798 | -,113 | ,087 |

| **Egger's Regression-Based Test^a^** | | | | | | |
| --- | --- | --- | --- | --- | --- | --- |
| Parameter | Coefficient | Std. Error | t | Sig. (2-tailed) | 95% Confidence Interval | |
|  |  |  |  |  | Lower | Upper |
| (Intercept) | -,076 | ,1445 | -,527 | ,607 | -,386 | ,234 |
| SE^b^ | ,321 | ,6570 | ,488 | ,633 | -1,088 | 1,730 |
| a. Random-effects meta-regression | | | | | | |
| b. Standard error of effect size | | | | | | |


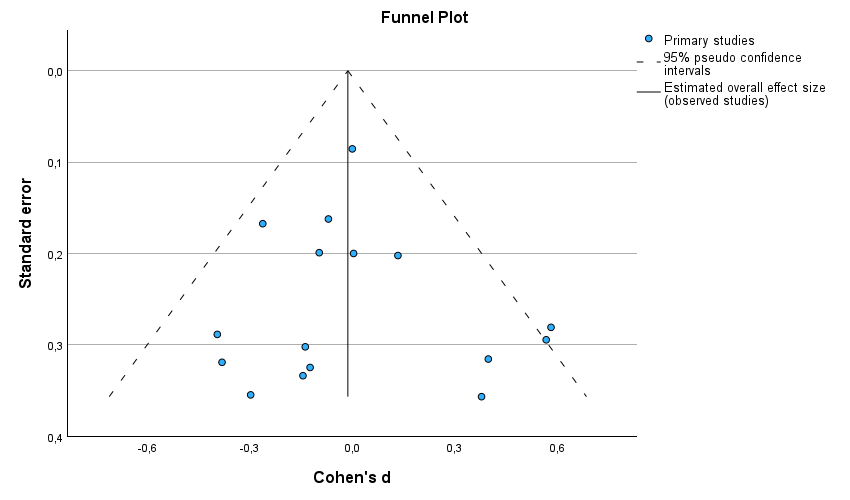


**stimulation_TCS**

| **Meta-Analysis Summary** | |
| --- | --- |
| Data Type | Raw |
| Outcome Type | Continuous |
| Effect Size Measure | Hedges' g |
| Model | Random-effects |
| Weight | Inverse-variance^a^ |
| Estimation Method | REML |
| Standard Error Adjustment | None |
| a. Random-effects weights including both within- and between-study variance. | |

| **Case Processing Summary** | | |
| --- | --- | --- |
|  | N | Percent |
| Included | 14 | 100,0% |
| Missing | 0 | 0,0% |
| Invalid^a^ | 0 | 0,0% |
| Total | 14 | 100,0% |
| a. Nonpositive variance or standard error, or insufficient study size. | | |

| **Effect Size Estimates** | | | | | | |
| --- | --- | --- | --- | --- | --- | --- |
|  | Effect Size | Std. Error | Z | Sig. (2-tailed) | 95% Confidence Interval | |
|  |  |  |  |  | Lower | Upper |
| Overall | -,514 | ,1770 | -2,903 | ,004 | -,861 | -,167 |

| **Egger's Regression-Based Test**^a^ | | | | | | |
| --- | --- | --- | --- | --- | --- | --- |
| Parameter | Coefficient | Std. Error | t | Sig. (2-tailed) | 95% Confidence Interval | |
|  |  |  |  |  | Lower | Upper |
| (Intercept) | ,259 | ,7988 | ,325 | ,751 | -1,481 | 2,000 |
| SE^b^ | -2,351 | 2,3664 | -,994 | ,340 | -7,507 | 2,805 |
| a. Random-effects meta-regression | | | | | | |
| b. Standard error of effect size | | | | | | |

**stimulation_TMS**

| **Meta-Analysis Summary** | |
| --- | --- |
| Data Type | Raw |
| Outcome Type | Continuous |
| Effect Size Measure | Hedges' g |
| Model | Random-effects |
| Weight | Inverse-variance^a^ |
| Estimation Method | REML |
| Standard Error Adjustment | None |
| a. Random-effects weights including both within- and between-study variance. | |

| **Case Processing Summary** | | |
| --- | --- | --- |
|  | N | Percent |
| Included | 41 | 100,0% |
| Missing | 0 | 0,0% |
| Invalid^a^ | 0 | 0,0% |
| Total | 41 | 100,0% |
| a. Nonpositive variance or standard error, or insufficient study size. | | |

| **Effect Size Estimates** | | | | | | |
| --- | --- | --- | --- | --- | --- | --- |
|  | Effect Size | Std. Error | Z | Sig. (2-tailed) | 95% Confidence Interval | |
|  |  |  |  |  | Lower | Upper |
| Overall | -,259 | ,0930 | -2,784 | ,005 | -,441 | -,077 |

| **Egger's Regression-Based Test**^a^ | | | | | | |
| --- | --- | --- | --- | --- | --- | --- |
| Parameter | Coefficient | Std. Error | t | Sig. (2-tailed) | 95% Confidence Interval | |
|  |  |  |  |  | Lower | Upper |
| (Intercept) | -,159 | ,3651 | -,435 | ,666 | -,897 | ,580 |
| SE^b^ | -,327 | 1,1530 | -,284 | ,778 | -2,660 | 2,005 |
| a. Random-effects meta-regression | | | | | | |
| b. Standard error of effect size | | | | | | |
